# Role of HRTPT in kidney proximal epithelial cell regeneration: Integrative differential expression and pathway analyses using microarray and scRNA‐seq

**DOI:** 10.1111/jcmm.16976

**Published:** 2021-10-09

**Authors:** Swojani Shrestha, Sonalika Singhal, Matthew Kalonick, Rachel Guyer, Alexis Volkert, Seema Somji, Scott H. Garrett, Donald A. Sens, Sandeep K. Singhal

**Affiliations:** ^1^ Department of Pathology School of Medicine and Health Sciences University of North Dakota Grand Forks North Dakota USA; ^2^ Department of Biomedical Engineering School of Electrical Engineering and Computer Science University of North Dakota Grand Forks North Dakota USA

**Keywords:** CD133, CD24, Cell regeneration, Human renal epithelial cell 24 TERT (HREC24T) cells, Human renal tubular precursor tert (HRTPT) cells, Microarray, renal progenitor cells

## Abstract

Damage to proximal tubules due to exposure to toxicants can lead to conditions such as acute kidney injury (AKI), chronic kidney disease (CKD) and ultimately end‐stage renal failure (ESRF). Studies have shown that kidney proximal epithelial cells can regenerate particularly after acute injury. In the previous study, we utilized an immortalized *in vitro* model of human renal proximal tubule epithelial cells, RPTEC/TERT1, to isolate HRTPT cell line that co‐expresses stem cell markers CD133 and CD24, and HREC24T cell line that expresses only CD24. HRTPT cells showed most of the key characteristics of stem/progenitor cells; however, HREC24T cells did not show any of these characteristics. The goal of this study was to further characterize and understand the global gene expression differences, upregulated pathways and gene interaction using scRNA‐seq in HRTPT cells. Affymetrix microarray analysis identified common gene sets and pathways specific to HRTPT and HREC24T cells analysed using DAVID, Reactome and Ingenuity software. Gene sets of HRTPT cells, in comparison with publicly available data set for CD133+ infant kidney, urine‐derived renal progenitor cells and human kidney‐derived epithelial proximal tubule cells showed substantial similarity in organization and interactions of the apical membrane. Single‐cell analysis of HRTPT cells identified unique gene clusters associated with CD133 and the 92 common gene sets from three data sets. In conclusion, the gene expression analysis identified a unique gene set for HRTPT cells and narrowed the co‐expressed gene set compared with other human renal–derived cell lines expressing CD133, which may provide deeper understanding in their role as progenitor/stem cells that participate in renal repair.

**Significant statement**

Cultures of human renal epithelial cells and their tert‐immortalized counterparts routinely express the surface markers of renal progenitor cells, CD24 and CD133. All cells express CD24, but a large minority fraction does not express CD133. Only those cells that express both markers have full differentiation potential. Here, we compare the global gene expression of CD24+/CD133+ (HRTPT) with cells that express only CD24+ (HREC24T). The CD133‐specific gene set was used to compare other human renal progenitor cell global gene expression data sets: infant progenitor cells and renal progenitor cells isolated from urine. A common gene set consisting of 92 genes was identified that defines the organization and interactions of the apical membrane. This gene set will provide a deeper understanding of the regulation of the renal progenitor cell population and the process of renal repair.

## 1 INTRODUCTION

Acute kidney injury (AKI), or synonymously acute tubular necrosis (ATN), is associated with an abrupt decline in renal function and is reported to have a mortality rate ranging from 18% to 80% depending on the severity of the insult.^1^, ^2^ The progression of AKI to chronic (CKD) or even end‐stage renal disease (ESRD) correlates with the duration and severity of the acute injury.^3^ It is well documented that the human kidney has the ability to regenerate damaged nephrons following AKI.^4^ AKI primarily damages the cells comprising the proximal tubules, and it is these tubules where regeneration and repair are most evident. The ability of renal regeneration following AKI offers the potential opportunity for enhanced repair with slowing or eliminating progression to CKD. There is strong evidence that the cells that participate in tubule cell renewal are generated from within the kidney itself and are not recruited from non‐renal sources.^5^, ^6^ Whether these cells are always present within the kidney or result from dedifferentiation of existing cells is presently not known with absolute certainty. A number of studies have shown that the progenitor cells capable of regenerating renal tubules, regardless of origin, are characterized by the co‐expression of surface markers CD133 and CD24.^5^, ^6^, ^7^, ^8^, ^9^, ^10^ The CD133+/CD24+ cells can proliferate in primary cell culture with retainment of phenotype and having the capacity to differentiate both in vivo and in vitro. In the developing human kidney, the CD133+ cells are a subset of CD24+ cells, which constitute the metanephric mesenchyme‐derived primordial nephron.^11^

The study of human CD133+/CD24+ renal progenitor cells is difficult due to the acquisition of human tissue, the small population of such cells within the kidney, their limited duration of viability following direct isolation and their limited lifespan in primary culture. Using flow cytometry, this laboratory demonstrated that a human renal proximal epithelial cell line (RPTEC/TERT), which was isolated from renal cortical tissue and subsequently immortalized by transduction with human telomerase reverse transcriptase (hTERT), that consists of approximately 70% of the cell population expressed both CD133 and CD24, while the remainder expressed CD24 but not CD133.^12^, ^13^, ^14^ The laboratory then employed cell sorting to establish a cell line that is composed of over 95% of cells expressing both CD133 and CD24. The HRTPT cells were shown to maintain the co‐expression of CD133 and CD24 and cell phenotype following extended subculture. The HRTPT cells formed multicellular spheroids (nephrospheres), a characteristic feature of stem/progenitor cells, and formed branched tubule‐like structures when grown on the surface of Matrigel and were able to grow and undergo neurogenic, adipogenic, osteogenic and tubulogenic differentiation. An identical protocol was used to generate a cell line from the RPTEC/TERT cell line that expressed CD24 in the absence of CD133.^12^, ^13^ This cell line, HREC24T, maintained its phenotype and the expression of CD24 and not CD133 over extended serial culture, but did not undergo spheroid formation and differentiation on the surface of Matrigel, nor neurogenic, adipogenic or osteogenic differentiation.

The objectives of the present study were to further characterize the gene expression of the HRTPT cells that might further define their role in renal epithelial cell regeneration. The first objective was to identify the differences in global gene expression when the HRTPT progenitor cells were compared to the HREC24T cells that do not display progenitor cell properties. The second objective was to use the differences in global gene expression to define cellular functions and pathways specific to each cell line. A third objective was to compare the CD133/24 co‐expressing gene derived from the above comparison of HRTPT and HREC24T cell lines with publically available databases of renal CD133 expressing cells. The final objective was to determine whether the HRTPT cell line harbours a single or multiple CD133 expressing progenitor cell populations.

## 2 MATERIALS AND METHODS

### 2.1 Cell culture

Stock cultures of RPTEC/TERT1 cells were obtained from American Type Culture Collection and were grown using serum‐free conditions as previously described by this laboratory.^15^, ^16^ The composition of the growth formulation was also as described previously.^12^, ^13^, ^15^ Confluent cultures of the immortalized RPTEC/TERT1 cell line were sorted into two different cell populations, namely HRTPT (CD133+/CD24+) cells and HREC24T (CD133‐CD24+) cells, using BD FACSAria (BD Biosciences**)** as detailed previously.^12^ Both cell lines were subcultured at 1:3 ratio, allowed to reach confluence and then used in the described experimental protocols. For treatment with FGFR inhibitor, SU5402 (Selleck Chemicals), HRTPT cells were grown to confluency and subcultured into growth media containing 5uM SU5402 inhibitor or DMSO for control, grown to confluency and harvested to obtain pellets for RNA and protein analyses.

### 2.2 Sample preparation for microarray and single‐cell analysis

HRTPT and HREC24T cells were harvested, and their respective RNAs were isolated using RNeasy Mini Kit using the manufacturer's protocol (Qiagen). Triplicate samples for each cell line were diluted to 100 ng/ul, packed in dry ice and transported to Genome Explorations, TN, for microarray analysis. For single‐cell analysis, the sample was prepared using Genewiz 10x genomic chromium single‐cell RNA‐seq protocol. Following confluency, HRTPT cells grown in three separate T‐25 flasks were trypsinized, centrifuged and resuspended in the growth media in separate tubes. To obtain a single‐cell suspension, the cells were gently pipetted up and down for several minutes. The viable cells were counted by mixing with 4x trypan blue using a haemocytometer, diluted to at least 1x 106 cells/ml, centrifuged and resuspended in 500ul of ice‐cold DMEM containing 20% serum and 10% DMSO and then transferred to cryotubes sandwiched between two styrofoam tube holders and stored in the −80 ℃ freezer for at least 4 h. The samples were packed in dry ice and shipped to Genewiz for single‐cell RNA sequencing.

For microarray analysis, the samples were processed and hybridized onto HTA2.0 arrays with a quality control (QC) report provided by the company, which showed all samples passed the QC test. The oligo (https://bioconductor.org/packages/release/bioc/html/oligo.html), frozen RMA and Barcode (https://www.bioconductor.org/packages/release/bioc/html/frma.html) packages of Bioconductor were used to preprocess microarray data using R. The data used for the analysis were log‐transformed and pareto‐scaled. After preprocess and transformation, the density distribution of data shows the uniform distribution across all samples (Figure S5). The robustness of the correlation across samples was tested using the multiscale bootstrap resampling method to avoid any technical/biological bias of data. The single‐cell sequencing data were analysed using the Loupe Browser provided by 10x Genomics (https://www.10xgenomics.com/products/loupe‐browser).

### 2.3 RNA isolation and digital droplet polymerase chain reaction (ddPCR)

Total RNA was isolated using Tri Reagent (Molecular Research Center, Inc.) as described previously.^17^ The measurement of mRNA expression of selected genes was assessed using ddPCR and commercially available primers (Bio‐Rad). The list of primers is provided in Table S1. For analysis, 100 ng of total RNA was subjected to complimentary DNA (cDNA) synthesis using the iScript cDNA Synthesis Kit (Bio‐Rad) in a total volume of 20 μl. Digital droplet PCR was performed utilizing the QX200 Droplet Digital PCR System (Bio‐Rad) that measures absolute copy number per µl of reaction mix. In brief, 12.5ul QX200 ddPCR EvaGreen Supermix, 2.5 ul of cDNA template and 10 ul of PrimePCR SYBR Green assay primers were mixed in the 96‐well plate and placed in the QX200 Droplet Generator. After this step, the plate was sealed and transferred to a 96‐well plate thermal cycler for PCR amplification at an annealing temperature of 60^0^C, and then placed in a QX200 Droplet Reader (Bio‐Rad) instrument, which generates the absolute quantification for each droplet per well. All samples were tested with ddPCR system in triplicates and represented as ±S.D.

### 2.4 Western blot analysis

The cell pellets were lysed using ice‐cold 1x RIPA buffer (Santa Cruz Biotechnology) followed by sonication, and the content was agitated for 30 mins on ice. Then, the tubes were centrifuged at 4^0^C and the supernatant was collected in fresh tubes. Protein concentration was determined using Pierce BCA Protein Assay Kit (Thermo Fisher). The protein levels were assessed using automated protein separation and immunodetection, Jess Simple Western System, and all of the steps were performed using the instructions and reagents provided by the manufacturer (ProteinSimple). In brief, each sample was diluted to 0.5 ug/ul in 0.1x sample buffer, combined with 4x fluorescent master mix in 4:1 ratio and denatured at 95^0^C for 5 mins accompanied by biotin ladder. The tubes were quickly vortexed and centrifuged for a min, and then, the recommended volume for samples, appropriate dilution of primary and secondary antibodies and luminol‐peroxide mix were loaded into a 12–230 kDa Jess 13‐well capillary plate for separation, centrifuged and then ran in the Jess instrument. The list of the primary and secondary antibodies is provided in Table S2. The relative protein expression was determined by the ratio of area under the peak to that of βeta‐actin generated by the Compass for SW software 5.0.1 (ProteinSimple).

### 2.5 Data

To overcome the lack of reference human kidney biopsy‐derived renal progenitors, we performed a meta‐analysis comparing our data with other data sets collected from NCBI GEO. The three data sets have been collected to validate the findings, (1) GSE128281, the microarray data of human urine‐derived renal progenitor cells (udRPCs), primary human renal epithelial proximal cells (hREPCs), udRPCs treated with CHIR99021 and iPSCs derived from udRPCs to human pluripotent cells (H1, H9 and B4); (2) GSE50892, gene expression data from kidney biopsies of liver disease patients; and (3) GSE90628, human infant kidney‐derived cells that were isolated and sorted by FACS for the expression of CD133.

### 2.6 Statistics

The machine‐learning approaches and classifiers were used to understand the sample characteristics; principal component analysis (PCA)^18^ was used to find the sample distribution between the groups. The goal here is to determine the distribution of samples and visualize how the global gene expression profile scattered in different groups. The correlation between the samples was calculated using Pearson's coefficient,^19^ and the heatmap method^20^ was used to plot the correlation coefficient value to find the most correlated samples. The Volcano plot^21^ was used to demonstrate the fold change (log2 ratio) difference against the absolute confidence (‐log10 adjusted *P*‐value) measured between the two groups. Each dot on the plot represents one gene (coloured, significant genes; black, non‐significant). The statistical significance of each gene within each data set was calculated by running t tests^22^ between the categories for conditions. The significance level of *P*‐value ≤0.05 was employed as a standard to filter genes. Further, the pathway and function enrichment analyses of the significant genes were carried out using Ingenuity Pathway Analysis (IPA, QIAGEN Inc.), David and Reactome.^23^, ^24^, ^25^, ^26^

Statistical analysis for ddPCR and protein‐level data consisted of one‐way ANOVA with Tukey's or Sidak's multiple comparisons testing performed by GraphPad Prism 8. All experiments were performed in triplicates, and the data are plotted as the mean ±SE of triplicate determinations.

## 3 RESULTS

### 3.1 Significantly different gene expressions between HRTPT and HREC24T cell lines

**FIGURE 1:**
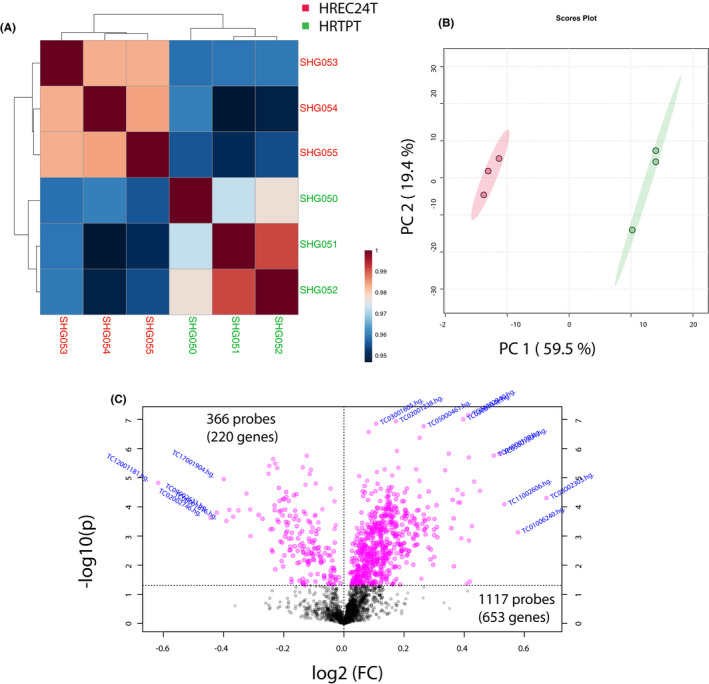
Gene expression data distribution in HRTPT vs HREC24T cells: (A) Pearson's correlations were calculated between each sample, and correlation coefficient values are shown by heatmap. The colour‐coding bar proves the value of correlation coefficient. The dendrogram represents the relation between the samples crated by using the hierarchical clustering approach. (B) The principal component analysis (PCA) plot showing clusters of samples based on similarity. The first two components of PLS‐DA (PC1 and PC2) of gene expression profile and overall variance between the groups are displayed. The red and green colour dots represent HREC24T and HRTPT, respectively. (C) Volcano plot displays the log2 fold change and ‐log10 *P*‐value of gene expression differentiating between HRTPT and HREC24T. Genes with higher than twofold (*P*‐value ≤0.05) are highlighted in red

**FIGURE 2:**
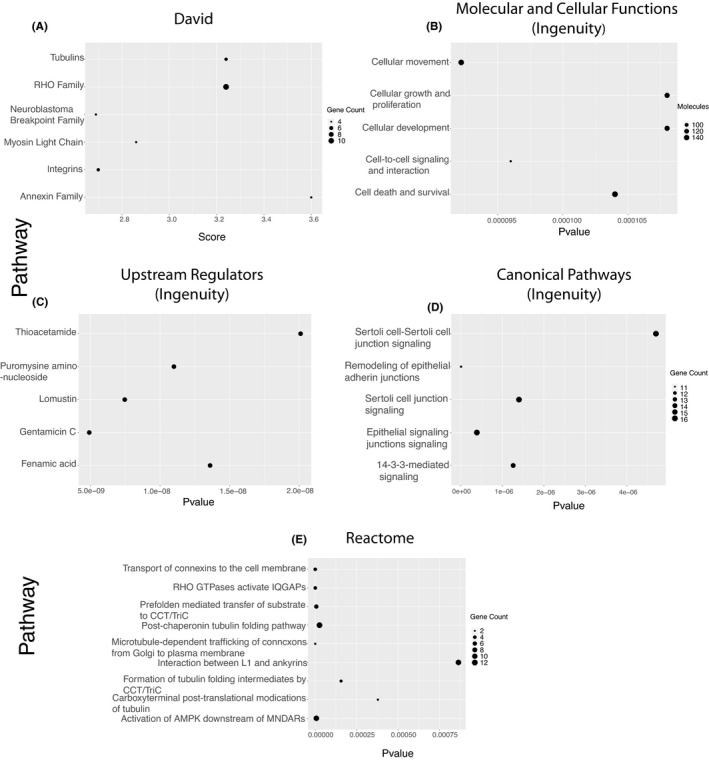
Bubble plot of pathways significantly associated with upregulated HRTPT genes obtained from (A) David analysis, (B) top molecular and cellular function (Ingenuity), (C) top upstream regulators (Ingenuity), (D) top canonical pathway analysis (Ingenuity) and (E) Reactome pathway analysis

The HRTPT cell line is able to differentiate into distinct lineages and form nephrospheres and tubular structures on the surface of Matrigel; however, all of these properties are absent in the HREC24T cell line. Global gene expression analysis on the two cell lines employing the Affymetrix HTA 2.0 chip is demonstrated in Figure 1. The heatmap of hierarchal clustering and principal component shows a clear separation of overall gene expression data between HRTPT and HREC24T cells (Figure 1A and 1B). A total of 1483 probes (873 unique genes) were identified as significant that were specific to the HRTPT cell line with *P*‐value <0.05(Table S3). The gene functional classification using DAVID software identified 16 gene groupings with enrichment scores from 0.15 to 3.61. Six of the gene groups display enrichment scores greater than 2.5 (Figure 2A). A pathway analysis using Reactome produced the 25 most significant pathways with 9 pathways having *P* ≤0.0001 (Figure 2E). An analysis by Ingenuity identified the top 5 canonical pathways out of which remodelling of adherens junctions and junctional signalling was the most prominent, from the unique set of HRTPT genes (Figure 2D). The tubulin genes were identified as a prominent gene grouping using David (Figure 2A), and also prominent entities in the initial 8 pathways were identified using Reactome (Figure 2E). Taken together, the majority of the results indicate that the major difference between the co‐expressing CD133 and CD24 cells when compared to those not expressing CD133 is centred around the organization of the apical membrane and its interaction with the extracellular environment.

### 3.2 Validation of significant genes in HRTPT cells analysed by DAVID, reactome and ingenuity pathway analysis

**FIGURE 3:**
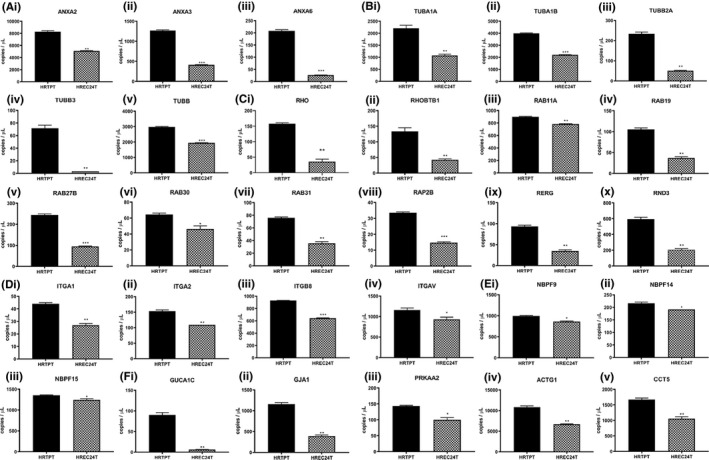
Expression of gene groups (David) in the HRTPT and HREC24T cells. qPCR analysis of A(i) ANXA2, (ii) ANXA3 and (iii) ANXA6; B(i) TUBA1A, (ii) TUBA1B, (iii) TUBB2A, (iv) TUBB3 and (v) TUBB; C(i) RHO, (ii) RHOBTB1, (iii) RAB11A, (iv) RAB19, (v) RAB27B, (vi) RAB30, (vii) RAB31, (viii) RAP2B, (ix) RERG and (x) RND3; D(i) ITGA1, (ii) ITGA2, (iii) ITGB8 and (iv) ITGAV; E(i) NBPF9, (ii) NBPF14 and (iii) NBPF15; and F(i) GUCA1C, (ii) GJA1, (iii) PRKAA2, (iv) ACTG1 and (v) CCT5 in HRTPT and HREC24T cells. ***, ** and * indicate significant differences in gene expression level in HRTPT and HREC24T cells at *P*‐values of ≤0.001, ≤0.01 and ≤0.05, respectively

The expression of many of the genes identified in the above groupings and pathways was further examined using qPCR to validate the array and determine expression levels of the genes between the HRTPT cells and the HREC24T cells (Figure 3). This included the gene expression analysis of the majority of tubulins (Figure 3Bi‐v), annexins (Figure 3Ai‐iii), integrins (Figure 3Di‐iv) and RHO‐GTPases (Figure 3Ci‐x) identified by David and Reactome analyses. The tubulin genes were identified frequently, both as a gene group when analysed by David and as a major participant in the first 8 of the 9 pathways identified using Reactome (Figure 3Bi‐v, Figure 2A and 2E). RHO‐GTPases were also identified as a gene group and in one of the pathways identified by Reactome (Figure 2E). The annexins and integrins were identified as a gene group, but not associated with a specific pathway. However, they would likely participate in the organization and interaction of the apical membrane as identified by Ingenuity Pathway Analysis (Figure 1D, Figure 3Ai‐iii,Di‐iv). The expression of several other genes identified in the first 8 Reactome pathways, NBPF9, NBPF14, NBPF15, GUCA1C, GJA1, ACTG1, PRKAA2 and CCT5, was also assessed for their expression between the two cell lines (Figure 3Ei‐iii,Fi‐v). Of these genes, connexin 43 (GJA1) showed substantial basal expression in the HRTPT cells, but was much lower when compared to the HREC24T cells (Figure 3Fii). Overall, the genes identified by David and Reactome were consistent with the top canonical pathways and top molecular and cellular functions identified using Ingenuity software (Figure 2B,D).

### 3.3 FGFR, FGF expression and treatment with SU5402 in HRTPT cells

**FIGURE 4:**
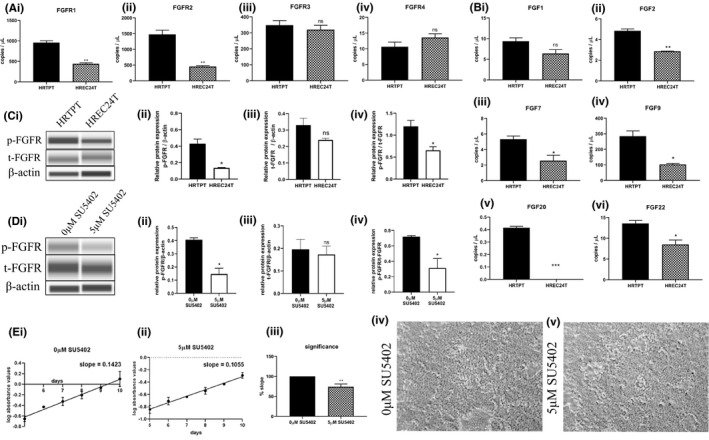
Expression of FGFRs, FGFs and treatment with FGFR inhibitor SU5402 in the HRTPT and HREC24T cells. qPCR analysis of A (i) FGFR1, (ii) FGFR2, (iii) FGFR3 and (iv) FGFR4; B (i) FGF1, (ii) FGF2, (iii) FGF7, (iv) FGF9, (v) FGF20 and (vi) FGF22. (C) Western blot analysis of p‐FGFR, t‐FGFR and β‐actin in (i) HRTPT and HREC24T cells and relative protein levels for (ii) p‐FGFR/β‐actin, (iii) t‐FGFR/ β‐actin and (iv) p‐FGFR/t‐FGFR. (D) Western blot analysis of p‐FGFR, t‐FGFR and β‐actin in HRTPT treated with (i) 0µM and 5µM SU5402 and relative protein levels of (ii) p‐FGFR/β‐actin, (iii) t‐FGFR/ β‐actin and (iv) p‐FGFR/t‐FGFR. MTT growth curve assay of HRTPT cells treated with E (i) 0µM, (ii) 5µM SU5402, (iii) fold change in growth curve in HRTPT cells treated with 5µM SU5402 compared with that of 0µM SU5402 in per cent; light‐level microscopy of HRTPT cells treated with (iv) 0µM or (v) 5µM SU5402. ***, ** and * indicate significant differences in gene expression or growth curve level in HRTPT and HREC24T cells at *P*‐values of ≤0.001, ≤0.01 and ≤0.05 respectively. ns indicates non‐significant

The HRTPT expression was further analysed using only the upregulated 1117 probes (653 unique genes) from global gene expression analysis (Figure 1C, Table S3). The genes identified from this analysis coupled with both David and Reactome identified FGFR2 as an important upregulated molecule. The analysis was expanded to include the expression of all 4 FGF receptor family members (Figure 4Ai‐iv). The FGFR1 and FGFR2 were demonstrated to have a substantial basal level of expression in the HRTPT cells with an increase in expression of approximately 50% and 75%, respectively, compared with the HREC24T cells (Figure 4Ai‐ii). The basal expression of FGFR3 was 20 to 30% lower, and FGFR4 expression was much lower than that of FGFR1 and FGFR2. The levels of mRNA expression of FGFR3 and FGFR4 showed no difference between the two cell lines (Figure 4Aiii,iv). The expression of the 1,2, 7, 9, 20 and 22 isoforms of fibroblast growth factor (FGF) was also determined for the HRTPT and HREC24T cell lines (Figure 4Bi‐vi). The FGF9 was the only FGF that showed substantial basal expression and was increased 70% in the HRTPT cells (Figure 4Biv). The other 5 FGF had basal expression levels over 200% lower than those of FGF9, although all were increased in expression compared with HREC24T cells. The HRTPT cells were shown to have elevated protein levels of the phosphorylated form of FGFR when compared to the HREC24T cell line, with both cell lines having comparable amounts of total unphosphorylated FGFR (Figure 4Ci‐iv). Treatment of the HRTPT cells with the FGFR inhibitor, SU5402, resulted in a reduction of phosphorylated form of FGFR with comparable amounts of total unphosphorylated FGFR (Figure 4Di‐iv). The SU5402 inhibitor was also used to determine its effect on the growth of the HRTPT cells (Figure 4Ei,ii). It was demonstrated that approximately 25% reduction in doubling time occurred when the HRTPT cells were treated with 5 μM of the SU5402 FGFR inhibitor (Figure 4Eiii). Treatment of the HRTPT cells with the FGFR inhibitor elicited no change in light microscopic cell morphology (Figure 4Eiv,v).

### 3.4 Analysis of HRTPT downregulated genes

**FIGURE 5:**
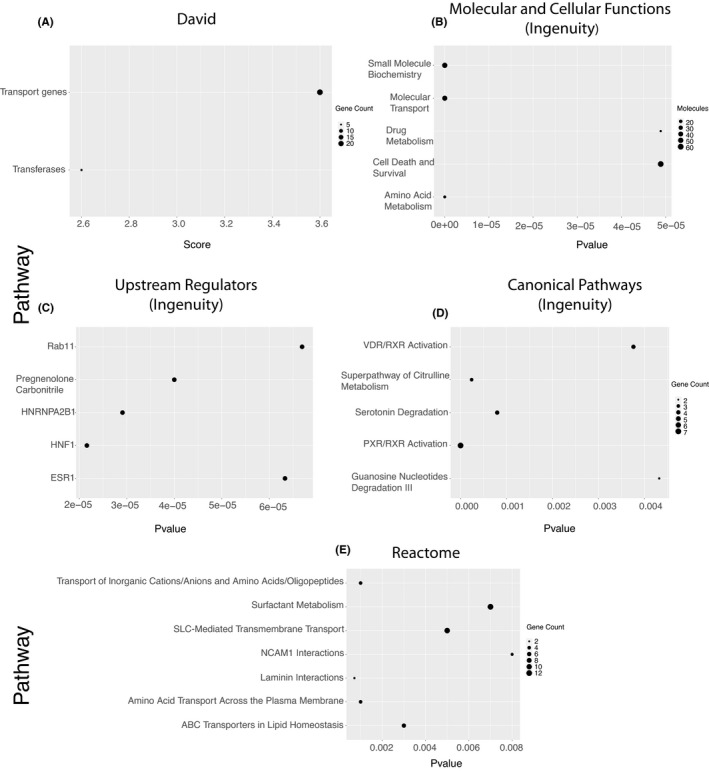
Bubble plot of pathways significantly associated with upregulated HREC24T genes obtained from (A) David analysis, (B) top molecular and cellular function (Ingenuity), (C) top upstream regulators (Ingenuity), (D) top canonical pathway analysis (Ingenuity) and (E) Reactome pathway analysis

Similar to the above section, the HRTPT‐downregulated 220 genes (366 probes) were further examined to find the functional associations (Figure 1C, Table S3). The pathway analysis by Reactome, David and Ingenuity software all identified groupings and pathways reflecting the large number of solute carrier family genes identified in the HREC24T cells when compared to the HRTPT cells (Figure 5A‐E). Seven of the downregulated genes were selected to validate their mRNA expression levels in HRTPT and HREC24T cells (Figure S1). Six out of seven genes had significantly lower levels of gene expression in HRTPT compared with HREC24T (Figure S1A‐G); however, ESR1 showed basal‐level detection in both cell lines (Figure S1H). A total of 17 solute carrier family members were identified as specific to the HREC24T cells (Table S4). All three analysis programmes identified transport as a feature of the HREC24T cells. Related to these transport processes were interactions between the cell surface and the extracellular environment as noted by the identification of specific transferases and laminin and NCAM1 interactions (Figure 5E). In addition, Ingenuity Pathway Analysis identified both PXR/RXR and VDR/RXR activation as top canonical pathways, both of which are nuclear receptors capable of a wide range of interactions with promoters of gene transcription (Figure 5D, Figure S2). This classification of global gene expression analysis between the two cell lines shows clear separation of distinct identities based on the presence or absence of CD133.

### 3.5 Association between HRTPT cells and infant kidney expressing CD133 progenitor cells

The gene set identified for the HRTPT cells as described above was compared with a publicly available database (GSE90628) that compared the expression of CD133‐ and CD133+ cells isolated from the human infant kidney biopsies.^27^ The infant kidney‐derived cells were isolated, and sorted by FACS, and the CD133+ and CD133‐ fractions were placed into cell culture for 2 passages before microarray analysis. The microarray analysis identified 3,948 genes with *P* < 0.05 that were uniquely expressed by the CD133+ cell fraction extracted from data sets for GSE90628 (Table S5). To identify the functional association, Reactome and Ingenuity profiles were compared between the 3948 CD133+ infant kidney cell gene set (Table S6B‐E) and the 873 HRTPT cell gene set (Figure 2B‐E). Reactome analysis demonstrated that there were no common pathways between the infant kidney cells expressing CD133 and the HRTPT cells (Figure 2E and Table S6B). Ingenuity Pathway Analysis showed no similarity for the top canonical pathways (Figure 2D and Table S6C), but did show high symmetry within the molecular and cellular function, which included cellular movement, cellular development, and cell death and survival being common between both sets of genes (Figure 2B and Table S6D). Ingenuity Pathway Analysis identified dexamethasone, ESR1 and beta‐oestradiol as upstream regulations, as well as TNF and TGFB1 (Table S6E). An analysis of gene groups employing David software could not be performed due to the large size of the set of the infant kidney genes (Table S6A).

An analysis to determine the common genes between the 873 HRTPT gene set and the 3,948 CD133+ infant gene set identified 332 co‐expressed genes (Figure S3). To validate the functional association with previous identified pathways between two sets of genes, we identify the pathways associated with 332 co‐expressed gene set using David, Reactome and Ingenuity software (Table S7A‐E). When compared to previously identified pathways (ie Figure 2 and Table S6), Ingenuity software provided the strongest comparison among the pathways with agreement in the area of molecular and cellular function, identifying cellular movement, cellular development, and cell death and survival as top 5 elements (Table S7D). In addition, both cellular growth and proliferation, and cell‐cell signalling were identified in both 873 HRTPT gene sets and 332 co‐expressed gene sets (Figure 2B and Table S7D). A further analysis employing Ingenuity demonstrated an interesting result in the area of upstream regulators, showing that both the 3,948 CD133+ infant kidney data set and the 332 co‐expressed data set did show identity for beta‐oestradiol, TGFB1 and dexamethasone (Tables S6E and S7E). There was no similarity of pathways identified by Reactome and the top canonical pathways determined by Ingenuity software among the 332 co‐expressed genes and the other 2 gene sets (Figure 2D and 2E, and Tables S7B and S7C, Tables S6B and S6C. There was one gene group identified by David that had identity with the 873 HRTPT gene set, identifying integrins A1, A2, B3 and B8 (Figure 2A and Table S7A). The expression of FGF9 was identified in the 332 common gene set between the CD133+ infant kidney cells and the gene set identified by the comparison between the HRTPT cells and the HREC24T cells. As noted previously, analysis of FGF and FGFR expression in the HRTPT cells was confirmed by qRT‐PCR that demonstrated FGF9, FGFR1 and FGFR2 had substantial basal expression and high protein levels of p‐FGFR in HRTPT compared with HREC24T cells (Figure 4A‐C). The 332 gene set from the above study did not identify either the FGFR1 or FGFR2 receptors as co‐expressed between the two data sets. However, only the FGFR1 receptor was present in the infant kidney CD133+ gene set and in the HRTPT array, indicating the FGFR1 receptor remains an active component that can be added to the 332 co‐expressed gene set. If one assumes that the CD133+ infant kidney cells possessed a population of progenitor cells, then the 332 gene set should contain the progenitor genes from both the original 873 HRTPT gene set and the 3,948 CD133+ gene set from the infant kidney.

### 3.6 Association between diverse urine‐derived progenitor cells, infant kidney CD133+ Cells and HRTPT cells

Urine from healthy human donors has been reported to be a readily available, non‐invasive source for the isolation and culture of human renal progenitor cells.^28^ The study isolated 9 putative renal progenitor cell cultures from the urine of 9 independent volunteers. The global gene expression pattern was determined for these cell lines as a group compared with that of a biopsy‐derived, commercial culture of human proximal tubule cells. This analysis demonstrated that 829 genes were co‐expressed by the udRPCs and the biopsy‐derived hREPCs (Figure S4; udRPCs vs hREPCs).

Subsequently, these 829 gene**s** were compared with the 873 gene set from HRTPT cells and the 3,948 CD133+ infant kidney gene set (Figure S4). The goal of this analysis was to determine whether the number of genes in the 873 HRTPT gene set could be reduced while preserving the CD133+ progenitor genotype. The results of this analysis showed that the 446 common gene set expressed by the infant kidney and the urine‐derived progenitor cells did not significantly express CD133 (PROM1), but did express the paralog PROM2 gene, which as expected showed no co‐expression of CD133(PROM1) or PROM2 for comparison analysis of 3 gene set table (Table S8). However, the previous analysis showed that CD133 (PROM1) was co‐expressed by both the HRTPT and the CD133+ infant kidney gene sets (Figure S3, shown in the list of genes).

**FIGURE 6:**
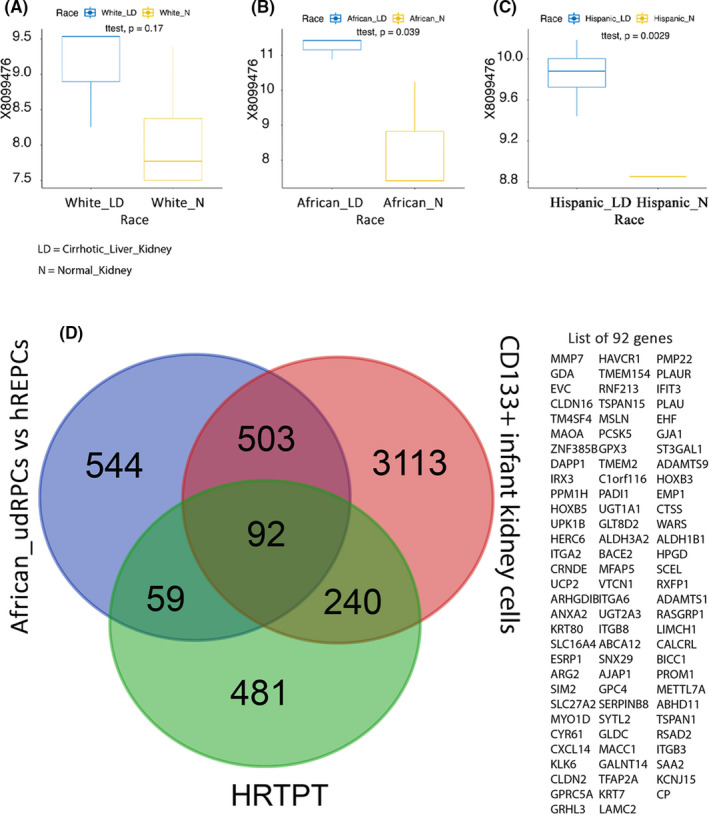
Boxplot for PROM1 (CD133) analysed based on race. Gene expression of PROM1 (probe id: X8099476) gene demonstrated by box plot between cirrhotic liver kidney (LD: blue colour) and normal kidney (N: yellow colour) patients for three different races: (A) White (number of LD =2, N = 4), (B) African American (number of LD =4, N = 2) and (C) Hispanic (number of LD =2, N = 2). The level of difference is measured by t test, and p‐value is provided for each comparison. (D) Venn diagram of African_udRPCs vs hREPCs, CD133+ infant kidney and HRTPT gene sets and the list of 92 common genes between three gene sets

Based on this laboratory's previous work, the biopsy‐derived human proximal tubule cells would be expected to possess a population of cells expressing CD133.^12^, ^13^ To explore the lack of CD133 expression in the udRPCs, when compared to the infant kidney and HRTPT cells, the data were reanalysed based on the race of the volunteers providing the urine samples. Of the nine volunteers providing urine samples, 2 were African American, 4 Hispanic, 2 White and 1 Asian. The analysis of the data using only the White volunteers showed no expression of CD133 with the infant kidney or the HRTPT gene sets similar to analysis found with the 3 gene set comparison. In contrast, reanalysis of the data using the samples from African American volunteers showed expression of CD133 in all three data sets and defined 92 common genes within the 3 data sets (Figure 6). The study by Rahman and co‐workers (2020) did provide a heat map of the expression of CD106, CD133, CD24 and PODXL for each of the nine urine‐derived renal progenitor cell lines. The expression of CD133 was high in all three cell cultures isolated from Black volunteers, but there were samples for Caucasian volunteers that were substantial low while others had low expression of CD133 (Figure 6A‐C). While this may explain the racial differences found for CD133 expression, the findings also leave open the possibility of differences in the role of CD133 renal progenitors based on race.

The analysis of the 92 gene set by David, Reactome and Ingenuity pathways showed similarity with the other CD133 expressing data sets presented in Figure 2, Figures S6 and S7 (Table S9A and E). Overall, as the number of genes in the data set narrowed, ESR1 and beta‐oestradiol were identified as upstream regulators and cellular movement and cell‐cell signalling as top canonical pathways (Table S9D,E). In most data sets, the integrins, cell junctions, cell surface interactions and receptors were identified frequently in gene groupings and Reactome pathways (Table S9A,B). The ESR1 was validated for expression in the HRTPT cell line and found to be near the background for detection (Figure S1H).

### 3.7 Single‐cell analysis of HRTPT cells

**FIGURE 7:**
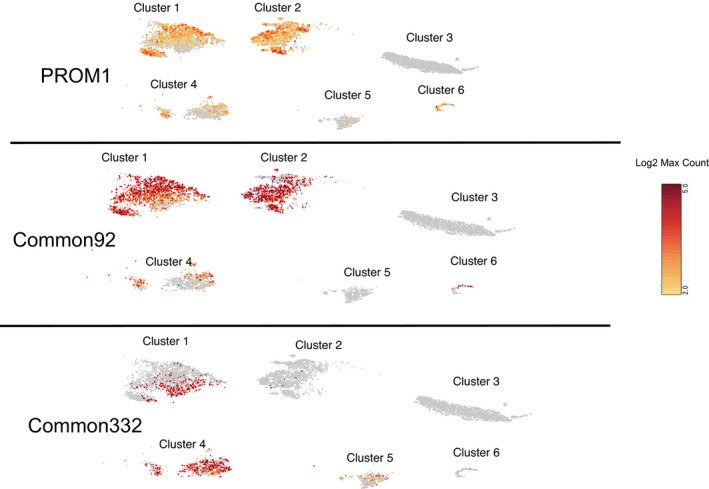
Single‐cell gene enrichment: A comparative enrichment of three gene set signatures defined as (A) PROM1, (B) common 92 and (C) common 332 in six different cluster of single‐cell data. The colour code bar represents the scale of log2 max count of gene set

The laboratory employed scRNA‐seq technology to find the clusters of similar cells by examining the higher expression of single gene CD133 (Figure 7A) and the set of genes named as common 92 (Figure 7B) and common 332 (Figure 7C). The entire cell population was divided into six clusters and examined the log2 feature maximum count to find the similarities between PROM1 and gene sets at cellular level to better understand the function of an individual cell in the context of its microenvironment. The outcome shows that the PROM1 and common 92 gene set are very close in terms of cellular pattern of log2 expression average of genes in that cell (Figure 7A and 7B) and partially enriched in the cluster 1, cluster 2, cluster 4 and cluster 6 only (Figure 7C).

## 4 DISCUSSION

The majority of laboratories studying renal tubule progenitor cells identify this population based on the co‐expression of CD133 and CD24 within the kidney and in renal‐derived cell cultures.^7^, ^8^, ^13^, ^28^, ^29^ This laboratory was able to sort the HRTPT cells isolated from a renal epithelial cell line having a cell population co‐expressing CD133 and CD24, and HREC24T cells expressing only CD24. As published previously,^12^, ^13^ only the HRTPT cell line displayed progenitor properties, whereas the HREC24T cell line displayed no features associated with renal progenitor cells. Following characterization, this laboratory performed a global gene expression analysis on the two cells lines, which identified 873 genes that were unique for the HRTPT cells. The analysis of this gene set by David, Reactome, Ingenuity's top 5 conical pathways, and molecular and cellular functions analysis software identified groups and pathways related to the cytoskeletal organization and its effect on the apical cell membrane, especially the role of microtubules in cytoskeletal dynamics.^30^, ^31^, ^32^, ^33^, ^34^ A disruption of microtubule dynamics has been shown to impact kidney repair following ischaemia/reperfusion.^31^ The only gene grouping that did not appear to be linked to microtubules and cytoskeleton was the neuroblastoma breakpoint genes that have not been highly studied, but do have a role in kidney development.^35^ Overall, the analysis of global gene expression allowed the definition of genes and pathways associated with a CD133 and CD24 co‐expressing cell line, known to have features associated with renal progenitor cells.

The association of renal CD133 with the progenitor phenotype appears to be largely accepted for renal tubular progenitor cells.^5^, ^6^, ^7^, ^8^, ^9^, ^10^, ^28^, ^29^ Therefore, with the goal to identify the genes essential for renal regeneration vs genes not involved in regeneration in cells expressing CD133, 873 gene sets for HRTPT were compared with another publically available global gene expression database. The database was derived from infant renal tissue sorted to obtain cell expression of CD133 only that identified 3,948 genes specific to CD133+ cell fraction. The analysis of 3948 genes independently using Reactome and Ingenuity showed no similarity to the pathway analysis or canonical pathways, and the top upstream regulators were determined for the HRTPT cells independently. In contrast, there was good agreement for molecular and cellular functions between the gene sets from the infant kidney CD133+ cells and the HRTPT cells.

The 3,948 CD133+ infant kidney cell gene set compared with the 873 HRTPT cell gene set together demonstrated that 322 genes were common between them, including CD133. Analysis for this combination gene groups showed some similarity to 873 gene set of HRTPT, with integrin and cell membrane organization and function being common features using DAVID and laminin interactions as common using Reactome. Top conical pathways and upstream regulators showed no similarity; in contrast, molecular and cellular functions determined by Ingenuity were similar between the two data sets. The 322 gene set and the 3,948 set were then compared with an additional publically available database where renal progenitor cell culture was isolated from human urine.^28^ This comparison identified 92 genes that were common in all three data sets, including CD133. An analysis by Reactome and David identified the organization and interactions of the apical cell member. Pathway analysis by Ingenuity identified Wnt/β‐catenin signalling, RXR/RXR activation and Sertoli cell functional signalling. Molecular and cellular functions identified cell‐to‐cell signalling and interactions and cellular movement. Dexamethasone, β‐oestradiol and ESR1 were identified as top upstream regulators. The identification of β‐oestradiol and the estrogen receptor (ESR1) was of interest since acute kidney injury is less common and less severe in women than in men.^36^, ^37^ The β‐oestradiol has also been shown to be protective in animal models of acute kidney injury.^38^, ^39^ However, in this study, ESR1 had very low detection levels in both cell lines and the RXR receptor expression was lower in HRTPT cell lines.

During the validation of the array, the FGFR1, FGFR2 and FGF9 were shown to have substantial expression in the HRTPT cells. The FGFR was shown to be phosphorylated, with reduction in the phosphorylated form of FGFR and a modest reduction in growth rate of the cells when treated with the SU5402 FGFR inhibitor in the HRTPT cells. Since there is no FGF in the serum‐free media on which the cells grow, it is possible that FGF serves as an autocrine loop for cell growth and differentiation. Several studies have implicated FGF role in renal progenitor biology, presenting further evidence of the potential importance of FGF and FGFR signalling in renal progenitor cells. FGF9 and 20 have been reported to maintain ‘stemness’ during embryonic nephron development.^40^ A recent review has detailed the possible role of FGF in the management of acute renal injury following ischaemia/reperfusion (IRI).^41^ Evidence suggests that alterations in the expression of endogenous FGF are associated with IRI and may alter the repair process and discovered that exogenous FGF ligand might also be protective against IRI. Mouse model studies showed that FGFR2 knockout aggravated acute tubular dysfunction.^42^ Another group isolated and cultured renal progenitor cells from both urine and amniotic fluid and proposed that renal progenitor cells maintain self‐renewal by active FGF signalling leading to the phosphorylation of TGF‐β‐SMAD2/3.^28^, ^43^

The RPTEC/TERT cell line, from which the HREC24T and HRTPT cell lines were isolated, was reported to possess many of the differentiated features of proximal tubule cells.^14^ Renal progenitor cells have also been reported to have differentiated features associated with the proximal and distal tubules, which possibly means that proximal tubule cell culture is a mixture of CD133 expressing and non‐expressing cells.^10^, ^44^ This laboratory's examination of primary human proximal tubule (HPT) cells isolated from the kidney cortex has been demonstrated to have differentiated features of the proximal tubule and also be comprised of a mixture of CD133+CD24+ and CD133‐CD24+ cells, almost in equal proportion.^13^, ^15^ These two evidences suggest that renal progenitor cultures and proximal tubule cell cultures are very similar, and possibly identical, with the major difference being the vocabulary of the laboratory. The fact that the two cell types could be sorted and placed into cell culture provides evidence that the two cell types do not rely on one another for cell homeostasis. The global gene expression identified 187 genes that were specific to the HREC24T cell line. The two most striking features of the HREC24T cells were that the pathway analysis and gene groupings reflected a small number of specific genes and the large number of transport genes (17/187) when compared to the HRTPT cells. The small number of genes specific to the HREC24T cells indirectly suggests that they also retain the differentiated features of the proximal tubule. This suggestion is strengthened by the prior study, which showed the cells have very similar morphology and display vectorial active transport.^12^, ^13^

In the human kidney, renal tubule progenitor cells expressing CD133 and CD24 are scattered as single cells within the nephron. The single progenitor cells have not been observed to replicate and form a focus of cells that could provide serial sections for immunohistochemical determination of the expression of multiple proteins. This poses the question of whether renal progenitor cells represent one unique cell type or whether there are multiple subtypes of renal progenitors within the CD133 and CD24 co‐expressing population. To gain initial insight into this question, the HRTPT cells were subjected to single‐cell RNA sequencing to determine whether there were unique clusters of genes that are associated with CD133 within the HRTPT cell line. The results demonstrated that there were unique clusters of cells that were associated with CD133 and the common 92 set of genes. This suggests that there may be unique subsets of renal progenitor cells within the HRTPT cell line or that not all subsets of CD133 expressing cells are progenitors.

There were two preliminary findings in the study that will require further investigation. The first is the relationship between PROM1 (CD133) and PROM2 expression, the major question being whether PROM2 can substitute wholly or partially for the function of PROM1 as the comparison of two publically available dataset representing renal progenitors showed expression of PROM2 but no co‐expression of CD133 when compared to the HRTPT data set. PROM2 has seen only limited study. The tissue distribution of PROM2 is very similar to PROM1, being highly expressed in the adult kidney and other epithelial tissues.^45^ PROM2 was shown to be co‐localized with PROM1 in its association with plasma membrane protrusions. Several other studies support at least a partial role for PROM2 in its ability to substitute for PROM1 in some situations.^46^, ^47^, ^48^ In the PROM1 KO mouse, some stem cells can still progress into transit‐amplifying cells, possibly through compensation mechanisms involving PROM2. The upregulation of PROM2 has also been observed in neural stem cells from the adult murine hippocampus in PROM1‐deficient animals. Thus, the role of prominin paralogues on cell fate and membrane protrusions remains an open area of investigation. The second finding suggested there might be an effect of race on the expression of PROM1 and PROM2 for renal progenitor cells. However, this observation was based on a very small set of samples and would require further investigation on a larger set of data with clinical information.

## 5 CONCLUSION

Unique gene sets specific to HRTPT cell line, having co‐expression of CD133 and CD24 and that displayed progenitor‐like characteristics, and HRECT24 cell line, expressing only CD24 that did not display progenitor characteristics, were identified providing the differences between the two cell population at the gene level. Moreover, the sequential comparisons of the HRTPT cells with two additional publically available databases narrowed the number of common genes to 92 between three comparisons. By utilization of the publicly available database and the empirical analysis of gene groups and pathways by David, Reactome and Ingenuity, this study disclosed several unique features of the HRTPT cells and association with renal progenitors in other studies. The findings and results presented on gene expression of HRTPT cell line are novel and essential to gain knowledge on mechanism of repair and regeneration in kidney.

## CONFLICTS OF INTEREST

The authors declare that they have no conflicts of interest.

## AUTHOR’S CONTRIBUTIONS

Swojani Shrestha: Collection and/or assembly of data; data analysis and interpretation; manuscript writing; final approval of the manuscript. Sonalika Singhal: Data analysis and interpretation; manuscript writing; final approval of the manuscript. Matthew Kalonick: Collection and/or assembly of data; data analysis and interpretation. Rachel Guyer: Collection and/or assembly of data; data analysis and interpretation. Alexis Volkert: Collection and/or assembly of data; data analysis and interpretation. Seema Somji: Final approval of the manuscript. Scott Garrett: Conception and design; manuscript writing; final approval of the manuscript. Donald A Sens: Conception and design; financial support; manuscript writing; final approval of the manuscript. Sandeep K. Singhal: Conception and design; collection and/or assembly of data; data analysis and interpretation; manuscript writing; final approval of the manuscript.

## Supporting information

Figure S1. Expression of upregulated gene groups (David) HREC24T cells. qPCR analysis of A. RXRA; B. VDR; C. NR1D1; D. IGFBP3; E. SLC7A2; F. SLC9A1; G. SLC47A1 and H. ESR1 in HRTPT and HREC24T cells. ***; **; * indicates significant differences in gene expression level in HRTPT and HREC24T cells at *P*‐value of ≤0.001; ≤0.01; ≤0.05 respectively

Figure S2. Ingenuity pathway analysis (A) PXR/RXR (B) VDR/RXR as top canonical pathways associated with significant genes. Red color represents the intersection of genes with pathways

Figure S3. Venn diagram of CD133+ Infant Kidney vs HRTPT Gene Sets and the list of 332 common genes between two gene sets

Figure S4. Venn diagram of udRPCs vs hREPCs, CD133+ infant kidney & HRTPT gene sets

Figure S5. Samples and features distribution before and after normalization of HREC24T and HRTPT gene expression data

Table S1. List of primers used for ddPCR

Table S2. List of Antibodies, source from where they were purchased, catalog number and dilution used for western blot

Table S3. List of 1483 probes (873 unique genes) that were differently expressed between HRTPT and HREC24T cell line (*P*‐value<0.05)

Table S4. A list of HREC24T transport Genes

Table S5. A list of 3,948 genes with *P*‐value <0.05 and other statistical measurements that were differently expressed between CD133+ versus CD133‐ cell fraction from infant kidney biopsies

Table S6. A list of pathways associated with differently expressed genes between CD133+ versus CD133‐ Infant Kidney

Table S7. A list of pathways associated with differently expressed genes between HRTPT Gene Set versus CD133+ Infant Kidney Gene Set

Table S8. T‐test outcome of PROM1 and PROM2 across HRTPT, CD133+ infant kidney & udRPCs vs hREPCs datasets

Table S9. A list of pathways associated with differently expressed genes between HRTPT Gene Set vs CD133+ Infant Kidney Gene Set vs African_ udRPCs vs hREPCs Gene Set
