# Supplementary figures and images for: Role of HRTPT in kidney proximal epithelial cell regeneration: Integrative differential expression and pathway analyses using microarray and scRNA‐seq
Source: J Cell Mol Med. 2021 Oct 9;25(22):10466–79. doi: 10.1111/jcmm.16976 (PMC8581341; doi:10.1111/jcmm.16976)

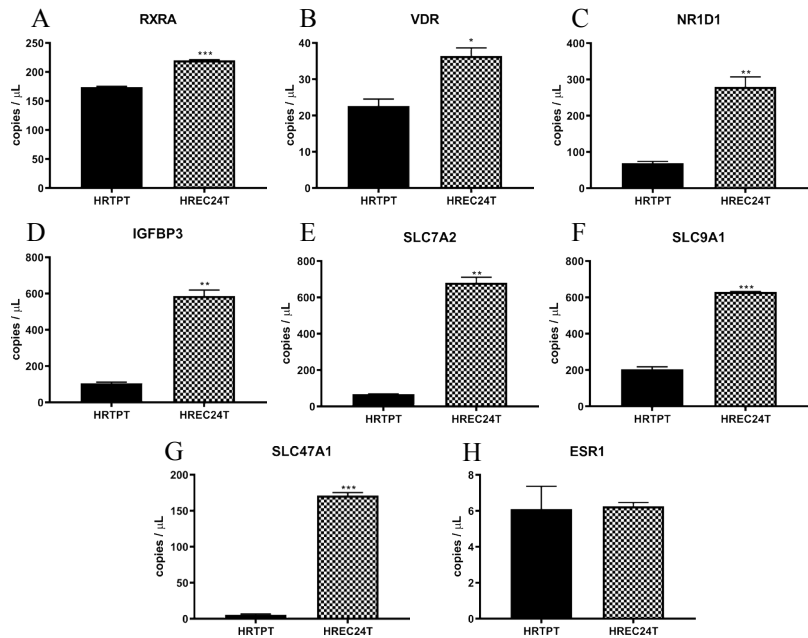

Figure S1.

Supplement: Supplementary file 1 — Figure S1. Expression of upregulated gene groups (David) HREC24T cells. qPCR analysis of A. RXRA; B. VDR; C. NR1D1; D. IGFBP3; E. SLC7A2; F. SLC9A1; G. SLC47A1 and H. ESR1 in HRTPT and HREC24T cells. ***; **; * indicates significant differences in gene expression level in HRTPT and HREC24T cells at P‐value of ≤0.001; ≤0.01; ≤0.05 respectively [file JCMM-25-10466-s003.pdf]

B

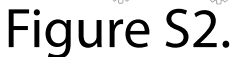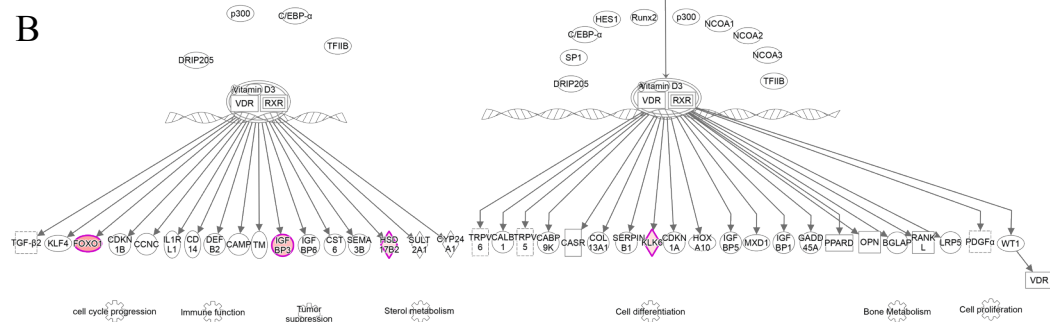

Supplement: Supplementary file 2 — Figure S2. Ingenuity pathway analysis (A) PXR/RXR (B) VDR/RXR as top canonical pathways associated with significant genes. Red color represents the intersection of genes with pathways [file JCMM-25-10466-s007.pdf]

udRPCs vs hREPCs

CD133<sup>+</sup> infant kidney cells

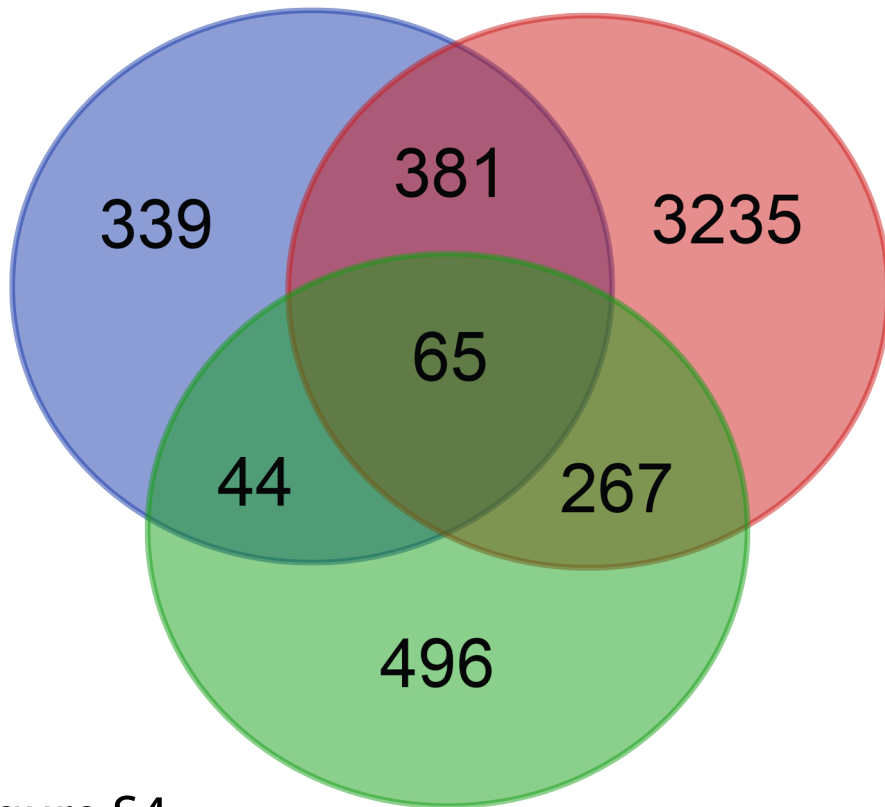

Figure S4.

HRTPT

Supplement: Supplementary file 4 — Figure S4. Venn diagram of udRPCs vs hREPCs, CD133+ infant kidney & HRTPT gene sets [file JCMM-25-10466-s010.pdf]
