# Supplementary material for: Role of HRTPT in kidney proximal epithelial cell regeneration: Integrative differential expression and pathway analyses using microarray and scRNA‐seq
Source: J Cell Mol Med. 2021 Oct 9;25(22):10466–79. doi: 10.1111/jcmm.16976 (PMC8581341; doi:10.1111/jcmm.16976)
Supplement: Supplementary file 3 — Figure S3. Venn diagram of CD133+ Infant Kidney vs HRTPT Gene Sets and the list of 332 common genes between two gene sets [file JCMM-25-10466-s012.pdf]

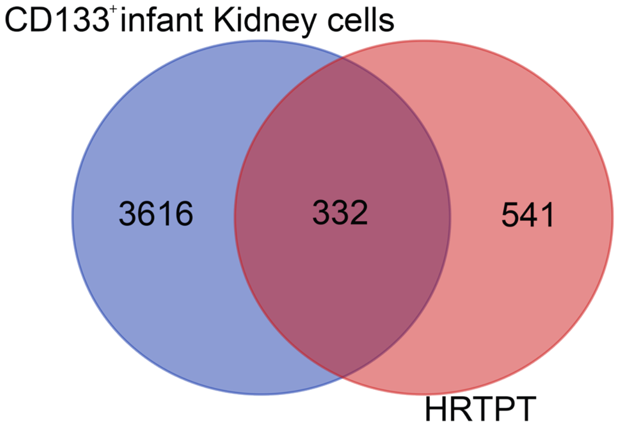

List of 332 genes

FSTL1	THBS1	KIF21A	UGT1A1	LHFP	HPGD
ESYT1	SLC16A4	LIN7A	MNS1	GALNT14	PLEKHH2
LRRC6	NME5	GPRC5A	HSD11B2	WFDC2	SCEL
FAM134B	PDE10A	GBP2	GLT8D2	TFAP2A	RXFP1
MMP7	MECOM	TMED5	PALLD	RAB11FIP1	TBX15
RDH10	ESRP1	CDH6	ZNF702P	KRT7	CCL2
KLHL14	ARG2	GRHL3	ALDH3A2	TFPI	SLC7A8
FAT4	LRRIQ1	HAVCR1	MEIS2	PKIA	PDE1A
GDA	SIM2	ARRDC4	SERPING1	HOXD3	ZMAT3
SLC15A2	SBSPON	TXNIP	TPCN1	LAMC2	DNAJC6
EVC	FAXDC2	RAP2B	QPRT	CD47	STX3
ACSM3	ANXA2P1	TMEM154	SLC17A3	SEMA4A	TMEM51-AS1
ANXA3	SLC27A2	RNF213	PIK3AP1	NUAK2	MOXD1
HNF1A-AS1	SKIL	SLC7A2	BACE2	KMO	ADAMTS1
CLDN16	IGFBP3	EDIL3	MFAP5	CLDN1	RASGRP1
DUSP4	XKR4	TSPAN15	VTCN1	PMP22	PPM1B
TM4SF4	ROR1	MIR181A2HG	PKHD1	COMMD9	SLC7A5
ZNF385B	MYO1D	PSAP	NKTR	B4GALT5	AK4
MAOA	BMP2K	SERPINE2	PERP	ME1	SLC15A1
ST8SIA4	ZNF486	LGMN	LACTB2	PLAUR	CHST9
F2RL1	CYR61	SPNS2	PWAR5	MIR3189	CLINT1
BCL2	AKT3	ORMDL2	CCDC113	IFIT3	FGF9
DAPP1	HSBP1	IFI44L	CSRP1	MOB3B	IFIT5
GNG12	LINC00472	DCTPP1	CTSB	PGK1	LIMCH1
LOC100506498	ARSJ	CLU	NEFL	WLS	TMEM173
RBPMS	FLVCR2	CXCL12	ITGA6	MYO1B	CALCRL
ATP10A	SAT1	SLC43A1	SGK2	SLC17A1	ADK
IRX3	CLDN10-AS1	SLC20A1	NET1	PLAU	PRR5L
NMNAT2	ST6GALNAC1	ARAP2	TOX	EHF	SLC9A1
PPM1H	UGCG	WDR72	UGT2A3	GOLM1	BICC1
HOXB5	EIF6	SNX30	ITGB8	NRP1	ANPEP
UPK1B	CXCL14	CAV1	PDE3A	GJA1	PROM1
NTN4	HSPB8	SHISA9	SLC7A11	UCHL1	KRTAP2-3
HERC6	KLK6	PAQR5	TIMP1	ST3GAL1	METTL7A
FGF12	HCP5	CXCL1	ABCA12	ATP13A3	NR1H4
SULF2	SFPQ	CAP2	AGA	ADAMTS9	ABHD11
NR2F2-AS1	SCARNA9	MIR205HG	ZC2HC1A	HOXB3	TSPAN1
ITGA2	DGKH	GLRB	SNX29	DSG2	RSAD2
BAIAP2L2	ANKRD10	MSLN	ZNF90	FAM133CP	CCDC146
MIR21	MYO6	DDX58	HABP2	IFIT2	ZNF667-AS1
LINC01111	CNKSR3	ANKIB1	AJAP1	PIK3R3	RHOBTB3
FOXO1	CKB	PCSK5	MOCS1	FAM169A	SLC34A2
STMN1	ABO	GPX3	HERC1	EMP1	SYT14
CRNDE	HMCN1	HPS5	GPC4	C14orf105	ITGB3
CD109	CLDN2	LHFPL3-AS2	SERPINB8	RAB27B	NLGN4Y
UCP2	ITGA1	TMEM2	ZNF528	CTSS	SAA2
ARHGDIB	FOLR1	PEG10	SASH1	GAS2L3	PMEPA1
KCNH1	ABCB1	GPC6	DKK3	WARS	C1orf186
ANXA2	CNR1	PTH1R	MYLK	RGL3	TMSB4X
PAM	FAR2	C1orf116	LOC1019270[illegible]	DDX60	KCNJ15
KRT80	KCNQ1OT1	ADAMTSL3	EMP3	B4GALT6	GCNT1
LRRC49	FABP3	TXNDC17	SYTL2	STK32A	CP
ERP27	ANXA6	FJX1	PMAIP1	TBXAS1	
GLIS3	SLC25A13	DPYSL3	GLDC	ALDH1B1	
SP140	TMEM14A	GDF15	MACC1	FAM196B	
ARHGAP29	SAMD9L	PADI1	SPA17	RND3	

Figure S3.
