# Supplementary material for: Role of HRTPT in kidney proximal epithelial cell regeneration: Integrative differential expression and pathway analyses using microarray and scRNA‐seq
Source: J Cell Mol Med. 2021 Oct 9;25(22):10466–79. doi: 10.1111/jcmm.16976 (PMC8581341; doi:10.1111/jcmm.16976)
Supplement: Supplementary file 5 — Figure S5. Samples and features distribution before and after normalization of HREC24T and HRTPT gene expression data [file JCMM-25-10466-s013.pdf]

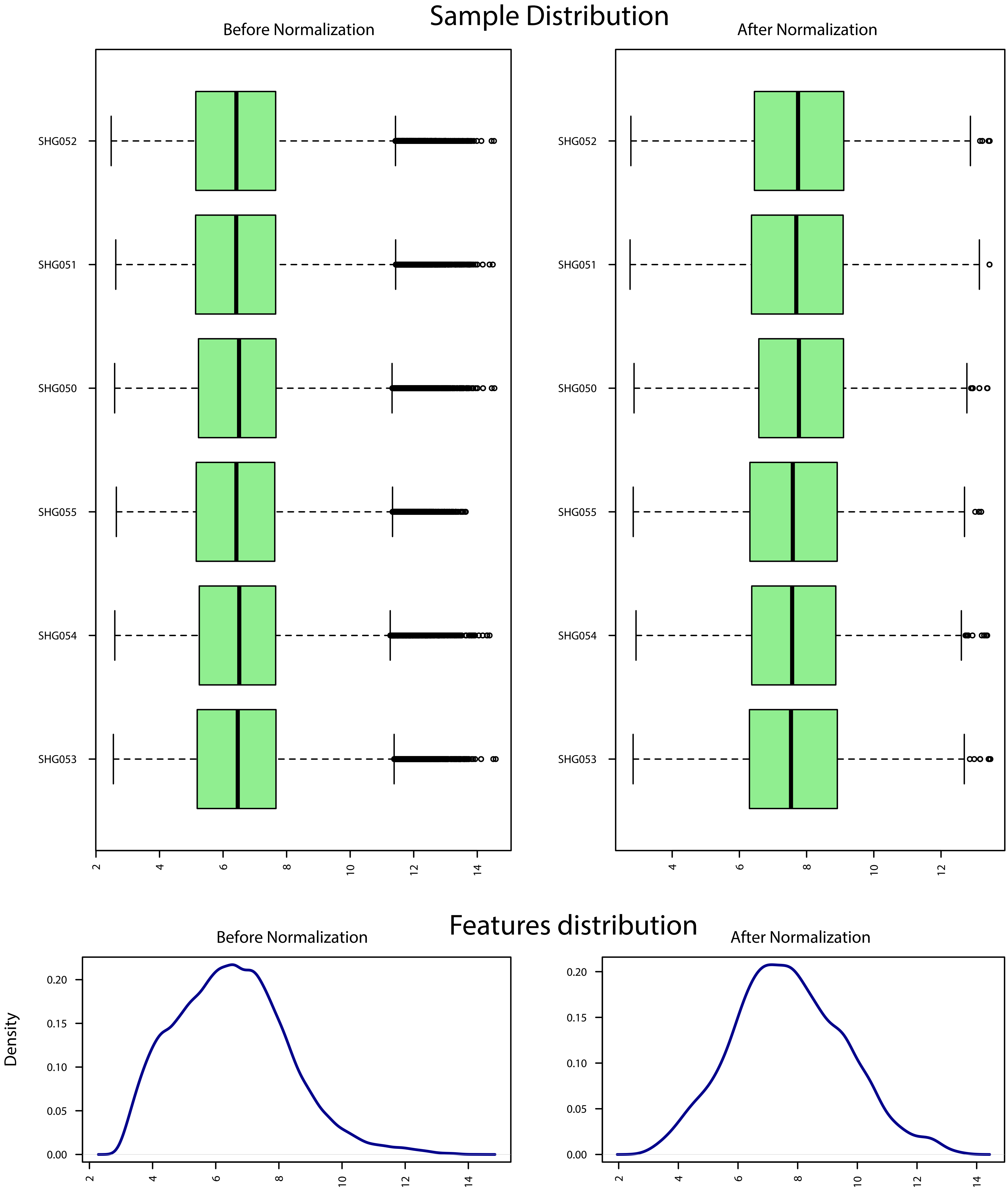
Sample Distribution
Before Normalization
After Normalization
SHG052
SHG051
SHG050
SHG055
SHG054
SHG053
2
4
6
8
10
12
14
Features distribution
Before Normalization
After Normalization
Density
0.00
0.05
0.10
0.15
0.20

Figure S5.
