# Supplementary material for: Role of HRTPT in kidney proximal epithelial cell regeneration: Integrative differential expression and pathway analyses using microarray and scRNA‐seq
Source: J Cell Mol Med. 2021 Oct 9;25(22):10466–79. doi: 10.1111/jcmm.16976 (PMC8581341; doi:10.1111/jcmm.16976)
Supplement: Supplementary file 6 — Table S1. List of primers used for ddPCR [file JCMM-25-10466-s006.docx]

| **Table S1**. List of primers use for ddPCR | | | |
| --- | --- | --- | --- |
|  | Genes | Catalog # / Unique Assay ID | Company |
| 1 | ANXA2 | qhsacid0038960 | BIORAD |
| 2 | ANXA3 | qhsaced0056400 | BIORAD |
| 3 | ANXA6 | qhsaced0056401 | BIORAD |
| 4 | TUBA1A | qhsaced0038944 | BIORAD |
| 5 | TUBA1B | qhsaced0038685 | BIORAD |
| 6 | TUBB2A | qhsaced0044757 | BIORAD |
| 7 | TUBB3 | qhsaced0005794 | BIORAD |
| 8 | TUBB | qhsaced0037246 | BIORAD |
| 9 | RHO | qHsaCID0016495 | BIORAD |
| 10 | RHOBTB1 | qhsaced0042320 | BIORAD |
| 11 | RAB11A | qhsacid0008475 | BIORAD |
| 12 | RAB19 | qhsaced0045347 | BIORAD |
| 13 | RAB27B | qhsacid0015714 | BIORAD |
| 14 | RAB30 | qhsaced0005490 | BIORAD |
| 15 | RAB31 | qhsaced0046554 | BIORAD |
| 16 | RAP2B | qhsaced0047463 | BIORAD |
| 17 | RERG | qhsaced0002401 | BIORAD |
| 18 | RND3 | qhsaced0043653 | BIORAD |
| 19 | GUCA1C | qhsacid0016674 | BIORAD |
| 20 | MYL12A | qhsaced0042877 | BIORAD |
| 21 | ITGA1 | qhsacid0017712 | BIORAD |
| 22 | ITGA2 | qhsacid0016134 | BIORAD |
| 23 | ITGB8 | qhsaced0043106 | BIORAD |
| 24 | ITGAV | qhsacid0006233 | BIORAD |
| 25 | NBPF9 | qhsaced0038145 | BIORAD |
| 26 | NBPF14 | qhsacid0037076 | BIORAD |
| 27 | NBPF15 | qhsacid0041770 | BIORAD |
| 28 | GJA1 | qhsacid0012977 | BIORAD |
| 29 | PRKAA2 | qhsacid0016799 | BIORAD |
| 30 | ACTG1 | qhsaced0005010 | BIORAD |
| 31 | CCT5 | qhsaced0004869 | BIORAD |
| 32 | FGFR1 | qhsaced0042405 | BIORAD |
| 33 | FGFR2 | qhsacid0021928 | BIORAD |
| 34 | FGFR3 | qHsaCED0004573 | BIORAD |
| 35 | FGFR4 | qhsaced0045915 | BIORAD |
| 36 | FGF1 | qhsaced0002206 | BIORAD |
| 37 | FGF2 | qhsaced0056993 | BIORAD |
| 38 | FGF7 | qhsaced0044952 | BIORAD |
| 39 | FGF9 | qhsacid0011429 | BIORAD |
| 40 | FGF20 | qhsacid0009517 | BIORAD |
| 41 | FGF22 | qhsaced0005584 | BIORAD |
| 42 | RXRA | qhsacid0005916 | BIORAD |
| 43 | VDR | qhsacid0023190 | BIORAD |
| 44 | NR1D1 | qhsacid0015596 | BIORAD |
| 45 | IGFBP3 | qhsacid0010824 | BIORAD |
| 46 | SLC7A2 | qhsacid0015651 | BIORAD |
| 47 | SLC9A1 | qhsacid0012512 | BIORAD |
| 48 | SLC47A1 | qhsacid0017630 | BIORAD |
| 49 | ESR1 | qhsaced0042641 | BIORAD |
