# Supplementary material for: Role of HRTPT in kidney proximal epithelial cell regeneration: Integrative differential expression and pathway analyses using microarray and scRNA‐seq
Source: J Cell Mol Med. 2021 Oct 9;25(22):10466–79. doi: 10.1111/jcmm.16976 (PMC8581341; doi:10.1111/jcmm.16976)
Supplement: Supplementary file 7 — Table S2. List of Antibodies, source from where they were purchased, catalog number and dilution used for western blot [file JCMM-25-10466-s009.docx]

| **Table S2.** List of Antibodies, source from where they were purchased, catalog number and dilution used | | | |
| --- | --- | --- | --- |
| **Antigen** | **Source** | **catalog #** | **dilution** |
| p-FGFR | R&D Systems | AF3285-SP | 1:100 |
| t-FGFR2 | Cell Signaling | 23328 | 1:250 |
| β-actin | abcam | 8226 | 1:5000 |
