# Supplementary material for: Role of HRTPT in kidney proximal epithelial cell regeneration: Integrative differential expression and pathway analyses using microarray and scRNA‐seq
Source: J Cell Mol Med. 2021 Oct 9;25(22):10466–79. doi: 10.1111/jcmm.16976 (PMC8581341; doi:10.1111/jcmm.16976)
Supplement: Supplementary file 8 — Table S3. List of 1483 probes (873 unique genes) that were differently expressed between HRTPT and HREC24T cell line (P‐value<0.05) [file JCMM-25-10466-s014.docx]

| Probes | t.stat | p.value | X.log10.p. | FDR | GeneName |
| --- | --- | --- | --- | --- | --- |
| TC04002940.hg.1 | -101.72 | 5.60E-08 | 7.2518 | 0.00014809 | PROM1 |
| TC03001046.hg.1 | -88.466 | 9.79E-08 | 7.0093 | 0.00014809 | CLDN16 |
| TC03001605.hg.1 | -83.904 | 1.21E-07 | 6.9174 | 0.00014809 | RNU6-26P |
| TC02001238.hg.1 | -80.021 | 1.46E-07 | 6.8351 | 0.00014809 | MAP2 |
| TC05000461.hg.1 | -79.758 | 1.48E-07 | 6.8294 | 0.00014809 | RHOBTB3 |
| TC13001044.hg.1 | -66.743 | 3.02E-07 | 6.5201 | 0.00025154 | NA |
| TC06000909.hg.1 | -57.999 | 5.29E-07 | 6.2764 | 0.00037793 | MARCKS |
| TC04000178.hg.1 | -46.333 | 1.30E-06 | 5.8868 | 0.00076121 | SLC34A2 |
| TC11002162.hg.1 | -44.386 | 1.54E-06 | 5.8123 | 0.00076121 | SYTL2 |
| TC01003900.hg.1 | 43.508 | 1.67E-06 | 5.7777 | 0.00076121 | NA |
| TC01001090.hg.1 | -42.056 | 1.91E-06 | 5.7188 | 0.00076121 | TXNIP |
| TC05001804.hg.1 | -41.771 | 1.96E-06 | 5.707 | 0.00076121 | CXCL14 |
| TC02000711.hg.1 | 41.685 | 1.98E-06 | 5.7034 | 0.00076121 | SLC20A1 |
| TC07001546.hg.1 | -38.835 | 2.63E-06 | 5.5806 | 0.0009378 | FGL2 |
| TC12000251.hg.1 | 37.328 | 3.08E-06 | 5.5121 | 0.00096366 | LRMP |
| TC17000241.hg.1 | 37.302 | 3.08E-06 | 5.5108 | 0.00096366 | SLC47A1 |
| TC12001080.hg.1 | 35.756 | 3.65E-06 | 5.4375 | 0.0010469 | SLC6A13 |
| TC02000786.hg.1 | 35.472 | 3.77E-06 | 5.4237 | 0.0010469 | MYO7B |
| TC15000270.hg.1 | -34.513 | 4.21E-06 | 5.3762 | 0.0011065 | THBS1 |
| TC01004040.hg.1 | -33.961 | 4.48E-06 | 5.3483 | 0.0011209 | AKT3 |
| TC15002797.hg.1 | -33.508 | 4.73E-06 | 5.325 | 0.0011262 | SLC12A1 |
| TC22000785.hg.1 | 32.79 | 5.16E-06 | 5.2875 | 0.0011721 | BAIAP2L2 |
| TC11003279.hg.1 | -32.391 | 5.42E-06 | 5.2663 | 0.0011772 | SYTL2 |
| TC21000134.hg.1 | 31.055 | 6.41E-06 | 5.1934 | 0.0013344 | SLC5A3 |
| TC08001312.hg.1 | -30.156 | 7.20E-06 | 5.1425 | 0.0014403 | TRAM1 |
| TC13000855.hg.1 | -29.685 | 7.67E-06 | 5.1153 | 0.0014744 | KDELC1 |
| TC01002493.hg.1 | -29.177 | 8.22E-06 | 5.0854 | 0.001521 | SFPQ |
| TC01006163.hg.1 | 28.093 | 9.55E-06 | 5.0199 | 0.0017053 | NA |
| TC04001338.hg.1 | -26.176 | 1.27E-05 | 4.8977 | 0.002142 | SCD5 |
| TC05000382.hg.1 | -26.001 | 1.30E-05 | 4.8861 | 0.002142 | NA |
| TC17001904.hg.1 | 25.572 | 1.39E-05 | 4.8573 | 0.002142 | ST6GALNAC1 |
| TC12000134.hg.1 | 25.536 | 1.40E-05 | 4.8549 | 0.002142 | RIMKLB |
| TC0X001624.hg.1 | -25.457 | 1.41E-05 | 4.8496 | 0.002142 | SAT1 |
| TC05001529.hg.1 | -25.242 | 1.46E-05 | 4.8348 | 0.0021506 | LOC644936 |
| TC01003629.hg.1 | -24.905 | 1.54E-05 | 4.8117 | 0.0022037 | IVNS1ABP |
| TC22000972.hg.1 | 24.521 | 1.64E-05 | 4.7848 | 0.0022791 | NA |
| TC06001083.hg.1 | -24.154 | 1.74E-05 | 4.7587 | 0.0022915 | NA |
| TC07001579.hg.1 | -24.014 | 1.78E-05 | 4.7487 | 0.0022915 | ABCB1 |
| TC10001320.hg.1 | -24 | 1.79E-05 | 4.7477 | 0.0022915 | RHOBTB1 |
| TC04001180.hg.1 | -22.997 | 2.12E-05 | 4.674 | 0.0026304 | OCIAD2 |
| TC11000278.hg.1 | -22.849 | 2.17E-05 | 4.6628 | 0.0026304 | BBOX1 |
| TC11003415.hg.1 | 22.715 | 2.23E-05 | 4.6527 | 0.0026304 | NA |
| TC12001181.hg.1 | 22.56 | 2.29E-05 | 4.6409 | 0.0026304 | MFAP5 |
| TC02003490.hg.1 | 22.489 | 2.32E-05 | 4.6354 | 0.0026304 | LINC01123 |
| TC03000262.hg.1 | -21.832 | 2.60E-05 | 4.5843 | 0.0027976 | PTH1R |
| TC12001472.hg.1 | -21.507 | 2.76E-05 | 4.5584 | 0.0027976 | TUBA1A |
| TC09001875.hg.1 | -21.445 | 2.80E-05 | 4.5534 | 0.0027976 | ANXA2P2 |
| 3447826_st | -21.351 | 2.85E-05 | 4.5458 | 0.0027976 | NA |
| TC01000723.hg.1 | -21.261 | 2.89E-05 | 4.5386 | 0.0027976 | ROR1 |
| TC22000427.hg.1 | 21.169 | 2.94E-05 | 4.5311 | 0.0027976 | MIOX |
| TC03002816.hg.1 | -21.161 | 2.95E-05 | 4.5304 | 0.0027976 | NA |
| TC10001232.hg.1 | -21.154 | 2.95E-05 | 4.5299 | 0.0027976 | BMS1P5 |
| TC17002878.hg.1 | -21.129 | 2.97E-05 | 4.5278 | 0.0027976 | ITGB3 |
| TC02000034.hg.1 | -20.848 | 3.13E-05 | 4.5048 | 0.0028682 | RSAD2 |
| TC01003147.hg.1 | -20.802 | 3.16E-05 | 4.5009 | 0.0028682 | LOC727820 |
| TC06002880.hg.1 | -20.608 | 3.27E-05 | 4.4848 | 0.0028771 | MYO6 |
| TC16001335.hg.1 | 20.599 | 3.28E-05 | 4.484 | 0.0028771 | SLC7A5 |
| TC08000127.hg.1 | 20.06 | 3.65E-05 | 4.4383 | 0.0030844 | SLC7A2 |
| TC03001691.hg.1 | -20.041 | 3.66E-05 | 4.4367 | 0.0030844 | FSTL1 |
| TC08001325.hg.1 | -19.981 | 3.70E-05 | 4.4316 | 0.0030844 | SBSPON |
| TC01001805.hg.1 | -19.878 | 3.78E-05 | 4.4226 | 0.0030968 | TGFB2 |
| TC05002442.hg.1 | -19.359 | 4.20E-05 | 4.377 | 0.0032289 | NA |
| TC10001711.hg.1 | -19.351 | 4.20E-05 | 4.3764 | 0.0032289 | FGFR2 |
| TC0X002096.hg.1 | -19.296 | 4.25E-05 | 4.3714 | 0.0032289 | ZC4H2 |
| TC01001314.hg.1 | 19.286 | 4.26E-05 | 4.3705 | 0.0032289 | SEMA4A |
| TC02001769.hg.1 | 19.188 | 4.35E-05 | 4.3618 | 0.0032289 | SLC8A1 |
| TC04001060.hg.1 | -19.16 | 4.37E-05 | 4.3593 | 0.0032289 | NA |
| TC04000437.hg.1 | -19.098 | 4.43E-05 | 4.3537 | 0.0032289 | ANXA3 |
| TC05000132.hg.1 | -18.968 | 4.55E-05 | 4.3419 | 0.0032289 | CDH6 |
| PSR11023315.hg.1 | -18.967 | 4.55E-05 | 4.3419 | 0.0032289 | NA |
| TC14001451.hg.1 | 18.922 | 4.59E-05 | 4.3378 | 0.0032289 | LGMN |
| TC1_gl000192_random000006.hg.1 | -18.864 | 4.65E-05 | 4.3325 | 0.0032289 | NA |
| TC16000126.hg.1 | 18.734 | 4.78E-05 | 4.3206 | 0.0032502 | DNASE1 |
| TC04000253.hg.1 | -18.628 | 4.89E-05 | 4.3108 | 0.0032502 | UCHL1 |
| TC10002021.hg.1 | -18.625 | 4.89E-05 | 4.3105 | 0.0032502 | NA |
| TC14001508.hg.1 | 18.536 | 4.99E-05 | 4.3023 | 0.0032502 | SLC25A29 |
| TC10000420.hg.1 | -18.517 | 5.01E-05 | 4.3005 | 0.0032502 | TSPAN15 |
| TC02002579.hg.1 | 18.397 | 5.14E-05 | 4.2893 | 0.0032605 | ZNF385B |
| TC13000824.hg.1 | -18.334 | 5.21E-05 | 4.2835 | 0.0032605 | SLC15A1 |
| TC10002080.hg.1 | -18.292 | 5.25E-05 | 4.2795 | 0.0032605 | NA |
| TC0X000512.hg.1 | -18.215 | 5.34E-05 | 4.2722 | 0.0032605 | NRK |
| TC02001420.hg.1 | 18.21 | 5.35E-05 | 4.2718 | 0.0032605 | UGT1A1 |
| TC09001683.hg.1 | 17.741 | 5.93E-05 | 4.2269 | 0.0035718 | ABO |
| TC17000312.hg.1 | -17.555 | 6.18E-05 | 4.2088 | 0.0036218 | LGALS9 |
| TC09001507.hg.1 | 17.548 | 6.19E-05 | 4.2081 | 0.0036218 | KIF12 |
| TC06000976.hg.1 | 17.486 | 6.28E-05 | 4.202 | 0.0036218 | LAMA2 |
| TC12000418.hg.1 | -17.47 | 6.30E-05 | 4.2004 | 0.0036218 | KRT7 |
| TC11001425.hg.1 | -17.204 | 6.70E-05 | 4.1741 | 0.0038048 | DKK3 |
| TC12001837.hg.1 | -17.126 | 6.82E-05 | 4.1663 | 0.0038301 | NTN4 |
| TC0X000388.hg.1 | -17.02 | 6.99E-05 | 4.1557 | 0.0038719 | OGT |
| TC08001313.hg.1 | -16.983 | 7.05E-05 | 4.1519 | 0.0038719 | LACTB2 |
| TC08002303.hg.1 | -16.836 | 7.29E-05 | 4.137 | 0.0039637 | SFRP1 |
| TC19000924.hg.1 | -16.749 | 7.45E-05 | 4.1281 | 0.0040023 | ZNF667-AS1 |
| TC09000560.hg.1 | -16.681 | 7.57E-05 | 4.1211 | 0.0040163 | UGCG |
| TC10000715.hg.1 | 16.645 | 7.63E-05 | 4.1173 | 0.0040163 | ABCC2 |
| TC07002809.hg.1 | -16.496 | 7.91E-05 | 4.1019 | 0.0041127 | LOC105375172 |
| TC02000270.hg.1 | -16.419 | 8.06E-05 | 4.0939 | 0.0041127 | PLEKHH2 |
| TC20001724.hg.1 | 16.415 | 8.06E-05 | 4.0935 | 0.0041127 | SGK2 |
| TC11002996.hg.1 | 16.332 | 8.23E-05 | 4.0848 | 0.0041278 | NA |
| TC05001731.hg.1 | -16.308 | 8.27E-05 | 4.0823 | 0.0041278 | ZNF608 |
| TC05001620.hg.1 | -16.276 | 8.34E-05 | 4.0788 | 0.0041278 | LIX1 |
| TC14000375.hg.1 | -16.008 | 8.90E-05 | 4.0504 | 0.0043375 | SYT16 |
| TC04000517.hg.1 | -15.993 | 8.94E-05 | 4.0488 | 0.0043375 | DAPP1 |
| TC08001654.hg.1 | -15.924 | 9.09E-05 | 4.0413 | 0.0043425 | LRRC6 |
| TC09000923.hg.1 | -15.911 | 9.12E-05 | 4.04 | 0.0043425 | TTC39B |
| TC01000080.hg.1 | -15.704 | 9.61E-05 | 4.0175 | 0.0044927 | AJAP1 |
| TC09002631.hg.1 | 15.699 | 9.62E-05 | 4.017 | 0.0044927 | NA |
| TC01001602.hg.1 | -15.624 | 9.80E-05 | 4.0087 | 0.0045156 | HMCN1 |
| TC19002567.hg.1 | -15.55 | 9.99E-05 | 4.0006 | 0.0045156 | ZNF600 |
| TC01003875.hg.1 | -15.547 | 9.99E-05 | 4.0003 | 0.0045156 | NA |
| TC01000789.hg.1 | -15.534 | 0.00010027 | 3.9988 | 0.0045156 | NEXN |
| TC04001347.hg.1 | -15.494 | 0.00010127 | 3.9945 | 0.0045201 | HPSE |
| TC08001431.hg.1 | 15.426 | 0.00010305 | 3.9869 | 0.0045255 | CDH17 |
| TC08001572.hg.1 | 15.397 | 0.00010381 | 3.9838 | 0.0045255 | LINC01151 |
| TC08001866.hg.1 | -15.359 | 0.00010483 | 3.9795 | 0.0045255 | NA |
| TC17001254.hg.1 | -15.353 | 0.00010501 | 3.9788 | 0.0045255 | LGALS9C |
| TC11002708.hg.1 | 15.243 | 0.00010803 | 3.9665 | 0.0046157 | SLC3A2 |
| TC13000347.hg.1 | -15.148 | 0.00011072 | 3.9558 | 0.0046443 | NA |
| TC11002606.hg.1 | -15.125 | 0.00011139 | 3.9532 | 0.0046443 | BBOX1 |
| TC11000247.hg.1 | 15.121 | 0.00011148 | 3.9528 | 0.0046443 | NA |
| TC02001361.hg.1 | 15.087 | 0.0001125 | 3.9489 | 0.0046476 | COL4A3 |
| TC01004449.hg.1 | -15.004 | 0.00011497 | 3.9394 | 0.0047111 | ROR1 |
| TC10001559.hg.1 | -14.969 | 0.00011603 | 3.9354 | 0.0047157 | PIK3AP1 |
| TC06002925.hg.1 | -14.894 | 0.00011835 | 3.9268 | 0.0047711 | NA |
| TC06000114.hg.1 | -14.776 | 0.00012211 | 3.9132 | 0.0048058 | CAP2 |
| TC09000687.hg.1 | -14.741 | 0.00012326 | 3.9092 | 0.0048058 | TMSB4XP4 |
| TC05002968.hg.1 | 14.719 | 0.00012398 | 3.9066 | 0.0048058 | FYB |
| TC12000778.hg.1 | -14.701 | 0.00012459 | 3.9045 | 0.0048058 | NR1H4 |
| TC02000423.hg.1 | -14.683 | 0.00012519 | 3.9024 | 0.0048058 | ATP6V1B1 |
| TC01003050.hg.1 | 14.641 | 0.0001266 | 3.8976 | 0.0048058 | HMGCS2 |
| TC16000036.hg.1 | 14.636 | 0.0001268 | 3.8969 | 0.0048058 | CACNA1H |
| TC01004159.hg.1 | -14.569 | 0.00012909 | 3.8891 | 0.0048058 | AJAP1 |
| TC15001260.hg.1 | 14.528 | 0.00013054 | 3.8843 | 0.0048058 | PLA2G4F |
| TC13000634.hg.1 | -14.523 | 0.00013071 | 3.8837 | 0.0048058 | SLC25A30 |
| TC01003791.hg.1 | -14.498 | 0.00013159 | 3.8808 | 0.0048058 | KCNH1 |
| TC19000670.hg.1 | -14.497 | 0.00013163 | 3.8806 | 0.0048058 | NA |
| TC20000076.hg.1 | -14.453 | 0.00013321 | 3.8755 | 0.0048058 | PLCB4 |
| TC10002665.hg.1 | -14.453 | 0.00013321 | 3.8755 | 0.0048058 | RHOBTB1 |
| TC09000075.hg.1 | -14.375 | 0.00013608 | 3.8662 | 0.0048058 | SNAPC3 |
| TC04000254.hg.1 | -14.374 | 0.00013615 | 3.866 | 0.0048058 | LIMCH1 |
| TC01005002.hg.1 | -14.369 | 0.00013632 | 3.8654 | 0.0048058 | SYT14 |
| TC20000913.hg.1 | -14.364 | 0.00013651 | 3.8648 | 0.0048058 | SULF2 |
| TC03001979.hg.1 | 14.326 | 0.00013794 | 3.8603 | 0.0048222 | MECOM |
| TC09002111.hg.1 | -14.097 | 0.00014696 | 3.8328 | 0.0051019 | NA |
| TC01000230.hg.1 | 13.939 | 0.00015364 | 3.8135 | 0.005297 | PADI1 |
| TC02004486.hg.1 | 13.883 | 0.00015607 | 3.8067 | 0.0053354 | NA |
| TC11000329.hg.1 | -13.839 | 0.00015804 | 3.8012 | 0.0053354 | EHF |
| TC03001713.hg.1 | -13.828 | 0.00015852 | 3.7999 | 0.0053354 | MYLK |
| TC20000602.hg.1 | -13.817 | 0.00015903 | 3.7985 | 0.0053354 | LRRN4 |
| TC11001793.hg.1 | 13.781 | 0.00016067 | 3.7941 | 0.0053544 | SLC43A1 |
| TC01001825.hg.1 | 13.727 | 0.00016316 | 3.7874 | 0.0053628 | NA |
| TC21000737.hg.1 | 13.724 | 0.00016333 | 3.7869 | 0.0053628 | BACE2 |
| TC11002228.hg.1 | -13.693 | 0.00016477 | 3.7831 | 0.0053628 | MMP7 |
| TC17001830.hg.1 | 13.684 | 0.00016521 | 3.782 | 0.0053628 | ABCA5 |
| TC02002419.hg.1 | -13.642 | 0.00016721 | 3.7767 | 0.0053927 | RND3 |
| TC14001538.hg.1 | -13.585 | 0.00016996 | 3.7697 | 0.0054139 | CKB |
| TC22000455.hg.1 | -13.584 | 0.00017003 | 3.7695 | 0.0054139 | NA |
| TC04000804.hg.1 | 13.555 | 0.00017148 | 3.7658 | 0.0054149 | RXFP1 |
| TC07001182.hg.1 | -13.54 | 0.00017223 | 3.7639 | 0.0054149 | MACC1 |
| TC11002605.hg.1 | -13.517 | 0.00017336 | 3.761 | 0.0054165 | BBOX1 |
| TC13001247.hg.1 | -13.474 | 0.00017553 | 3.7556 | 0.0054502 | NA |
| TC09002827.hg.1 | 13.429 | 0.00017784 | 3.75 | 0.0054879 | NA |
| TC08001864.hg.1 | -13.398 | 0.00017949 | 3.746 | 0.0055046 | RBPMS |
| TC18000770.hg.1 | 13.374 | 0.00018078 | 3.7429 | 0.0055068 | NA |
| TC06000836.hg.1 | 13.335 | 0.00018283 | 3.738 | 0.0055068 | GRIK2 |
| TC12000684.hg.1 | -13.335 | 0.00018286 | 3.7379 | 0.0055068 | LRRIQ1 |
| TC08001985.hg.1 | -13.284 | 0.00018559 | 3.7314 | 0.0055349 | LINC01109 |
| TC07001618.hg.1 | 13.255 | 0.00018719 | 3.7277 | 0.0055349 | PDK4 |
| TC01004613.hg.1 | 13.242 | 0.00018792 | 3.726 | 0.0055349 | KCNC4 |
| TC05000469.hg.1 | -13.237 | 0.00018822 | 3.7253 | 0.0055349 | CAST |
| TC21000736.hg.1 | 13.202 | 0.00019018 | 3.7208 | 0.0055478 | BACE2 |
| TC05003109.hg.1 | -13.152 | 0.00019304 | 3.7144 | 0.0055478 | LOC644936 |
| TC15000945.hg.1 | -13.151 | 0.0001931 | 3.7142 | 0.0055478 | ARRDC4 |
| TC08000241.hg.1 | -13.104 | 0.0001958 | 3.7082 | 0.0055478 | NA |
| TC18000245.hg.1 | 13.103 | 0.00019589 | 3.708 | 0.0055478 | LOC100505817 |
| TC08001515.hg.1 | -13.072 | 0.00019769 | 3.704 | 0.0055478 | NA |
| TC01004997.hg.1 | -13.056 | 0.00019867 | 3.7019 | 0.0055478 | MIR205HG |
| TC11002081.hg.1 | -13.025 | 0.00020054 | 3.6978 | 0.0055478 | PGM2L1 |
| TC10001014.hg.1 | -13.017 | 0.00020098 | 3.6968 | 0.0055478 | PRKCQ |
| TC08000403.hg.1 | -13.014 | 0.0002012 | 3.6964 | 0.0055478 | SDCBP |
| TC03000002.hg.1 | -13.008 | 0.00020153 | 3.6957 | 0.0055478 | CHL1 |
| TC06000945.hg.1 | -12.987 | 0.00020281 | 3.6929 | 0.0055478 | GJA1 |
| TC01004246.hg.1 | -12.956 | 0.00020477 | 3.6887 | 0.0055478 | NA |
| TC04001384.hg.1 | -12.94 | 0.00020572 | 3.6867 | 0.0055478 | NA |
| TC07002185.hg.1 | -12.934 | 0.00020612 | 3.6859 | 0.0055478 | ITGB8 |
| TC05000801.hg.1 | -12.929 | 0.00020642 | 3.6853 | 0.0055478 | STK32A |
| TC11002153.hg.1 | -12.892 | 0.00020876 | 3.6804 | 0.0055636 | RAB30 |
| TC01005736.hg.1 | -12.885 | 0.00020923 | 3.6794 | 0.0055636 | VTCN1 |
| TC10000362.hg.1 | -12.825 | 0.00021309 | 3.6714 | 0.0056226 | BICC1 |
| TC02002491.hg.1 | -12.815 | 0.0002137 | 3.6702 | 0.0056226 | SCN3A |
| TC16000264.hg.1 | -12.689 | 0.00022214 | 3.6534 | 0.0057926 | TNRC6A |
| TC15000800.hg.1 | -12.669 | 0.00022353 | 3.6507 | 0.0057926 | ADAMTSL3 |
| TC08002026.hg.1 | -12.668 | 0.00022364 | 3.6505 | 0.0057926 | CPQ |
| TC09001589.hg.1 | -12.633 | 0.00022603 | 3.6458 | 0.0058134 | HSPA5 |
| TC01005824.hg.1 | -12.623 | 0.00022677 | 3.6444 | 0.0058134 | S100A10 |
| TC06000097.hg.1 | -12.573 | 0.00023032 | 3.6377 | 0.0058743 | RNF182 |
| TC22000195.hg.1 | -12.418 | 0.00024174 | 3.6166 | 0.0061344 | EWSR1 |
| TC02003288.hg.1 | 12.369 | 0.00024552 | 3.6099 | 0.0061596 | NA |
| TC01001753.hg.1 | -12.362 | 0.0002461 | 3.6089 | 0.0061596 | SYT14 |
| TC17001459.hg.1 | 12.347 | 0.00024723 | 3.6069 | 0.0061596 | NR1D1 |
| TC01002971.hg.1 | 12.342 | 0.00024767 | 3.6061 | 0.0061596 | SLC16A4 |
| TC10000839.hg.1 | -12.293 | 0.00025156 | 3.5994 | 0.0062254 | ATRNL1 |
| TC03002529.hg.1 | -12.233 | 0.0002564 | 3.5911 | 0.0063141 | NA |
| TC18000189.hg.1 | -12.191 | 0.00025984 | 3.5853 | 0.0063673 | RAB27B |
| TC16000223.hg.1 | 12.154 | 0.00026294 | 3.5801 | 0.0064055 | ACSM3 |
| TC12000803.hg.1 | -12.142 | 0.00026396 | 3.5785 | 0.0064055 | HSP90B1 |
| TC03000676.hg.1 | -12.11 | 0.00026678 | 3.5738 | 0.0064231 | LOC90246 |
| TC02002428.hg.1 | -12.09 | 0.00026844 | 3.5711 | 0.0064231 | NEB |
| TC01001106.hg.1 | -12.076 | 0.00026973 | 3.5691 | 0.0064231 | NBPF1 |
| TC10000475.hg.1 | -12.074 | 0.00026982 | 3.5689 | 0.0064231 | PLAU |
| TC06002615.hg.1 | -12.001 | 0.00027635 | 3.5585 | 0.0065472 | SOX4 |
| TC01005632.hg.1 | 11.961 | 0.00027998 | 3.5529 | 0.0066019 | TMED5 |
| TC17000047.hg.1 | 11.9 | 0.00028564 | 3.5442 | 0.0067037 | ARRB2 |
| TC01002401.hg.1 | 11.873 | 0.00028813 | 3.5404 | 0.0067074 | SLC9A1 |
| TC15001532.hg.1 | 11.87 | 0.00028848 | 3.5399 | 0.0067074 | GOLGA2P11 |
| TC01000818.hg.1 | -11.817 | 0.00029351 | 3.5324 | 0.006764 | CYR61 |
| TC03002264.hg.1 | -11.816 | 0.00029362 | 3.5322 | 0.006764 | NA |
| TC17000728.hg.1 | -11.74 | 0.00030116 | 3.5212 | 0.0068752 | MIR21 |
| TC08000618.hg.1 | 11.727 | 0.00030247 | 3.5193 | 0.0068752 | NA |
| TC17001711.hg.1 | -11.726 | 0.00030257 | 3.5192 | 0.0068752 | MMD |
| TC1_gl000192_random000003.hg.1 | -11.71 | 0.0003042 | 3.5168 | 0.0068811 | HYDIN |
| TC14001727.hg.1 | -11.661 | 0.00030914 | 3.5098 | 0.0069612 | NA |
| TC18000205.hg.1 | -11.639 | 0.00031143 | 3.5066 | 0.0069709 | MALT1 |
| TC05002527.hg.1 | -11.613 | 0.00031423 | 3.5028 | 0.0069709 | CAST |
| TC03000448.hg.1 | -11.602 | 0.00031532 | 3.5013 | 0.0069709 | NA |
| TC01003042.hg.1 | -11.591 | 0.00031656 | 3.4995 | 0.0069709 | TBX15 |
| TC06003006.hg.1 | -11.589 | 0.0003167 | 3.4994 | 0.0069709 | MARCKS |
| TC01006361.hg.1 | 11.578 | 0.00031793 | 3.4977 | 0.0069709 | GBP2 |
| TC06000747.hg.1 | -11.519 | 0.00032434 | 3.489 | 0.0070598 | MYO6 |
| TC09000330.hg.1 | 11.505 | 0.00032587 | 3.487 | 0.0070598 | GDA |
| TC19000232.hg.1 | -11.461 | 0.00033074 | 3.4805 | 0.0070598 | CALR |
| TC15001462.hg.1 | 11.453 | 0.00033171 | 3.4792 | 0.0070598 | WDR72 |
| TC02002746.hg.1 | 11.453 | 0.00033173 | 3.4792 | 0.0070598 | ABCA12 |
| TC01000100.hg.1 | 11.452 | 0.00033179 | 3.4791 | 0.0070598 | PER3 |
| TC22000953.hg.1 | 11.451 | 0.00033188 | 3.479 | 0.0070598 | NA |
| TC20000494.hg.1 | 11.412 | 0.00033629 | 3.4733 | 0.0071234 | SLCO4A1 |
| TC05000472.hg.1 | -11.36 | 0.00034237 | 3.4655 | 0.0071878 | LNPEP |
| TC03002405.hg.1 | -11.357 | 0.00034276 | 3.465 | 0.0071878 | NA |
| TC19001810.hg.1 | -11.347 | 0.00034394 | 3.4635 | 0.0071878 | ZNF702P |
| TC19002060.hg.1 | -11.337 | 0.00034509 | 3.4621 | 0.0071878 | NA |
| TC16000480.hg.1 | -11.325 | 0.00034652 | 3.4603 | 0.0071878 | HERPUD1 |
| TC0X000053.hg.1 | -11.285 | 0.00035139 | 3.4542 | 0.007202 | TMSB4X |
| TC03000627.hg.1 | -11.281 | 0.00035183 | 3.4537 | 0.007202 | CASR |
| 3151639_st | -11.275 | 0.00035252 | 3.4528 | 0.007202 | NA |
| TC01003771.hg.1 | -11.272 | 0.00035297 | 3.4523 | 0.007202 | C1orf116 |
| TC07002818.hg.1 | -11.246 | 0.00035607 | 3.4485 | 0.0072165 | MACC1 |
| TC01000236.hg.1 | 11.212 | 0.00036038 | 3.4432 | 0.0072165 | ARHGEF10L |
| TC17000383.hg.1 | -11.207 | 0.00036098 | 3.4425 | 0.0072165 | CCL2 |
| TC13001043.hg.1 | -11.204 | 0.00036133 | 3.4421 | 0.0072165 | NA |
| TC02000274.hg.1 | 11.202 | 0.00036158 | 3.4418 | 0.0072165 | PPM1B |
| TC08002263.hg.1 | -11.18 | 0.00036436 | 3.4385 | 0.0072165 | CLU |
| TC06003712.hg.1 | -11.178 | 0.00036459 | 3.4382 | 0.0072165 | NA |
| TC22000488.hg.1 | 11.173 | 0.00036523 | 3.4374 | 0.0072165 | PRODH |
| TC06001796.hg.1 | -11.147 | 0.00036864 | 3.4334 | 0.0072318 | PKHD1 |
| TC02000750.hg.1 | 11.145 | 0.0003689 | 3.4331 | 0.0072318 | STEAP3 |
| TC10002603.hg.1 | -11.122 | 0.00037185 | 3.4296 | 0.0072369 | NA |
| TC06001959.hg.1 | -11.12 | 0.00037205 | 3.4294 | 0.0072369 | MMS22L |
| TC22000467.hg.1 | -11.055 | 0.00038075 | 3.4194 | 0.0073632 | NA |
| TC04001650.hg.1 | 11.049 | 0.00038149 | 3.4185 | 0.0073632 | TMEM154 |
| TC14001144.hg.1 | -11.028 | 0.00038433 | 3.4153 | 0.0073895 | FERMT2 |
| TC11000337.hg.1 | -11.009 | 0.0003869 | 3.4124 | 0.0074104 | FJX1 |
| TC05001151.hg.1 | -10.98 | 0.00039097 | 3.4079 | 0.0074598 | SEMA5A |
| TC01002398.hg.1 | 10.96 | 0.00039371 | 3.4048 | 0.0074835 | NR0B2 |
| TC20001408.hg.1 | -10.912 | 0.0004005 | 3.3974 | 0.0075838 | NA |
| TC13001719.hg.1 | 10.872 | 0.00040633 | 3.3911 | 0.0076265 | FOXO1 |
| TC08000380.hg.1 | -10.861 | 0.00040793 | 3.3894 | 0.0076265 | XKR4 |
| TC19002613.hg.1 | -10.86 | 0.00040795 | 3.3894 | 0.0076265 | PSG4 |
| TC18000430.hg.1 | -10.838 | 0.00041118 | 3.386 | 0.0076265 | CHST9 |
| TC12000189.hg.1 | -10.837 | 0.00041138 | 3.3858 | 0.0076265 | EMP1 |
| TC07000125.hg.1 | -10.807 | 0.00041586 | 3.3811 | 0.0076265 | ITGB8 |
| TC09001955.hg.1 | 10.806 | 0.00041596 | 3.3809 | 0.0076265 | NA |
| TC05001484.hg.1 | -10.792 | 0.00041818 | 3.3786 | 0.0076265 | FAM169A |
| TC01003455.hg.1 | 10.788 | 0.00041865 | 3.3781 | 0.0076265 | SH2D1B |
| TC12000002.hg.1 | -10.786 | 0.00041894 | 3.3778 | 0.0076265 | WASH3P |
| TC17000218.hg.1 | -10.782 | 0.00041954 | 3.3772 | 0.0076265 | NA |
| TC01004452.hg.1 | -10.749 | 0.00042461 | 3.372 | 0.0076906 | AK4 |
| TC04001038.hg.1 | -10.727 | 0.00042806 | 3.3685 | 0.0077082 | NA |
| TC04001408.hg.1 | -10.723 | 0.00042866 | 3.3679 | 0.0077082 | ADH6 |
| TC19000798.hg.1 | -10.713 | 0.00043025 | 3.3663 | 0.007709 | ZNF528 |
| TC21000345.hg.1 | -10.698 | 0.00043261 | 3.3639 | 0.0077236 | ADAMTS1 |
| TC16001373.hg.1 | 10.683 | 0.00043494 | 3.3616 | 0.0077342 | MSLN |
| TC09000149.hg.1 | -10.673 | 0.00043646 | 3.3601 | 0.0077342 | ANXA2 |
| TC02001752.hg.1 | 10.665 | 0.00043785 | 3.3587 | 0.0077342 | NA |
| TC11000412.hg.1 | -10.649 | 0.00044043 | 3.3561 | 0.0077525 | PTPRJ |
| TC17002136.hg.1 | -10.637 | 0.00044236 | 3.3542 | 0.0077549 | LGALS9 |
| TC04002616.hg.1 | -10.629 | 0.00044367 | 3.3529 | 0.0077549 | NA |
| TC04000411.hg.1 | -10.617 | 0.00044549 | 3.3512 | 0.0077597 | CXCL1 |
| TC05001845.hg.1 | -10.569 | 0.00045344 | 3.3435 | 0.0078707 | TMEM173 |
| TC12001079.hg.1 | 10.524 | 0.00046098 | 3.3363 | 0.0078952 | SLC6A12 |
| TC04002954.hg.1 | 10.52 | 0.00046172 | 3.3356 | 0.0078952 | ARAP2 |
| TC09002412.hg.1 | -10.502 | 0.00046473 | 3.3328 | 0.0078952 | NA |
| TC18000952.hg.1 | -10.492 | 0.00046645 | 3.3312 | 0.0078952 | NA |
| TC08002602.hg.1 | -10.478 | 0.00046897 | 3.3289 | 0.0078952 | RP1 |
| TC16001433.hg.1 | -10.476 | 0.00046932 | 3.3285 | 0.0078952 | ITPRIPL2 |
| TC03001604.hg.1 | -10.475 | 0.00046951 | 3.3284 | 0.0078952 | DCBLD2 |
| TC05001947.hg.1 | -10.472 | 0.00046994 | 3.328 | 0.0078952 | ANXA6 |
| TC01000185.hg.1 | -10.468 | 0.00047061 | 3.3273 | 0.0078952 | TMEM51 |
| TC10000290.hg.1 | -10.468 | 0.00047065 | 3.3273 | 0.0078952 | ZNF22 |
| TC21000167.hg.1 | -10.424 | 0.00047842 | 3.3202 | 0.0079861 | KCNJ15 |
| TC01003160.hg.1 | -10.419 | 0.00047926 | 3.3194 | 0.0079861 | NA |
| TC06001521.hg.1 | 10.371 | 0.00048803 | 3.3116 | 0.0080196 | POU5F1 |
| TC01006302.hg.1 | -10.37 | 0.00048816 | 3.3114 | 0.0080196 | NBPF15 |
| TC0X001378.hg.1 | -10.37 | 0.00048824 | 3.3114 | 0.0080196 | GPC3 |
| TC09000746.hg.1 | -10.363 | 0.00048943 | 3.3103 | 0.0080196 | NUP214 |
| TC08001816.hg.1 | 10.354 | 0.00049105 | 3.3089 | 0.0080196 | SLC7A2 |
| TC01001747.hg.1 | -10.349 | 0.00049203 | 3.308 | 0.0080196 | MIR205HG |
| TC08000401.hg.1 | -10.341 | 0.0004935 | 3.3067 | 0.0080196 | FAM110B |
| TC10000312.hg.1 | -10.338 | 0.00049411 | 3.3062 | 0.0080196 | AGAP9 |
| TC15002785.hg.1 | -10.299 | 0.00050134 | 3.2999 | 0.0081107 | SNORD107 |
| TC07003297.hg.1 | -10.283 | 0.00050439 | 3.2972 | 0.0081337 | NSUN5P2 |
| TC18000716.hg.1 | -10.257 | 0.00050932 | 3.293 | 0.0081778 | NA |
| TC11000034.hg.1 | -10.247 | 0.00051123 | 3.2914 | 0.0081778 | CD151 |
| TC01006240.hg.1 | -10.234 | 0.00051388 | 3.2891 | 0.0081778 | AKT3 |
| TC05000347.hg.1 | -10.225 | 0.00051555 | 3.2877 | 0.0081778 | NA |
| TC03002108.hg.1 | -10.211 | 0.00051836 | 3.2854 | 0.0081778 | CLDN1 |
| TC21000888.hg.1 | -10.207 | 0.0005192 | 3.2847 | 0.0081778 | NA |
| TC14000554.hg.1 | -10.205 | 0.00051943 | 3.2845 | 0.0081778 | CALM3 |
| TC05002048.hg.1 | 10.193 | 0.00052179 | 3.2825 | 0.0081778 | FAM196B |
| TC07001034.hg.1 | 10.193 | 0.00052185 | 3.2825 | 0.0081778 | PAXIP1-AS1 |
| TC01000921.hg.1 | -10.169 | 0.00052664 | 3.2785 | 0.0082071 | AMY1B |
| TC17001039.hg.1 | 10.167 | 0.000527 | 3.2782 | 0.0082071 | CAMTA2 |
| TC03001714.hg.1 | -10.139 | 0.0005327 | 3.2735 | 0.0082701 | CCDC14 |
| TC06002662.hg.1 | -10.082 | 0.0005445 | 3.264 | 0.0083972 | ZNF391 |
| TC07002222.hg.1 | -10.075 | 0.00054592 | 3.2629 | 0.0083972 | CREB5 |
| TC18000440.hg.1 | -10.067 | 0.00054764 | 3.2615 | 0.0083972 | B4GALT6 |
| TC10001374.hg.1 | 10.058 | 0.0005496 | 3.26 | 0.0083972 | PSAP |
| TC08002466.hg.1 | -10.044 | 0.00055255 | 3.2576 | 0.0083972 | NA |
| TC15002814.hg.1 | -10.037 | 0.00055398 | 3.2565 | 0.0083972 | GATM |
| TC08000482.hg.1 | 10.03 | 0.0005555 | 3.2553 | 0.0083972 | RDH10 |
| TC21000157.hg.1 | 10.028 | 0.00055587 | 3.255 | 0.0083972 | SIM2 |
| TC09000335.hg.1 | -10.028 | 0.00055601 | 3.2549 | 0.0083972 | ANXA1 |
| TC08001166.hg.1 | -9.9402 | 0.00057521 | 3.2402 | 0.0086517 | SFRP1 |
| TC05001973.hg.1 | -9.9352 | 0.00057632 | 3.2393 | 0.0086517 | FAXDC2 |
| TC10002620.hg.1 | -9.8857 | 0.00058757 | 3.2309 | 0.0087941 | NA |
| TC10001110.hg.1 | -9.8721 | 0.00059071 | 3.2286 | 0.008797 | PRTFDC1 |
| TC04001504.hg.1 | -9.8696 | 0.00059128 | 3.2282 | 0.008797 | PDE5A |
| TC14000951.hg.1 | -9.8525 | 0.00059526 | 3.2253 | 0.00883 | SLC7A8 |
| TC19000357.hg.1 | 9.823 | 0.00060222 | 3.2202 | 0.0089068 | MIR3189 |
| TC0Y000160.hg.1 | -9.8153 | 0.00060405 | 3.2189 | 0.0089074 | NA |
| TC02003008.hg.1 | -9.7917 | 0.00060969 | 3.2149 | 0.0089485 | RSAD2 |
| TC07002414.hg.1 | -9.7887 | 0.00061041 | 3.2144 | 0.0089485 | NSUN5P1 |
| TC02004178.hg.1 | 9.7341 | 0.00062375 | 3.205 | 0.0091065 | SLC8A1 |
| TC02001528.hg.1 | -9.7298 | 0.00062483 | 3.2042 | 0.0091065 | TMSB4XP2 |
| TC13000335.hg.1 | 9.7068 | 0.00063057 | 3.2003 | 0.00915 | CLDN10 |
| TC0X001530.hg.1 | -9.6869 | 0.00063557 | 3.1968 | 0.00915 | FLNA |
| TC06000737.hg.1 | -9.6822 | 0.00063678 | 3.196 | 0.00915 | CD109 |
| TC10000979.hg.1 | -9.6805 | 0.00063721 | 3.1957 | 0.00915 | KLF6 |
| TC13001240.hg.1 | 9.6796 | 0.00063743 | 3.1956 | 0.00915 | CLDN10 |
| TC0X002307.hg.1 | -9.6701 | 0.00063987 | 3.1939 | 0.00915 | L1CAM |
| TC04001615.hg.1 | 9.6671 | 0.00064063 | 3.1934 | 0.00915 | ZNF827 |
| TC05000244.hg.1 | -9.6532 | 0.0006442 | 3.191 | 0.0091748 | NA |
| TC21000168.hg.1 | -9.6315 | 0.00064981 | 3.1872 | 0.0092131 | NA |
| TC05000935.hg.1 | -9.6286 | 0.00065057 | 3.1867 | 0.0092131 | KCNIP1 |
| TC01002807.hg.1 | -9.607 | 0.00065623 | 3.1829 | 0.0092415 | ADGRL4 |
| TC11000752.hg.1 | -9.6069 | 0.00065628 | 3.1829 | 0.0092415 | NA |
| TC21000738.hg.1 | 9.5911 | 0.00066045 | 3.1802 | 0.0092742 | BACE2 |
| TC04002491.hg.1 | -9.5804 | 0.0006633 | 3.1783 | 0.0092881 | ERVH-1 |
| TC11002074.hg.1 | 9.5679 | 0.00066667 | 3.1761 | 0.0093091 | UCP2 |
| TC18000877.hg.1 | -9.55 | 0.00067148 | 3.173 | 0.0093174 | NA |
| TC20000349.hg.1 | -9.5489 | 0.00067178 | 3.1728 | 0.0093174 | WFDC2 |
| TC04000484.hg.1 | -9.545 | 0.00067285 | 3.1721 | 0.0093174 | HERC6 |
| TC12001751.hg.1 | -9.5137 | 0.00068142 | 3.1666 | 0.00941 | PHLDA1 |
| TC13000872.hg.1 | -9.5044 | 0.00068402 | 3.1649 | 0.0094199 | COL4A1 |
| TC11000580.hg.1 | -9.4968 | 0.00068612 | 3.1636 | 0.0094229 | RNU6-45P |
| TC02004166.hg.1 | 9.4704 | 0.00069353 | 3.1589 | 0.0094768 | NA |
| TC10001166.hg.1 | -9.4693 | 0.00069384 | 3.1587 | 0.0094768 | NRP1 |
| TC15002331.hg.1 | -9.4514 | 0.00069893 | 3.1556 | 0.0095108 | ADAMTSL3 |
| TC05001295.hg.1 | 9.4472 | 0.00070013 | 3.1548 | 0.0095108 | FYB |
| TC0X000987.hg.1 | -9.4389 | 0.00070249 | 3.1534 | 0.009517 | FLJ25917 |
| TC01000081.hg.1 | -9.4089 | 0.00071118 | 3.148 | 0.0096086 | AJAP1 |
| TC15001211.hg.1 | 9.3789 | 0.00071998 | 3.1427 | 0.0097013 | MEIS2 |
| TC02004489.hg.1 | 9.3541 | 0.00072738 | 3.1382 | 0.0097496 | NA |
| TC16000167.hg.1 | 9.3538 | 0.00072746 | 3.1382 | 0.0097496 | SNX29 |
| TC15000609.hg.1 | -9.3432 | 0.00073066 | 3.1363 | 0.0097569 | RAB11A |
| PSR11017832.hg.1 | -9.339 | 0.00073191 | 3.1355 | 0.0097569 | NA |
| TC04000839.hg.1 | -9.3141 | 0.00073949 | 3.1311 | 0.0098233 | PALLD |
| TC07003339.hg.1 | -9.3097 | 0.00074082 | 3.1303 | 0.0098233 | CAV1 |
| TC01002763.hg.1 | -9.2774 | 0.00075081 | 3.1245 | 0.0099262 | WLS |
| TC18000554.hg.1 | -9.2718 | 0.00075255 | 3.1235 | 0.0099262 | BCL2 |
| TC15001505.hg.1 | -9.236 | 0.00076387 | 3.117 | 0.010049 | ANXA2 |
| TC14001171.hg.1 | -9.2282 | 0.00076636 | 3.1156 | 0.010055 | C14orf105 |
| TC03002115.hg.1 | -9.2211 | 0.00076864 | 3.1143 | 0.010059 | FGF12 |
| TC16000127.hg.1 | 9.195 | 0.00077705 | 3.1095 | 0.01008 | DNASE1 |
| TC03001634.hg.1 | -9.1918 | 0.0007781 | 3.109 | 0.01008 | GUCA1C |
| TC07000301.hg.1 | 9.1899 | 0.00077873 | 3.1086 | 0.01008 | NA |
| TC10002589.hg.1 | -9.1776 | 0.00078274 | 3.1064 | 0.01008 | CXCL12 |
| TC01004026.hg.1 | -9.1696 | 0.00078536 | 3.1049 | 0.01008 | OPN3 |
| TC05000072.hg.1 | -9.1644 | 0.00078709 | 3.104 | 0.01008 | CCT5 |
| TC14000943.hg.1 | -9.153 | 0.00079086 | 3.1019 | 0.01008 | AJUBA |
| TC05001638.hg.1 | 9.1526 | 0.00079101 | 3.1018 | 0.01008 | ST8SIA4 |
| TC08001248.hg.1 | -9.1512 | 0.00079146 | 3.1016 | 0.01008 | TOX |
| TC13000701.hg.1 | -9.1482 | 0.00079245 | 3.101 | 0.01008 | DIAPH3 |
| TC03002313.hg.1 | -9.1458 | 0.00079326 | 3.1006 | 0.01008 | NA |
| TC06000135.hg.1 | -9.1421 | 0.0007945 | 3.0999 | 0.01008 | SOX4 |
| TC02004666.hg.1 | -9.0921 | 0.00081145 | 3.0907 | 0.010269 | MXRA7 |
| TC16001861.hg.1 | -9.0787 | 0.00081606 | 3.0883 | 0.010302 | NA |
| TC20000264.hg.1 | -9.0529 | 0.00082502 | 3.0835 | 0.010389 | EPB41L1 |
| TC10000621.hg.1 | -9.0231 | 0.00083556 | 3.078 | 0.010485 | PAPSS2 |
| TC03001720.hg.1 | -9.0153 | 0.00083833 | 3.0766 | 0.010485 | HEG1 |
| TC08001068.hg.1 | -9.0137 | 0.00083893 | 3.0763 | 0.010485 | NEFL |
| TC08000500.hg.1 | -9.0063 | 0.00084158 | 3.0749 | 0.010491 | LINC01111 |
| TC12001081.hg.1 | 8.9913 | 0.00084698 | 3.0721 | 0.010532 | LOC102723544 |
| TC04001227.hg.1 | -8.9719 | 0.00085402 | 3.0685 | 0.010589 | NA |
| TC17001157.hg.1 | -8.9541 | 0.00086056 | 3.0652 | 0.010589 | PMP22 |
| TC07000018.hg.1 | 8.9513 | 0.00086163 | 3.0647 | 0.010589 | GPER1 |
| TC02002179.hg.1 | 8.946 | 0.00086357 | 3.0637 | 0.010589 | NA |
| TC15000749.hg.1 | -8.9428 | 0.00086477 | 3.0631 | 0.010589 | ARNT2 |
| TC12003245.hg.1 | -8.938 | 0.00086653 | 3.0622 | 0.010589 | MYL6 |
| TC11003478.hg.1 | -8.9352 | 0.00086758 | 3.0617 | 0.010589 | SAA2 |
| TC04001655.hg.1 | -8.9317 | 0.00086889 | 3.061 | 0.010589 | ANXA2P1 |
| TC07001726.hg.1 | 8.9271 | 0.0008706 | 3.0602 | 0.010589 | LHFPL3-AS2 |
| TC11000754.hg.1 | -8.9064 | 0.00087839 | 3.0563 | 0.010658 | FOLR1 |
| TC01002452.hg.1 | -8.887 | 0.00088579 | 3.0527 | 0.010722 | FABP3 |
| TC02002823.hg.1 | -8.8741 | 0.00089075 | 3.0502 | 0.010748 | SERPINE2 |
| TC10001240.hg.1 | -8.8672 | 0.0008934 | 3.049 | 0.010748 | AGAP9 |
| TC03001128.hg.1 | -8.8647 | 0.00089438 | 3.0485 | 0.010748 | EGOT |
| TC02001486.hg.1 | 8.8257 | 0.00090964 | 3.0411 | 0.010905 | D2HGDH |
| TC09000052.hg.1 | -8.8183 | 0.00091256 | 3.0397 | 0.010914 | NA |
| TC02000952.hg.1 | -8.8128 | 0.00091478 | 3.0387 | 0.010914 | GPD2 |
| TC12001904.hg.1 | -8.801 | 0.00091948 | 3.0365 | 0.010927 | GLT8D2 |
| TC20000072.hg.1 | -8.7933 | 0.00092256 | 3.035 | 0.010927 | PLCB1 |
| TC13001557.hg.1 | -8.7899 | 0.00092394 | 3.0344 | 0.010927 | NA |
| TC09001618.hg.1 | 8.7873 | 0.00092497 | 3.0339 | 0.010927 | FAM102A |
| TC02002605.hg.1 | -8.7828 | 0.00092682 | 3.033 | 0.010927 | CALCRL |
| TC12001786.hg.1 | 8.7567 | 0.00093745 | 3.0281 | 0.011027 | RASSF9 |
| TC15001837.hg.1 | -8.7418 | 0.00094357 | 3.0252 | 0.011073 | ANPEP |
| TC15000045.hg.1 | -8.7318 | 0.00094774 | 3.0233 | 0.011095 | SNORD108 |
| TC10002529.hg.1 | -8.7183 | 0.00095337 | 3.0207 | 0.011135 | NA |
| TC19001275.hg.1 | -8.705 | 0.00095895 | 3.0182 | 0.011145 | BST2 |
| TC03003007.hg.1 | -8.7048 | 0.00095904 | 3.0182 | 0.011145 | NA |
| TC03001527.hg.1 | 8.7004 | 0.00096089 | 3.0173 | 0.011145 | ADAMTS9 |
| TC09000922.hg.1 | -8.6859 | 0.00096708 | 3.0145 | 0.011189 | NA |
| TC07001205.hg.1 | 8.681 | 0.00096918 | 3.0136 | 0.011189 | DFNA5 |
| TC12001391.hg.1 | -8.6682 | 0.00097464 | 3.0112 | 0.011208 | KIF21A |
| TC10000637.hg.1 | -8.6668 | 0.00097528 | 3.0109 | 0.011208 | IFIT3 |
| TC21000185.hg.1 | 8.5993 | 0.0010049 | 2.9979 | 0.011522 | BACE2 |
| TC01006309.hg.1 | -8.5919 | 0.0010082 | 2.9965 | 0.011533 | NBPF9 |
| TC05002434.hg.1 | -8.5852 | 0.0010113 | 2.9951 | 0.011542 | MAP1B |
| TC18000047.hg.1 | -8.5757 | 0.0010155 | 2.9933 | 0.011564 | RAB31 |
| TC04000916.hg.1 | 8.5704 | 0.0010179 | 2.9923 | 0.011565 | F11 |
| TC15002504.hg.1 | 8.5272 | 0.0010378 | 2.9839 | 0.011764 | PLA2G4F |
| TC09000323.hg.1 | -8.5216 | 0.0010404 | 2.9828 | 0.011767 | MAMDC2 |
| TC03002782.hg.1 | -8.5131 | 0.0010444 | 2.9811 | 0.011785 | NA |
| TC05002857.hg.1 | -8.4901 | 0.0010553 | 2.9766 | 0.011881 | SEMA5A |
| TC04002792.hg.1 | -8.4766 | 0.0010617 | 2.974 | 0.011919 | ANXA2P1 |
| TC12001657.hg.1 | -8.4732 | 0.0010633 | 2.9733 | 0.011919 | PPM1H |
| TC19002032.hg.1 | -8.4658 | 0.0010669 | 2.9719 | 0.011932 | ZNF90 |
| TC01000987.hg.1 | -8.439 | 0.0010799 | 2.9666 | 0.01205 | CTTNBP2NL |
| PSR11023322.hg.1 | -8.4286 | 0.001085 | 2.9646 | 0.01208 | NA |
| TC02004761.hg.1 | -8.4061 | 0.0010962 | 2.9601 | 0.012177 | ANKRD44 |
| TC07003035.hg.1 | -8.3921 | 0.0011031 | 2.9574 | 0.012227 | FGL2 |
| TC11002773.hg.1 | -8.3834 | 0.0011075 | 2.9556 | 0.012249 | FOLR1 |
| TC08000250.hg.1 | -8.3678 | 0.0011155 | 2.9525 | 0.01229 | NRG1 |
| TC04000887.hg.1 | -8.3626 | 0.0011181 | 2.9515 | 0.01229 | WWC2 |
| TC01002884.hg.1 | -8.3598 | 0.0011195 | 2.951 | 0.01229 | ARHGAP29 |
| TC20000109.hg.1 | -8.3538 | 0.0011226 | 2.9498 | 0.01229 | DSTN |
| TC01005354.hg.1 | 8.3521 | 0.0011235 | 2.9494 | 0.01229 | SLC9A1 |
| TC05001733.hg.1 | -8.3374 | 0.0011311 | 2.9465 | 0.012337 | NA |
| TC22000329.hg.1 | -8.3341 | 0.0011327 | 2.9459 | 0.012337 | NA |
| TC13000346.hg.1 | -8.2976 | 0.0011519 | 2.9386 | 0.012518 | FARP1 |
| TC01005141.hg.1 | -8.2663 | 0.0011687 | 2.9323 | 0.012624 | CHRM3 |
| TC22000058.hg.1 | 8.266 | 0.0011688 | 2.9323 | 0.012624 | LOC440792 |
| TC01004328.hg.1 | -8.2628 | 0.0011705 | 2.9316 | 0.012624 | NA |
| TC02003993.hg.1 | -8.2606 | 0.0011717 | 2.9312 | 0.012624 | NA |
| TC04001570.hg.1 | 8.2429 | 0.0011813 | 2.9276 | 0.012693 | SLC7A11 |
| TC07001979.hg.1 | -8.2395 | 0.0011832 | 2.9269 | 0.012693 | OR2A7 |
| TC01006085.hg.1 | -8.2239 | 0.0011918 | 2.9238 | 0.012757 | KCNH1 |
| TC02000466.hg.1 | 8.2107 | 0.0011991 | 2.9211 | 0.012788 | HK2 |
| TC03001719.hg.1 | 8.2095 | 0.0011998 | 2.9209 | 0.012788 | MUC13 |
| TC12001277.hg.1 | 8.1856 | 0.0012132 | 2.9161 | 0.012904 | ERP27 |
| TC07003151.hg.1 | -8.1748 | 0.0012193 | 2.9139 | 0.012941 | GRM8 |
| TC07001355.hg.1 | 8.158 | 0.0012289 | 2.9105 | 0.013016 | IGFBP3 |
| TC04000440.hg.1 | -8.1363 | 0.0012414 | 2.9061 | 0.01312 | BMP2K |
| TC13000803.hg.1 | 8.1092 | 0.0012573 | 2.9005 | 0.01326 | CLDN10-AS1 |
| TC12000182.hg.1 | -8.08 | 0.0012747 | 2.8946 | 0.013393 | GPRC5A |
| TC0X000296.hg.1 | -8.079 | 0.0012753 | 2.8944 | 0.013393 | MAGED1 |
| TC03002464.hg.1 | -8.0666 | 0.0012828 | 2.8918 | 0.013421 | PHLDB2 |
| TC03000806.hg.1 | 8.0658 | 0.0012833 | 2.8917 | 0.013421 | TM4SF4 |
| TC01003614.hg.1 | 8.0447 | 0.0012961 | 2.8874 | 0.013527 | NMNAT2 |
| TC01004716.hg.1 | -8.0229 | 0.0013096 | 2.8829 | 0.013629 | NA |
| TC01006368.hg.1 | -8.0152 | 0.0013144 | 2.8813 | 0.013629 | NBPF14 |
| TC09000899.hg.1 | -8.0117 | 0.0013165 | 2.8806 | 0.013629 | GLDC |
| TC02001126.hg.1 | -8.0112 | 0.0013169 | 2.8805 | 0.013629 | MYO1B |
| TC01000729.hg.1 | -8.0007 | 0.0013234 | 2.8783 | 0.013655 | DNAJC6 |
| TC15002091.hg.1 | -7.9986 | 0.0013248 | 2.8779 | 0.013655 | PWAR5 |
| TC05002526.hg.1 | -7.994 | 0.0013277 | 2.8769 | 0.013656 | CAST |
| TC19002569.hg.1 | -7.9836 | 0.0013343 | 2.8747 | 0.013696 | NA |
| TC14001509.hg.1 | 7.9579 | 0.0013507 | 2.8694 | 0.013837 | WARS |
| TC02002747.hg.1 | -7.9457 | 0.0013587 | 2.8669 | 0.013889 | FN1 |
| 3475646_st | -7.9279 | 0.0013703 | 2.8632 | 0.013979 | NA |
| TC03001489.hg.1 | -7.8751 | 0.0014055 | 2.8522 | 0.014309 | IL17RD |
| TC12002221.hg.1 | 7.8707 | 0.0014085 | 2.8512 | 0.014311 | NA |
| TC17002615.hg.1 | 7.858 | 0.0014172 | 2.8486 | 0.014365 | NR1D1 |
| TC0X000025.hg.1 | -7.8545 | 0.0014195 | 2.8479 | 0.014365 | STS |
| TC06001226.hg.1 | -7.8493 | 0.0014231 | 2.8468 | 0.014372 | TUBB2A |
| TC0X001353.hg.1 | -7.8361 | 0.0014322 | 2.844 | 0.014435 | SMARCA1 |
| TC02004669.hg.1 | -7.8314 | 0.0014355 | 2.843 | 0.014439 | NA |
| TC18000375.hg.1 | -7.8152 | 0.0014468 | 2.8396 | 0.014524 | NA |
| TC15002503.hg.1 | -7.7747 | 0.0014757 | 2.831 | 0.014778 | EHD4 |
| TC22000153.hg.1 | 7.7713 | 0.0014781 | 2.8303 | 0.014778 | BCRP3 |
| TC11002370.hg.1 | -7.7129 | 0.001521 | 2.8179 | 0.015176 | USP2 |
| TC17001353.hg.1 | -7.6917 | 0.0015369 | 2.8134 | 0.015304 | MYO1D |
| TC05001989.hg.1 | -7.6867 | 0.0015407 | 2.8123 | 0.015312 | CLINT1 |
| TC02001100.hg.1 | -7.6799 | 0.0015458 | 2.8108 | 0.015333 | ITGAV |
| TC04001487.hg.1 | -7.6676 | 0.0015553 | 2.8082 | 0.015396 | ARSJ |
| TC09002706.hg.1 | -7.6586 | 0.0015622 | 2.8063 | 0.015434 | BAAT |
| TC08000698.hg.1 | 7.6335 | 0.0015818 | 2.8009 | 0.015596 | DEPTOR |
| TC10001754.hg.1 | 7.6239 | 0.0015893 | 2.7988 | 0.015639 | CLRN3 |
| TC03001018.hg.1 | -7.5999 | 0.0016084 | 2.7936 | 0.015782 | DNAJB11 |
| TC01001847.hg.1 | 7.5977 | 0.0016101 | 2.7931 | 0.015782 | CNIH4 |
| TC0X001073.hg.1 | -7.5828 | 0.0016221 | 2.7899 | 0.015866 | WNK3 |
| TC21000342.hg.1 | -7.5792 | 0.001625 | 2.7891 | 0.015866 | NA |
| TC11002226.hg.1 | -7.5608 | 0.0016401 | 2.7851 | 0.015979 | TMEM123 |
| TC21000719.hg.1 | -7.5558 | 0.0016442 | 2.7841 | 0.015979 | NA |
| TC06001260.hg.1 | 7.5534 | 0.0016461 | 2.7835 | 0.015979 | TFAP2A |
| TC13000295.hg.1 | -7.5201 | 0.0016739 | 2.7763 | 0.016217 | SCEL |
| TC08001639.hg.1 | -7.5104 | 0.0016821 | 2.7742 | 0.016264 | NA |
| TC06002710.hg.1 | 7.5046 | 0.001687 | 2.7729 | 0.016281 | NA |
| TC08000782.hg.1 | -7.4792 | 0.0017087 | 2.7673 | 0.016458 | NA |
| TC12002282.hg.1 | 7.4652 | 0.0017208 | 2.7643 | 0.016507 | SLCO1B3 |
| TC13001641.hg.1 | 7.4643 | 0.0017217 | 2.7641 | 0.016507 | CLDN10-AS1 |
| TC10001069.hg.1 | -7.462 | 0.0017236 | 2.7636 | 0.016507 | RSU1 |
| TC12000363.hg.1 | -7.4516 | 0.0017327 | 2.7613 | 0.016562 | NA |
| TC05000515.hg.1 | 7.4322 | 0.0017499 | 2.757 | 0.016694 | MAN2A1 |
| TC0X000851.hg.1 | -7.4248 | 0.0017565 | 2.7554 | 0.016725 | GPR143 |
| TC19002056.hg.1 | -7.4138 | 0.0017663 | 2.7529 | 0.016787 | NA |
| TC07000881.hg.1 | -7.3894 | 0.0017884 | 2.7475 | 0.016964 | RAB19 |
| TC18000141.hg.1 | -7.3802 | 0.0017969 | 2.7455 | 0.017012 | DTNA |
| TC13000037.hg.1 | -7.3759 | 0.0018008 | 2.7445 | 0.017017 | FGF9 |
| TC01006430.hg.1 | -7.3658 | 0.0018101 | 2.7423 | 0.017067 | NBPF9 |
| TC04002009.hg.1 | -7.3598 | 0.0018157 | 2.741 | 0.017067 | NA |
| TC01000580.hg.1 | -7.3591 | 0.0018163 | 2.7408 | 0.017067 | TSPAN1 |
| TC01003210.hg.1 | -7.3452 | 0.0018293 | 2.7377 | 0.017133 | CTSS |
| TC08002496.hg.1 | 7.3443 | 0.0018302 | 2.7375 | 0.017133 | NA |
| TC09002110.hg.1 | -7.3401 | 0.0018341 | 2.7366 | 0.017138 | NA |
| TC06001860.hg.1 | -7.3303 | 0.0018434 | 2.7344 | 0.017193 | LINC00472 |
| TC10002411.hg.1 | -7.3125 | 0.0018603 | 2.7304 | 0.017291 | KLF6 |
| TC02004874.hg.1 | 7.3119 | 0.0018609 | 2.7303 | 0.017291 | RAB17 |
| TC04002457.hg.1 | -7.2923 | 0.0018798 | 2.7259 | 0.017435 | WDR1 |
| TC04001196.hg.1 | -7.2885 | 0.0018836 | 2.725 | 0.017437 | ERVMER34-1 |
| TC14001387.hg.1 | -7.2815 | 0.0018904 | 2.7234 | 0.017468 | GALC |
| TC01000493.hg.1 | -7.2457 | 0.0019258 | 2.7154 | 0.017721 | MACF1 |
| TC19001347.hg.1 | -7.2449 | 0.0019265 | 2.7152 | 0.017721 | ZNF737 |
| TC02004487.hg.1 | 7.243 | 0.0019285 | 2.7148 | 0.017721 | NA |
| TC04001718.hg.1 | -7.2247 | 0.0019469 | 2.7106 | 0.017858 | DDX60 |
| TC07003312.hg.1 | -7.221 | 0.0019507 | 2.7098 | 0.01786 | CCDC146 |
| TC10001255.hg.1 | -7.2097 | 0.0019622 | 2.7073 | 0.017932 | AGAP4 |
| PSR11023321.hg.1 | -7.1987 | 0.0019735 | 2.7048 | 0.018003 | NA |
| TC17001644.hg.1 | -7.1852 | 0.0019875 | 2.7017 | 0.018097 | HOXB5 |
| TC06003713.hg.1 | -7.1714 | 0.0020019 | 2.6986 | 0.018195 | NA |
| 3597903_st | -7.1657 | 0.0020079 | 2.6973 | 0.018217 | NA |
| 3607147_st | -7.1617 | 0.0020121 | 2.6964 | 0.018222 | NA |
| TC16000045.hg.1 | 7.1542 | 0.00202 | 2.6947 | 0.01826 | MAPK8IP3 |
| TC08000209.hg.1 | -7.1425 | 0.0020325 | 2.692 | 0.01834 | SCARA3 |
| TC15001140.hg.1 | 7.1307 | 0.0020451 | 2.6893 | 0.018421 | GOLGA8R |
| TC16000501.hg.1 | -7.1101 | 0.0020675 | 2.6846 | 0.018589 | CCDC113 |
| TC10001212.hg.1 | -7.0939 | 0.0020853 | 2.6808 | 0.018697 | CXCL12 |
| TC20000337.hg.1 | -7.0922 | 0.0020872 | 2.6804 | 0.018697 | YWHAB |
| TC0X001279.hg.1 | -7.0889 | 0.0020908 | 2.6797 | 0.018697 | CAPN6 |
| TC17001868.hg.1 | -7.0841 | 0.0020962 | 2.6786 | 0.018712 | NA |
| TC06001337.hg.1 | -7.0702 | 0.0021116 | 2.6754 | 0.018816 | SLC17A1 |
| TC12002166.hg.1 | -7.0668 | 0.0021154 | 2.6746 | 0.018816 | WASH1 |
| TC03001023.hg.1 | -7.0613 | 0.0021216 | 2.6733 | 0.018838 | HRG |
| TC01005350.hg.1 | -7.0573 | 0.0021261 | 2.6724 | 0.018844 | STMN1 |
| TC12002866.hg.1 | -7.051 | 0.0021333 | 2.671 | 0.018852 | NA |
| TC04002890.hg.1 | -7.0499 | 0.0021345 | 2.6707 | 0.018852 | FAT1 |
| TC15000259.hg.1 | -7.0377 | 0.0021484 | 2.6679 | 0.018942 | SPRED1 |
| TC05000243.hg.1 | -7.0317 | 0.0021553 | 2.6665 | 0.018969 | MAP3K1 |
| TC03001915.hg.1 | 7.0193 | 0.0021696 | 2.6636 | 0.019061 | LOC105374165 |
| TC02002474.hg.1 | -7.0035 | 0.002188 | 2.6599 | 0.019162 | MXRA7 |
| PSR01006340.hg.1 | -7.0006 | 0.0021914 | 2.6593 | 0.019162 | NA |
| TC06002043.hg.1 | -6.9996 | 0.0021926 | 2.659 | 0.019162 | FRK |
| TC07001214.hg.1 | -6.9949 | 0.0021982 | 2.6579 | 0.019177 | HNRNPA2B1 |
| TC02002832.hg.1 | 6.9834 | 0.0022117 | 2.6553 | 0.019258 | COL4A4 |
| TC17002825.hg.1 | -6.9805 | 0.0022151 | 2.6546 | 0.019258 | ACTG1 |
| TC16002069.hg.1 | -6.9767 | 0.0022197 | 2.6537 | 0.019265 | QPRT |
| TC15000181.hg.1 | 6.9564 | 0.002244 | 2.649 | 0.019442 | GOLGA8Q |
| TC16001579.hg.1 | -6.9524 | 0.0022489 | 2.648 | 0.01945 | NA |
| TC13000198.hg.1 | 6.9456 | 0.0022571 | 2.6465 | 0.019487 | SETDB2 |
| TC06001971.hg.1 | -6.9387 | 0.0022655 | 2.6448 | 0.019526 | NA |
| TC0Y000187.hg.1 | 6.9124 | 0.0022979 | 2.6387 | 0.019746 | NA |
| TC08002065.hg.1 | -6.9117 | 0.0022989 | 2.6385 | 0.019746 | ENY2 |
| TC08001455.hg.1 | -6.9009 | 0.0023123 | 2.6359 | 0.019794 | TSPYL5 |
| TC03000234.hg.1 | -6.8983 | 0.0023156 | 2.6353 | 0.019794 | NA |
| TC13001534.hg.1 | -6.8977 | 0.0023163 | 2.6352 | 0.019794 | DIAPH3 |
| TC08001921.hg.1 | -6.8919 | 0.0023236 | 2.6338 | 0.019807 | NA |
| TC04002747.hg.1 | -6.8902 | 0.0023258 | 2.6334 | 0.019807 | NA |
| TC14000056.hg.1 | 6.8816 | 0.0023367 | 2.6314 | 0.019866 | PNP |
| TC6_cox_hap2000061.hg.1 | 6.8649 | 0.0023581 | 2.6274 | 0.01999 | HCP5 |
| TC15001476.hg.1 | -6.8639 | 0.0023593 | 2.6272 | 0.01999 | MNS1 |
| TC01002240.hg.1 | -6.8593 | 0.0023652 | 2.6261 | 0.020006 | TMEM51-AS1 |
| TC04000065.hg.1 | 6.8526 | 0.0023738 | 2.6245 | 0.020045 | EVC |
| TC09001204.hg.1 | -6.8364 | 0.0023949 | 2.6207 | 0.020189 | TMEM2 |
| TC02000230.hg.1 | -6.8316 | 0.0024013 | 2.6196 | 0.020209 | CRIM1 |
| TC05001182.hg.1 | -6.8204 | 0.002416 | 2.6169 | 0.020299 | FAM134B |
| TC01004893.hg.1 | -6.8043 | 0.0024375 | 2.6131 | 0.020389 | NA |
| TC02001072.hg.1 | -6.7994 | 0.002444 | 2.6119 | 0.020389 | LOC101927027 |
| TC08002467.hg.1 | -6.7993 | 0.0024441 | 2.6119 | 0.020389 | NA |
| TC02002908.hg.1 | 6.795 | 0.0024499 | 2.6108 | 0.020389 | RAB17 |
| TC19001375.hg.1 | 6.7906 | 0.0024558 | 2.6098 | 0.020389 | NA |
| TC07000055.hg.1 | -6.7894 | 0.0024575 | 2.6095 | 0.020389 | FSCN1 |
| TC07001606.hg.1 | -6.7881 | 0.0024593 | 2.6092 | 0.020389 | SAMD9L |
| TC10000635.hg.1 | -6.788 | 0.0024594 | 2.6092 | 0.020389 | NA |
| TC18000128.hg.1 | -6.7707 | 0.0024829 | 2.605 | 0.02055 | DSG2 |
| TC11003006.hg.1 | -6.7668 | 0.0024883 | 2.6041 | 0.02056 | KCNQ1OT1 |
| TC16000650.hg.1 | -6.7611 | 0.0024961 | 2.6027 | 0.020591 | HSBP1 |
| TC16001124.hg.1 | 6.7526 | 0.0025079 | 2.6007 | 0.020654 | IRX3 |
| TC0X000207.hg.1 | 6.7475 | 0.0025149 | 2.5995 | 0.020678 | MAOA |
| TC09000696.hg.1 | -6.7303 | 0.0025389 | 2.5953 | 0.020841 | SET |
| TC16001125.hg.1 | 6.7263 | 0.0025446 | 2.5944 | 0.020853 | CRNDE |
| TC08002539.hg.1 | -6.7189 | 0.002555 | 2.5926 | 0.020873 | ST3GAL1 |
| TC01001364.hg.1 | -6.7187 | 0.0025554 | 2.5925 | 0.020873 | PEA15 |
| PSR14009915.hg.1 | 6.7122 | 0.0025646 | 2.591 | 0.020914 | NA |
| TC11000951.hg.1 | -6.704 | 0.0025763 | 2.589 | 0.020975 | C11orf70 |
| TC15001257.hg.1 | -6.6933 | 0.0025916 | 2.5864 | 0.021066 | EHD4 |
| TC09000535.hg.1 | 6.687 | 0.0026008 | 2.5849 | 0.021106 | SLC44A1 |
| TC10000363.hg.1 | -6.6827 | 0.002607 | 2.5839 | 0.021122 | FAM133CP |
| TC02002616.hg.1 | 6.676 | 0.0026168 | 2.5822 | 0.021167 | ORMDL1 |
| TC19002254.hg.1 | -6.6602 | 0.00264 | 2.5784 | 0.021309 | ZNF667-AS1 |
| TC02001356.hg.1 | 6.6582 | 0.0026428 | 2.5779 | 0.021309 | NA |
| TC08000648.hg.1 | -6.6541 | 0.002649 | 2.5769 | 0.021324 | DCAF13 |
| TC01002791.hg.1 | 6.638 | 0.002673 | 2.573 | 0.02148 | SLC44A5 |
| TC06001126.hg.1 | -6.6354 | 0.0026769 | 2.5724 | 0.02148 | SNX9 |
| TC17001485.hg.1 | -6.6317 | 0.0026824 | 2.5715 | 0.021489 | KRTAP2-3 |
| TC02004919.hg.1 | 6.6262 | 0.0026907 | 2.5701 | 0.021521 | HOXD10 |
| TC05001977.hg.1 | -6.606 | 0.0027214 | 2.5652 | 0.021709 | HAVCR1 |
| TC09002547.hg.1 | -6.6051 | 0.0027228 | 2.565 | 0.021709 | NA |
| TC0X001486.hg.1 | -6.5935 | 0.0027407 | 2.5621 | 0.021816 | CD99L2 |
| TC21000717.hg.1 | -6.5666 | 0.0027826 | 2.5555 | 0.022021 | KCNJ15 |
| TC06000658.hg.1 | -6.563 | 0.0027883 | 2.5547 | 0.022021 | TMEM14A |
| TC06000231.hg.1 | -6.563 | 0.0027884 | 2.5546 | 0.022021 | NA |
| TC06001391.hg.1 | -6.563 | 0.0027884 | 2.5546 | 0.022021 | NA |
| TC06001412.hg.1 | -6.563 | 0.0027884 | 2.5546 | 0.022021 | NA |
| TC10001732.hg.1 | -6.549 | 0.0028105 | 2.5512 | 0.022161 | CTBP2 |
| TC15000635.hg.1 | 6.5332 | 0.0028359 | 2.5473 | 0.022307 | PAQR5 |
| TC12003238.hg.1 | -6.5318 | 0.0028381 | 2.547 | 0.022307 | DDX47 |
| TC01001950.hg.1 | -6.5229 | 0.0028526 | 2.5448 | 0.022386 | GPR137B |
| TC02002645.hg.1 | -6.5183 | 0.00286 | 2.5436 | 0.022409 | ANKRD44 |
| TC03001629.hg.1 | -6.5086 | 0.0028758 | 2.5412 | 0.022486 | CD47 |
| TC04000798.hg.1 | -6.5068 | 0.0028788 | 2.5408 | 0.022486 | GLRB |
| TC05000497.hg.1 | -6.4925 | 0.0029024 | 2.5372 | 0.022635 | PAM |
| TC08000980.hg.1 | -6.4898 | 0.0029069 | 2.5366 | 0.022635 | CTSB |
| TC06000347.hg.1 | -6.4838 | 0.002917 | 2.5351 | 0.022678 | TUBB |
| TC10002626.hg.1 | -6.4808 | 0.0029219 | 2.5343 | 0.022681 | NA |
| TC02002733.hg.1 | 6.4615 | 0.0029544 | 2.5295 | 0.022886 | LANCL1 |
| TC12000276.hg.1 | -6.4597 | 0.0029574 | 2.5291 | 0.022886 | FAR2 |
| TC12001470.hg.1 | -6.4549 | 0.0029656 | 2.5279 | 0.022914 | TUBA1B |
| TC09000568.hg.1 | -6.4504 | 0.0029733 | 2.5268 | 0.022914 | SNX30 |
| TC19001370.hg.1 | -6.4489 | 0.0029759 | 2.5264 | 0.022914 | ZNF681 |
| TC0X001188.hg.1 | -6.4468 | 0.0029794 | 2.5259 | 0.022914 | HDX |
| TC03001884.hg.1 | 6.437 | 0.0029962 | 2.5234 | 0.023004 | CP |
| TC08000575.hg.1 | 6.4323 | 0.0030045 | 2.5222 | 0.023004 | ESRP1 |
| TC01001974.hg.1 | -6.432 | 0.003005 | 2.5222 | 0.023004 | KMO |
| TC02003715.hg.1 | -6.4277 | 0.0030123 | 2.5211 | 0.023025 | NA |
| TC07002874.hg.1 | -6.4034 | 0.003055 | 2.515 | 0.023316 | PDE1C |
| TC06002630.hg.1 | -6.3763 | 0.0031032 | 2.5082 | 0.023601 | NA |
| PSR11017828.hg.1 | -6.3749 | 0.0031058 | 2.5078 | 0.023601 | NA |
| TC17000042.hg.1 | -6.3739 | 0.0031076 | 2.5076 | 0.023601 | SPNS2 |
| TC03001700.hg.1 | -6.3719 | 0.0031112 | 2.5071 | 0.023601 | ILDR1 |
| TC01002050.hg.1 | -6.3594 | 0.0031339 | 2.5039 | 0.023737 | WASH7P |
| TC14002183.hg.1 | 6.35 | 0.0031511 | 2.5015 | 0.023825 | AHNAK2 |
| TC08000391.hg.1 | -6.3443 | 0.0031616 | 2.5001 | 0.023825 | LINC01606 |
| TC12000399.hg.1 | 6.3431 | 0.0031638 | 2.4998 | 0.023825 | METTL7A |
| TC06003758.hg.1 | -6.3422 | 0.0031654 | 2.4996 | 0.023825 | CNR1 |
| TC10000824.hg.1 | -6.3401 | 0.0031693 | 2.499 | 0.023825 | HABP2 |
| PSR11023323.hg.1 | -6.3237 | 0.0031998 | 2.4949 | 0.024018 | NA |
| TC04001769.hg.1 | -6.3189 | 0.0032088 | 2.4937 | 0.024049 | AGA |
| TC01001790.hg.1 | -6.3115 | 0.0032227 | 2.4918 | 0.024117 | SMYD2 |
| TC08001275.hg.1 | -6.3087 | 0.003228 | 2.4911 | 0.024121 | CYP7B1 |
| TC01002708.hg.1 | -6.3055 | 0.003234 | 2.4903 | 0.024129 | JUN |
| JUC11012306.hg.1 | -6.299 | 0.0032464 | 2.4886 | 0.02417 | NA |
| TC12002700.hg.1 | 6.2976 | 0.003249 | 2.4882 | 0.02417 | NA |
| TC01004896.hg.1 | -6.2915 | 0.0032608 | 2.4867 | 0.024206 | GS1-204I12.4 |
| TC04000140.hg.1 | -6.29 | 0.0032637 | 2.4863 | 0.024206 | CPEB2 |
| PSR03025024.hg.1 | -6.2856 | 0.0032721 | 2.4852 | 0.024211 | NA |
| TC05002544.hg.1 | -6.2846 | 0.003274 | 2.4849 | 0.024211 | PAM |
| TC09001930.hg.1 | 6.2754 | 0.0032917 | 2.4826 | 0.024306 | NA |
| TC01005732.hg.1 | -6.263 | 0.0033158 | 2.4794 | 0.024419 | CD58 |
| TC01006110.hg.1 | 6.262 | 0.0033177 | 2.4792 | 0.024419 | ESRRG |
| TC11001173.hg.1 | 6.2601 | 0.0033216 | 2.4787 | 0.024419 | DCPS |
| TC03001892.hg.1 | -6.2532 | 0.003335 | 2.4769 | 0.024471 | COMMD2 |
| TC06003935.hg.1 | -6.2514 | 0.0033386 | 2.4764 | 0.024471 | NA |
| TC02003402.hg.1 | -6.2434 | 0.0033545 | 2.4744 | 0.024471 | NA |
| TC22000034.hg.1 | 6.243 | 0.0033552 | 2.4743 | 0.024471 | NA |
| TC07003069.hg.1 | -6.2418 | 0.0033575 | 2.474 | 0.024471 | PON2 |
| TC12002014.hg.1 | 6.2415 | 0.0033581 | 2.4739 | 0.024471 | NOS1 |
| PSR11023319.hg.1 | -6.2285 | 0.003384 | 2.4706 | 0.024624 | NA |
| TC01000309.hg.1 | -6.2198 | 0.0034016 | 2.4683 | 0.024716 | GRHL3 |
| TC05003098.hg.1 | -6.2034 | 0.0034348 | 2.4641 | 0.024893 | NA |
| TC06002110.hg.1 | -6.2028 | 0.003436 | 2.4639 | 0.024893 | OR2A4 |
| TC11000910.hg.1 | -6.1993 | 0.0034432 | 2.463 | 0.02491 | SCARNA9 |
| TC15000391.hg.1 | -6.1921 | 0.0034581 | 2.4612 | 0.024977 | SLC27A2 |
| 3090556_st | -6.1899 | 0.0034625 | 2.4606 | 0.024977 | NA |
| TC18000447.hg.1 | 6.1724 | 0.0034989 | 2.4561 | 0.025203 | KLHL14 |
| TC02000854.hg.1 | 6.1648 | 0.0035147 | 2.4541 | 0.025243 | LOC150776 |
| TC12000010.hg.1 | -6.1641 | 0.0035161 | 2.4539 | 0.025243 | WNK1 |
| TC10002082.hg.1 | -6.1625 | 0.0035196 | 2.4535 | 0.025243 | NA |
| TC22001267.hg.1 | 6.1583 | 0.0035285 | 2.4524 | 0.02527 | NA |
| TC16002043.hg.1 | -6.1532 | 0.0035391 | 2.4511 | 0.02531 | NPIPA1 |
| TC16000095.hg.1 | -6.148 | 0.0035502 | 2.4497 | 0.025354 | TNFRSF12A |
| TC02002180.hg.1 | 6.1428 | 0.0035613 | 2.4484 | 0.025396 | LINC01594 |
| TC08002271.hg.1 | -6.1289 | 0.003591 | 2.4448 | 0.025572 | DUSP4 |
| TC06002412.hg.1 | -6.1237 | 0.0036022 | 2.4434 | 0.025586 | NA |
| TC17001723.hg.1 | 6.1233 | 0.0036032 | 2.4433 | 0.025586 | SRSF1 |
| TC16000230.hg.1 | 6.1182 | 0.0036142 | 2.442 | 0.025603 | SNX29P1 |
| TC09000449.hg.1 | -6.1174 | 0.0036158 | 2.4418 | 0.025603 | NA |
| TC05000218.hg.1 | -6.1075 | 0.0036375 | 2.4392 | 0.02572 | ITGA2 |
| TC06004140.hg.1 | -6.0992 | 0.0036557 | 2.437 | 0.025812 | CNKSR3 |
| TC17000061.hg.1 | -6.0949 | 0.0036652 | 2.4359 | 0.025842 | ZFP3 |
| TC08000699.hg.1 | 6.0884 | 0.0036797 | 2.4342 | 0.025872 | NA |
| TC16000548.hg.1 | -6.0881 | 0.0036803 | 2.4341 | 0.025872 | NUTF2 |
| TC01005389.hg.1 | -6.086 | 0.0036849 | 2.4336 | 0.025872 | BSDC1 |
| TC09002250.hg.1 | -6.0822 | 0.0036933 | 2.4326 | 0.025895 | MIR181A2HG |
| TC04002701.hg.1 | -6.0786 | 0.0037016 | 2.4316 | 0.025895 | NA |
| TC12000663.hg.1 | 6.0776 | 0.0037037 | 2.4314 | 0.025895 | NA |
| TC05002528.hg.1 | -6.0712 | 0.0037182 | 2.4297 | 0.02596 | CAST |
| TC10002081.hg.1 | -6.063 | 0.0037366 | 2.4275 | 0.025998 | NA |
| TC04002147.hg.1 | -6.0618 | 0.0037392 | 2.4272 | 0.025998 | NA |
| TC19000356.hg.1 | 6.0618 | 0.0037393 | 2.4272 | 0.025998 | GDF15 |
| TC15001922.hg.1 | -6.0575 | 0.003749 | 2.4261 | 0.026026 | NR2F2-AS1 |
| TC0M000011.hg.1 | 6.0554 | 0.0037537 | 2.4255 | 0.026026 | NA |
| TC03000625.hg.1 | -6.0474 | 0.003772 | 2.4234 | 0.026084 | SLC15A2 |
| TC12001513.hg.1 | -6.0464 | 0.0037743 | 2.4232 | 0.026084 | KRT80 |
| 3607151_st | -6.045 | 0.0037776 | 2.4228 | 0.026084 | NA |
| PSR01006342.hg.1 | -6.0388 | 0.0037917 | 2.4212 | 0.02613 | NA |
| TC06002116.hg.1 | 6.0371 | 0.0037957 | 2.4207 | 0.02613 | TAAR3 |
| TC18000017.hg.1 | -6.0352 | 0.0038001 | 2.4202 | 0.02613 | MYL12B |
| TC03002038.hg.1 | -6.0321 | 0.0038072 | 2.4194 | 0.026143 | GNB4 |
| TC22001027.hg.1 | 6.0278 | 0.0038173 | 2.4182 | 0.026177 | BCRP3 |
| TC04000182.hg.1 | -6.0244 | 0.0038251 | 2.4174 | 0.026194 | TBC1D19 |
| TC02003159.hg.1 | -6.0216 | 0.0038317 | 2.4166 | 0.026204 | NA |
| TC07001120.hg.1 | -6.0192 | 0.0038374 | 2.416 | 0.026206 | ACTB |
| TC09000347.hg.1 | 6.0045 | 0.0038719 | 2.4121 | 0.026406 | PCSK5 |
| TC09000278.hg.1 | -5.9997 | 0.0038832 | 2.4108 | 0.026447 | NA |
| TC02001737.hg.1 | -5.9957 | 0.0038927 | 2.4097 | 0.026476 | NA |
| TC03002106.hg.1 | -5.9644 | 0.0039681 | 2.4014 | 0.026889 | P3H2 |
| TC14002338.hg.1 | 5.9614 | 0.0039754 | 2.4006 | 0.026889 | PPP1R3E |
| TC20000336.hg.1 | -5.9609 | 0.0039767 | 2.4005 | 0.026889 | KCNK15 |
| TC12000890.hg.1 | 5.9608 | 0.0039769 | 2.4005 | 0.026889 | TPCN1 |
| TC06003852.hg.1 | 5.958 | 0.0039838 | 2.3997 | 0.026889 | TAAR3 |
| TC19001593.hg.1 | -5.9572 | 0.0039857 | 2.3995 | 0.026889 | PLAUR |
| TC01003035.hg.1 | -5.9522 | 0.0039979 | 2.3982 | 0.026934 | VTCN1 |
| TC15001079.hg.1 | -5.9335 | 0.0040444 | 2.3931 | 0.027211 | ATP10A |
| TC16000645.hg.1 | 5.9242 | 0.0040676 | 2.3907 | 0.027331 | HSD17B2 |
| TC01002769.hg.1 | -5.9087 | 0.0041068 | 2.3865 | 0.027556 | LRRC40 |
| TC04002789.hg.1 | 5.9065 | 0.0041122 | 2.3859 | 0.027556 | NA |
| TC12000412.hg.1 | -5.897 | 0.0041367 | 2.3833 | 0.027653 | NA |
| TC02001713.hg.1 | -5.8966 | 0.0041377 | 2.3832 | 0.027653 | GALNT14 |
| TC07001851.hg.1 | 5.876 | 0.0041907 | 2.3777 | 0.02797 | KCP |
| TC07002773.hg.1 | -5.8729 | 0.004199 | 2.3769 | 0.027988 | ACTB |
| TC06003152.hg.1 | -5.8532 | 0.0042507 | 2.3715 | 0.028274 | SNX9 |
| TC16001250.hg.1 | -5.8522 | 0.0042533 | 2.3713 | 0.028274 | PHLPP2 |
| TC14001578.hg.1 | 5.8499 | 0.0042594 | 2.3707 | 0.028277 | NA |
| TC02004506.hg.1 | -5.8283 | 0.0043173 | 2.3648 | 0.028624 | NA |
| TC07002300.hg.1 | 5.821 | 0.0043371 | 2.3628 | 0.028717 | NA |
| TC20001228.hg.1 | 5.8187 | 0.0043434 | 2.3622 | 0.02872 | SGK2 |
| TC03003322.hg.1 | -5.8155 | 0.004352 | 2.3613 | 0.028739 | UPK1B |
| TC01004216.hg.1 | -5.8097 | 0.004368 | 2.3597 | 0.02877 | KLHDC7A |
| TC01001408.hg.1 | -5.8069 | 0.0043758 | 2.3589 | 0.02877 | NA |
| TC04002082.hg.1 | -5.8047 | 0.0043817 | 2.3584 | 0.02877 | NA |
| TC0M000019.hg.1 | 5.8046 | 0.0043819 | 2.3583 | 0.02877 | NA |
| TC02004034.hg.1 | 5.8031 | 0.0043861 | 2.3579 | 0.02877 | LOC285147 |
| TC07000126.hg.1 | -5.8013 | 0.0043911 | 2.3574 | 0.02877 | NA |
| TC08001929.hg.1 | -5.796 | 0.0044056 | 2.356 | 0.028827 | LINC01606 |
| TC15000066.hg.1 | -5.7927 | 0.004415 | 2.3551 | 0.02885 | SNORD116-23 |
| TC09001154.hg.1 | -5.7878 | 0.0044286 | 2.3537 | 0.028863 | NA |
| TC02000874.hg.1 | 5.7863 | 0.0044328 | 2.3533 | 0.028863 | MGAT5 |
| TC16001902.hg.1 | 5.7858 | 0.0044342 | 2.3532 | 0.028863 | NA |
| TC19002097.hg.1 | -5.7755 | 0.0044629 | 2.3504 | 0.028986 | FXYD5 |
| TC0X000521.hg.1 | -5.7749 | 0.0044647 | 2.3502 | 0.028986 | CLDN2 |
| TC05001909.hg.1 | -5.768 | 0.0044841 | 2.3483 | 0.029074 | DPYSL3 |
| TC06003365.hg.1 | -5.7657 | 0.0044906 | 2.3477 | 0.029078 | NA |
| TC10002083.hg.1 | -5.7617 | 0.0045019 | 2.3466 | 0.029081 | NA |
| TC05000279.hg.1 | -5.7615 | 0.0045026 | 2.3465 | 0.029081 | NLN |
| TC13000880.hg.1 | -5.7589 | 0.00451 | 2.3458 | 0.029091 | ANKRD10-IT1 |
| TC12001755.hg.1 | -5.7452 | 0.0045493 | 2.3421 | 0.029278 | CSRP2 |
| TC0X001637.hg.1 | 5.7427 | 0.0045564 | 2.3414 | 0.029278 | NA |
| TC14000280.hg.1 | -5.7426 | 0.0045566 | 2.3414 | 0.029278 | ARF6 |
| TC06003833.hg.1 | -5.7379 | 0.0045702 | 2.3401 | 0.029328 | NA |
| TC01000311.hg.1 | -5.7351 | 0.0045784 | 2.3393 | 0.029343 | RCAN3 |
| TC15001456.hg.1 | -5.7248 | 0.0046085 | 2.3364 | 0.029471 | MYO5A |
| TC01003030.hg.1 | -5.7223 | 0.0046158 | 2.3358 | 0.029471 | IGSF3 |
| TC14001562.hg.1 | 5.7222 | 0.0046161 | 2.3357 | 0.029471 | AHNAK2 |
| TC08000378.hg.1 | -5.7175 | 0.00463 | 2.3344 | 0.029522 | XKR4 |
| TC01006369.hg.1 | -5.7148 | 0.0046378 | 2.3337 | 0.029534 | NBPF9 |
| TC04001926.hg.1 | -5.7048 | 0.0046674 | 2.3309 | 0.029685 | NA |
| TC12002976.hg.1 | -5.6748 | 0.0047576 | 2.3226 | 0.030187 | NA |
| TC17001617.hg.1 | -5.673 | 0.0047631 | 2.3221 | 0.030187 | ARL17A |
| TC12001380.hg.1 | -5.6714 | 0.0047681 | 2.3217 | 0.030187 | SYT10 |
| TC09000295.hg.1 | 5.6706 | 0.0047705 | 2.3214 | 0.030187 | ANKRD20A4 |
| PSR11002952.hg.1 | -5.6684 | 0.0047772 | 2.3208 | 0.030191 | NA |
| TC06004072.hg.1 | 5.657 | 0.0048122 | 2.3177 | 0.030365 | HCP5 |
| TC02004744.hg.1 | 5.6555 | 0.0048169 | 2.3172 | 0.030365 | TFPI |
| TC01004933.hg.1 | -5.635 | 0.0048808 | 2.3115 | 0.030713 | LHX9 |
| TC17000246.hg.1 | 5.6339 | 0.0048843 | 2.3112 | 0.030713 | ALDH3A2 |
| TC15001544.hg.1 | -5.6198 | 0.0049287 | 2.3073 | 0.030953 | HERC1 |
| TC06001879.hg.1 | -5.6172 | 0.0049371 | 2.3065 | 0.030967 | COL12A1 |
| TC19002677.hg.1 | -5.6111 | 0.0049563 | 2.3048 | 0.031049 | RGL3 |
| TC16001031.hg.1 | -5.599 | 0.0049955 | 2.3014 | 0.031255 | DCTPP1 |
| TC01003333.hg.1 | 5.5856 | 0.0050389 | 2.2977 | 0.031479 | PAQR6 |
| TC09000999.hg.1 | -5.584 | 0.005044 | 2.2972 | 0.031479 | DDX58 |
| TC19002413.hg.1 | -5.5819 | 0.0050511 | 2.2966 | 0.031484 | NA |
| TC01003901.hg.1 | 5.5794 | 0.0050591 | 2.2959 | 0.031495 | ITPKB |
| TC12001279.hg.1 | -5.5727 | 0.0050813 | 2.294 | 0.031594 | RERG |
| TC04001948.hg.1 | -5.5594 | 0.0051253 | 2.2903 | 0.031828 | NA |
| TC02001030.hg.1 | 5.5572 | 0.0051328 | 2.2896 | 0.031835 | ITGA6 |
| TC08000505.hg.1 | -5.5538 | 0.0051441 | 2.2887 | 0.031865 | PKIA |
| TC01003874.hg.1 | -5.545 | 0.0051735 | 2.2862 | 0.032008 | TP53BP2 |
| TC01000685.hg.1 | -5.5372 | 0.0052001 | 2.284 | 0.032127 | PRKAA2 |
| TC18000856.hg.1 | -5.5325 | 0.005216 | 2.2827 | 0.032127 | NA |
| TC03002973.hg.1 | -5.5318 | 0.0052183 | 2.2825 | 0.032127 | NA |
| TC12000012.hg.1 | -5.5318 | 0.0052184 | 2.2825 | 0.032127 | WNK1 |
| TC08001099.hg.1 | -5.5209 | 0.0052557 | 2.2794 | 0.03219 | DUSP4 |
| TC20000928.hg.1 | -5.5207 | 0.0052563 | 2.2793 | 0.03219 | B4GALT5 |
| TC18000727.hg.1 | -5.5181 | 0.0052653 | 2.2786 | 0.03219 | ONECUT2 |
| PSR01001654.hg.1 | -5.5156 | 0.005274 | 2.2779 | 0.03219 | NA |
| TC03000837.hg.1 | -5.512 | 0.0052865 | 2.2768 | 0.03219 | RAP2B |
| TC01002352.hg.1 | -5.5088 | 0.0052976 | 2.2759 | 0.03219 | FUCA1 |
| TC03002466.hg.1 | -5.5083 | 0.0052991 | 2.2758 | 0.03219 | NA |
| TC6_apd_hap1000029.hg.1 | -5.5045 | 0.0053124 | 2.2747 | 0.03219 | TUBB |
| TC6_dbb_hap3000038.hg.1 | -5.5045 | 0.0053124 | 2.2747 | 0.03219 | TUBB |
| TC6_mann_hap4000040.hg.1 | -5.5045 | 0.0053124 | 2.2747 | 0.03219 | TUBB |
| TC6_mcf_hap5000032.hg.1 | -5.5045 | 0.0053124 | 2.2747 | 0.03219 | TUBB |
| TC6_qbl_hap6000038.hg.1 | -5.5045 | 0.0053124 | 2.2747 | 0.03219 | TUBB |
| TC6_ssto_hap7000038.hg.1 | -5.5045 | 0.0053124 | 2.2747 | 0.03219 | TUBB |
| TC14002303.hg.1 | -5.4937 | 0.0053502 | 2.2716 | 0.032351 | FLVCR2 |
| TC05001558.hg.1 | -5.4927 | 0.0053537 | 2.2713 | 0.032351 | EDIL3 |
| TC07003019.hg.1 | -5.4912 | 0.005359 | 2.2709 | 0.032351 | ABHD11 |
| TC01000802.hg.1 | -5.4895 | 0.0053649 | 2.2704 | 0.032351 | NA |
| TC09001234.hg.1 | 5.4243 | 0.005601 | 2.2517 | 0.033734 | NA |
| TC0X001376.hg.1 | -5.4193 | 0.0056197 | 2.2503 | 0.033806 | GPC4 |
| TC11000548.hg.1 | 5.4143 | 0.0056382 | 2.2489 | 0.033876 | SCGB2A1 |
| TC04000555.hg.1 | -5.4121 | 0.0056464 | 2.2482 | 0.033885 | CYP2U1 |
| TC08001269.hg.1 | -5.4087 | 0.0056595 | 2.2472 | 0.033923 | GGH |
| TC10002530.hg.1 | -5.4051 | 0.0056729 | 2.2462 | 0.033963 | NA |
| TC19002544.hg.1 | -5.3989 | 0.0056964 | 2.2444 | 0.034002 | NA |
| TC22000571.hg.1 | 5.3984 | 0.0056984 | 2.2442 | 0.034002 | NA |
| TC11000345.hg.1 | -5.398 | 0.0056999 | 2.2441 | 0.034002 | PRR5L |
| TC10000769.hg.1 | 5.3961 | 0.005707 | 2.2436 | 0.034004 | CNNM2 |
| TC15002540.hg.1 | -5.39 | 0.0057304 | 2.2418 | 0.034102 | MYO5A |
| TC09002021.hg.1 | -5.3871 | 0.0057413 | 2.241 | 0.034109 | GCNT1 |
| TC02000207.hg.1 | -5.3861 | 0.0057451 | 2.2407 | 0.034109 | EHD3 |
| TC07001570.hg.1 | 5.3683 | 0.0058141 | 2.2355 | 0.034477 | SEMA3D |
| TC12001123.hg.1 | -5.3657 | 0.0058239 | 2.2348 | 0.034494 | C12orf4 |
| TC21000892.hg.1 | -5.364 | 0.0058306 | 2.2343 | 0.034494 | ADAMTS5 |
| TC06001074.hg.1 | 5.3605 | 0.0058444 | 2.2333 | 0.034534 | SASH1 |
| TC03000221.hg.1 | -5.358 | 0.005854 | 2.2325 | 0.03455 | NKTR |
| TC01000794.hg.1 | -5.3527 | 0.0058752 | 2.231 | 0.034635 | IFI44L |
| TC07003154.hg.1 | -5.3449 | 0.0059057 | 2.2287 | 0.034763 | NA |
| TC02002439.hg.1 | -5.3436 | 0.0059108 | 2.2284 | 0.034763 | NA |
| TC0X002048.hg.1 | -5.3379 | 0.0059335 | 2.2267 | 0.034855 | NA |
| TC04001593.hg.1 | -5.3356 | 0.0059428 | 2.226 | 0.034868 | NA |
| TC01003240.hg.1 | -5.3311 | 0.005961 | 2.2247 | 0.034902 | S100A11 |
| TC13001592.hg.1 | -5.3307 | 0.0059624 | 2.2246 | 0.034902 | RBM26 |
| TC02002210.hg.1 | -5.3133 | 0.0060326 | 2.2195 | 0.035245 | NA |
| TC15000046.hg.1 | -5.3117 | 0.0060392 | 2.219 | 0.035245 | PWAR6 |
| TC22001420.hg.1 | 5.311 | 0.0060422 | 2.2188 | 0.035245 | CHKB-CPT1B |
| TC06002899.hg.1 | 5.2977 | 0.0060965 | 2.2149 | 0.03552 | LOC101928714 |
| TC03002723.hg.1 | 5.2904 | 0.0061268 | 2.2128 | 0.035655 | NA |
| TC19000374.hg.1 | -5.2742 | 0.0061946 | 2.208 | 0.035987 | MIR640 |
| TC20000861.hg.1 | 5.2733 | 0.0061982 | 2.2077 | 0.035987 | OSER1 |
| TC04001002.hg.1 | -5.2691 | 0.0062158 | 2.2065 | 0.036048 | NA |
| TC03000917.hg.1 | -5.2635 | 0.0062397 | 2.2048 | 0.036144 | SKIL |
| TC04000903.hg.1 | 5.2566 | 0.006269 | 2.2028 | 0.036272 | SNX25 |
| TC19000699.hg.1 | -5.2511 | 0.0062925 | 2.2012 | 0.036366 | EMP3 |
| TC12000781.hg.1 | -5.2468 | 0.0063111 | 2.1999 | 0.036415 | UTP20 |
| TC03002118.hg.1 | -5.2445 | 0.0063209 | 2.1992 | 0.036415 | MB21D2 |
| TC03003161.hg.1 | 5.244 | 0.0063228 | 2.1991 | 0.036415 | NA |
| PSR11017826.hg.1 | -5.2414 | 0.0063342 | 2.1983 | 0.036437 | NA |
| TC20001450.hg.1 | -5.2398 | 0.0063413 | 2.1978 | 0.036437 | NA |
| TC02004979.hg.1 | -5.2371 | 0.0063528 | 2.197 | 0.036461 | NA |
| TC07000266.hg.1 | -5.2328 | 0.0063715 | 2.1958 | 0.036527 | STK17A |
| TC01003745.hg.1 | -5.2248 | 0.0064064 | 2.1934 | 0.036684 | NUAK2 |
| TC09001112.hg.1 | 5.2182 | 0.0064354 | 2.1914 | 0.036808 | LOC101059935 |
| TC19001764.hg.1 | 5.2165 | 0.006443 | 2.1909 | 0.03681 | KLK6 |
| TC17002137.hg.1 | -5.205 | 0.0064938 | 2.1875 | 0.037058 | NA |
| TC16000198.hg.1 | -5.2017 | 0.0065084 | 2.1865 | 0.037099 | NPIPA1 |
| TC15001174.hg.1 | 5.1999 | 0.0065165 | 2.186 | 0.037103 | GOLGA8O |
| TC15001217.hg.1 | -5.1956 | 0.0065358 | 2.1847 | 0.03717 | RASGRP1 |
| TC01006428.hg.1 | -5.1933 | 0.0065464 | 2.184 | 0.037188 | NBPF9 |
| TC05001714.hg.1 | -5.1874 | 0.0065727 | 2.1823 | 0.037272 | LOX |
| TC03000946.hg.1 | -5.1867 | 0.0065761 | 2.182 | 0.037272 | KCCAT211 |
| TC11001282.hg.1 | -5.1848 | 0.0065845 | 2.1815 | 0.037278 | KCNQ1OT1 |
| TC03003201.hg.1 | 5.1794 | 0.006609 | 2.1799 | 0.037326 | MECOM |
| TC03003160.hg.1 | 5.1787 | 0.006612 | 2.1797 | 0.037326 | NA |
| TC02003240.hg.1 | 5.1774 | 0.0066184 | 2.1792 | 0.037326 | SPTBN1 |
| TC07003096.hg.1 | 5.1763 | 0.006623 | 2.1789 | 0.037326 | LHFPL3-AS2 |
| TC09002517.hg.1 | 5.1724 | 0.0066409 | 2.1778 | 0.037385 | NA |
| TC08002245.hg.1 | 5.1702 | 0.006651 | 2.1771 | 0.0374 | CSGALNACT1 |
| TC05000918.hg.1 | -5.1625 | 0.0066863 | 2.1748 | 0.037556 | WWC1 |
| TC07000873.hg.1 | -5.1605 | 0.0066959 | 2.1742 | 0.037567 | NA |
| TC0X002226.hg.1 | -5.1586 | 0.0067047 | 2.1736 | 0.037575 | SMARCA1 |
| TC11001465.hg.1 | -5.1429 | 0.0067775 | 2.1689 | 0.037907 | HPS5 |
| TC10000047.hg.1 | -5.1426 | 0.006779 | 2.1688 | 0.037907 | NET1 |
| TC11002170.hg.1 | -5.1372 | 0.0068043 | 2.1672 | 0.037979 | FZD4 |
| TC11002677.hg.1 | -5.1357 | 0.0068118 | 2.1667 | 0.037979 | SERPING1 |
| TC04002560.hg.1 | -5.135 | 0.0068148 | 2.1665 | 0.037979 | IGFBP7 |
| PSR01006341.hg.1 | -5.113 | 0.0069199 | 2.1599 | 0.038495 | NA |
| TC04002851.hg.1 | -5.1124 | 0.0069227 | 2.1597 | 0.038495 | AGA |
| TC09001495.hg.1 | -5.107 | 0.0069488 | 2.1581 | 0.038597 | ZNF883 |
| TC12003212.hg.1 | -5.0935 | 0.0070145 | 2.154 | 0.03889 | ALDH2 |
| TC07003333.hg.1 | -5.0929 | 0.0070171 | 2.1538 | 0.03889 | NSUN5P1 |
| TC14001929.hg.1 | 5.0874 | 0.0070445 | 2.1522 | 0.038977 | PPP1R3E |
| TC0X001828.hg.1 | 5.0866 | 0.0070484 | 2.1519 | 0.038977 | NA |
| TC6_ssto_hap7000048.hg.1 | 5.0776 | 0.0070926 | 2.1492 | 0.039178 | HCP5 |
| TC17002723.hg.1 | 5.0695 | 0.007133 | 2.1467 | 0.03934 | NA |
| TC02001117.hg.1 | 5.0685 | 0.0071377 | 2.1464 | 0.03934 | NA |
| TC14001777.hg.1 | -5.0652 | 0.0071545 | 2.1454 | 0.03936 | NA |
| TC08000379.hg.1 | -5.0647 | 0.007157 | 2.1453 | 0.03936 | LOC105375844 |
| TC01003471.hg.1 | -5.054 | 0.0072109 | 2.142 | 0.039505 | MIR921 |
| TC13000329.hg.1 | -5.0534 | 0.0072137 | 2.1418 | 0.039505 | GPC6 |
| TC17000918.hg.1 | -5.0528 | 0.007217 | 2.1416 | 0.039505 | RNF213 |
| TC17002909.hg.1 | -5.0525 | 0.0072184 | 2.1416 | 0.039505 | HOXB3 |
| TC15000059.hg.1 | -5.051 | 0.0072262 | 2.1411 | 0.039505 | SNORD116-13 |
| TC12000227.hg.1 | 5.05 | 0.0072309 | 2.1408 | 0.039505 | PDE3A |
| 3597901_st | -5.0468 | 0.0072474 | 2.1398 | 0.039529 | NA |
| TC19000384.hg.1 | -5.0425 | 0.0072692 | 2.1385 | 0.039529 | ZNF486 |
| PSR01006338.hg.1 | -5.0421 | 0.0072714 | 2.1384 | 0.039529 | NA |
| TC16001417.hg.1 | -5.041 | 0.0072769 | 2.1381 | 0.039529 | SHISA9 |
| TC13000741.hg.1 | -5.0397 | 0.0072838 | 2.1376 | 0.039529 | KCTD12 |
| TC01004845.hg.1 | -5.0384 | 0.0072904 | 2.1372 | 0.039529 | PAPPA2 |
| TC02001176.hg.1 | 5.0375 | 0.0072948 | 2.137 | 0.039529 | CASP10 |
| TC05003082.hg.1 | -5.0368 | 0.0072985 | 2.1368 | 0.039529 | NA |
| TC03002133.hg.1 | -5.0232 | 0.0073689 | 2.1326 | 0.039867 | ATP13A3 |
| TC15000895.hg.1 | 5.0057 | 0.0074606 | 2.1272 | 0.040301 | NA |
| TC14001678.hg.1 | -5.0048 | 0.0074653 | 2.127 | 0.040301 | ARF6 |
| TC19001369.hg.1 | -4.9783 | 0.0076068 | 2.1188 | 0.040973 | ZNF675 |
| TC16002096.hg.1 | -4.9766 | 0.0076154 | 2.1183 | 0.040973 | NPIPA1 |
| TC08000239.hg.1 | -4.9755 | 0.0076215 | 2.118 | 0.040973 | NA |
| TC21000884.hg.1 | 4.9753 | 0.0076225 | 2.1179 | 0.040973 | NA |
| TC6_cox_hap2000045.hg.1 | -4.9692 | 0.0076558 | 2.116 | 0.041108 | TUBB |
| TC15000060.hg.1 | -4.965 | 0.0076787 | 2.1147 | 0.041186 | SNORD116-14 |
| TC04001753.hg.1 | -4.9627 | 0.0076911 | 2.114 | 0.041209 | HPGD |
| TC01002908.hg.1 | -4.957 | 0.0077223 | 2.1123 | 0.041332 | PLPPR5 |
| TC04001949.hg.1 | -4.9508 | 0.0077566 | 2.1103 | 0.041471 | NA |
| TC06003630.hg.1 | 4.9434 | 0.0077978 | 2.108 | 0.041609 | MOCS1 |
| TC02001054.hg.1 | 4.9428 | 0.007801 | 2.1078 | 0.041609 | HOXD3 |
| TC04002580.hg.1 | -4.9417 | 0.0078073 | 2.1075 | 0.041609 | NA |
| TC10002226.hg.1 | -4.9348 | 0.0078459 | 2.1054 | 0.04177 | HELLS |
| TC12001659.hg.1 | -4.9297 | 0.0078748 | 2.1038 | 0.041879 | PPM1H |
| TC15000049.hg.1 | -4.9248 | 0.0079022 | 2.1023 | 0.041979 | SNORD116-2 |
| TC11002242.hg.1 | -4.9233 | 0.0079105 | 2.1018 | 0.041979 | PDGFD |
| TC01004027.hg.1 | -4.9177 | 0.0079425 | 2.1 | 0.042077 | CHML |
| TC08000462.hg.1 | 4.9171 | 0.0079457 | 2.0999 | 0.042077 | LOC101926892 |
| TC10001416.hg.1 | -4.9131 | 0.0079688 | 2.0986 | 0.042154 | AGAP5 |
| TC02004488.hg.1 | 4.9019 | 0.0080332 | 2.0951 | 0.04245 | NA |
| 3447830_st | -4.8995 | 0.0080467 | 2.0944 | 0.042477 | NA |
| TC17000132.hg.1 | -4.8963 | 0.0080651 | 2.0934 | 0.042529 | MIR4521 |
| TC21000145.hg.1 | 4.872 | 0.008208 | 2.0858 | 0.043237 | RIMKLBP1 |
| TC02000961.hg.1 | 4.8691 | 0.0082248 | 2.0849 | 0.04328 | NA |
| TC06002589.hg.1 | -4.8614 | 0.0082709 | 2.0824 | 0.043477 | NA |
| TC0X001983.hg.1 | -4.8595 | 0.0082822 | 2.0819 | 0.04349 | NA |
| TC09001890.hg.1 | -4.8566 | 0.0082995 | 2.0809 | 0.043535 | RPL36AP33 |
| 3151626_st | -4.8542 | 0.0083139 | 2.0802 | 0.043565 | NA |
| TC07002772.hg.1 | -4.8394 | 0.0084034 | 2.0755 | 0.043941 | ACTB |
| TC0X001186.hg.1 | -4.8343 | 0.008435 | 2.0739 | 0.043941 | RPS6KA6 |
| TC12003020.hg.1 | -4.8341 | 0.008436 | 2.0739 | 0.043941 | NA |
| TC11003400.hg.1 | 4.8336 | 0.0084394 | 2.0737 | 0.043941 | CDON |
| TC01002055.hg.1 | -4.828 | 0.0084736 | 2.0719 | 0.043941 | NA |
| TC01003865.hg.1 | -4.828 | 0.0084736 | 2.0719 | 0.043941 | NA |
| TC04001502.hg.1 | -4.828 | 0.0084736 | 2.0719 | 0.043941 | NA |
| TC09000858.hg.1 | -4.828 | 0.0084736 | 2.0719 | 0.043941 | NA |
| TC16000715.hg.1 | -4.828 | 0.0084736 | 2.0719 | 0.043941 | NA |
| TC19000980.hg.1 | -4.828 | 0.0084736 | 2.0719 | 0.043941 | NA |
| TC15001337.hg.1 | 4.8252 | 0.0084909 | 2.071 | 0.043954 | CEP152 |
| TC0X002337.hg.1 | -4.8247 | 0.0084937 | 2.0709 | 0.043954 | ACSL4 |
| TC21000735.hg.1 | 4.8215 | 0.0085135 | 2.0699 | 0.044012 | NA |
| TC0X001180.hg.1 | -4.8115 | 0.0085762 | 2.0667 | 0.04429 | ITM2A |
| TC17000073.hg.1 | -4.8061 | 0.0086098 | 2.065 | 0.044417 | TXNDC17 |
| TC19001366.hg.1 | -4.7941 | 0.0086856 | 2.0612 | 0.044762 | NA |
| TC01005047.hg.1 | 4.7881 | 0.0087235 | 2.0593 | 0.044911 | NA |
| TC19000974.hg.1 | -4.7866 | 0.0087334 | 2.0588 | 0.044916 | WASH5P |
| TC09002894.hg.1 | -4.7816 | 0.0087652 | 2.0572 | 0.044991 | GOLM1 |
| TC09000844.hg.1 | -4.7815 | 0.008766 | 2.0572 | 0.044991 | TUBB4B |
| TC04002245.hg.1 | 4.7779 | 0.0087891 | 2.0561 | 0.045063 | MMAA |
| 47420069_st | -4.7754 | 0.0088049 | 2.0553 | 0.045098 | NA |
| PSR05015334.hg.1 | -4.7683 | 0.008851 | 2.053 | 0.045257 | NA |
| TC20001131.hg.1 | -4.7678 | 0.0088541 | 2.0529 | 0.045257 | RIN2 |
| TC08001920.hg.1 | -4.7654 | 0.0088702 | 2.0521 | 0.045293 | NA |
| TC15000065.hg.1 | -4.7617 | 0.0088939 | 2.0509 | 0.045368 | SNORD116-20 |
| PSR03025025.hg.1 | -4.7575 | 0.0089216 | 2.0496 | 0.045463 | NA |
| 3151628_st | -4.7483 | 0.008982 | 2.0466 | 0.045724 | NA |
| TC05003439.hg.1 | -4.7371 | 0.0090563 | 2.043 | 0.046055 | ITGA1 |
| TC12002170.hg.1 | -4.7317 | 0.0090926 | 2.0413 | 0.046193 | NA |
| TC09002898.hg.1 | 4.7262 | 0.0091296 | 2.0395 | 0.046334 | ANKRD20A3 |
| TC21000891.hg.1 | -4.7236 | 0.0091475 | 2.0387 | 0.046372 | ADAMTS1 |
| TC09000561.hg.1 | -4.7224 | 0.0091556 | 2.0383 | 0.046372 | MIR4668 |
| TC18000016.hg.1 | -4.72 | 0.0091713 | 2.0376 | 0.046404 | MYL12A |
| TC02003597.hg.1 | 4.7129 | 0.00922 | 2.0353 | 0.046603 | MGAT5 |
| TC16001720.hg.1 | -4.6989 | 0.0093159 | 2.0308 | 0.04704 | TUBB3 |
| PSR01065865.hg.1 | -4.6962 | 0.0093348 | 2.0299 | 0.047089 | NA |
| TC04002109.hg.1 | -4.6945 | 0.0093462 | 2.0294 | 0.047098 | NA |
| TC11002221.hg.1 | 4.6867 | 0.0094011 | 2.0268 | 0.047327 | TRPC6 |
| PSR05015343.hg.1 | -4.6824 | 0.0094308 | 2.0255 | 0.047429 | NA |
| TC12000932.hg.1 | -4.6653 | 0.0095518 | 2.0199 | 0.047989 | HSPB8 |
| TC07002618.hg.1 | -4.6571 | 0.0096107 | 2.0172 | 0.048237 | TBXAS1 |
| 2930617_st | -4.6547 | 0.0096277 | 2.0165 | 0.048274 | NA |
| TC21000113.hg.1 | -4.6495 | 0.0096651 | 2.0148 | 0.048413 | HUNK |
| TC06002661.hg.1 | -4.6366 | 0.0097587 | 2.0106 | 0.048825 | ZNF391 |
| 2930626_st | -4.6355 | 0.0097669 | 2.0102 | 0.048825 | NA |
| TC18000876.hg.1 | -4.6285 | 0.0098186 | 2.008 | 0.048999 | CHST9 |
| TC16000263.hg.1 | -4.6281 | 0.0098213 | 2.0078 | 0.048999 | NA |
| TC14002234.hg.1 | 4.6255 | 0.0098405 | 2.007 | 0.049045 | IGHD3-10 |
| TC04002771.hg.1 | 4.6234 | 0.0098562 | 2.0063 | 0.049058 | ZNF827 |
| TC08001988.hg.1 | -4.6225 | 0.0098627 | 2.006 | 0.049058 | PKIA |
| 3475644_st | -4.6211 | 0.0098726 | 2.0056 | 0.049059 | NA |
| TC21000402.hg.1 | -4.6181 | 0.009895 | 2.0046 | 0.049061 | C21orf62 |
| TC14001316.hg.1 | -4.6171 | 0.0099025 | 2.0043 | 0.049061 | ACYP1 |
| TC02001246.hg.1 | 4.6163 | 0.0099088 | 2.004 | 0.049061 | CPS1 |
| TC04001845.hg.1 | -4.6158 | 0.0099122 | 2.0038 | 0.049061 | NA |
| PSR04018304.hg.1 | -4.6095 | 0.0099594 | 2.0018 | 0.049245 | NA |
| TC17001952.hg.1 | -4.6079 | 0.0099714 | 2.0012 | 0.049256 | ACTG1 |
| TC11000967.hg.1 | -4.5926 | 0.010087 | 1.9962 | 0.049779 | RNA5SP348 |
| TC01005769.hg.1 | -4.581 | 0.010176 | 1.9924 | 0.050166 | NA |
| TC02000762.hg.1 | -4.5761 | 0.010214 | 1.9908 | 0.050304 | INHBB |
| TC09000879.hg.1 | -4.5712 | 0.010251 | 1.9892 | 0.05044 | GLIS3 |
| TC02003749.hg.1 | -4.5679 | 0.010277 | 1.9881 | 0.050453 | NA |
| TC02002329.hg.1 | -4.567 | 0.010284 | 1.9878 | 0.050453 | NA |
| TC13000598.hg.1 | -4.567 | 0.010284 | 1.9878 | 0.050453 | NA |
| TC08002451.hg.1 | -4.5549 | 0.010379 | 1.9839 | 0.050865 | YWHAZ |
| TC01005883.hg.1 | -4.5534 | 0.010391 | 1.9833 | 0.050876 | TAGLN2 |
| TC10000636.hg.1 | -4.5518 | 0.010403 | 1.9828 | 0.050886 | IFIT2 |
| 47424057_st | -4.5503 | 0.010415 | 1.9823 | 0.050886 | NA |
| PSR16021742.hg.1 | -4.5491 | 0.010424 | 1.9819 | 0.050886 | NA |
| TC11000332.hg.1 | -4.5472 | 0.01044 | 1.9813 | 0.050886 | CD44 |
| PSR04018301.hg.1 | -4.5467 | 0.010444 | 1.9811 | 0.050886 | NA |
| TC13000151.hg.1 | -4.5351 | 0.010536 | 1.9773 | 0.051287 | DGKH |
| TC12001504.hg.1 | -4.5292 | 0.010584 | 1.9754 | 0.051467 | NA |
| TC04002099.hg.1 | 4.5227 | 0.010636 | 1.9732 | 0.051671 | NA |
| TC07002184.hg.1 | -4.5131 | 0.010715 | 1.97 | 0.052002 | ITGB8 |
| TC02003704.hg.1 | 4.511 | 0.010732 | 1.9693 | 0.052037 | NA |
| TC02000881.hg.1 | 4.5001 | 0.010821 | 1.9657 | 0.052419 | R3HDM1 |
| TC0Y000263.hg.1 | -4.4959 | 0.010857 | 1.9643 | 0.052539 | NLGN4Y |
| TC18001001.hg.1 | -4.4848 | 0.01095 | 1.9606 | 0.052937 | SERPINB8 |
| TC0X001343.hg.1 | -4.4828 | 0.010966 | 1.96 | 0.052965 | NA |
| TC09000988.hg.1 | -4.4702 | 0.011073 | 1.9557 | 0.053391 | MOB3B |
| TC15000048.hg.1 | -4.4699 | 0.011075 | 1.9556 | 0.053391 | SNORD116-1 |
| TC09001419.hg.1 | -4.4662 | 0.011107 | 1.9544 | 0.053411 | BAAT |
| TC15000067.hg.1 | -4.466 | 0.011109 | 1.9543 | 0.053411 | SNORD116-24 |
| 3671544_st | 4.4657 | 0.011112 | 1.9542 | 0.053411 | NA |
| TC10000258.hg.1 | -4.4563 | 0.011192 | 1.9511 | 0.053747 | NA |
| TC04000003.hg.1 | -4.45 | 0.011247 | 1.949 | 0.053957 | NA |
| TC01002828.hg.1 | -4.4385 | 0.011347 | 1.9451 | 0.054386 | BCL10 |
| TC11003248.hg.1 | -4.436 | 0.011369 | 1.9443 | 0.054438 | C2CD3 |
| TC07003060.hg.1 | -4.4316 | 0.011408 | 1.9428 | 0.054548 | FAM133DP |
| PSR16017882.hg.1 | -4.4309 | 0.011414 | 1.9426 | 0.054548 | NA |
| TC14002040.hg.1 | -4.4262 | 0.011456 | 1.941 | 0.054697 | HIF1A-AS2 |
| 3581347_st | -4.4248 | 0.011468 | 1.9405 | 0.054703 | NA |
| TC14002203.hg.1 | -4.4171 | 0.011537 | 1.9379 | 0.054979 | SLIRP |
| TC06002070.hg.1 | -4.4084 | 0.011615 | 1.935 | 0.055298 | NA |
| TC15001004.hg.1 | -4.4029 | 0.011665 | 1.9331 | 0.055484 | WASH3P |
| TC11002453.hg.1 | 4.3998 | 0.011693 | 1.9321 | 0.055566 | NA |
| 3374446_st | -4.3934 | 0.011752 | 1.9299 | 0.055736 | NA |
| TC12000779.hg.1 | 4.3923 | 0.011761 | 1.9295 | 0.055736 | GAS2L3 |
| TC03002079.hg.1 | -4.3922 | 0.011763 | 1.9295 | 0.055736 | IGF2BP2 |
| TC02004993.hg.1 | -4.3894 | 0.011788 | 1.9286 | 0.055802 | C2orf15 |
| TC15002071.hg.1 | 4.3706 | 0.011963 | 1.9222 | 0.056577 | GOLGA8IP |
| TC15000222.hg.1 | 4.3647 | 0.012018 | 1.9202 | 0.056786 | GOLGA8N |
| TC10000820.hg.1 | 4.3612 | 0.012051 | 1.919 | 0.056875 | TCF7L2 |
| TC04000559.hg.1 | -4.3602 | 0.01206 | 1.9187 | 0.056875 | OSTC |
| TC08002379.hg.1 | -4.3472 | 0.012184 | 1.9142 | 0.057398 | LOC286191 |
| TC12002256.hg.1 | -4.3462 | 0.012194 | 1.9139 | 0.057398 | NA |
| 2886354_st | -4.327 | 0.012379 | 1.9073 | 0.058215 | NA |
| TC02004470.hg.1 | 4.3237 | 0.012411 | 1.9062 | 0.058311 | NA |
| TC01000109.hg.1 | 4.3149 | 0.012498 | 1.9032 | 0.058664 | HMGN2P17 |
| TC18000922.hg.1 | 4.3105 | 0.012541 | 1.9017 | 0.058811 | ZBTB7C |
| TC20001392.hg.1 | -4.3049 | 0.012596 | 1.8998 | 0.058962 | NA |
| TC03000632.hg.1 | -4.3037 | 0.012608 | 1.8993 | 0.058962 | DTX3L |
| TC04000588.hg.1 | -4.3037 | 0.012609 | 1.8993 | 0.058962 | NA |
| TC22001326.hg.1 | -4.3008 | 0.012638 | 1.8983 | 0.059045 | MYH9 |
| TC12001278.hg.1 | -4.2984 | 0.012662 | 1.8975 | 0.0591 | ARHGDIB |
| TC07002232.hg.1 | 4.289 | 0.012756 | 1.8943 | 0.059404 | NA |
| TC01003759.hg.1 | -4.2885 | 0.012762 | 1.8941 | 0.059404 | C1orf186 |
| TC0X000238.hg.1 | -4.2874 | 0.012772 | 1.8937 | 0.059404 | TIMP1 |
| TC01000938.hg.1 | -4.2872 | 0.012775 | 1.8937 | 0.059404 | SCARNA2 |
| TC07001281.hg.1 | -4.286 | 0.012786 | 1.8933 | 0.059404 | NA |
| TC17_ctg5_hap1000003.hg.1 | -4.2804 | 0.012844 | 1.8913 | 0.059617 | ARL17A |
| TC0X000910.hg.1 | -4.2758 | 0.01289 | 1.8897 | 0.059776 | SCARNA9L |
| TC08001143.hg.1 | -4.2728 | 0.012921 | 1.8887 | 0.059863 | RP11-90P5.7 |
| TC06001046.hg.1 | -4.2713 | 0.012936 | 1.8882 | 0.059879 | ADGRG6 |
| TC05001719.hg.1 | 4.2499 | 0.013159 | 1.8808 | 0.060853 | MGC32805 |
| TC14002059.hg.1 | -4.2436 | 0.013225 | 1.8786 | 0.061101 | ACTN1 |
| TC17002276.hg.1 | -4.2405 | 0.013258 | 1.8775 | 0.061196 | NME1-NME2 |
| TC11003310.hg.1 | 4.2394 | 0.01327 | 1.8771 | 0.061196 | TRPC6 |
| TC14001434.hg.1 | 4.237 | 0.013295 | 1.8763 | 0.061249 | RPS6KA5 |
| TC07002433.hg.1 | 4.236 | 0.013306 | 1.876 | 0.061249 | NA |
| TC06001908.hg.1 | 4.2335 | 0.013332 | 1.8751 | 0.061314 | IBTK |
| TC10000640.hg.1 | -4.2312 | 0.013358 | 1.8743 | 0.061367 | IFIT5 |
| TC04002840.hg.1 | -4.2297 | 0.013373 | 1.8738 | 0.061367 | NA |
| TC18000741.hg.1 | -4.229 | 0.013381 | 1.8735 | 0.061367 | PMAIP1 |
| TC05001854.hg.1 | -4.2233 | 0.013442 | 1.8715 | 0.06159 | HBEGF |
| TC08002291.hg.1 | -4.2141 | 0.013541 | 1.8683 | 0.061989 | RAB11FIP1 |
| TC12000055.hg.1 | 4.2086 | 0.013601 | 1.8664 | 0.062206 | NA |
| TC01001585.hg.1 | -4.2029 | 0.013663 | 1.8644 | 0.062434 | LAMC2 |
| TC10001258.hg.1 | -4.1974 | 0.013725 | 1.8625 | 0.062658 | CTGLF12P |
| TC05000078.hg.1 | -4.1918 | 0.013787 | 1.8605 | 0.062883 | NA |
| TC01005282.hg.1 | -4.1673 | 0.014062 | 1.852 | 0.064035 | TMEM51-AS1 |
| TC04002845.hg.1 | 4.167 | 0.014065 | 1.8519 | 0.064035 | NA |
| TC09000268.hg.1 | -4.1628 | 0.014113 | 1.8504 | 0.064194 | NA |
| TC06003988.hg.1 | -4.1556 | 0.014196 | 1.8478 | 0.064515 | PDE10A |
| TC01005403.hg.1 | -4.1525 | 0.014231 | 1.8468 | 0.064615 | NA |
| TC03003223.hg.1 | 4.1444 | 0.014326 | 1.8439 | 0.064982 | ZMAT3 |
| 3332080_st | -4.1433 | 0.014338 | 1.8435 | 0.064982 | NA |
| TC16002074.hg.1 | -4.1403 | 0.014373 | 1.8424 | 0.065083 | MT1M |
| TC03001408.hg.1 | -4.139 | 0.014388 | 1.842 | 0.06509 | RHOA |
| TC11000340.hg.1 | -4.1345 | 0.01444 | 1.8404 | 0.065269 | NA |
| TC06003695.hg.1 | -4.1293 | 0.014502 | 1.8386 | 0.06546 | DST |
| TC02000994.hg.1 | -4.1287 | 0.014509 | 1.8384 | 0.06546 | B3GALT1 |
| TC02003185.hg.1 | -4.1202 | 0.014611 | 1.8353 | 0.065859 | EML4 |
| TC02003877.hg.1 | 4.116 | 0.01466 | 1.8339 | 0.066022 | SP140 |
| TC15001679.hg.1 | -4.1097 | 0.014735 | 1.8316 | 0.066301 | NA |
| TC6_mcf_hap5000046.hg.1 | 4.1087 | 0.014748 | 1.8313 | 0.066301 | HCP5 |
| TC10001171.hg.1 | -4.1052 | 0.01479 | 1.83 | 0.066429 | NA |
| TC10000340.hg.1 | -4.08 | 0.015098 | 1.8211 | 0.06775 | AGAP6 |
| TC03002239.hg.1 | -4.0753 | 0.015157 | 1.8194 | 0.06794 | NA |
| TC19001721.hg.1 | -4.0745 | 0.015167 | 1.8191 | 0.06794 | RRAS |
| PSR01006332.hg.1 | -4.0661 | 0.015272 | 1.8161 | 0.068347 | NA |
| TC04002881.hg.1 | 4.0612 | 0.015333 | 1.8144 | 0.068559 | SLED1 |
| TC01003822.hg.1 | 4.0425 | 0.015572 | 1.8077 | 0.06949 | ESRRG |
| TC20001222.hg.1 | -4.0425 | 0.015572 | 1.8077 | 0.06949 | NA |
| TC01001534.hg.1 | -4.0417 | 0.015583 | 1.8074 | 0.06949 | PAPPA2 |
| TC01000975.hg.1 | -4.035 | 0.015669 | 1.805 | 0.06973 | PIFO |
| TC11001945.hg.1 | -4.0338 | 0.015685 | 1.8045 | 0.06973 | CFL1 |
| TC0X000281.hg.1 | -4.0338 | 0.015685 | 1.8045 | 0.06973 | NA |
| TC15002722.hg.1 | -4.0332 | 0.015692 | 1.8043 | 0.06973 | NR2F2-AS1 |
| 47424020_st | -4.0282 | 0.015757 | 1.8025 | 0.069897 | NA |
| TC11001557.hg.1 | -4.0281 | 0.015758 | 1.8025 | 0.069897 | COMMD9 |
| TC0X002132.hg.1 | -4.0265 | 0.015779 | 1.8019 | 0.06993 | ZDHHC15 |
| TC05000837.hg.1 | 4.023 | 0.015826 | 1.8006 | 0.070073 | GPX3 |
| TC01003412.hg.1 | -4.0153 | 0.015927 | 1.7979 | 0.070335 | TSTD1 |
| TC6_mann_hap4000130.hg.1 | 4.0153 | 0.015927 | 1.7979 | 0.070335 | POU5F1 |
| TC6_mcf_hap5000130.hg.1 | 4.0153 | 0.015927 | 1.7979 | 0.070335 | POU5F1 |
| TC01003613.hg.1 | -4.0132 | 0.015955 | 1.7971 | 0.070395 | NA |
| TC15002279.hg.1 | 4.0113 | 0.015979 | 1.7964 | 0.070442 | NA |
| TC13001682.hg.1 | -4.0067 | 0.01604 | 1.7948 | 0.070647 | NA |
| TC05000866.hg.1 | 4.0003 | 0.016125 | 1.7925 | 0.07096 | CYFIP2 |
| TC13001419.hg.1 | -3.9818 | 0.016376 | 1.7858 | 0.072001 | NA |
| TC02000500.hg.1 | -3.9808 | 0.016391 | 1.7854 | 0.072001 | TMSB10 |
| TC09002799.hg.1 | -3.9787 | 0.016419 | 1.7846 | 0.072063 | FAM129B |
| TC16000947.hg.1 | -3.9747 | 0.016474 | 1.7832 | 0.072238 | NA |
| TC20000833.hg.1 | -3.9733 | 0.016493 | 1.7827 | 0.072259 | TGM2 |
| TC11003053.hg.1 | -3.9664 | 0.016588 | 1.7802 | 0.072613 | NA |
| TC20001671.hg.1 | -3.962 | 0.01665 | 1.7786 | 0.072822 | PMEPA1 |
| PSR14009917.hg.1 | 3.958 | 0.016705 | 1.7772 | 0.072988 | NA |
| TC05001142.hg.1 | -3.9571 | 0.016718 | 1.7768 | 0.072988 | MIR4454 |
| TC0X000986.hg.1 | -3.9451 | 0.016887 | 1.7725 | 0.073606 | MIR222 |
| TC14001255.hg.1 | -3.9446 | 0.016894 | 1.7723 | 0.073606 | ACTN1 |
| TC01004768.hg.1 | 3.944 | 0.016903 | 1.772 | 0.073606 | NA |
| 47424041_st | -3.942 | 0.016931 | 1.7713 | 0.073662 | NA |
| TC07000971.hg.1 | -3.9333 | 0.017055 | 1.7681 | 0.074139 | RNY5 |
| TC05002959.hg.1 | -3.932 | 0.017074 | 1.7677 | 0.074156 | NA |
| TC18000461.hg.1 | -3.9245 | 0.017182 | 1.7649 | 0.074561 | TPGS2 |
| TC01000319.hg.1 | -3.9224 | 0.017213 | 1.7641 | 0.07463 | TMEM50A |
| TC14000409.hg.1 | 3.919 | 0.017262 | 1.7629 | 0.074778 | ARG2 |
| TC11003282.hg.1 | -3.9029 | 0.017499 | 1.757 | 0.075737 | NA |
| TC09002932.hg.1 | 3.8963 | 0.017596 | 1.7546 | 0.076039 | ANKRD20A3 |
| TC12002841.hg.1 | -3.8961 | 0.017599 | 1.7545 | 0.076039 | PRICKLE1 |
| TC11002832.hg.1 | -3.8951 | 0.017614 | 1.7541 | 0.076039 | NA |
| PSR01002146.hg.1 | -3.8912 | 0.017672 | 1.7527 | 0.076223 | NA |
| TC6_qbl_hap6000143.hg.1 | 3.8754 | 0.017911 | 1.7469 | 0.077077 | POU5F1 |
| TC6_ssto_hap7000125.hg.1 | 3.8754 | 0.017911 | 1.7469 | 0.077077 | POU5F1 |
| TC15001606.hg.1 | 3.8751 | 0.017916 | 1.7468 | 0.077077 | TLE3 |
| TC02004860.hg.1 | -3.8678 | 0.018027 | 1.7441 | 0.077487 | NA |
| TC12002057.hg.1 | -3.8586 | 0.018169 | 1.7407 | 0.07803 | HNF1A-AS1 |
| TC13000861.hg.1 | -3.8545 | 0.018233 | 1.7391 | 0.078173 | ARGLU1 |
| TC21000887.hg.1 | -3.8544 | 0.018234 | 1.7391 | 0.078173 | APP |
| TC14001253.hg.1 | 3.85 | 0.018302 | 1.7375 | 0.0784 | ZFP36L1 |
| TC03002006.hg.1 | -3.8484 | 0.018327 | 1.7369 | 0.078441 | TNFSF10 |
| TC03000423.hg.1 | -3.8257 | 0.018687 | 1.7285 | 0.079912 | GPR27 |
| TC02004735.hg.1 | -3.8232 | 0.018727 | 1.7275 | 0.080015 | PDE1A |
| TC08001990.hg.1 | -3.8152 | 0.018856 | 1.7246 | 0.080495 | ZC2HC1A |
| TC14000914.hg.1 | -3.7985 | 0.019128 | 1.7183 | 0.081531 | LINC00641 |
| TC01002374.hg.1 | -3.7983 | 0.019131 | 1.7183 | 0.081531 | RSRP1 |
| TC12002978.hg.1 | -3.796 | 0.019169 | 1.7174 | 0.081624 | LIN7A |
| 3151622_st | -3.7902 | 0.019265 | 1.7152 | 0.081946 | NA |
| TC14001298.hg.1 | -3.7895 | 0.019277 | 1.7149 | 0.081946 | LOC100506498 |
| TC15002198.hg.1 | -3.7865 | 0.019326 | 1.7139 | 0.082084 | TMOD2 |
| TC06001390.hg.1 | -3.7839 | 0.01937 | 1.7129 | 0.082199 | NA |
| TC13000904.hg.1 | -3.7811 | 0.019418 | 1.7118 | 0.082331 | NA |
| TC15001187.hg.1 | -3.7794 | 0.019446 | 1.7112 | 0.08238 | LOC101929988 |
| TC04001130.hg.1 | -3.7653 | 0.019684 | 1.7059 | 0.083252 | UGDH |
| TC01006233.hg.1 | -3.7653 | 0.019685 | 1.7059 | 0.083252 | CHML |
| TC06001912.hg.1 | -3.7625 | 0.019732 | 1.7048 | 0.083296 | ME1 |
| TC01002616.hg.1 | -3.7618 | 0.019743 | 1.7046 | 0.083296 | PIK3R3 |
| TC07003097.hg.1 | -3.7617 | 0.019745 | 1.7045 | 0.083296 | LINC01004 |
| 47420908_st | 3.7545 | 0.01987 | 1.7018 | 0.083643 | NA |
| TC19000542.hg.1 | -3.7541 | 0.019877 | 1.7017 | 0.083643 | NA |
| PSR05015308.hg.1 | -3.754 | 0.019878 | 1.7016 | 0.083643 | NA |
| TC09000186.hg.1 | -3.7424 | 0.02008 | 1.6972 | 0.084371 | RPL36AP33 |
| TC02002590.hg.1 | -3.7421 | 0.020084 | 1.6971 | 0.084371 | PDE1A |
| 3706893_st | -3.7381 | 0.020155 | 1.6956 | 0.084597 | NA |
| TC05001853.hg.1 | -3.7368 | 0.020177 | 1.6951 | 0.084617 | PFDN1 |
| TC01000730.hg.1 | -3.7349 | 0.020211 | 1.6944 | 0.084688 | LEPR |
| TC10002624.hg.1 | -3.7323 | 0.020256 | 1.6935 | 0.084806 | CTGLF12P |
| TC16000540.hg.1 | 3.7275 | 0.020341 | 1.6916 | 0.085066 | HSD11B2 |
| TC21000186.hg.1 | 3.7269 | 0.020352 | 1.6914 | 0.085066 | NA |
| TC09000859.hg.1 | -3.7237 | 0.020408 | 1.6902 | 0.085176 | WASH1 |
| TC21000730.hg.1 | 3.7235 | 0.020412 | 1.6901 | 0.085176 | B3GALT5 |
| TC11002877.hg.1 | -3.7147 | 0.02057 | 1.6868 | 0.085764 | NA |
| TC0X001584.hg.1 | -3.7091 | 0.02067 | 1.6847 | 0.086106 | STS |
| TC01003094.hg.1 | -3.7022 | 0.020795 | 1.6821 | 0.086554 | LOC727820 |
| PSR05015351.hg.1 | -3.6972 | 0.020887 | 1.6801 | 0.086644 | NA |
| TC6_dbb_hap3000142.hg.1 | 3.6962 | 0.020906 | 1.6797 | 0.086644 | POU5F1 |
| TC07002489.hg.1 | 3.6948 | 0.02093 | 1.6792 | 0.086644 | STAG3L5P-PVRIG2P-PILRB |
| TC13001246.hg.1 | -3.6907 | 0.021006 | 1.6777 | 0.086644 | NA |
| TC01001107.hg.1 | -3.6893 | 0.021032 | 1.6771 | 0.086644 | NA |
| TC01001129.hg.1 | -3.6893 | 0.021032 | 1.6771 | 0.086644 | NA |
| TC01003105.hg.1 | -3.6893 | 0.021032 | 1.6771 | 0.086644 | NA |
| TC01003140.hg.1 | -3.6893 | 0.021032 | 1.6771 | 0.086644 | NA |
| TC06000228.hg.1 | -3.6893 | 0.021032 | 1.6771 | 0.086644 | NA |
| TC09000917.hg.1 | -3.6893 | 0.021032 | 1.6771 | 0.086644 | NA |
| TC15001309.hg.1 | -3.6893 | 0.021032 | 1.6771 | 0.086644 | NA |
| TC15001310.hg.1 | -3.6893 | 0.021032 | 1.6771 | 0.086644 | NA |
| 47420669_st | 3.6888 | 0.021041 | 1.6769 | 0.086644 | NA |
| 3374465_st | -3.6852 | 0.021108 | 1.6756 | 0.086847 | NA |
| TC04002517.hg.1 | -3.6834 | 0.02114 | 1.6749 | 0.086908 | UGDH |
| PSR03025028.hg.1 | -3.6762 | 0.021275 | 1.6721 | 0.087391 | NA |
| TC05000941.hg.1 | -3.6728 | 0.021339 | 1.6708 | 0.087582 | NA |
| PSR16011591.hg.1 | -3.6649 | 0.021487 | 1.6678 | 0.088117 | NA |
| TC06000628.hg.1 | 3.6611 | 0.02156 | 1.6663 | 0.088272 | SLC25A27 |
| TC16000560.hg.1 | -3.6611 | 0.02156 | 1.6663 | 0.088272 | NA |
| TC05003299.hg.1 | -3.6514 | 0.021745 | 1.6626 | 0.088957 | NA |
| TC15000052.hg.1 | -3.64 | 0.021964 | 1.6583 | 0.089778 | SNORD116-2 |
| TC07001836.hg.1 | -3.6378 | 0.022007 | 1.6574 | 0.089879 | NA |
| TC01002760.hg.1 | -3.6279 | 0.022201 | 1.6536 | 0.0906 | GNG12 |
| TC06002089.hg.1 | -3.6247 | 0.022263 | 1.6524 | 0.090707 | NA |
| TC10001691.hg.1 | -3.6247 | 0.022264 | 1.6524 | 0.090707 | RAB11FIP2 |
| TC0X002324.hg.1 | -3.6145 | 0.022466 | 1.6485 | 0.091434 | WASH1 |
| TC18000458.hg.1 | -3.6139 | 0.022479 | 1.6482 | 0.091434 | SLC39A6 |
| TC06001419.hg.1 | -3.6124 | 0.022509 | 1.6476 | 0.091471 | NA |
| PSR14010168.hg.1 | -3.6116 | 0.022525 | 1.6473 | 0.091471 | NA |
| TC07001581.hg.1 | -3.609 | 0.022576 | 1.6463 | 0.091606 | SRI |
| TC11003270.hg.1 | -3.6055 | 0.022647 | 1.645 | 0.09182 | RAB30 |
| TC02003869.hg.1 | 3.6011 | 0.022737 | 1.6433 | 0.092035 | NA |
| TC05001233.hg.1 | -3.601 | 0.022737 | 1.6433 | 0.092035 | MTMR12 |
| TC10002687.hg.1 | -3.5978 | 0.022803 | 1.642 | 0.092226 | NA |
| 47419414_st | -3.5938 | 0.022885 | 1.6405 | 0.092483 | NA |
| TC18000910.hg.1 | 3.5915 | 0.022931 | 1.6396 | 0.092596 | NA |
| TC0Y000323.hg.1 | -3.5887 | 0.022989 | 1.6385 | 0.092753 | NA |
| TC05002422.hg.1 | -3.585 | 0.023065 | 1.637 | 0.092987 | SMA4 |
| TC20001747.hg.1 | -3.5819 | 0.023128 | 1.6359 | 0.093119 | EIF6 |
| TC04002024.hg.1 | -3.5816 | 0.023135 | 1.6357 | 0.093119 | NA |
| TC07003313.hg.1 | 3.5789 | 0.02319 | 1.6347 | 0.093265 | PILRB |
| TC11002689.hg.1 | -3.5745 | 0.023281 | 1.633 | 0.093555 | STX3 |
| TC05000372.hg.1 | -3.5722 | 0.02333 | 1.6321 | 0.093677 | F2RL1 |
| TC07000578.hg.1 | -3.5616 | 0.023553 | 1.628 | 0.094496 | SDHAF3 |
| TC07001167.hg.1 | -3.5571 | 0.023648 | 1.6262 | 0.094769 | NA |
| TC10001228.hg.1 | -3.5566 | 0.023659 | 1.626 | 0.094769 | AGAP4 |
| TC17000877.hg.1 | -3.5551 | 0.02369 | 1.6254 | 0.094806 | SNORD1A |
| TC09000998.hg.1 | -3.5543 | 0.023706 | 1.6251 | 0.094806 | NA |
| TC07001994.hg.1 | -3.5491 | 0.023818 | 1.6231 | 0.095178 | RNY1 |
| TC16000455.hg.1 | -3.5388 | 0.02404 | 1.6191 | 0.095987 | LPCAT2 |
| TC15000548.hg.1 | -3.5363 | 0.024094 | 1.6181 | 0.096128 | NA |
| TC01005005.hg.1 | -3.5234 | 0.024375 | 1.6131 | 0.09717 | NA |
| TC06001414.hg.1 | -3.5158 | 0.024543 | 1.6101 | 0.09776 | NA |
| TC02001766.hg.1 | -3.5148 | 0.024566 | 1.6097 | 0.097775 | NA |
| TC14000386.hg.1 | 3.5066 | 0.024748 | 1.6065 | 0.098397 | SYNE2 |
| TC15000513.hg.1 | -3.506 | 0.024762 | 1.6062 | 0.098397 | NA |
| TC13000585.hg.1 | -3.5008 | 0.024877 | 1.6042 | 0.098778 | LHFP |
| TC19001584.hg.1 | -3.4884 | 0.02516 | 1.5993 | 0.099813 | PSG9 |
| TC06001692.hg.1 | -3.4876 | 0.025178 | 1.599 | 0.099813 | GLO1 |
| TC02000315.hg.1 | 3.4821 | 0.025305 | 1.5968 | 0.10024 | SPTBN1 |
| 3183494_st | -3.4805 | 0.02534 | 1.5962 | 0.1003 | NA |
| TC18000555.hg.1 | -3.4784 | 0.025388 | 1.5954 | 0.10041 | NA |
| TC16000891.hg.1 | -3.4729 | 0.025517 | 1.5932 | 0.10084 | NTAN1 |
| TC10001281.hg.1 | -3.4687 | 0.025615 | 1.5915 | 0.10113 | AGAP7P |
| TC16000879.hg.1 | -3.468 | 0.02563 | 1.5912 | 0.10113 | CPPED1 |
| TC16001084.hg.1 | -3.4657 | 0.025685 | 1.5903 | 0.10126 | VPS35 |
| TC02004165.hg.1 | 3.4607 | 0.025801 | 1.5884 | 0.10164 | NA |
| TC08000125.hg.1 | -3.4591 | 0.025839 | 1.5877 | 0.10166 | ZDHHC2 |
| TC02001613.hg.1 | 3.4587 | 0.025848 | 1.5876 | 0.10166 | TTC32 |
| TC11003369.hg.1 | 3.4565 | 0.0259 | 1.5867 | 0.10179 | NA |
| TC09000222.hg.1 | -3.4492 | 0.026073 | 1.5838 | 0.10239 | ALDH1B1 |
| TC06000278.hg.1 | -3.4407 | 0.026277 | 1.5804 | 0.10311 | NA |
| TC03000714.hg.1 | -3.4387 | 0.026324 | 1.5797 | 0.10321 | ACPP |
| TC20000047.hg.1 | 3.4353 | 0.026407 | 1.5783 | 0.10346 | FTLP3 |
| TC02003411.hg.1 | -3.4293 | 0.026553 | 1.5759 | 0.10395 | NA |
| TC12000642.hg.1 | -3.4219 | 0.026733 | 1.5729 | 0.10457 | GLIPR1 |
| TC07003061.hg.1 | -3.4173 | 0.026847 | 1.5711 | 0.10493 | CDK6 |
| TC14001176.hg.1 | -3.4126 | 0.026962 | 1.5692 | 0.1053 | NA |
| PSR14009919.hg.1 | 3.3991 | 0.027301 | 1.5638 | 0.10654 | NA |
| 47422060_st | -3.3975 | 0.02734 | 1.5632 | 0.10661 | NA |
| TC06002113.hg.1 | -3.3818 | 0.027739 | 1.5569 | 0.10808 | MOXD1 |
| TC4_ctg9_hap1000005.hg.1 | -3.3799 | 0.027787 | 1.5562 | 0.10818 | UGT2A3 |
| TC04000892.hg.1 | -3.3773 | 0.027856 | 1.5551 | 0.10837 | NA |
| 2928559_st | -3.3745 | 0.027928 | 1.554 | 0.10849 | NA |
| TC12002571.hg.1 | -3.3743 | 0.027931 | 1.5539 | 0.10849 | PTPN11 |
| TC02002488.hg.1 | -3.3718 | 0.027996 | 1.5529 | 0.10866 | SNORA70F |
| TC16001761.hg.1 | 3.3709 | 0.028021 | 1.5525 | 0.10867 | NA |
| TC15001104.hg.1 | 3.3673 | 0.028113 | 1.5511 | 0.10894 | GOLGA8IP |
| TC05002135.hg.1 | -3.3657 | 0.028156 | 1.5504 | 0.10902 | HNRNPH1 |
| PSR03025023.hg.1 | -3.3598 | 0.028309 | 1.5481 | 0.10953 | NA |
| TC14001917.hg.1 | -3.3511 | 0.028541 | 1.5445 | 0.11034 | LINC00641 |
| TC06001410.hg.1 | -3.3482 | 0.028616 | 1.5434 | 0.11055 | NA |
| TC19001802.hg.1 | -3.3441 | 0.028727 | 1.5417 | 0.11089 | ZNF83 |
| TC13000924.hg.1 | 3.3394 | 0.028852 | 1.5398 | 0.11129 | NA |
| PSR16011592.hg.1 | -3.3354 | 0.028959 | 1.5382 | 0.11162 | NA |
| TC06000451.hg.1 | 3.3326 | 0.029035 | 1.5371 | 0.11182 | NA |
| 47423391_st | -3.3262 | 0.029209 | 1.5345 | 0.11241 | NA |
| TC02003679.hg.1 | -3.3222 | 0.029317 | 1.5329 | 0.11274 | NA |
| TC14002082.hg.1 | -3.3189 | 0.02941 | 1.5315 | 0.11301 | LOC100506498 |
| TC13000282.hg.1 | -3.3167 | 0.02947 | 1.5306 | 0.11315 | NA |
| TC03003057.hg.1 | -3.3107 | 0.029634 | 1.5282 | 0.11369 | MYLK |
| TC0X001158.hg.1 | -3.3099 | 0.029658 | 1.5279 | 0.1137 | MIR421 |
| TC06003119.hg.1 | 3.2951 | 0.03007 | 1.5219 | 0.11509 | SAMD5 |
| TC19000814.hg.1 | -3.2948 | 0.030081 | 1.5217 | 0.11509 | NA |
| TC05001824.hg.1 | -3.2944 | 0.030091 | 1.5216 | 0.11509 | NME5 |
| TC07000562.hg.1 | 3.2907 | 0.030194 | 1.5201 | 0.1154 | PEG10 |
| TC13000878.hg.1 | -3.2848 | 0.030364 | 1.5176 | 0.11596 | ANKRD10 |
| TC09002694.hg.1 | -3.2795 | 0.030514 | 1.5155 | 0.11644 | NA |
| TC0X000425.hg.1 | -3.2783 | 0.030549 | 1.515 | 0.11649 | PGK1 |
| PSR05015329.hg.1 | -3.2738 | 0.03068 | 1.5131 | 0.1169 | NA |
| TC06000987.hg.1 | 3.2671 | 0.030872 | 1.5104 | 0.11744 | NA |
| TC04000615.hg.1 | -3.2667 | 0.030884 | 1.5103 | 0.11744 | NA |
| TC0X001982.hg.1 | -3.2664 | 0.030893 | 1.5101 | 0.11744 | NA |
| TC09002933.hg.1 | 3.2518 | 0.031323 | 1.5041 | 0.11898 | LOC101060026 |
| TC15000348.hg.1 | -3.2493 | 0.031398 | 1.5031 | 0.11918 | NA |
| TC15001949.hg.1 | 3.2478 | 0.03144 | 1.5025 | 0.11925 | NA |
| TC12002874.hg.1 | 3.2467 | 0.031473 | 1.5021 | 0.11928 | NA |
| TC02004559.hg.1 | 3.2394 | 0.031692 | 1.499 | 0.12002 | NA |
| TC12000491.hg.1 | -3.2368 | 0.031771 | 1.498 | 0.12023 | ORMDL2 |
| TC07000595.hg.1 | -3.2334 | 0.031872 | 1.4966 | 0.12052 | NA |
| TC06002157.hg.1 | 3.2311 | 0.031943 | 1.4956 | 0.1207 | PERP |
| 47420982_st | 3.2255 | 0.032113 | 1.4933 | 0.12125 | NA |
| PSR03025029.hg.1 | -3.2199 | 0.032284 | 1.491 | 0.1218 | NA |
| PSR12005963.hg.1 | -3.2183 | 0.032332 | 1.4904 | 0.12189 | NA |
| TC01000099.hg.1 | -3.217 | 0.032374 | 1.4898 | 0.12196 | VAMP3 |
| TC04000636.hg.1 | 3.2087 | 0.032628 | 1.4864 | 0.12282 | FAT4 |
| TC02004595.hg.1 | -3.2069 | 0.032686 | 1.4856 | 0.12295 | NA |
| TC01001881.hg.1 | -3.2051 | 0.03274 | 1.4849 | 0.12297 | NA |
| TC01003854.hg.1 | -3.2051 | 0.03274 | 1.4849 | 0.12297 | NA |
| TC10000517.hg.1 | 3.2011 | 0.032865 | 1.4833 | 0.12334 | NA |
| TC12003121.hg.1 | -3.1889 | 0.033251 | 1.4782 | 0.1247 | NA |
| TC01003692.hg.1 | -3.1718 | 0.033799 | 1.4711 | 0.12666 | CSRP1 |
| TC07001659.hg.1 | 3.1706 | 0.033839 | 1.4706 | 0.12671 | NA |
| 47424353_st | -3.1673 | 0.033945 | 1.4692 | 0.12702 | NA |
| TC07003298.hg.1 | -3.1557 | 0.034324 | 1.4644 | 0.12833 | TRIM74 |
| TC07002713.hg.1 | -3.154 | 0.034383 | 1.4637 | 0.12833 | NA |
| TC15000069.hg.1 | -3.1526 | 0.034427 | 1.4631 | 0.12833 | SNORD116-26 |
| TC09000007.hg.1 | -3.1526 | 0.034428 | 1.4631 | 0.12833 | NA |
| TC08000463.hg.1 | -3.1526 | 0.034429 | 1.4631 | 0.12833 | NA |
| TC0Y000356.hg.1 | -3.1519 | 0.034449 | 1.4628 | 0.12833 | WASH1 |
| TC16001588.hg.1 | 3.1423 | 0.03477 | 1.4588 | 0.12942 | NA |
| TC16000894.hg.1 | -3.1392 | 0.034875 | 1.4575 | 0.12971 | PKD1P6 |
| TC01001201.hg.1 | -3.1385 | 0.034898 | 1.4572 | 0.12971 | MLLT11 |
| TC07002999.hg.1 | -3.1374 | 0.034934 | 1.4568 | 0.12972 | NA |
| TC15000064.hg.1 | -3.1361 | 0.03498 | 1.4562 | 0.12972 | SNORD116-18 |
| TC01004414.hg.1 | -3.1361 | 0.03498 | 1.4562 | 0.12972 | PRKAA2 |
| TC22001175.hg.1 | -3.1304 | 0.035173 | 1.4538 | 0.13034 | NA |
| 3332060_st | -3.1265 | 0.035304 | 1.4522 | 0.1307 | NA |
| TC0X001776.hg.1 | 3.1258 | 0.035328 | 1.4519 | 0.1307 | NA |
| TC08001678.hg.1 | 3.1252 | 0.035347 | 1.4516 | 0.1307 | NA |
| TC01005764.hg.1 | -3.1243 | 0.035379 | 1.4512 | 0.13072 | NA |
| TC16000105.hg.1 | -3.1214 | 0.035479 | 1.45 | 0.13097 | NA |
| 3597908_st | -3.1204 | 0.035512 | 1.4496 | 0.13097 | NA |
| TC06001427.hg.1 | -3.12 | 0.035526 | 1.4495 | 0.13097 | HIST1H3I |
| 47422085_st | -3.1185 | 0.035579 | 1.4488 | 0.13103 | NA |
| TC01001902.hg.1 | -3.118 | 0.035594 | 1.4486 | 0.13103 | NA |
| PSR05015353.hg.1 | -3.1159 | 0.035668 | 1.4477 | 0.1312 | NA |
| TC19002036.hg.1 | -3.1136 | 0.035748 | 1.4468 | 0.1314 | NA |
| TC02004558.hg.1 | 3.1064 | 0.035997 | 1.4437 | 0.13222 | NA |
| TC09002457.hg.1 | -3.102 | 0.03615 | 1.4419 | 0.13268 | NA |
| TC15000647.hg.1 | -3.1004 | 0.036207 | 1.4412 | 0.13279 | LRRC49 |
| TC12000565.hg.1 | -3.0921 | 0.036499 | 1.4377 | 0.13377 | NA |
| TC05001314.hg.1 | -3.0911 | 0.036536 | 1.4373 | 0.13381 | NA |
| TC01003014.hg.1 | -3.0884 | 0.036632 | 1.4361 | 0.13406 | NRAS |
| TC01002299.hg.1 | -3.0835 | 0.036805 | 1.4341 | 0.13459 | NA |
| TC15002176.hg.1 | -3.0802 | 0.036924 | 1.4327 | 0.13493 | NA |
| TC15000062.hg.1 | -3.0792 | 0.036961 | 1.4323 | 0.13496 | SNORD116-16 |
| TC14001140.hg.1 | 3.0759 | 0.037081 | 1.4309 | 0.1353 | TXNDC16 |
| TC19000093.hg.1 | -3.072 | 0.03722 | 1.4292 | 0.13572 | NA |
| TC01005808.hg.1 | 3.071 | 0.037257 | 1.4288 | 0.13575 | NA |
| TC12002424.hg.1 | -3.0701 | 0.037288 | 1.4284 | 0.13576 | HMGA2 |
| TC01002925.hg.1 | -3.0677 | 0.037377 | 1.4274 | 0.13597 | NA |
| TC16001163.hg.1 | -3.0671 | 0.0374 | 1.4271 | 0.13597 | RNU6-21P |
| TC06001338.hg.1 | -3.0654 | 0.037459 | 1.4264 | 0.13609 | SLC17A3 |
| TC05003053.hg.1 | -3.064 | 0.037511 | 1.4258 | 0.13618 | NA |
| TC19000434.hg.1 | -3.0613 | 0.03761 | 1.4247 | 0.13644 | NA |
| PSR05015297.hg.1 | -3.0567 | 0.037779 | 1.4228 | 0.13695 | NA |
| TC11001107.hg.1 | 3.0521 | 0.037949 | 1.4208 | 0.13747 | SORL1 |
| TC0Y000089.hg.1 | -3.047 | 0.038138 | 1.4186 | 0.13796 | NA |
| TC0Y000215.hg.1 | -3.047 | 0.038138 | 1.4186 | 0.13796 | NA |
| TC11000277.hg.1 | -3.0446 | 0.03823 | 1.4176 | 0.13819 | FIBIN |
| PSR03025032.hg.1 | -3.0355 | 0.038571 | 1.4137 | 0.13932 | NA |
| TC13001526.hg.1 | 3.0347 | 0.038603 | 1.4134 | 0.13933 | NA |
| TC6_cox_hap2000153.hg.1 | 3.0333 | 0.038656 | 1.4128 | 0.13942 | POU5F1 |
| TC05002956.hg.1 | -3.0256 | 0.038948 | 1.4095 | 0.14035 | NA |
| TC07001907.hg.1 | 3.0251 | 0.038969 | 1.4093 | 0.14035 | KIAA1549 |
| TC07000718.hg.1 | -3.0182 | 0.039231 | 1.4064 | 0.14116 | TES |
| 3064820_st | -3.0177 | 0.039253 | 1.4061 | 0.14116 | NA |
| TC0X002127.hg.1 | -3.017 | 0.039278 | 1.4058 | 0.14116 | FTX |
| TC19001337.hg.1 | -3.0163 | 0.039307 | 1.4055 | 0.14116 | NA |
| TC14001118.hg.1 | 3.0125 | 0.039453 | 1.4039 | 0.14143 | NA |
| TC14001400.hg.1 | 3.0117 | 0.039485 | 1.4036 | 0.14143 | NA |
| TC0X001156.hg.1 | -3.0112 | 0.039505 | 1.4033 | 0.14143 | FTX |
| TC03000968.hg.1 | -3.011 | 0.03951 | 1.4033 | 0.14143 | NA |
| TC14002091.hg.1 | -3.0107 | 0.039522 | 1.4032 | 0.14143 | NA |
| TC02002861.hg.1 | 3.0076 | 0.039643 | 1.4018 | 0.14176 | SNORD20 |
| TC19002007.hg.1 | 3.0041 | 0.039781 | 1.4003 | 0.14215 | NA |
| 47419564_st | -2.9956 | 0.040117 | 1.3967 | 0.14325 | NA |
| TC05002504.hg.1 | -2.9925 | 0.04024 | 1.3953 | 0.14358 | NA |
| TC06000239.hg.1 | -2.9847 | 0.040552 | 1.392 | 0.14459 | NA |
| TC11001142.hg.1 | -2.9707 | 0.041115 | 1.386 | 0.14649 | SPA17 |
| TC15001667.hg.1 | -2.9688 | 0.041193 | 1.3852 | 0.14663 | PTPN9 |
| TC03003211.hg.1 | -2.9684 | 0.04121 | 1.385 | 0.14663 | TNFSF10 |
| PSR03025031.hg.1 | -2.9671 | 0.041262 | 1.3845 | 0.14671 | NA |
| TC07001043.hg.1 | 2.9654 | 0.041331 | 1.3837 | 0.14682 | RBM33 |
| TC07000547.hg.1 | -2.9649 | 0.041352 | 1.3835 | 0.14682 | ANKIB1 |
| TC09001175.hg.1 | -2.964 | 0.041388 | 1.3831 | 0.14684 | NA |
| TC11003188.hg.1 | -2.9537 | 0.041816 | 1.3787 | 0.14824 | NA |
| TC04001263.hg.1 | -2.9531 | 0.041841 | 1.3784 | 0.14824 | UGT2A3 |
| TC09000460.hg.1 | -2.9465 | 0.042116 | 1.3755 | 0.14911 | NA |
| TC14000003.hg.1 | -2.9445 | 0.0422 | 1.3747 | 0.14919 | NA |
| TC22000459.hg.1 | -2.9445 | 0.0422 | 1.3747 | 0.14919 | NA |
| TC22000414.hg.1 | -2.9402 | 0.042379 | 1.3729 | 0.14972 | NA |
| 2924327_st | -2.9361 | 0.042555 | 1.3711 | 0.15023 | NA |
| TC15000057.hg.1 | -2.9345 | 0.042624 | 1.3703 | 0.15037 | SNORD116-11 |
| TC16000595.hg.1 | -2.9288 | 0.042864 | 1.3679 | 0.15111 | NA |
| TC11001660.hg.1 | 2.9267 | 0.042957 | 1.367 | 0.15132 | NA |
| TC01003157.hg.1 | -2.9261 | 0.042983 | 1.3667 | 0.15132 | NA |
| TC07001470.hg.1 | -2.9243 | 0.04306 | 1.3659 | 0.15148 | POLR2J4 |
| TC17000618.hg.1 | 2.9232 | 0.043106 | 1.3655 | 0.15154 | CDK5RAP3 |
| 47423590_st | -2.9206 | 0.043217 | 1.3643 | 0.15178 | NA |
| PSR14010170.hg.1 | -2.9202 | 0.043237 | 1.3641 | 0.15178 | NA |
| TC22001370.hg.1 | -2.9178 | 0.043339 | 1.3631 | 0.15204 | TTLL1 |
| TC20000613.hg.1 | 2.9169 | 0.043379 | 1.3627 | 0.15207 | NA |
| TC06000222.hg.1 | -2.9154 | 0.043443 | 1.3621 | 0.15219 | NA |
| TC14000805.hg.1 | -2.9138 | 0.043514 | 1.3614 | 0.15233 | NA |
| TC11002594.hg.1 | -2.913 | 0.043549 | 1.361 | 0.15234 | NAV2 |
| TC16000893.hg.1 | -2.9102 | 0.04367 | 1.3598 | 0.15266 | PKD1P6 |
| TC17000639.hg.1 | -2.9073 | 0.043795 | 1.3586 | 0.15299 | NA |
| TC16000236.hg.1 | -2.9065 | 0.043833 | 1.3582 | 0.15302 | NA |
| TC19001983.hg.1 | 2.9031 | 0.04398 | 1.3567 | 0.15342 | NA |
| TC03000367.hg.1 | -2.8941 | 0.04438 | 1.3528 | 0.15471 | NA |
| TC07001619.hg.1 | -2.8921 | 0.044467 | 1.352 | 0.15488 | SLC25A13 |
| TC16000224.hg.1 | 2.8916 | 0.044491 | 1.3517 | 0.15488 | NA |
| TC03000636.hg.1 | 2.8898 | 0.04457 | 1.351 | 0.15505 | DIRC2 |
| TC04001810.hg.1 | 2.8891 | 0.044603 | 1.3506 | 0.15506 | SLED1 |
| TC05002125.hg.1 | -2.8866 | 0.044716 | 1.3495 | 0.15532 | CLK4 |
| TC0X000587.hg.1 | -2.886 | 0.044741 | 1.3493 | 0.15532 | UBE2A |
| 47419399_st | 2.8837 | 0.044848 | 1.3483 | 0.15558 | NA |
| TC18000344.hg.1 | -2.8784 | 0.045088 | 1.3459 | 0.15626 | NA |
| TC03003002.hg.1 | -2.878 | 0.045104 | 1.3458 | 0.15626 | DCBLD2 |
| 47420654_st | 2.8728 | 0.045339 | 1.3435 | 0.15696 | NA |
| TC02004976.hg.1 | -2.8717 | 0.045389 | 1.3431 | 0.15702 | IGKV1D-33 |
| TC22000791.hg.1 | -2.8646 | 0.045717 | 1.3399 | 0.15804 | DDX17 |
| TC01004846.hg.1 | -2.864 | 0.045745 | 1.3397 | 0.15804 | NA |
| TC14001181.hg.1 | -2.8626 | 0.045808 | 1.3391 | 0.15814 | GPR135 |
| TC06001348.hg.1 | -2.8619 | 0.045842 | 1.3387 | 0.15815 | HIST1H4D |
| TC09001085.hg.1 | -2.8603 | 0.045915 | 1.338 | 0.15829 | NA |
| TC14001393.hg.1 | 2.8587 | 0.045988 | 1.3374 | 0.15844 | NA |
| TC05003354.hg.1 | -2.8515 | 0.046324 | 1.3342 | 0.15949 | LOC100130394 |
| TC01004689.hg.1 | -2.8482 | 0.046479 | 1.3327 | 0.15991 | NA |
| PSR16017891.hg.1 | -2.8452 | 0.04662 | 1.3314 | 0.16029 | NA |
| TC10000479.hg.1 | -2.8443 | 0.046665 | 1.331 | 0.16033 | ADK |
| TC13000715.hg.1 | -2.8432 | 0.046717 | 1.3305 | 0.1604 | HNRNPA3P5 |
| TC06003615.hg.1 | -2.8408 | 0.046827 | 1.3295 | 0.16067 | NA |
| TC12000220.hg.1 | -2.8386 | 0.046933 | 1.3285 | 0.16092 | NA |
| TC01002627.hg.1 | -2.8369 | 0.047013 | 1.3278 | 0.16108 | PDZK1IP1 |
| TC0X001165.hg.1 | -2.8303 | 0.047332 | 1.3248 | 0.16206 | ZDHHC15 |
| TC06003777.hg.1 | -2.8287 | 0.047405 | 1.3242 | 0.1622 | NA |
| TC01002648.hg.1 | 2.8272 | 0.047479 | 1.3235 | 0.16234 | TTC39A |
| TC13000716.hg.1 | 2.8232 | 0.047675 | 1.3217 | 0.1629 | NA |
| TC09001119.hg.1 | 2.8137 | 0.048137 | 1.3175 | 0.16426 | NA |
| TC09001146.hg.1 | 2.8137 | 0.048137 | 1.3175 | 0.16426 | NA |
| TC08001692.hg.1 | 2.8114 | 0.048247 | 1.3165 | 0.16444 | NA |
| TC20000424.hg.1 | -2.8106 | 0.048287 | 1.3162 | 0.16444 | DOK5 |
| TC05001505.hg.1 | -2.8101 | 0.048311 | 1.316 | 0.16444 | SNORA47 |
| TC12000504.hg.1 | -2.8099 | 0.048323 | 1.3158 | 0.16444 | ESYT1 |
| TC08000767.hg.1 | -2.8079 | 0.04842 | 1.315 | 0.16466 | EFR3A |
| 3475282_st | -2.8034 | 0.048643 | 1.313 | 0.1652 | NA |
| 47420088_st | -2.8026 | 0.048685 | 1.3126 | 0.1652 | NA |
| TC09002454.hg.1 | 2.802 | 0.048711 | 1.3124 | 0.1652 | NA |
| TC01005585.hg.1 | -2.8012 | 0.048751 | 1.312 | 0.1652 | NA |
| TC18000142.hg.1 | -2.8009 | 0.048768 | 1.3119 | 0.1652 | MAPRE2 |
| TC08001922.hg.1 | -2.8006 | 0.048782 | 1.3117 | 0.1652 | NA |
| TC10001393.hg.1 | -2.8 | 0.048809 | 1.3115 | 0.1652 | MIR1256 |
| TC07002714.hg.1 | -2.7978 | 0.048922 | 1.3105 | 0.16537 | NA |
| TC13000707.hg.1 | -2.7977 | 0.048925 | 1.3105 | 0.16537 | PCDH20 |
| TC02004779.hg.1 | 2.7967 | 0.048977 | 1.31 | 0.16541 | NA |
| TC09000302.hg.1 | 2.7961 | 0.049005 | 1.3098 | 0.16541 | NA |
| TC01000581.hg.1 | 2.7923 | 0.049197 | 1.3081 | 0.16595 | LURAP1 |
