# Supplementary material for: Role of HRTPT in kidney proximal epithelial cell regeneration: Integrative differential expression and pathway analyses using microarray and scRNA‐seq
Source: J Cell Mol Med. 2021 Oct 9;25(22):10466–79. doi: 10.1111/jcmm.16976 (PMC8581341; doi:10.1111/jcmm.16976)
Supplement: Supplementary file 9 — Table S4. A list of HREC24T transport Genes [file JCMM-25-10466-s005.docx]

| **Table S4**. HREC24T Transport Genes | | | |
| --- | --- | --- | --- |
| Gene | Expression vs HRTPT | kidney in situ** | function |
| SLC20A1 | 43.5 | Low | Sodium-Phosphate Symporter |
| SLC47A1 | 37.0 | Medium | Renal Organic Cation Transporter |
| SLC6AJ3 | 35.0 | Medium* | Sodium- and chloride-dependent GABA transporter 2 |
| SLC5A3 | 31.0 | Low | Sodium/myo-inositol cotransporter |
| SLC7A5 | 20.0 | Low | Large neutral amino acids transporter small subunit 1 |
| SLC7A2 | 20.0 | Medium | Cationic Amino Acid Transporter -arg, lys, orn |
| SLC25A29 | 18.5 | Low | Mitochondrial Transporter |
| SLC3A2 | 15.2 | High | Regulation of Ca levels and Transports L-Type AA |
| SLC43A1 | 13.8 | Medium | Transport Large Neutral Amino Acids |
| SLC16A4 | 12.3 | Medium | Monocarboxylate transporter 5 |
| SLC6AI2 | 10.5 | Low | Sodium- and chloride-dependent betaine transporter |
| SLC7A2 | 10.4 | Medium | Cationic Amino Acid Transporter |
| SLC8A1 | 9.7 | Low | Sodium/Calcium Exchanger |
| SLC9A1 | 8.3 | Low | Sodium/hydrogen exchanger 1 |
| SLC7A11 | 8.2 | Low | Cystine/glutamate transporter |
| SLC44A1 | 6.7 | Low | Choline transporter-like protein 1 |
| SLC44A5 | 6.6 | Low | Choline transporter-like protein 5 |
