# Supplementary material for: Role of HRTPT in kidney proximal epithelial cell regeneration: Integrative differential expression and pathway analyses using microarray and scRNA‐seq
Source: J Cell Mol Med. 2021 Oct 9;25(22):10466–79. doi: 10.1111/jcmm.16976 (PMC8581341; doi:10.1111/jcmm.16976)
Supplement: Supplementary file 10 — Table S5. A list of 3,948 genes with P‐value <0.05 and other statistical measurements that were differently expressed between CD133+ versus CD133‐ cell fraction from infant kidney biopsies [file JCMM-25-10466-s001.docx]

| Probes | t.stat | p.value | X.log10.p. | FDR | Symbol |
| --- | --- | --- | --- | --- | --- |
| 9388_at | -166.27 | 7.85E-09 | 8.1052 | 2.46E-05 | LIPG |
| 7111_at | -157.06 | 9.86E-09 | 8.0062 | 2.46E-05 | TMOD1 |
| 5080_at | 114.29 | 3.51E-08 | 7.4541 | 4.45E-05 | PAX6 |
| 54796_at | 105.62 | 4.82E-08 | 7.3171 | 4.45E-05 | BNC2 |
| 307_at | 104.62 | 5.01E-08 | 7.3006 | 4.45E-05 | ANXA4 |
| 6514_at | 102.92 | 5.34E-08 | 7.2721 | 4.45E-05 | SLC2A2 |
| 6519_at | 95.695 | 7.15E-08 | 7.1457 | 5.11E-05 | SLC3A1 |
| 25984_at | -74.253 | 1.97E-07 | 6.7052 | 0.00010893 | KRT23 |
| 3918_at | -74.041 | 1.99E-07 | 6.7003 | 0.00010893 | LAMC2 |
| 648987_at | 67.962 | 2.81E-07 | 6.5515 | 0.00010893 | LOC648987 |
| 2028_at | 67.867 | 2.82E-07 | 6.5491 | 0.00010893 | ENPEP |
| 10786_at | 67.787 | 2.84E-07 | 6.5471 | 0.00010893 | SLC17A3 |
| 120224_at | -63.878 | 3.60E-07 | 6.444 | 0.00010893 | TMEM45B |
| 256764_at | 63.832 | 3.61E-07 | 6.4427 | 0.00010893 | WDR72 |
| 999_at | -62.708 | 3.87E-07 | 6.4119 | 0.00010893 | CDH1 |
| 3694_at | -61.531 | 4.18E-07 | 6.379 | 0.00010893 | ITGB6 |
| 1366_at | -60.513 | 4.47E-07 | 6.35 | 0.00010893 | CLDN7 |
| 27071_at | -60.216 | 4.56E-07 | 6.3415 | 0.00010893 | DAPP1 |
| 10568_at | 59.394 | 4.81E-07 | 6.3176 | 0.00010893 | SLC34A2 |
| 635_at | 58.727 | 5.03E-07 | 6.298 | 0.00010893 | BHMT |
| 57605_at | 58.592 | 5.08E-07 | 6.294 | 0.00010893 | PITPNM2 |
| 6588_at | 57.256 | 5.57E-07 | 6.254 | 0.00010893 | SLN |
| 80736_at | -56.884 | 5.72E-07 | 6.2427 | 0.00010893 | SLC44A4 |
| 116441_at | 56.657 | 5.81E-07 | 6.2358 | 0.00010893 | TM4SF18 |
| 1364_at | -55.781 | 6.18E-07 | 6.2087 | 0.00010893 | CLDN4 |
| 1326_at | -55.659 | 6.24E-07 | 6.2049 | 0.00010893 | MAP3K8 |
| 652995_at | -54.431 | 6.82E-07 | 6.1662 | 0.00010893 | UCA1 |
| 1368_at | 54.415 | 6.83E-07 | 6.1657 | 0.00010893 | CPM |
| 1577_at | 53.757 | 7.17E-07 | 6.1446 | 0.00010893 | CYP3A5 |
| 84868_at | 53.594 | 7.26E-07 | 6.1393 | 0.00010893 | HAVCR2 |
| 4880_at | 53.293 | 7.42E-07 | 6.1295 | 0.00010893 | NPPC |
| 3484_at | 53.252 | 7.44E-07 | 6.1282 | 0.00010893 | IGFBP1 |
| 5653_at | -53.173 | 7.49E-07 | 6.1256 | 0.00010893 | KLK6 |
| 5724_at | -52.861 | 7.67E-07 | 6.1154 | 0.00010893 | PTAFR |
| 54600_at | 52.847 | 7.67E-07 | 6.115 | 0.00010893 | UGT1A9 |
| 266977_at | -52.557 | 7.84E-07 | 6.1054 | 0.00010893 | GPR110 |
| 9414_at | -51.864 | 8.27E-07 | 6.0824 | 0.00010995 | TJP2 |
| 1604_at | -51.731 | 8.36E-07 | 6.0779 | 0.00010995 | CD55 |
| 199974_at | 51.215 | 8.70E-07 | 6.0605 | 0.00011051 | CYP4Z1 |
| 10610_at | -51.005 | 8.84E-07 | 6.0534 | 0.00011051 | ST6GALNAC2 |
| 9245_at | -48.388 | 1.09E-06 | 5.9621 | 0.00012253 | GCNT3 |
| 2312_at | 47.927 | 1.13E-06 | 5.9454 | 0.00012253 | FLG |
| 2239_at | 47.911 | 1.14E-06 | 5.9448 | 0.00012253 | GPC4 |
| 4747_at | 47.901 | 1.14E-06 | 5.9445 | 0.00012253 | NEFL |
| 200879_at | -47.645 | 1.16E-06 | 5.9352 | 0.00012253 | LIPH |
| 51474_at | -47.627 | 1.16E-06 | 5.9345 | 0.00012253 | LIMA1 |
| 306_at | -47.586 | 1.17E-06 | 5.933 | 0.00012253 | ANXA3 |
| 56999_at | -47.486 | 1.18E-06 | 5.9294 | 0.00012253 | ADAMTS9 |
| 3910_at | 46.843 | 1.24E-06 | 5.9057 | 0.00012624 | LAMA4 |
| 157506_at | 46.652 | 1.26E-06 | 5.8987 | 0.00012624 | RDH10 |
| 93273_at | -46.423 | 1.29E-06 | 5.8901 | 0.00012624 | LEMD1 |
| 240_at | -46.188 | 1.31E-06 | 5.8813 | 0.00012635 | ALOX5 |
| 3690_at | 45.634 | 1.38E-06 | 5.8604 | 0.00013008 | ITGB3 |
| 5174_at | 45.304 | 1.42E-06 | 5.8478 | 0.00013142 | PDZK1 |
| 5314_at | 44.95 | 1.46E-06 | 5.8342 | 0.00013315 | PKHD1 |
| 3949_at | -44.413 | 1.54E-06 | 5.8133 | 0.00013594 | LDLR |
| 26298_at | -44.151 | 1.57E-06 | 5.8031 | 0.00013594 | EHF |
| 10125_at | -44.054 | 1.59E-06 | 5.7993 | 0.00013594 | RASGRP1 |
| 4070_at | -43.938 | 1.60E-06 | 5.7947 | 0.00013594 | TACSTD2 |
| 4217_at | -43.632 | 1.65E-06 | 5.7826 | 0.00013744 | MAP3K5 |
| 57111_at | -42.711 | 1.80E-06 | 5.7456 | 0.00014624 | RAB25 |
| 3576_at | -42.451 | 1.84E-06 | 5.735 | 0.00014624 | CXCL8 |
| 4118_at | -42.438 | 1.84E-06 | 5.7345 | 0.00014624 | MAL |
| 6289_at | -41.932 | 1.93E-06 | 5.7137 | 0.00015102 | SAA2 |
| 50507_at | 41.394 | 2.04E-06 | 5.6913 | 0.00015457 | NOX4 |
| 64084_at | 41.369 | 2.04E-06 | 5.6902 | 0.00015457 | CLSTN2 |
| 91851_at | 41.079 | 2.10E-06 | 5.678 | 0.0001565 | CHRDL1 |
| 4216_at | 40.933 | 2.13E-06 | 5.6719 | 0.0001565 | MAP3K4 |
| 6750_at | 40.387 | 2.25E-06 | 5.6486 | 0.00016272 | SST |
| 53358_at | -39.929 | 2.35E-06 | 5.6288 | 0.00016699 | SHC3 |
| 23043_at | 39.823 | 2.38E-06 | 5.6242 | 0.00016699 | TNIK |
| 216_at | 39.606 | 2.43E-06 | 5.6147 | 0.00016699 | ALDH1A1 |
| 9365_at | 39.434 | 2.47E-06 | 5.6072 | 0.00016699 | KL |
| 2152_at | -39.429 | 2.47E-06 | 5.607 | 0.00016699 | F3 |
| 1004_at | 39.21 | 2.53E-06 | 5.5973 | 0.00016846 | CDH6 |
| 5631_at | 39.025 | 2.58E-06 | 5.5891 | 0.00016941 | PRPS1 |
| 338809_at | -38.795 | 2.64E-06 | 5.5789 | 0.00017121 | C12orf74 |
| 3620_at | -38.513 | 2.72E-06 | 5.5662 | 0.00017226 | IDO1 |
| 693199_at | -38.487 | 2.72E-06 | 5.5651 | 0.00017226 | MIR614 |
| 1601_at | 38.194 | 2.81E-06 | 5.5518 | 0.00017407 | DAB2 |
| 309_at | 38.147 | 2.82E-06 | 5.5497 | 0.00017407 | ANXA6 |
| 5924_at | 37.824 | 2.92E-06 | 5.535 | 0.0001753 | RASGRF2 |
| 55652_at | 37.646 | 2.97E-06 | 5.5268 | 0.0001753 | SLC48A1 |
| 8842_at | -37.487 | 3.02E-06 | 5.5194 | 0.0001753 | PROM1 |
| 124989_at | 37.464 | 3.03E-06 | 5.5184 | 0.0001753 | EFCAB13 |
| 56606_at | 37.463 | 3.03E-06 | 5.5183 | 0.0001753 | SLC2A9 |
| 221981_at | -37.404 | 3.05E-06 | 5.5156 | 0.0001753 | THSD7A |
| 80223_at | -37.272 | 3.09E-06 | 5.5095 | 0.00017556 | RAB11FIP1 |
| 7345_at | -37.178 | 3.13E-06 | 5.5051 | 0.00017556 | UCHL1 |
| 50940_at | 36.566 | 3.34E-06 | 5.4763 | 0.0001855 | PDE11A |
| 771_at | 36.164 | 3.49E-06 | 5.4572 | 0.00019172 | CA12 |
| 5649_at | 36.06 | 3.53E-06 | 5.4522 | 0.00019183 | RELN |
| 374_at | -35.918 | 3.59E-06 | 5.4453 | 0.00019203 | AREG |
| 26031_at | -35.857 | 3.61E-06 | 5.4424 | 0.00019203 | OSBPL3 |
| 94025_at | -35.495 | 3.76E-06 | 5.4248 | 0.00019785 | MUC16 |
| 3673_at | -35.382 | 3.81E-06 | 5.4193 | 0.0001983 | ITGA2 |
| 54845_at | -35.235 | 3.87E-06 | 5.4121 | 0.00019955 | ESRP1 |
| 27283_at | 34.967 | 3.99E-06 | 5.3989 | 0.00020361 | TINAG |
| 253461_at | 34.7 | 4.12E-06 | 5.3856 | 0.00020749 | ZBTB38 |
| 57205_at | 34.626 | 4.15E-06 | 5.3819 | 0.00020749 | ATP10D |
| 2018_at | 34.153 | 4.38E-06 | 5.358 | 0.00021456 | EMX2 |
| 969_at | -34.093 | 4.42E-06 | 5.355 | 0.00021456 | CD69 |
| 6586_at | 34.083 | 4.42E-06 | 5.3545 | 0.00021456 | SLIT3 |
| 84959_at | 33.796 | 4.57E-06 | 5.3398 | 0.00021784 | UBASH3B |
| 6568_at | 33.791 | 4.58E-06 | 5.3396 | 0.00021784 | SLC17A1 |
| 57631_at | -33.572 | 4.70E-06 | 5.3283 | 0.00022033 | LRCH2 |
| 23028_at | -33.426 | 4.78E-06 | 5.3208 | 0.00022033 | KDM1A |
| 3552_at | -33.385 | 4.80E-06 | 5.3186 | 0.00022033 | IL1A |
| 10866_at | -33.38 | 4.80E-06 | 5.3184 | 0.00022033 | HCP5 |
| 5069_at | -33.171 | 4.93E-06 | 5.3075 | 0.00022251 | PAPPA |
| 3664_at | -33.038 | 5.01E-06 | 5.3006 | 0.00022251 | IRF6 |
| 23328_at | -33.026 | 5.01E-06 | 5.2999 | 0.00022251 | SASH1 |
| 4354_at | 32.945 | 5.06E-06 | 5.2957 | 0.00022251 | MPP1 |
| 54102_at | -32.86 | 5.11E-06 | 5.2912 | 0.00022251 | CLIC6 |
| 10083_at | 32.853 | 5.12E-06 | 5.2908 | 0.00022251 | USH1C |
| 4316_at | -32.733 | 5.19E-06 | 5.2845 | 0.00022385 | MMP7 |
| 91748_at | 32.585 | 5.29E-06 | 5.2767 | 0.00022596 | ELMSAN1 |
| 5507_at | 32.51 | 5.34E-06 | 5.2727 | 0.00022612 | PPP1R3C |
| 51204_at | 32.402 | 5.41E-06 | 5.2669 | 0.00022723 | TACO1 |
| 23360_at | -32.192 | 5.55E-06 | 5.2556 | 0.00023033 | FNBP4 |
| 2034_at | 32.157 | 5.58E-06 | 5.2537 | 0.00023033 | EPAS1 |
| 115677_at | 31.78 | 5.84E-06 | 5.2333 | 0.00023785 | NOSTRIN |
| 123_at | 31.768 | 5.85E-06 | 5.2327 | 0.00023785 | PLIN2 |
| 51312_at | -31.649 | 5.94E-06 | 5.2262 | 0.0002395 | SLC25A37 |
| 11227_at | -31.541 | 6.02E-06 | 5.2203 | 0.00024082 | GALNT5 |
| 6582_at | 31.356 | 6.16E-06 | 5.2101 | 0.00024367 | SLC22A2 |
| 130367_at | -31.295 | 6.21E-06 | 5.2067 | 0.00024367 | SGPP2 |
| 2012_at | -31.262 | 6.24E-06 | 5.2049 | 0.00024367 | EMP1 |
| 25925_at | 31.149 | 6.33E-06 | 5.1986 | 0.00024531 | ZNF521 |
| 23348_at | -30.85 | 6.58E-06 | 5.1819 | 0.00025231 | DOCK9 |
| 79094_at | -30.81 | 6.61E-06 | 5.1797 | 0.00025231 | CHAC1 |
| 9508_at | 30.654 | 6.75E-06 | 5.1709 | 0.00025443 | ADAMTS3 |
| 389136_at | -30.629 | 6.77E-06 | 5.1695 | 0.00025443 | VGLL3 |
| 4301_at | -30.518 | 6.87E-06 | 5.1632 | 0.00025621 | MLLT4 |
| 2037_at | -30.327 | 7.04E-06 | 5.1523 | 0.00026076 | EPB41L2 |
| 401237_at | -30.201 | 7.16E-06 | 5.1451 | 0.00026317 | CASC15 |
| 4731_at | 30.101 | 7.26E-06 | 5.1393 | 0.00026387 | NDUFV3 |
| 7113_at | -30.071 | 7.28E-06 | 5.1376 | 0.00026387 | TMPRSS2 |
| 51365_at | 29.978 | 7.37E-06 | 5.1323 | 0.00026522 | PLA1A |
| 1545_at | 29.915 | 7.44E-06 | 5.1286 | 0.00026553 | CYP1B1 |
| 3488_at | -29.769 | 7.58E-06 | 5.1202 | 0.00026884 | IGFBP5 |
| 114569_at | -29.686 | 7.67E-06 | 5.1154 | 0.00026993 | MAL2 |
| 23089_at | 29.486 | 7.88E-06 | 5.1036 | 0.00027538 | PEG10 |
| 5650_at | -29.237 | 8.15E-06 | 5.089 | 0.00028111 | KLK7 |
| 6696_at | 29.142 | 8.25E-06 | 5.0834 | 0.00028111 | SPP1 |
| 5327_at | -29.121 | 8.28E-06 | 5.0821 | 0.00028111 | PLAT |
| 9353_at | -29.094 | 8.31E-06 | 5.0805 | 0.00028111 | SLIT2 |
| 1356_at | 29.082 | 8.32E-06 | 5.0797 | 0.00028111 | CP |
| 19_at | 28.895 | 8.54E-06 | 5.0686 | 0.00028355 | ABCA1 |
| 23475_at | 28.886 | 8.55E-06 | 5.068 | 0.00028355 | QPRT |
| 56649_at | -28.828 | 8.62E-06 | 5.0646 | 0.00028355 | TMPRSS4 |
| 857_at | 28.825 | 8.62E-06 | 5.0644 | 0.00028355 | CAV1 |
| 57153_at | -28.701 | 8.77E-06 | 5.0569 | 0.00028659 | SLC44A2 |
| 6563_at | 28.528 | 8.99E-06 | 5.0465 | 0.00029019 | SLC14A1 |
| 91351_at | -28.518 | 9.00E-06 | 5.0459 | 0.00029019 | DDX60L |
| 57722_at | -28.381 | 9.17E-06 | 5.0376 | 0.00029112 | IGDCC4 |
| 4072_at | -28.355 | 9.21E-06 | 5.0359 | 0.00029112 | EPCAM |
| 3219_at | -28.354 | 9.21E-06 | 5.0359 | 0.00029112 | HOXB9 |
| 283651_at | -28.265 | 9.32E-06 | 5.0305 | 0.00029112 | HMGN2P46 |
| 2650_at | 28.254 | 9.34E-06 | 5.0298 | 0.00029112 | GCNT1 |
| 81846_at | -28.224 | 9.38E-06 | 5.028 | 0.00029112 | SBF2 |
| 494470_at | -28.038 | 9.63E-06 | 5.0165 | 0.00029705 | RNF165 |
| 22861_at | -27.881 | 9.85E-06 | 5.0068 | 0.00030194 | NLRP1 |
| 340061_at | -27.817 | 9.94E-06 | 5.0028 | 0.00030286 | TMEM173 |
| 8671_at | 27.633 | 1.02E-05 | 4.9914 | 0.00030539 | SLC4A4 |
| 1001_at | -27.612 | 1.02E-05 | 4.99 | 0.00030539 | CDH3 |
| 347733_at | -27.57 | 1.03E-05 | 4.9874 | 0.00030539 | TUBB2B |
| 5789_at | 27.506 | 1.04E-05 | 4.9834 | 0.00030539 | PTPRD |
| 9971_at | 27.481 | 1.04E-05 | 4.9818 | 0.00030539 | NR1H4 |
| 79815_at | 27.434 | 1.05E-05 | 4.9789 | 0.00030539 | NIPAL2 |
| 10857_at | 27.413 | 1.05E-05 | 4.9775 | 0.00030539 | PGRMC1 |
| 64063_at | -27.408 | 1.05E-05 | 4.9772 | 0.00030539 | PRSS22 |
| 51561_at | -27.381 | 1.06E-05 | 4.9755 | 0.00030539 | IL23A |
| 9267_at | -27.349 | 1.06E-05 | 4.9735 | 0.00030539 | CYTH1 |
| 10867_at | 27.253 | 1.08E-05 | 4.9674 | 0.00030791 | TSPAN9 |
| 54625_at | -26.845 | 1.14E-05 | 4.9413 | 0.00032516 | PARP14 |
| 127294_at | 26.719 | 1.17E-05 | 4.9332 | 0.00032942 | MYOM3 |
| 1510_at | -26.576 | 1.19E-05 | 4.9239 | 0.00033466 | CTSE |
| 220_at | -26.477 | 1.21E-05 | 4.9174 | 0.00033533 | ALDH1A3 |
| 9891_at | -26.474 | 1.21E-05 | 4.9172 | 0.00033533 | NUAK1 |
| 26762_at | 26.451 | 1.21E-05 | 4.9157 | 0.00033533 | HAVCR1 |
| 6567_at | 26.408 | 1.22E-05 | 4.913 | 0.00033562 | SLC16A2 |
| 221806_at | -26.34 | 1.23E-05 | 4.9085 | 0.00033725 | VWDE |
| 6493_at | -26.25 | 1.25E-05 | 4.9025 | 0.00034004 | SIM2 |
| 342897_at | -25.908 | 1.32E-05 | 4.8799 | 0.00035629 | NCCRP1 |
| 5770_at | 25.844 | 1.33E-05 | 4.8756 | 0.00035788 | PTPN1 |
| 339535_at | 25.763 | 1.35E-05 | 4.8702 | 0.00035911 | LINC01139 |
| 57570_at | 25.753 | 1.35E-05 | 4.8695 | 0.00035911 | TRMT5 |
| 3426_at | 25.693 | 1.36E-05 | 4.8655 | 0.00036052 | CFI |
| 6307_at | -25.611 | 1.38E-05 | 4.86 | 0.00036321 | MSMO1 |
| 388630_at | 25.515 | 1.40E-05 | 4.8535 | 0.00036621 | TRABD2B |
| 6533_at | 25.491 | 1.41E-05 | 4.8518 | 0.00036621 | SLC6A6 |
| 6590_at | -25.396 | 1.43E-05 | 4.8454 | 0.00036978 | SLPI |
| 166647_at | -25.222 | 1.47E-05 | 4.8335 | 0.00037398 | GPR125 |
| 867_at | -25.212 | 1.47E-05 | 4.8328 | 0.00037398 | CBL |
| 2121_at | 25.197 | 1.47E-05 | 4.8318 | 0.00037398 | EVC |
| 1794_at | 25.194 | 1.47E-05 | 4.8316 | 0.00037398 | DOCK2 |
| 1848_at | 25.096 | 1.50E-05 | 4.8249 | 0.00037788 | DUSP6 |
| 2591_at | -25.01 | 1.52E-05 | 4.8189 | 0.0003807 | GALNT3 |
| 2878_at | 24.986 | 1.52E-05 | 4.8173 | 0.0003807 | GPX3 |
| 1106_at | -24.869 | 1.55E-05 | 4.8092 | 0.00038409 | CHD2 |
| 5732_at | 24.868 | 1.55E-05 | 4.8091 | 0.00038409 | PTGER2 |
| 4060_at | 24.709 | 1.59E-05 | 4.798 | 0.0003921 | LUM |
| 56670_at | 24.508 | 1.64E-05 | 4.7839 | 0.00040307 | SUCNR1 |
| 57451_at | 24.435 | 1.66E-05 | 4.7787 | 0.00040423 | TENM2 |
| 7837_at | -24.43 | 1.67E-05 | 4.7784 | 0.00040423 | PXDN |
| 387695_at | 24.395 | 1.68E-05 | 4.7759 | 0.00040456 | C10orf99 |
| 4319_at | -24.349 | 1.69E-05 | 4.7726 | 0.00040569 | MMP10 |
| 4139_at | -24.242 | 1.72E-05 | 4.765 | 0.00040961 | MARK1 |
| 83641_at | 24.232 | 1.72E-05 | 4.7643 | 0.00040961 | FAM107B |
| 9122_at | 24.069 | 1.77E-05 | 4.7526 | 0.00041876 | SLC16A4 |
| 3162_at | 24.033 | 1.78E-05 | 4.75 | 0.00041928 | HMOX1 |
| 55959_at | 23.911 | 1.81E-05 | 4.7413 | 0.00042584 | SULF2 |
| 5793_at | 23.721 | 1.87E-05 | 4.7275 | 0.00043559 | PTPRG |
| 3480_at | 23.669 | 1.89E-05 | 4.7237 | 0.00043559 | IGF1R |
| 729830_at | -23.663 | 1.89E-05 | 4.7233 | 0.00043559 | FAM160A1 |
| 2731_at | 23.629 | 1.90E-05 | 4.7208 | 0.00043559 | GLDC |
| 80380_at | -23.621 | 1.90E-05 | 4.7202 | 0.00043559 | PDCD1LG2 |
| 10475_at | 23.609 | 1.91E-05 | 4.7194 | 0.00043559 | TRIM38 |
| 7498_at | -23.558 | 1.92E-05 | 4.7156 | 0.00043735 | XDH |
| 255488_at | -23.459 | 1.96E-05 | 4.7083 | 0.00044275 | RNF144B |
| 10184_at | 23.344 | 2.00E-05 | 4.6999 | 0.00044875 | LHFPL2 |
| 6288_at | -23.327 | 2.00E-05 | 4.6986 | 0.00044875 | SAA1 |
| 23555_at | -23.187 | 2.05E-05 | 4.6882 | 0.00045234 | TSPAN15 |
| 143903_at | -23.186 | 2.05E-05 | 4.6881 | 0.00045234 | LAYN |
| 2069_at | -23.166 | 2.06E-05 | 4.6866 | 0.00045234 | EREG |
| 388335_at | 23.147 | 2.06E-05 | 4.6852 | 0.00045234 | TMEM220 |
| 11213_at | -23.103 | 2.08E-05 | 4.6819 | 0.00045234 | IRAK3 |
| 80206_at | 23.098 | 2.08E-05 | 4.6816 | 0.00045234 | FHOD3 |
| 2983_at | 23.096 | 2.08E-05 | 4.6814 | 0.00045234 | GUCY1B3 |
| 4585_at | -23.075 | 2.09E-05 | 4.6798 | 0.00045234 | MUC4 |
| 8796_at | 22.882 | 2.16E-05 | 4.6653 | 0.00046564 | SCEL |
| 1287_at | -22.852 | 2.17E-05 | 4.6631 | 0.00046607 | COL4A5 |
| 92017_at | 22.798 | 2.19E-05 | 4.659 | 0.00046799 | SNX29 |
| 5874_at | -22.756 | 2.21E-05 | 4.6558 | 0.00046799 | RAB27B |
| 8406_at | -22.755 | 2.21E-05 | 4.6557 | 0.00046799 | SRPX |
| 3914_at | -22.72 | 2.22E-05 | 4.6531 | 0.00046886 | LAMB3 |
| 23303_at | -22.653 | 2.25E-05 | 4.6479 | 0.00047245 | KIF13B |
| 55601_at | -22.621 | 2.26E-05 | 4.6455 | 0.00047312 | DDX60 |
| 83658_at | 22.498 | 2.31E-05 | 4.6361 | 0.00048038 | DYNLRB1 |
| 255324_at | -22.487 | 2.32E-05 | 4.6353 | 0.00048038 | EPGN |
| 7980_at | 22.425 | 2.34E-05 | 4.6305 | 0.00048369 | TFPI2 |
| 114134_at | 22.386 | 2.36E-05 | 4.6275 | 0.00048505 | SLC2A13 |
| 7020_at | -22.339 | 2.38E-05 | 4.6238 | 0.00048589 | TFAP2A |
| 6299_at | 22.33 | 2.38E-05 | 4.6232 | 0.00048589 | SALL1 |
| 134285_at | -22.288 | 2.40E-05 | 4.62 | 0.00048657 | TMEM171 |
| 51133_at | 22.276 | 2.40E-05 | 4.619 | 0.00048657 | KCTD3 |
| 84419_at | -22.235 | 2.42E-05 | 4.6158 | 0.00048719 | C15orf48 |
| 64116_at | 22.209 | 2.43E-05 | 4.6138 | 0.00048719 | SLC39A8 |
| 26289_at | 22.202 | 2.44E-05 | 4.6132 | 0.00048719 | AK5 |
| 10551_at | -22.152 | 2.46E-05 | 4.6094 | 0.00048961 | AGR2 |
| 5305_at | 22.067 | 2.50E-05 | 4.6028 | 0.00049514 | PIP4K2A |
| 10205_at | -22.035 | 2.51E-05 | 4.6002 | 0.00049607 | MPZL2 |
| 3169_at | -22.006 | 2.52E-05 | 4.598 | 0.00049665 | FOXA1 |
| 3696_at | -21.877 | 2.58E-05 | 4.5878 | 0.00050583 | ITGB8 |
| 57089_at | 21.862 | 2.59E-05 | 4.5866 | 0.00050583 | ENTPD7 |
| 79888_at | 21.793 | 2.62E-05 | 4.5812 | 0.00051021 | LPCAT1 |
| 3934_at | -21.718 | 2.66E-05 | 4.5753 | 0.00051524 | LCN2 |
| 9829_at | 21.662 | 2.69E-05 | 4.5708 | 0.00051534 | DNAJC6 |
| 85477_at | -21.644 | 2.70E-05 | 4.5694 | 0.00051534 | SCIN |
| 847_at | 21.631 | 2.70E-05 | 4.5683 | 0.00051534 | CAT |
| 4313_at | -21.627 | 2.70E-05 | 4.568 | 0.00051534 | MMP2 |
| 29943_at | -21.612 | 2.71E-05 | 4.5668 | 0.00051534 | PADI1 |
| 5610_at | -21.57 | 2.73E-05 | 4.5634 | 0.00051741 | EIF2AK2 |
| 6337_at | -21.503 | 2.77E-05 | 4.5581 | 0.00052016 | SCNN1A |
| 102724124_at | -21.5 | 2.77E-05 | 4.5579 | 0.00052016 | LOC102724124 |
| 55729_at | -21.478 | 2.78E-05 | 4.5561 | 0.00052038 | ATF7IP |
| 89795_at | -21.355 | 2.84E-05 | 4.5461 | 0.0005304 | NAV3 |
| 8542_at | -21.298 | 2.87E-05 | 4.5416 | 0.00053289 | APOL1 |
| 92292_at | 21.29 | 2.88E-05 | 4.5409 | 0.00053289 | GLYATL1 |
| 54541_at | 21.265 | 2.89E-05 | 4.5389 | 0.00053334 | DDIT4 |
| 101927960_at | 21.193 | 2.93E-05 | 4.533 | 0.00053736 | LOC101927960 |
| 1992_at | -21.169 | 2.94E-05 | 4.5311 | 0.00053736 | SERPINB1 |
| 4642_at | -21.147 | 2.96E-05 | 4.5293 | 0.00053736 | MYO1D |
| 84675_at | -21.147 | 2.96E-05 | 4.5293 | 0.00053736 | TRIM55 |
| 64761_at | -21.069 | 3.00E-05 | 4.5229 | 0.00054334 | PARP12 |
| 54852_at | 20.977 | 3.05E-05 | 4.5154 | 0.00054649 | PAQR5 |
| 29015_at | -20.97 | 3.06E-05 | 4.5148 | 0.00054649 | SLC43A3 |
| 4810_at | 20.965 | 3.06E-05 | 4.5144 | 0.00054649 | NHS |
| 55304_at | 20.962 | 3.06E-05 | 4.5141 | 0.00054649 | SPTLC3 |
| 4582_at | -20.837 | 3.13E-05 | 4.5038 | 0.00055644 | MUC1 |
| 10186_at | 20.83 | 3.14E-05 | 4.5032 | 0.00055644 | LHFP |
| 23767_at | 20.807 | 3.15E-05 | 4.5013 | 0.00055689 | FLRT3 |
| 1009_at | -20.758 | 3.18E-05 | 4.4973 | 0.00056013 | CDH11 |
| 2791_at | 20.739 | 3.19E-05 | 4.4957 | 0.00056016 | GNG11 |
| 114757_at | -20.634 | 3.26E-05 | 4.4869 | 0.00056962 | CYGB |
| 642934_at | 20.603 | 3.28E-05 | 4.4843 | 0.00057105 | LOC642934 |
| 60681_at | -20.542 | 3.32E-05 | 4.4792 | 0.00057404 | FKBP10 |
| 8447_at | 20.54 | 3.32E-05 | 4.479 | 0.00057404 | DOC2B |
| 10165_at | 20.499 | 3.35E-05 | 4.4756 | 0.00057662 | SLC25A13 |
| 4792_at | -20.476 | 3.36E-05 | 4.4737 | 0.00057684 | NFKBIA |
| 116372_at | 20.461 | 3.37E-05 | 4.4724 | 0.00057684 | LYPD1 |
| 3772_at | 20.383 | 3.42E-05 | 4.4658 | 0.00058367 | KCNJ15 |
| 10809_at | -20.34 | 3.45E-05 | 4.4622 | 0.00058656 | STARD10 |
| 2697_at | -20.267 | 3.50E-05 | 4.4561 | 0.00059294 | GJA1 |
| 3773_at | 20.209 | 3.54E-05 | 4.4511 | 0.00059776 | KCNJ16 |
| 51299_at | 20.154 | 3.58E-05 | 4.4464 | 0.00060223 | NRN1 |
| 692075_at | -19.985 | 3.70E-05 | 4.4319 | 0.00062009 | SNORD6 |
| 6564_at | -19.955 | 3.72E-05 | 4.4293 | 0.00062009 | SLC15A1 |
| 23072_at | 19.933 | 3.74E-05 | 4.4274 | 0.00062009 | HECW1 |
| 10891_at | 19.896 | 3.77E-05 | 4.4242 | 0.00062009 | PPARGC1A |
| 10561_at | -19.878 | 3.78E-05 | 4.4227 | 0.00062009 | IFI44 |
| 54498_at | 19.872 | 3.78E-05 | 4.4221 | 0.00062009 | SMOX |
| 987_at | -19.853 | 3.80E-05 | 4.4205 | 0.00062009 | LRBA |
| 8728_at | -19.841 | 3.81E-05 | 4.4194 | 0.00062009 | ADAM19 |
| 57154_at | -19.84 | 3.81E-05 | 4.4194 | 0.00062009 | SMURF1 |
| 90139_at | 19.839 | 3.81E-05 | 4.4193 | 0.00062009 | TSPAN18 |
| 5270_at | 19.778 | 3.86E-05 | 4.414 | 0.00062569 | SERPINE2 |
| 1301_at | -19.745 | 3.88E-05 | 4.411 | 0.00062788 | COL11A1 |
| 112399_at | 19.712 | 3.91E-05 | 4.4082 | 0.00062847 | EGLN3 |
| 11167_at | -19.7 | 3.92E-05 | 4.4072 | 0.00062847 | FSTL1 |
| 4067_at | -19.692 | 3.92E-05 | 4.4064 | 0.00062847 | LYN |
| 140469_at | -19.66 | 3.95E-05 | 4.4036 | 0.00062899 | MYO3B |
| 9052_at | -19.656 | 3.95E-05 | 4.4033 | 0.00062899 | GPRC5A |
| 27289_at | -19.611 | 3.99E-05 | 4.3994 | 0.00062998 | RND1 |
| 346389_at | -19.606 | 3.99E-05 | 4.3989 | 0.00062998 | MACC1 |
| 664701_at | 19.587 | 4.01E-05 | 4.3972 | 0.00062998 | ZNF826P |
| 8714_at | 19.561 | 4.03E-05 | 4.3949 | 0.00062998 | ABCC3 |
| 23743_at | 19.561 | 4.03E-05 | 4.3949 | 0.00062998 | BHMT2 |
| 6578_at | 19.555 | 4.03E-05 | 4.3944 | 0.00062998 | SLCO2A1 |
| 22881_at | 19.517 | 4.06E-05 | 4.3911 | 0.00063285 | ANKRD6 |
| 6480_at | -19.488 | 4.09E-05 | 4.3885 | 0.00063465 | ST6GAL1 |
| 118788_at | -19.47 | 4.10E-05 | 4.3869 | 0.00063503 | PIK3AP1 |
| 51129_at | 19.379 | 4.18E-05 | 4.3788 | 0.0006449 | ANGPTL4 |
| 3936_at | -19.361 | 4.20E-05 | 4.3772 | 0.00064529 | LCP1 |
| 3955_at | 19.304 | 4.25E-05 | 4.3721 | 0.00064878 | LFNG |
| 7965_at | 19.301 | 4.25E-05 | 4.3719 | 0.00064878 | AIMP2 |
| 259217_at | 19.282 | 4.26E-05 | 4.3702 | 0.00064878 | HSPA12A |
| 57685_at | 19.275 | 4.27E-05 | 4.3696 | 0.00064878 | CACHD1 |
| 9770_at | -19.259 | 4.28E-05 | 4.3681 | 0.0006489 | RASSF2 |
| 2982_at | 19.245 | 4.30E-05 | 4.3669 | 0.0006489 | GUCY1A3 |
| 23136_at | 19.119 | 4.41E-05 | 4.3556 | 0.0006594 | EPB41L3 |
| 25890_at | -19.109 | 4.42E-05 | 4.3547 | 0.0006594 | ABI3BP |
| 387647_at | 19.089 | 4.44E-05 | 4.3529 | 0.0006594 | PTCHD3P1 |
| 5359_at | -19.087 | 4.44E-05 | 4.3527 | 0.0006594 | PLSCR1 |
| 54020_at | -19.079 | 4.45E-05 | 4.352 | 0.0006594 | SLC37A1 |
| 26509_at | -19.034 | 4.49E-05 | 4.3479 | 0.0006594 | MYOF |
| 23508_at | -19.033 | 4.49E-05 | 4.3478 | 0.0006594 | TTC9 |
| 54739_at | -19.03 | 4.49E-05 | 4.3476 | 0.0006594 | XAF1 |
| 55930_at | -19.025 | 4.50E-05 | 4.3471 | 0.0006594 | MYO5C |
| 5168_at | 19.024 | 4.50E-05 | 4.347 | 0.0006594 | ENPP2 |
| 51752_at | -18.989 | 4.53E-05 | 4.3438 | 0.00066207 | ERAP1 |
| 84916_at | 18.976 | 4.54E-05 | 4.3427 | 0.00066207 | CIRH1A |
| 26227_at | -18.919 | 4.60E-05 | 4.3375 | 0.00066781 | PHGDH |
| 55286_at | -18.907 | 4.61E-05 | 4.3364 | 0.00066781 | C4orf19 |
| 8777_at | -18.856 | 4.66E-05 | 4.3317 | 0.00067231 | MPDZ |
| 64776_at | 18.848 | 4.67E-05 | 4.331 | 0.00067231 | C11orf1 |
| 7188_at | -18.821 | 4.69E-05 | 4.3285 | 0.00067417 | TRAF5 |
| 55002_at | 18.724 | 4.79E-05 | 4.3196 | 0.00068615 | TMCO3 |
| 8828_at | -18.71 | 4.80E-05 | 4.3184 | 0.00068616 | NRP2 |
| 5635_at | -18.668 | 4.85E-05 | 4.3145 | 0.00069003 | PRPSAP1 |
| 4129_at | 18.654 | 4.86E-05 | 4.3132 | 0.00069003 | MAOB |
| 10964_at | -18.643 | 4.87E-05 | 4.3122 | 0.00069003 | IFI44L |
| 4638_at | 18.533 | 4.99E-05 | 4.302 | 0.00070448 | MYLK |
| 4651_at | -18.439 | 5.09E-05 | 4.2932 | 0.00071684 | MYO10 |
| 6303_at | -18.411 | 5.12E-05 | 4.2906 | 0.00071911 | SAT1 |
| 84162_at | -18.38 | 5.15E-05 | 4.2878 | 0.00072184 | KIAA1109 |
| 100287934_at | -18.318 | 5.22E-05 | 4.2819 | 0.00072899 | LOC100287934 |
| 9583_at | -18.309 | 5.24E-05 | 4.2811 | 0.00072899 | ENTPD4 |
| 64762_at | 18.29 | 5.26E-05 | 4.2793 | 0.00072988 | GAREM |
| 4837_at | 18.273 | 5.28E-05 | 4.2778 | 0.0007305 | NNMT |
| 4599_at | -18.256 | 5.29E-05 | 4.2761 | 0.00073119 | MX1 |
| 3075_at | 18.228 | 5.33E-05 | 4.2735 | 0.00073202 | CFH |
| 147040_at | 18.226 | 5.33E-05 | 4.2733 | 0.00073202 | KCTD11 |
| 57514_at | 18.174 | 5.39E-05 | 4.2684 | 0.00073827 | ARHGAP31 |
| 5033_at | 18.137 | 5.43E-05 | 4.2649 | 0.0007406 | P4HA1 |
| 4122_at | -18.135 | 5.44E-05 | 4.2646 | 0.0007406 | MAN2A2 |
| 10418_at | -18.044 | 5.55E-05 | 4.2561 | 0.00075053 | SPON1 |
| 6697_at | 18.043 | 5.55E-05 | 4.2559 | 0.00075053 | SPR |
| 2260_at | 18.033 | 5.56E-05 | 4.2549 | 0.00075053 | FGFR1 |
| 5318_at | -18.024 | 5.57E-05 | 4.2541 | 0.00075053 | PKP2 |
| 3397_at | 17.987 | 5.62E-05 | 4.2505 | 0.00075473 | ID1 |
| 5652_at | -17.949 | 5.66E-05 | 4.247 | 0.00075837 | PRSS8 |
| 1606_at | -17.887 | 5.74E-05 | 4.241 | 0.00075837 | DGKA |
| 1836_at | 17.885 | 5.74E-05 | 4.2408 | 0.00075837 | SLC26A2 |
| 7412_at | 17.851 | 5.79E-05 | 4.2376 | 0.00075837 | VCAM1 |
| 4891_at | -17.85 | 5.79E-05 | 4.2374 | 0.00075837 | SLC11A2 |
| 4306_at | -17.848 | 5.79E-05 | 4.2372 | 0.00075837 | NR3C2 |
| 55785_at | -17.843 | 5.80E-05 | 4.2367 | 0.00075837 | FGD6 |
| 9162_at | 17.836 | 5.81E-05 | 4.2361 | 0.00075837 | DGKI |
| 115290_at | 17.821 | 5.83E-05 | 4.2346 | 0.00075837 | FBXO17 |
| 23670_at | 17.802 | 5.85E-05 | 4.2328 | 0.00075837 | TMEM2 |
| 6319_at | -17.801 | 5.85E-05 | 4.2327 | 0.00075837 | SCD |
| 3459_at | -17.792 | 5.86E-05 | 4.2319 | 0.00075837 | IFNGR1 |
| 1824_at | -17.785 | 5.87E-05 | 4.2311 | 0.00075837 | DSC2 |
| 8508_at | -17.784 | 5.87E-05 | 4.2311 | 0.00075837 | NIPSNAP1 |
| 117177_at | -17.783 | 5.88E-05 | 4.231 | 0.00075837 | RAB3IP |
| 10623_at | 17.775 | 5.89E-05 | 4.2302 | 0.00075837 | POLR3C |
| 9793_at | -17.741 | 5.93E-05 | 4.2269 | 0.0007622 | CKAP5 |
| 693139_at | -17.683 | 6.01E-05 | 4.2213 | 0.00077008 | MIR554 |
| 83716_at | 17.664 | 6.03E-05 | 4.2194 | 0.00077144 | CRISPLD2 |
| 8853_at | -17.64 | 6.07E-05 | 4.2171 | 0.0007715 | ASAP2 |
| 4683_at | 17.635 | 6.07E-05 | 4.2166 | 0.0007715 | NBN |
| 283987_at | -17.629 | 6.08E-05 | 4.216 | 0.0007715 | HID1 |
| 80262_at | 17.608 | 6.11E-05 | 4.214 | 0.0007732 | C16orf70 |
| 8821_at | -17.557 | 6.18E-05 | 4.209 | 0.00077764 | INPP4B |
| 80114_at | 17.545 | 6.20E-05 | 4.2078 | 0.00077764 | BICC1 |
| 552860_at | -17.535 | 6.21E-05 | 4.2068 | 0.00077764 | SAMD12-AS1 |
| 1666_at | 17.53 | 6.22E-05 | 4.2064 | 0.00077764 | DECR1 |
| 9955_at | -17.527 | 6.22E-05 | 4.206 | 0.00077764 | HS3ST3A1 |
| 341346_at | -17.492 | 6.27E-05 | 4.2026 | 0.0007818 | SMCO2 |
| 51274_at | -17.467 | 6.31E-05 | 4.2002 | 0.00078431 | KLF3 |
| 961_at | -17.426 | 6.37E-05 | 4.1961 | 0.00078888 | CD47 |
| 4897_at | 17.42 | 6.38E-05 | 4.1955 | 0.00078888 | NRCAM |
| 636_at | 17.393 | 6.41E-05 | 4.1929 | 0.00079054 | BICD1 |
| 4983_at | -17.389 | 6.42E-05 | 4.1924 | 0.00079054 | OPHN1 |
| 54577_at | 17.375 | 6.44E-05 | 4.191 | 0.00079115 | UGT1A7 |
| 629_at | -17.346 | 6.48E-05 | 4.1882 | 0.00079443 | CFB |
| 22902_at | 17.317 | 6.53E-05 | 4.1853 | 0.00079687 | RUFY3 |
| 11174_at | -17.311 | 6.54E-05 | 4.1847 | 0.00079687 | ADAMTS6 |
| 9056_at | 17.271 | 6.60E-05 | 4.1807 | 0.00080051 | SLC7A7 |
| 55793_at | 17.27 | 6.60E-05 | 4.1806 | 0.00080051 | FAM63A |
| 3756_at | -17.254 | 6.62E-05 | 4.179 | 0.00080147 | KCNH1 |
| 128710_at | 17.238 | 6.65E-05 | 4.1775 | 0.00080162 | SLX4IP |
| 120892_at | 17.232 | 6.65E-05 | 4.1769 | 0.00080162 | LRRK2 |
| 3416_at | -17.215 | 6.68E-05 | 4.1752 | 0.00080198 | IDE |
| 6768_at | -17.209 | 6.69E-05 | 4.1746 | 0.00080198 | ST14 |
| 647024_at | -17.183 | 6.73E-05 | 4.172 | 0.00080483 | C6orf132 |
| 11254_at | -17.156 | 6.77E-05 | 4.1693 | 0.00080606 | SLC6A14 |
| 7114_at | -17.144 | 6.79E-05 | 4.1681 | 0.00080606 | TMSB4X |
| 1591_at | 17.136 | 6.80E-05 | 4.1673 | 0.00080606 | CYP24A1 |
| 6376_at | -17.126 | 6.82E-05 | 4.1663 | 0.00080606 | CX3CL1 |
| 374443_at | -17.125 | 6.82E-05 | 4.1662 | 0.00080606 | LOC374443 |
| 79589_at | 17.104 | 6.85E-05 | 4.164 | 0.00080815 | RNF128 |
| 57523_at | -17.054 | 6.93E-05 | 4.1591 | 0.00081327 | NYNRIN |
| 50810_at | -17.048 | 6.94E-05 | 4.1585 | 0.00081327 | HDGFRP3 |
| 2683_at | 17.046 | 6.95E-05 | 4.1582 | 0.00081327 | B4GALT1 |
| 3557_at | -17.014 | 7.00E-05 | 4.155 | 0.00081695 | IL1RN |
| 55573_at | 17.004 | 7.02E-05 | 4.154 | 0.00081695 | CDV3 |
| 29126_at | -16.996 | 7.03E-05 | 4.1532 | 0.00081695 | CD274 |
| 550112_at | 16.95 | 7.10E-05 | 4.1485 | 0.00082274 | UBA6-AS1 |
| 143_at | -16.935 | 7.13E-05 | 4.147 | 0.00082274 | PARP4 |
| 4094_at | 16.935 | 7.13E-05 | 4.147 | 0.00082274 | MAF |
| 81792_at | -16.926 | 7.14E-05 | 4.1461 | 0.00082274 | ADAMTS12 |
| 101928100_at | -16.898 | 7.19E-05 | 4.1433 | 0.00082629 | LOC101928100 |
| 51200_at | 16.82 | 7.32E-05 | 4.1353 | 0.00083958 | CPA4 |
| 1012_at | -16.798 | 7.36E-05 | 4.1331 | 0.0008399 | CDH13 |
| 10642_at | 16.795 | 7.37E-05 | 4.1328 | 0.0008399 | IGF2BP1 |
| 162461_at | 16.772 | 7.41E-05 | 4.1304 | 0.0008399 | TMEM92 |
| 7739_at | -16.77 | 7.41E-05 | 4.1302 | 0.0008399 | ZNF185 |
| 57707_at | 16.77 | 7.41E-05 | 4.1302 | 0.0008399 | TLDC1 |
| 33_at | 16.739 | 7.46E-05 | 4.127 | 0.00084344 | ACADL |
| 56477_at | -16.733 | 7.47E-05 | 4.1264 | 0.00084344 | CCL28 |
| 3553_at | -16.686 | 7.56E-05 | 4.1216 | 0.00085101 | IL1B |
| 29958_at | 16.638 | 7.65E-05 | 4.1166 | 0.00085879 | DMGDH |
| 9900_at | -16.629 | 7.66E-05 | 4.1157 | 0.00085879 | SV2A |
| 10053_at | -16.611 | 7.69E-05 | 4.1138 | 0.00086053 | AP1M2 |
| 55195_at | 16.598 | 7.72E-05 | 4.1125 | 0.00086124 | C14orf105 |
| 5872_at | 16.551 | 7.80E-05 | 4.1077 | 0.00086623 | RAB13 |
| 170384_at | 16.55 | 7.81E-05 | 4.1076 | 0.00086623 | FUT11 |
| 9805_at | -16.537 | 7.83E-05 | 4.1062 | 0.00086623 | SCRN1 |
| 271_at | 16.521 | 7.86E-05 | 4.1046 | 0.00086623 | AMPD2 |
| 818_at | -16.513 | 7.88E-05 | 4.1037 | 0.00086623 | CAMK2G |
| 101927873_at | -16.509 | 7.88E-05 | 4.1033 | 0.00086623 | LINC01508 |
| 26585_at | -16.505 | 7.89E-05 | 4.1029 | 0.00086623 | GREM1 |
| 57674_at | -16.496 | 7.91E-05 | 4.1019 | 0.00086623 | RNF213 |
| 7764_at | -16.49 | 7.92E-05 | 4.1013 | 0.00086623 | ZNF217 |
| 151176_at | 16.47 | 7.96E-05 | 4.0993 | 0.00086837 | FAM132B |
| 400221_at | 16.453 | 7.99E-05 | 4.0975 | 0.00086837 | FLJ22447 |
| 84141_at | 16.439 | 8.02E-05 | 4.096 | 0.00086837 | EVA1A |
| 22807_at | -16.424 | 8.05E-05 | 4.0944 | 0.00086837 | IKZF2 |
| 2936_at | 16.418 | 8.06E-05 | 4.0938 | 0.00086837 | GSR |
| 4311_at | -16.417 | 8.06E-05 | 4.0937 | 0.00086837 | MME |
| 5021_at | 16.412 | 8.07E-05 | 4.0931 | 0.00086837 | OXTR |
| 2114_at | -16.398 | 8.10E-05 | 4.0917 | 0.00086837 | ETS2 |
| 6398_at | -16.397 | 8.10E-05 | 4.0916 | 0.00086837 | SECTM1 |
| 92856_at | 16.39 | 8.11E-05 | 4.0909 | 0.00086837 | IMP4 |
| 23092_at | -16.35 | 8.19E-05 | 4.0867 | 0.00087362 | ARHGAP26 |
| 10257_at | 16.344 | 8.20E-05 | 4.086 | 0.00087362 | ABCC4 |
| 8900_at | -16.336 | 8.22E-05 | 4.0852 | 0.00087362 | CCNA1 |
| 146057_at | -16.328 | 8.24E-05 | 4.0843 | 0.00087362 | TTBK2 |
| 6695_at | 16.313 | 8.26E-05 | 4.0828 | 0.00087362 | SPOCK1 |
| 25937_at | 16.312 | 8.27E-05 | 4.0827 | 0.00087362 | WWTR1 |
| 4299_at | 16.303 | 8.28E-05 | 4.0818 | 0.00087363 | AFF1 |
| 4212_at | 16.275 | 8.34E-05 | 4.0788 | 0.00087782 | MEIS2 |
| 55556_at | 16.231 | 8.43E-05 | 4.0742 | 0.00088532 | ENOSF1 |
| 26278_at | -16.176 | 8.54E-05 | 4.0683 | 0.00089362 | SACS |
| 3214_at | -16.176 | 8.54E-05 | 4.0683 | 0.00089362 | HOXB4 |
| 664_at | 16.152 | 8.59E-05 | 4.0658 | 0.00089388 | BNIP3 |
| 79840_at | 16.144 | 8.61E-05 | 4.0649 | 0.00089388 | NHEJ1 |
| 1462_at | 16.143 | 8.61E-05 | 4.0648 | 0.00089388 | VCAN |
| 53346_at | -16.141 | 8.62E-05 | 4.0646 | 0.00089388 | TM6SF1 |
| 5966_at | -16.132 | 8.64E-05 | 4.0637 | 0.00089389 | REL |
| 84898_at | 16.112 | 8.68E-05 | 4.0616 | 0.00089637 | PLXDC2 |
| 6492_at | -16.051 | 8.81E-05 | 4.055 | 0.00090804 | SIM1 |
| 144402_at | 16.017 | 8.88E-05 | 4.0514 | 0.00091375 | CPNE8 |
| 89781_at | -16.002 | 8.92E-05 | 4.0498 | 0.00091444 | HPS4 |
| 84125_at | 15.998 | 8.93E-05 | 4.0493 | 0.00091444 | LRRIQ1 |
| 55118_at | 15.952 | 9.03E-05 | 4.0444 | 0.0009229 | CRTAC1 |
| 2065_at | -15.923 | 9.09E-05 | 4.0413 | 0.00092728 | ERBB3 |
| 3912_at | -15.917 | 9.11E-05 | 4.0406 | 0.00092728 | LAMB1 |
| 642587_at | -15.899 | 9.15E-05 | 4.0387 | 0.00092951 | MIR205HG |
| 3213_at | -15.89 | 9.17E-05 | 4.0377 | 0.00092972 | HOXB3 |
| 115811_at | -15.88 | 9.19E-05 | 4.0366 | 0.00093007 | IQCD |
| 3855_at | -15.84 | 9.28E-05 | 4.0323 | 0.0009375 | KRT7 |
| 92211_at | 15.822 | 9.32E-05 | 4.0304 | 0.00093946 | CDHR1 |
| 8824_at | 15.815 | 9.34E-05 | 4.0296 | 0.00093946 | CES2 |
| 3660_at | -15.788 | 9.40E-05 | 4.0267 | 0.00094398 | IRF2 |
| 3383_at | -15.763 | 9.46E-05 | 4.024 | 0.00094799 | ICAM1 |
| 6781_at | 15.749 | 9.50E-05 | 4.0224 | 0.00094946 | STC1 |
| 8631_at | -15.724 | 9.56E-05 | 4.0197 | 0.00095347 | SKAP1 |
| 5502_at | 15.593 | 9.88E-05 | 4.0054 | 0.00098345 | PPP1R1A |
| 4008_at | -15.572 | 9.93E-05 | 4.0031 | 0.00098575 | LMO7 |
| 59277_at | -15.56 | 9.96E-05 | 4.0018 | 0.00098575 | NTN4 |
| 51090_at | -15.551 | 9.98E-05 | 4.0007 | 0.00098575 | PLLP |
| 6347_at | 15.547 | 9.99E-05 | 4.0003 | 0.00098575 | CCL2 |
| 5205_at | -15.536 | 0.0001002 | 3.9991 | 0.00098575 | ATP8B1 |
| 7424_at | -15.53 | 0.00010035 | 3.9985 | 0.00098575 | VEGFC |
| 47_at | 15.526 | 0.00010046 | 3.998 | 0.00098575 | ACLY |
| 55625_at | 15.522 | 0.00010057 | 3.9975 | 0.00098575 | ZDHHC7 |
| 51084_at | 15.503 | 0.00010105 | 3.9955 | 0.00098858 | CRYL1 |
| 23428_at | 15.467 | 0.00010198 | 3.9915 | 0.00099229 | SLC7A8 |
| 9873_at | -15.464 | 0.00010205 | 3.9912 | 0.00099229 | FCHSD2 |
| 6678_at | 15.459 | 0.00010218 | 3.9906 | 0.00099229 | SPARC |
| 51442_at | -15.453 | 0.00010234 | 3.9899 | 0.00099229 | VGLL1 |
| 4952_at | -15.45 | 0.00010242 | 3.9896 | 0.00099229 | OCRL |
| 8100_at | 15.44 | 0.00010268 | 3.9885 | 0.00099281 | IFT88 |
| 9368_at | 15.417 | 0.0001033 | 3.9859 | 0.00099694 | SLC9A3R1 |
| 5163_at | 15.373 | 0.00010447 | 3.981 | 0.0010054 | PDK1 |
| 51110_at | 15.366 | 0.00010464 | 3.9803 | 0.0010054 | LACTB2 |
| 7490_at | 15.361 | 0.00010478 | 3.9797 | 0.0010054 | WT1 |
| 5265_at | 15.352 | 0.00010503 | 3.9787 | 0.0010058 | SERPINA1 |
| 3778_at | 15.329 | 0.00010565 | 3.9761 | 0.0010089 | KCNMA1 |
| 3400_at | 15.325 | 0.00010575 | 3.9757 | 0.0010089 | ID4 |
| 7356_at | -15.311 | 0.00010613 | 3.9742 | 0.001009 | SCGB1A1 |
| 51465_at | -15.31 | 0.00010617 | 3.974 | 0.001009 | UBE2J1 |
| 79600_at | 15.285 | 0.00010685 | 3.9712 | 0.0010126 | TCTN1 |
| 7402_at | -15.275 | 0.00010713 | 3.9701 | 0.0010126 | UTRN |
| 3207_at | -15.274 | 0.00010716 | 3.97 | 0.0010126 | HOXA11 |
| 54431_at | -15.26 | 0.00010755 | 3.9684 | 0.0010144 | DNAJC10 |
| 7405_at | -15.252 | 0.00010778 | 3.9675 | 0.0010147 | UVRAG |
| 4835_at | 15.202 | 0.00010917 | 3.9619 | 0.0010258 | NQO2 |
| 27286_at | 15.165 | 0.00011022 | 3.9577 | 0.001031 | SRPX2 |
| 9131_at | 15.163 | 0.0001103 | 3.9574 | 0.001031 | AIFM1 |
| 9982_at | -15.161 | 0.00011034 | 3.9573 | 0.001031 | FGFBP1 |
| 2568_at | -15.141 | 0.00011092 | 3.955 | 0.0010323 | GABRP |
| 10367_at | 15.141 | 0.00011093 | 3.955 | 0.0010323 | MICU1 |
| 56938_at | -15.135 | 0.00011109 | 3.9543 | 0.0010323 | ARNTL2 |
| 316_at | 15.105 | 0.00011198 | 3.9509 | 0.0010345 | AOX1 |
| 54502_at | 15.102 | 0.00011205 | 3.9506 | 0.0010345 | RBM47 |
| 9201_at | 15.101 | 0.00011207 | 3.9505 | 0.0010345 | DCLK1 |
| 23481_at | 15.098 | 0.00011217 | 3.9501 | 0.0010345 | PES1 |
| 16_at | -15.076 | 0.00011283 | 3.9476 | 0.0010387 | AARS |
| 6549_at | -15.031 | 0.00011416 | 3.9425 | 0.001049 | SLC9A2 |
| 358_at | 15.024 | 0.00011436 | 3.9417 | 0.001049 | AQP1 |
| 4318_at | -15.006 | 0.0001149 | 3.9397 | 0.001052 | MMP9 |
| 23175_at | -14.996 | 0.00011521 | 3.9385 | 0.0010529 | LPIN1 |
| 481_at | -14.988 | 0.00011545 | 3.9376 | 0.0010531 | ATP1B1 |
| 283431_at | 14.944 | 0.00011679 | 3.9326 | 0.0010582 | GAS2L3 |
| 100131897_at | 14.934 | 0.00011712 | 3.9314 | 0.0010582 | FAM196B |
| 7482_at | 14.933 | 0.00011713 | 3.9313 | 0.0010582 | WNT2B |
| 125113_at | 14.931 | 0.00011718 | 3.9311 | 0.0010582 | KRT222 |
| 1278_at | 14.928 | 0.00011728 | 3.9308 | 0.0010582 | COL1A2 |
| 247_at | -14.926 | 0.00011736 | 3.9305 | 0.0010582 | ALOX15B |
| 54749_at | 14.922 | 0.00011749 | 3.93 | 0.0010582 | EPDR1 |
| 6558_at | -14.902 | 0.0001181 | 3.9278 | 0.0010605 | SLC12A2 |
| 147798_at | -14.894 | 0.00011836 | 3.9268 | 0.0010605 | TMC4 |
| 6474_at | 14.888 | 0.00011854 | 3.9261 | 0.0010605 | SHOX2 |
| 8605_at | -14.884 | 0.00011866 | 3.9257 | 0.0010605 | PLA2G4C |
| 79711_at | 14.87 | 0.00011911 | 3.9241 | 0.0010605 | IPO4 |
| 3157_at | -14.868 | 0.00011915 | 3.9239 | 0.0010605 | HMGCS1 |
| 2045_at | 14.866 | 0.00011923 | 3.9236 | 0.0010605 | EPHA7 |
| 10482_at | 14.858 | 0.00011948 | 3.9227 | 0.0010609 | NXF1 |
| 2530_at | -14.809 | 0.00012104 | 3.9171 | 0.0010728 | FUT8 |
| 79633_at | 14.786 | 0.0001218 | 3.9144 | 0.0010776 | FAT4 |
| 84952_at | 14.76 | 0.00012265 | 3.9113 | 0.0010832 | CGNL1 |
| 55608_at | -14.743 | 0.00012319 | 3.9094 | 0.0010861 | ANKRD10 |
| 55225_at | -14.68 | 0.00012529 | 3.9021 | 0.0011027 | RAVER2 |
| 196047_at | 14.648 | 0.00012638 | 3.8983 | 0.0011083 | EMX2OS |
| 8837_at | -14.647 | 0.00012641 | 3.8982 | 0.0011083 | CFLAR |
| 1962_at | 14.641 | 0.00012662 | 3.8975 | 0.0011083 | EHHADH |
| 58191_at | -14.629 | 0.00012702 | 3.8961 | 0.0011083 | CXCL16 |
| 84817_at | 14.629 | 0.00012704 | 3.8961 | 0.0011083 | TXNDC17 |
| 57568_at | 14.617 | 0.00012743 | 3.8947 | 0.0011098 | SIPA1L2 |
| 7099_at | 14.602 | 0.00012794 | 3.893 | 0.0011123 | TLR4 |
| 411_at | 14.573 | 0.00012897 | 3.8895 | 0.0011159 | ARSB |
| 3572_at | 14.571 | 0.00012902 | 3.8894 | 0.0011159 | IL6ST |
| 1690_at | -14.571 | 0.00012903 | 3.8893 | 0.0011159 | COCH |
| 1281_at | 14.528 | 0.00013052 | 3.8843 | 0.001126 | COL3A1 |
| 22873_at | 14.525 | 0.00013065 | 3.8839 | 0.001126 | DZIP1 |
| 80031_at | 14.507 | 0.00013128 | 3.8818 | 0.0011296 | SEMA6D |
| 374378_at | 14.495 | 0.00013169 | 3.8804 | 0.0011312 | GALNT18 |
| 5255_at | -14.485 | 0.00013207 | 3.8792 | 0.0011325 | PHKA1 |
| 54858_at | -14.476 | 0.00013238 | 3.8782 | 0.0011332 | PGPEP1 |
| 10370_at | 14.46 | 0.00013297 | 3.8762 | 0.0011353 | CITED2 |
| 4741_at | 14.45 | 0.00013334 | 3.8751 | 0.0011353 | NEFM |
| 11221_at | -14.449 | 0.00013338 | 3.8749 | 0.0011353 | DUSP10 |
| 79154_at | 14.444 | 0.00013354 | 3.8744 | 0.0011353 | DHRS11 |
| 7832_at | -14.43 | 0.00013405 | 3.8727 | 0.0011377 | BTG2 |
| 6318_at | 14.387 | 0.00013566 | 3.8676 | 0.0011479 | SERPINB4 |
| 1003_at | -14.385 | 0.00013571 | 3.8674 | 0.0011479 | CDH5 |
| 7223_at | -14.37 | 0.00013628 | 3.8656 | 0.0011496 | TRPC4 |
| 444_at | -14.368 | 0.00013636 | 3.8653 | 0.0011496 | ASPH |
| 157638_at | -14.354 | 0.00013687 | 3.8637 | 0.0011519 | FAM84B |
| 1054_at | -14.338 | 0.00013748 | 3.8618 | 0.0011524 | CEBPG |
| 2222_at | -14.337 | 0.00013754 | 3.8616 | 0.0011524 | FDFT1 |
| 101928079_at | -14.334 | 0.00013762 | 3.8613 | 0.0011524 | LINC01057 |
| 6801_at | -14.316 | 0.00013831 | 3.8592 | 0.0011562 | STRN |
| 79745_at | -14.308 | 0.00013864 | 3.8581 | 0.001157 | CLIP4 |
| 9076_at | -14.295 | 0.00013911 | 3.8566 | 0.001159 | CLDN1 |
| 3382_at | -14.272 | 0.00014 | 3.8539 | 0.0011634 | ICA1 |
| 26002_at | -14.269 | 0.0001401 | 3.8535 | 0.0011634 | MOXD1 |
| 80005_at | -14.25 | 0.00014086 | 3.8512 | 0.0011677 | DOCK5 |
| 79977_at | -14.241 | 0.00014121 | 3.8501 | 0.0011687 | GRHL2 |
| 5325_at | 14.214 | 0.00014228 | 3.8469 | 0.0011738 | PLAGL1 |
| 3983_at | 14.213 | 0.00014229 | 3.8468 | 0.0011738 | ABLIM1 |
| 10276_at | -14.192 | 0.00014312 | 3.8443 | 0.0011785 | NET1 |
| 7037_at | 14.187 | 0.00014334 | 3.8436 | 0.0011785 | TFRC |
| 196410_at | 14.171 | 0.00014397 | 3.8417 | 0.0011806 | METTL7B |
| 440_at | -14.164 | 0.00014423 | 3.8409 | 0.0011806 | ASNS |
| 10397_at | 14.157 | 0.00014451 | 3.8401 | 0.0011806 | NDRG1 |
| 4938_at | -14.157 | 0.00014453 | 3.84 | 0.0011806 | OAS1 |
| 3257_at | 14.147 | 0.00014493 | 3.8389 | 0.0011819 | HPS1 |
| 7070_at | 14.136 | 0.00014536 | 3.8375 | 0.0011823 | THY1 |
| 3199_at | -14.134 | 0.00014546 | 3.8373 | 0.0011823 | HOXA2 |
| 1520_at | -14.125 | 0.00014584 | 3.8361 | 0.0011835 | CTSS |
| 29995_at | 14.109 | 0.00014647 | 3.8343 | 0.0011867 | LMCD1 |
| 79986_at | -14.095 | 0.00014706 | 3.8325 | 0.0011874 | ZNF702P |
| 9685_at | -14.093 | 0.00014713 | 3.8323 | 0.0011874 | CLINT1 |
| 79188_at | 14.087 | 0.00014736 | 3.8316 | 0.0011874 | TMEM43 |
| 12_at | -14.081 | 0.00014762 | 3.8308 | 0.0011874 | SERPINA3 |
| 384_at | 14.078 | 0.00014775 | 3.8305 | 0.0011874 | ARG2 |
| 84679_at | -14.06 | 0.00014848 | 3.8283 | 0.0011914 | SLC9A7 |
| 3880_at | -14.041 | 0.0001493 | 3.8259 | 0.0011957 | KRT19 |
| 9564_at | 14.036 | 0.0001495 | 3.8254 | 0.0011957 | BCAR1 |
| 4144_at | 14.016 | 0.00015034 | 3.8229 | 0.0012006 | MAT2A |
| 3992_at | -13.98 | 0.00015187 | 3.8185 | 0.0012108 | FADS1 |
| 1846_at | -13.959 | 0.00015278 | 3.8159 | 0.0012162 | DUSP4 |
| 6397_at | -13.951 | 0.00015312 | 3.815 | 0.0012169 | SEC14L1 |
| 27122_at | 13.932 | 0.00015394 | 3.8126 | 0.0012214 | DKK3 |
| 1808_at | 13.926 | 0.00015418 | 3.812 | 0.0012214 | DPYSL2 |
| 5920_at | -13.899 | 0.00015536 | 3.8087 | 0.0012288 | RARRES3 |
| 151887_at | 13.89 | 0.00015577 | 3.8075 | 0.0012301 | CCDC80 |
| 205_at | 13.882 | 0.00015612 | 3.8065 | 0.001231 | AK4 |
| 3691_at | -13.875 | 0.00015644 | 3.8057 | 0.0012315 | ITGB4 |
| 64135_at | -13.864 | 0.00015693 | 3.8043 | 0.001232 | IFIH1 |
| 93663_at | -13.862 | 0.00015699 | 3.8041 | 0.001232 | ARHGAP18 |
| 3624_at | -13.851 | 0.00015748 | 3.8028 | 0.0012339 | INHBA |
| 3233_at | 13.831 | 0.0001584 | 3.8002 | 0.0012361 | HOXD4 |
| 3281_at | 13.828 | 0.00015853 | 3.7999 | 0.0012361 | HSBP1 |
| 9568_at | 13.826 | 0.0001586 | 3.7997 | 0.0012361 | GABBR2 |
| 10123_at | 13.823 | 0.00015875 | 3.7993 | 0.0012361 | ARL4C |
| 23586_at | -13.805 | 0.00015956 | 3.7971 | 0.0012405 | DDX58 |
| 8501_at | -13.788 | 0.00016035 | 3.7949 | 0.0012447 | SLC43A1 |
| 259232_at | -13.772 | 0.0001611 | 3.7929 | 0.0012486 | NALCN |
| 6001_at | -13.755 | 0.00016188 | 3.7908 | 0.0012527 | RGS10 |
| 6364_at | -13.749 | 0.00016216 | 3.7901 | 0.0012529 | CCL20 |
| 27134_at | -13.734 | 0.00016284 | 3.7883 | 0.0012562 | TJP3 |
| 23081_at | 13.721 | 0.00016345 | 3.7866 | 0.001259 | KDM4C |
| 7155_at | -13.711 | 0.0001639 | 3.7854 | 0.0012605 | TOP2B |
| 2244_at | 13.695 | 0.00016465 | 3.7834 | 0.0012628 | FGB |
| 6372_at | -13.691 | 0.00016486 | 3.7829 | 0.0012628 | CXCL6 |
| 5244_at | -13.689 | 0.00016495 | 3.7826 | 0.0012628 | ABCB4 |
| 9771_at | -13.671 | 0.00016581 | 3.7804 | 0.0012633 | RAPGEF5 |
| 3487_at | 13.669 | 0.0001659 | 3.7801 | 0.0012633 | IGFBP4 |
| 8644_at | 13.669 | 0.00016592 | 3.7801 | 0.0012633 | AKR1C3 |
| 100133172_at | 13.666 | 0.00016603 | 3.7798 | 0.0012633 | FAM66A |
| 241_at | -13.654 | 0.00016664 | 3.7782 | 0.001266 | ALOX5AP |
| 2200_at | 13.644 | 0.00016709 | 3.7771 | 0.0012675 | FBN1 |
| 4826_at | -13.637 | 0.00016746 | 3.7761 | 0.0012684 | NNAT |
| 3730_at | -13.627 | 0.00016791 | 3.7749 | 0.0012695 | KAL1 |
| 10584_at | -13.623 | 0.00016812 | 3.7744 | 0.0012695 | COLEC10 |
| 394_at | -13.592 | 0.00016964 | 3.7705 | 0.0012773 | ARHGAP5 |
| 81788_at | -13.591 | 0.00016966 | 3.7704 | 0.0012773 | NUAK2 |
| 5999_at | 13.58 | 0.00017023 | 3.769 | 0.0012791 | RGS4 |
| 7097_at | -13.568 | 0.00017081 | 3.7675 | 0.0012791 | TLR2 |
| 7805_at | -13.568 | 0.00017084 | 3.7674 | 0.0012791 | LAPTM5 |
| 9839_at | 13.565 | 0.00017096 | 3.7671 | 0.0012791 | ZEB2 |
| 9050_at | -13.561 | 0.00017117 | 3.7666 | 0.0012791 | PSTPIP2 |
| 112616_at | -13.554 | 0.00017152 | 3.7657 | 0.0012798 | CMTM7 |
| 256987_at | -13.529 | 0.00017278 | 3.7625 | 0.0012844 | SERINC5 |
| 54982_at | 13.521 | 0.00017314 | 3.7616 | 0.0012844 | CLN6 |
| 1861_at | 13.521 | 0.00017314 | 3.7616 | 0.0012844 | TOR1A |
| 400451_at | -13.521 | 0.00017317 | 3.7615 | 0.0012844 | FAM174B |
| 2150_at | -13.503 | 0.00017408 | 3.7592 | 0.0012893 | F2RL1 |
| 79772_at | -13.489 | 0.00017479 | 3.7575 | 0.0012898 | MCTP1 |
| 9734_at | -13.486 | 0.00017492 | 3.7572 | 0.0012898 | HDAC9 |
| 100873962_at | 13.486 | 0.00017493 | 3.7571 | 0.0012898 | LINC00278 |
| 59350_at | -13.472 | 0.00017566 | 3.7553 | 0.0012913 | RXFP1 |
| 92092_at | 13.467 | 0.00017591 | 3.7547 | 0.0012913 | ZC3HAV1L |
| 81553_at | 13.467 | 0.00017591 | 3.7547 | 0.0012913 | FAM49A |
| 100128252_at | 13.449 | 0.00017683 | 3.7524 | 0.0012962 | ZNF667-AS1 |
| 1728_at | 13.43 | 0.00017783 | 3.75 | 0.0013015 | NQO1 |
| 5099_at | -13.42 | 0.00017833 | 3.7488 | 0.0013034 | PCDH7 |
| 220213_at | -13.396 | 0.0001796 | 3.7457 | 0.0013107 | OTUD1 |
| 29109_at | 13.389 | 0.00017996 | 3.7448 | 0.0013114 | FHOD1 |
| 7404_at | -13.382 | 0.00018033 | 3.7439 | 0.0013122 | UTY |
| 63917_at | 13.373 | 0.00018083 | 3.7427 | 0.0013129 | GALNT11 |
| 345079_at | -13.362 | 0.00018142 | 3.7413 | 0.0013129 | SOWAHB |
| 55258_at | 13.36 | 0.00018147 | 3.7412 | 0.0013129 | THNSL2 |
| 10079_at | -13.36 | 0.00018148 | 3.7412 | 0.0013129 | ATP9A |
| 123263_at | 13.352 | 0.00018191 | 3.7401 | 0.0013141 | MTFMT |
| 200162_at | 13.338 | 0.00018267 | 3.7383 | 0.0013177 | SPAG17 |
| 55199_at | 13.33 | 0.00018311 | 3.7373 | 0.001319 | FAM86C1 |
| 55966_at | -13.311 | 0.00018416 | 3.7348 | 0.0013247 | AJAP1 |
| 8792_at | -13.301 | 0.00018466 | 3.7336 | 0.0013263 | TNFRSF11A |
| 55715_at | 13.296 | 0.00018496 | 3.7329 | 0.0013266 | DOK4 |
| 347902_at | -13.29 | 0.00018528 | 3.7322 | 0.0013269 | AMIGO2 |
| 6744_at | -13.267 | 0.00018655 | 3.7292 | 0.0013341 | SSFA2 |
| 199731_at | 13.254 | 0.00018727 | 3.7275 | 0.0013374 | CADM4 |
| 8287_at | -13.246 | 0.00018771 | 3.7265 | 0.0013381 | USP9Y |
| 10162_at | 13.243 | 0.0001879 | 3.7261 | 0.0013381 | LPCAT3 |
| 79679_at | -13.21 | 0.00018971 | 3.7219 | 0.001349 | VTCN1 |
| 18_at | 13.198 | 0.00019038 | 3.7204 | 0.0013518 | ABAT |
| 8611_at | 13.182 | 0.00019131 | 3.7183 | 0.0013565 | PPAP2A |
| 152816_at | 13.166 | 0.00019222 | 3.7162 | 0.001359 | C4orf26 |
| 83660_at | 13.164 | 0.00019236 | 3.7159 | 0.001359 | TLN2 |
| 6472_at | -13.162 | 0.00019248 | 3.7156 | 0.001359 | SHMT2 |
| 57727_at | 13.149 | 0.00019318 | 3.714 | 0.0013621 | NCOA5 |
| 9547_at | 13.124 | 0.00019467 | 3.7107 | 0.0013691 | CXCL14 |
| 5423_at | -13.123 | 0.00019473 | 3.7106 | 0.0013691 | POLB |
| 10765_at | -13.11 | 0.00019546 | 3.7089 | 0.0013704 | KDM5B |
| 29923_at | 13.11 | 0.00019546 | 3.7089 | 0.0013704 | HILPDA |
| 6809_at | 13.096 | 0.00019628 | 3.7071 | 0.001374 | STX3 |
| 339400_at | 13.086 | 0.00019685 | 3.7059 | 0.001374 | FLG-AS1 |
| 51400_at | 13.086 | 0.00019691 | 3.7057 | 0.001374 | PPME1 |
| 1845_at | 13.081 | 0.0001972 | 3.7051 | 0.001374 | DUSP3 |
| 196527_at | -13.076 | 0.00019749 | 3.7045 | 0.001374 | ANO6 |
| 55163_at | 13.073 | 0.00019762 | 3.7042 | 0.001374 | PNPO |
| 255928_at | -13.053 | 0.00019882 | 3.7015 | 0.0013774 | SYT14 |
| 1634_at | 13.051 | 0.00019895 | 3.7013 | 0.0013774 | DCN |
| 54836_at | -13.048 | 0.00019912 | 3.7009 | 0.0013774 | BSPRY |
| 112817_at | 13.047 | 0.00019921 | 3.7007 | 0.0013774 | HOGA1 |
| 149478_at | 13.017 | 0.00020102 | 3.6968 | 0.001388 | BTBD19 |
| 26579_at | -12.996 | 0.00020227 | 3.6941 | 0.0013947 | MYEOV |
| 7145_at | 12.984 | 0.00020303 | 3.6924 | 0.001398 | TNS1 |
| 5576_at | 12.964 | 0.00020425 | 3.6898 | 0.0014028 | PRKAR2A |
| 376940_at | -12.962 | 0.00020436 | 3.6896 | 0.0014028 | ZC3H6 |
| 83930_at | 12.959 | 0.00020458 | 3.6891 | 0.0014028 | STARD3NL |
| 10924_at | 12.943 | 0.00020557 | 3.687 | 0.0014078 | SMPDL3A |
| 79158_at | 12.919 | 0.00020704 | 3.684 | 0.0014151 | GNPTAB |
| 402569_at | -12.917 | 0.00020721 | 3.6836 | 0.0014151 | KPNA7 |
| 54463_at | 12.904 | 0.00020803 | 3.6819 | 0.0014187 | FAM134B |
| 83667_at | 12.874 | 0.0002099 | 3.678 | 0.0014287 | SESN2 |
| 92249_at | 12.872 | 0.00021006 | 3.6777 | 0.0014287 | LINC01278 |
| 9282_at | -12.861 | 0.00021075 | 3.6762 | 0.00143 | MED14 |
| 22854_at | 12.86 | 0.00021082 | 3.6761 | 0.00143 | NTNG1 |
| 2149_at | 12.833 | 0.00021256 | 3.6725 | 0.0014398 | F2R |
| 3491_at | -12.814 | 0.0002138 | 3.67 | 0.0014463 | CYR61 |
| 93664_at | -12.792 | 0.00021523 | 3.6671 | 0.001454 | CADPS2 |
| 10929_at | 12.774 | 0.00021642 | 3.6647 | 0.0014601 | SRSF8 |
| 7109_at | -12.763 | 0.00021717 | 3.6632 | 0.0014631 | TRAPPC10 |
| 84456_at | -12.752 | 0.00021792 | 3.6617 | 0.0014654 | L3MBTL3 |
| 10406_at | -12.747 | 0.0002182 | 3.6611 | 0.0014654 | WFDC2 |
| 23165_at | -12.743 | 0.00021851 | 3.6605 | 0.0014654 | NUP205 |
| 5655_at | -12.74 | 0.00021868 | 3.6602 | 0.0014654 | KLK10 |
| 55655_at | -12.72 | 0.00022002 | 3.6575 | 0.0014693 | NLRP2 |
| 6373_at | -12.716 | 0.00022034 | 3.6569 | 0.0014693 | CXCL11 |
| 51347_at | -12.715 | 0.00022042 | 3.6568 | 0.0014693 | TAOK3 |
| 51278_at | -12.714 | 0.00022045 | 3.6567 | 0.0014693 | IER5 |
| 79689_at | -12.703 | 0.00022122 | 3.6552 | 0.0014693 | STEAP4 |
| 253260_at | -12.702 | 0.00022128 | 3.6551 | 0.0014693 | RICTOR |
| 10085_at | -12.701 | 0.00022132 | 3.655 | 0.0014693 | EDIL3 |
| 30846_at | 12.677 | 0.000223 | 3.6517 | 0.0014785 | EHD2 |
| 51097_at | -12.647 | 0.00022508 | 3.6477 | 0.0014894 | SCCPDH |
| 39_at | -12.645 | 0.00022523 | 3.6474 | 0.0014894 | ACAT2 |
| 54842_at | -12.632 | 0.00022611 | 3.6457 | 0.0014931 | MFSD6 |
| 11138_at | -12.619 | 0.000227 | 3.644 | 0.001497 | TBC1D8 |
| 4241_at | -12.614 | 0.00022739 | 3.6432 | 0.0014977 | MFI2 |
| 582_at | 12.602 | 0.00022825 | 3.6416 | 0.0015003 | BBS1 |
| 653857_at | 12.6 | 0.00022839 | 3.6413 | 0.0015003 | ACTR3C |
| 55196_at | -12.571 | 0.00023047 | 3.6374 | 0.001512 | KIAA1551 |
| 387700_at | 12.554 | 0.00023164 | 3.6352 | 0.0015177 | SLC16A12 |
| 54464_at | -12.524 | 0.00023388 | 3.631 | 0.0015285 | XRN1 |
| 2810_at | -12.523 | 0.0002339 | 3.631 | 0.0015285 | SFN |
| 114884_at | 12.518 | 0.00023429 | 3.6303 | 0.001529 | OSBPL10 |
| 6505_at | 12.509 | 0.00023492 | 3.6291 | 0.0015292 | SLC1A1 |
| 479_at | -12.509 | 0.00023493 | 3.6291 | 0.0015292 | ATP12A |
| 1843_at | 12.5 | 0.00023562 | 3.6278 | 0.0015297 | DUSP1 |
| 10142_at | -12.5 | 0.00023562 | 3.6278 | 0.0015297 | AKAP9 |
| 22801_at | 12.481 | 0.00023703 | 3.6252 | 0.0015337 | ITGA11 |
| 160335_at | -12.48 | 0.00023714 | 3.625 | 0.0015337 | TMTC2 |
| 6575_at | -12.479 | 0.00023716 | 3.625 | 0.0015337 | SLC20A2 |
| 9819_at | 12.471 | 0.00023775 | 3.6239 | 0.0015355 | TSC22D2 |
| 100506658_at | -12.461 | 0.00023854 | 3.6224 | 0.0015363 | OCLN |
| 79572_at | 12.459 | 0.0002387 | 3.6221 | 0.0015363 | ATP13A3 |
| 389337_at | -12.454 | 0.00023904 | 3.6215 | 0.0015363 | ARHGEF37 |
| 5034_at | 12.453 | 0.0002391 | 3.6214 | 0.0015363 | P4HB |
| 57194_at | 12.426 | 0.00024119 | 3.6176 | 0.0015464 | ATP10A |
| 4093_at | 12.423 | 0.0002414 | 3.6173 | 0.0015464 | SMAD9 |
| 6091_at | -12.418 | 0.00024175 | 3.6166 | 0.0015464 | ROBO1 |
| 100507098_at | 12.416 | 0.0002419 | 3.6164 | 0.0015464 | ADAMTS9-AS2 |
| 692197_at | -12.404 | 0.00024284 | 3.6147 | 0.0015492 | SNORD77 |
| 54977_at | -12.402 | 0.00024296 | 3.6145 | 0.0015492 | SLC25A38 |
| 10659_at | -12.395 | 0.00024352 | 3.6135 | 0.0015501 | CELF2 |
| 3708_at | 12.392 | 0.00024373 | 3.6131 | 0.0015501 | ITPR1 |
| 10846_at | 12.375 | 0.00024508 | 3.6107 | 0.0015568 | PDE10A |
| 57460_at | -12.361 | 0.00024619 | 3.6087 | 0.0015569 | PPM1H |
| 6835_at | 12.36 | 0.00024622 | 3.6087 | 0.0015569 | SURF2 |
| 147339_at | -12.36 | 0.00024622 | 3.6087 | 0.0015569 | C18orf25 |
| 7026_at | 12.359 | 0.00024635 | 3.6084 | 0.0015569 | NR2F2 |
| 54467_at | -12.351 | 0.00024693 | 3.6074 | 0.0015586 | ANKIB1 |
| 1075_at | 12.321 | 0.00024933 | 3.6032 | 0.0015718 | CTSC |
| 4646_at | -12.312 | 0.00025002 | 3.602 | 0.0015741 | MYO6 |
| 5743_at | -12.303 | 0.00025074 | 3.6008 | 0.0015766 | PTGS2 |
| 1767_at | 12.293 | 0.00025152 | 3.5994 | 0.0015772 | DNAH5 |
| 6894_at | -12.292 | 0.00025162 | 3.5993 | 0.0015772 | TARBP1 |
| 7073_at | -12.29 | 0.00025177 | 3.599 | 0.0015772 | TIAL1 |
| 8506_at | -12.286 | 0.00025209 | 3.5984 | 0.0015772 | CNTNAP1 |
| 55748_at | 12.277 | 0.00025281 | 3.5972 | 0.0015797 | CNDP2 |
| 7477_at | -12.26 | 0.00025422 | 3.5948 | 0.0015864 | WNT7B |
| 4256_at | 12.256 | 0.00025451 | 3.5943 | 0.0015864 | MGP |
| 79974_at | -12.245 | 0.00025543 | 3.5927 | 0.00159 | CPED1 |
| 3627_at | -12.241 | 0.00025572 | 3.5922 | 0.00159 | CXCL10 |
| 330_at | -12.23 | 0.0002566 | 3.5907 | 0.0015935 | BIRC3 |
| 30815_at | 12.226 | 0.000257 | 3.5901 | 0.001594 | ST6GALNAC6 |
| 26234_at | -12.209 | 0.00025839 | 3.5877 | 0.0016006 | FBXL5 |
| 388115_at | -12.196 | 0.00025948 | 3.5859 | 0.0016054 | C15orf52 |
| 116362_at | -12.179 | 0.00026084 | 3.5836 | 0.0016118 | RBP7 |
| 8829_at | 12.161 | 0.00026243 | 3.581 | 0.0016196 | NRP1 |
| 54978_at | 12.148 | 0.00026347 | 3.5793 | 0.0016232 | SLC35F6 |
| 64792_at | 12.146 | 0.00026366 | 3.579 | 0.0016232 | IFT22 |
| 6382_at | 12.137 | 0.00026444 | 3.5777 | 0.001626 | SDC1 |
| 3226_at | 12.128 | 0.00026523 | 3.5764 | 0.0016274 | HOXC10 |
| 22795_at | 12.119 | 0.00026598 | 3.5751 | 0.0016274 | NID2 |
| 3131_at | 12.119 | 0.00026599 | 3.5751 | 0.0016274 | HLF |
| 2534_at | -12.117 | 0.00026616 | 3.5749 | 0.0016274 | FYN |
| 1946_at | 12.115 | 0.0002663 | 3.5746 | 0.0016274 | EFNA5 |
| 2355_at | 12.108 | 0.00026691 | 3.5736 | 0.0016281 | FOSL2 |
| 3460_at | -12.101 | 0.00026749 | 3.5727 | 0.0016281 | IFNGR2 |
| 3485_at | -12.098 | 0.00026779 | 3.5722 | 0.0016281 | IGFBP2 |
| 8817_at | 12.095 | 0.00026803 | 3.5718 | 0.0016281 | FGF18 |
| 7476_at | -12.095 | 0.00026803 | 3.5718 | 0.0016281 | WNT7A |
| 9536_at | -12.033 | 0.00027345 | 3.5631 | 0.001659 | PTGES |
| 886_at | 12.025 | 0.00027418 | 3.562 | 0.0016614 | CCKAR |
| 64077_at | 12.014 | 0.00027514 | 3.5604 | 0.0016652 | LHPP |
| 1140_at | -11.995 | 0.00027692 | 3.5576 | 0.0016715 | CHRNB1 |
| 6732_at | -11.992 | 0.00027712 | 3.5573 | 0.0016715 | SRPK1 |
| 9712_at | -11.992 | 0.00027719 | 3.5572 | 0.0016715 | USP6NL |
| 79710_at | 11.983 | 0.000278 | 3.556 | 0.001672 | MORC4 |
| 100507062_at | -11.98 | 0.00027821 | 3.5556 | 0.001672 | PSMD6-AS2 |
| 10371_at | -11.98 | 0.00027827 | 3.5555 | 0.001672 | SEMA3A |
| 219790_at | -11.968 | 0.00027929 | 3.5539 | 0.0016757 | RTKN2 |
| 51421_at | -11.965 | 0.00027957 | 3.5535 | 0.0016757 | AMOTL2 |
| 56655_at | 11.958 | 0.00028026 | 3.5524 | 0.0016779 | POLE4 |
| 80824_at | -11.953 | 0.00028072 | 3.5517 | 0.0016786 | DUSP16 |
| 158471_at | 11.94 | 0.00028192 | 3.5499 | 0.0016838 | PRUNE2 |
| 79730_at | -11.928 | 0.00028304 | 3.5482 | 0.0016884 | NSUN7 |
| 401720_at | 11.905 | 0.00028518 | 3.5449 | 0.0016992 | FIGNL2 |
| 25825_at | -11.887 | 0.00028686 | 3.5423 | 0.0017072 | BACE2 |
| 145741_at | 11.878 | 0.00028769 | 3.5411 | 0.00171 | C2CD4A |
| 57216_at | -11.869 | 0.00028856 | 3.5398 | 0.0017132 | VANGL2 |
| 27248_at | -11.848 | 0.00029051 | 3.5368 | 0.0017224 | ERLEC1 |
| 388962_at | 11.845 | 0.0002908 | 3.5364 | 0.0017224 | BOLA3 |
| 59338_at | -11.839 | 0.00029142 | 3.5355 | 0.001724 | PLEKHA1 |
| 23321_at | -11.832 | 0.00029213 | 3.5344 | 0.0017253 | TRIM2 |
| 23780_at | -11.828 | 0.00029245 | 3.534 | 0.0017253 | APOL2 |
| 5947_at | -11.826 | 0.00029268 | 3.5336 | 0.0017253 | RBP1 |
| 1514_at | 11.817 | 0.00029352 | 3.5324 | 0.0017267 | CTSL |
| 401505_at | 11.816 | 0.00029359 | 3.5323 | 0.0017267 | TOMM5 |
| 4864_at | 11.804 | 0.00029483 | 3.5304 | 0.0017319 | NPC1 |
| 55103_at | -11.799 | 0.00029532 | 3.5297 | 0.0017328 | RALGPS2 |
| 395_at | 11.786 | 0.00029661 | 3.5278 | 0.0017378 | ARHGAP6 |
| 3720_at | -11.783 | 0.00029688 | 3.5274 | 0.0017378 | JARID2 |
| 2058_at | -11.762 | 0.00029891 | 3.5245 | 0.0017477 | EPRS |
| 81704_at | 11.752 | 0.0002999 | 3.523 | 0.0017514 | DOCK8 |
| 958_at | -11.748 | 0.00030034 | 3.5224 | 0.0017519 | CD40 |
| 7124_at | -11.74 | 0.0003011 | 3.5213 | 0.0017543 | TNF |
| 9289_at | 11.728 | 0.00030238 | 3.5194 | 0.0017597 | GPR56 |
| 1809_at | 11.72 | 0.00030318 | 3.5183 | 0.0017623 | DPYSL3 |
| 9473_at | -11.713 | 0.00030381 | 3.5174 | 0.0017639 | THEMIS2 |
| 10346_at | -11.708 | 0.0003044 | 3.5166 | 0.0017643 | TRIM22 |
| 7694_at | 11.706 | 0.00030459 | 3.5163 | 0.0017643 | ZNF135 |
| 100507056_at | -11.701 | 0.00030511 | 3.5155 | 0.0017651 | CCAT1 |
| 51019_at | 11.698 | 0.00030542 | 3.5151 | 0.0017651 | CCDC53 |
| 56954_at | 11.689 | 0.00030633 | 3.5138 | 0.0017664 | NIT2 |
| 1105_at | -11.685 | 0.00030666 | 3.5133 | 0.0017664 | CHD1 |
| 4148_at | 11.685 | 0.0003067 | 3.5133 | 0.0017664 | MATN3 |
| 64600_at | -11.674 | 0.00030781 | 3.5117 | 0.0017707 | PLA2G2F |
| 1906_at | -11.668 | 0.00030845 | 3.5108 | 0.0017724 | EDN1 |
| 26154_at | -11.653 | 0.00031003 | 3.5086 | 0.0017794 | ABCA12 |
| 9055_at | -11.645 | 0.00031081 | 3.5075 | 0.0017818 | PRC1 |
| 154664_at | -11.634 | 0.00031194 | 3.5059 | 0.0017862 | ABCA13 |
| 54509_at | -11.625 | 0.0003129 | 3.5046 | 0.0017897 | RHOF |
| 23187_at | 11.619 | 0.00031354 | 3.5037 | 0.0017913 | PHLDB1 |
| 54984_at | 11.613 | 0.00031417 | 3.5028 | 0.0017919 | PINX1 |
| 23353_at | -11.611 | 0.00031437 | 3.5026 | 0.0017919 | SUN1 |
| 5358_at | -11.601 | 0.00031546 | 3.5011 | 0.0017961 | PLS3 |
| 64976_at | 11.592 | 0.00031643 | 3.4997 | 0.0017982 | MRPL40 |
| 3635_at | -11.589 | 0.00031677 | 3.4993 | 0.0017982 | INPP5D |
| 9518_at | -11.588 | 0.00031691 | 3.4991 | 0.0017982 | GDF15 |
| 5569_at | -11.571 | 0.00031869 | 3.4966 | 0.0018063 | PKIA |
| 26470_at | 11.506 | 0.00032578 | 3.4871 | 0.0018444 | SEZ6L2 |
| 79817_at | -11.496 | 0.00032688 | 3.4856 | 0.0018479 | MOB3B |
| 7077_at | -11.493 | 0.00032715 | 3.4853 | 0.0018479 | TIMP2 |
| 154761_at | 11.485 | 0.00032812 | 3.484 | 0.0018513 | LOC154761 |
| 388_at | 11.456 | 0.00033133 | 3.4797 | 0.0018658 | RHOB |
| 3422_at | -11.455 | 0.00033144 | 3.4796 | 0.0018658 | IDI1 |
| 7074_at | -11.449 | 0.00033217 | 3.4786 | 0.0018658 | TIAM1 |
| 2254_at | 11.447 | 0.00033238 | 3.4784 | 0.0018658 | FGF9 |
| 101927991_at | 11.445 | 0.00033255 | 3.4781 | 0.0018658 | LOC101927991 |
| 10020_at | -11.425 | 0.0003349 | 3.4751 | 0.0018766 | GNE |
| 54566_at | -11.421 | 0.00033526 | 3.4746 | 0.0018766 | EPB41L4B |
| 4201_at | 11.419 | 0.0003356 | 3.4742 | 0.0018766 | MEA1 |
| 54890_at | 11.41 | 0.00033655 | 3.473 | 0.0018798 | ALKBH5 |
| 6317_at | 11.401 | 0.00033756 | 3.4716 | 0.0018819 | SERPINB3 |
| 5066_at | 11.4 | 0.00033769 | 3.4715 | 0.0018819 | PAM |
| 79956_at | -11.377 | 0.00034037 | 3.468 | 0.001893 | ERMP1 |
| 693230_at | 11.377 | 0.00034042 | 3.468 | 0.001893 | MIR645 |
| 57530_at | -11.373 | 0.00034085 | 3.4674 | 0.0018932 | CGN |
| 90993_at | 11.365 | 0.00034178 | 3.4663 | 0.0018963 | CREB3L1 |
| 666_at | 11.357 | 0.00034278 | 3.465 | 0.0018997 | BOK |
| 5744_at | 11.351 | 0.00034351 | 3.4641 | 0.0019017 | PTHLH |
| 26353_at | 11.328 | 0.00034621 | 3.4607 | 0.0019136 | HSPB8 |
| 53373_at | -11.326 | 0.00034643 | 3.4604 | 0.0019136 | TPCN1 |
| 100507334_at | -11.323 | 0.00034681 | 3.4599 | 0.0019136 | LOC100507334 |
| 7025_at | 11.318 | 0.00034742 | 3.4591 | 0.0019149 | NR2F1 |
| 1040_at | -11.311 | 0.00034819 | 3.4582 | 0.001917 | CDS1 |
| 80162_at | -11.293 | 0.00035037 | 3.4555 | 0.0019268 | ATHL1 |
| 51704_at | -11.29 | 0.00035081 | 3.4549 | 0.0019271 | GPRC5B |
| 92999_at | 11.281 | 0.00035178 | 3.4537 | 0.0019304 | ZBTB47 |
| 25818_at | -11.273 | 0.00035285 | 3.4524 | 0.0019312 | KLK5 |
| 4277_at | -11.269 | 0.00035331 | 3.4518 | 0.0019312 | MICB |
| 6728_at | -11.269 | 0.00035332 | 3.4518 | 0.0019312 | SRP19 |
| 54704_at | -11.268 | 0.00035348 | 3.4516 | 0.0019312 | PDP1 |
| 169200_at | 11.235 | 0.0003575 | 3.4467 | 0.0019468 | TMEM64 |
| 284021_at | -11.233 | 0.0003577 | 3.4465 | 0.0019468 | MILR1 |
| 79729_at | 11.232 | 0.00035784 | 3.4463 | 0.0019468 | SH3D21 |
| 23491_at | 11.232 | 0.0003579 | 3.4462 | 0.0019468 | CES3 |
| 81563_at | -11.226 | 0.00035858 | 3.4454 | 0.0019484 | C1orf21 |
| 56926_at | 11.214 | 0.00036016 | 3.4435 | 0.0019549 | NCLN |
| 6571_at | 11.2 | 0.00036187 | 3.4414 | 0.001959 | SLC18A2 |
| 10331_at | -11.198 | 0.00036208 | 3.4412 | 0.001959 | B3GNT3 |
| 135228_at | 11.197 | 0.0003623 | 3.4409 | 0.001959 | CD109 |
| 285513_at | 11.195 | 0.00036248 | 3.4407 | 0.001959 | GPRIN3 |
| 9748_at | -11.192 | 0.0003629 | 3.4402 | 0.0019591 | SLK |
| 3026_at | 11.183 | 0.00036406 | 3.4388 | 0.0019633 | HABP2 |
| 57480_at | -11.179 | 0.00036446 | 3.4383 | 0.0019633 | PLEKHG1 |
| 80004_at | -11.166 | 0.00036622 | 3.4363 | 0.0019701 | ESRP2 |
| 54532_at | -11.163 | 0.00036652 | 3.4359 | 0.0019701 | USP53 |
| 221393_at | -11.156 | 0.00036741 | 3.4349 | 0.001972 | GPR115 |
| 4147_at | 11.155 | 0.00036765 | 3.4346 | 0.001972 | MATN2 |
| 57118_at | -11.147 | 0.00036867 | 3.4334 | 0.0019753 | CAMK1D |
| 7436_at | 11.143 | 0.00036909 | 3.4329 | 0.0019754 | VLDLR |
| 23198_at | -11.138 | 0.0003698 | 3.432 | 0.0019754 | PSME4 |
| 6690_at | -11.136 | 0.00037006 | 3.4317 | 0.0019754 | SPINK1 |
| 1741_at | -11.131 | 0.00037064 | 3.431 | 0.0019754 | DLG3 |
| 2590_at | 11.13 | 0.00037086 | 3.4308 | 0.0019754 | GALNT2 |
| 55014_at | -11.127 | 0.00037126 | 3.4303 | 0.0019754 | STX17 |
| 23635_at | 11.124 | 0.00037165 | 3.4299 | 0.0019754 | SSBP2 |
| 4716_at | 11.122 | 0.00037184 | 3.4296 | 0.0019754 | NDUFB10 |
| 114786_at | 11.109 | 0.00037354 | 3.4277 | 0.0019823 | XKR4 |
| 10110_at | 11.105 | 0.00037405 | 3.4271 | 0.0019829 | SGK2 |
| 89796_at | 11.099 | 0.0003748 | 3.4262 | 0.0019829 | NAV1 |
| 400566_at | 11.099 | 0.00037484 | 3.4262 | 0.0019829 | C17orf97 |
| 677811_at | -11.092 | 0.00037583 | 3.425 | 0.0019845 | SNORA28 |
| 3570_at | 11.091 | 0.00037594 | 3.4249 | 0.0019845 | IL6R |
| 654321_at | -11.073 | 0.00037831 | 3.4222 | 0.0019938 | SNORA75 |
| 64795_at | -11.07 | 0.00037868 | 3.4217 | 0.0019938 | RMND5A |
| 202_at | -11.068 | 0.00037891 | 3.4215 | 0.0019938 | AIM1 |
| 80036_at | 11.06 | 0.00038009 | 3.4201 | 0.0019961 | TRPM3 |
| 387914_at | 11.059 | 0.00038013 | 3.4201 | 0.0019961 | SHISA2 |
| 5654_at | 11.054 | 0.0003809 | 3.4192 | 0.001998 | HTRA1 |
| 122786_at | -11.041 | 0.00038253 | 3.4173 | 0.0020045 | FRMD6 |
| 7941_at | -11.033 | 0.00038367 | 3.416 | 0.0020076 | PLA2G7 |
| 10725_at | -11.031 | 0.00038392 | 3.4158 | 0.0020076 | NFAT5 |
| 23179_at | 11.014 | 0.0003862 | 3.4132 | 0.0020174 | RGL1 |
| 114327_at | 11.002 | 0.00038796 | 3.4112 | 0.0020244 | EFHC1 |
| 94120_at | 10.979 | 0.00039102 | 3.4078 | 0.0020383 | SYTL3 |
| 5552_at | 10.972 | 0.00039206 | 3.4067 | 0.0020415 | SRGN |
| 30818_at | 10.968 | 0.00039266 | 3.406 | 0.0020426 | KCNIP3 |
| 64167_at | -10.964 | 0.00039314 | 3.4055 | 0.0020429 | ERAP2 |
| 6584_at | 10.944 | 0.00039589 | 3.4024 | 0.0020515 | SLC22A5 |
| 2077_at | 10.944 | 0.00039594 | 3.4024 | 0.0020515 | ERF |
| 64145_at | -10.944 | 0.00039602 | 3.4023 | 0.0020515 | ZFYVE20 |
| 1802_at | 10.932 | 0.00039764 | 3.4005 | 0.0020544 | DPH2 |
| 22924_at | 10.926 | 0.00039845 | 3.3996 | 0.0020544 | MAPRE3 |
| 1999_at | -10.926 | 0.00039855 | 3.3995 | 0.0020544 | ELF3 |
| 9096_at | 10.921 | 0.00039918 | 3.3988 | 0.0020544 | TBX18 |
| 3248_at | 10.919 | 0.00039952 | 3.3985 | 0.0020544 | HPGD |
| 10256_at | -10.917 | 0.00039977 | 3.3982 | 0.0020544 | CNKSR1 |
| 100506178_at | -10.916 | 0.00039993 | 3.398 | 0.0020544 | LOC100506178 |
| 84620_at | 10.915 | 0.00040012 | 3.3978 | 0.0020544 | ST6GAL2 |
| 11279_at | 10.914 | 0.00040028 | 3.3976 | 0.0020544 | KLF8 |
| 131368_at | -10.897 | 0.0004027 | 3.395 | 0.0020646 | ZPLD1 |
| 10906_at | -10.894 | 0.00040313 | 3.3946 | 0.0020646 | TRAFD1 |
| 8463_at | -10.891 | 0.0004035 | 3.3942 | 0.0020646 | TEAD2 |
| 84451_at | 10.886 | 0.00040421 | 3.3934 | 0.0020661 | KIAA1804 |
| 27293_at | -10.881 | 0.00040489 | 3.3927 | 0.0020675 | SMPDL3B |
| 51203_at | -10.873 | 0.00040614 | 3.3913 | 0.0020696 | NUSAP1 |
| 1832_at | -10.872 | 0.00040622 | 3.3912 | 0.0020696 | DSP |
| 1428_at | 10.87 | 0.00040655 | 3.3909 | 0.0020696 | CRYM |
| 56951_at | 10.846 | 0.00041008 | 3.3871 | 0.0020839 | C5orf15 |
| 25976_at | 10.845 | 0.0004102 | 3.387 | 0.0020839 | TIPARP |
| 55971_at | -10.833 | 0.00041201 | 3.3851 | 0.002091 | BAIAP2L1 |
| 81607_at | -10.827 | 0.0004129 | 3.3842 | 0.0020934 | PVRL4 |
| 3417_at | -10.822 | 0.00041366 | 3.3834 | 0.0020951 | IDH1 |
| 27147_at | -10.806 | 0.00041603 | 3.3809 | 0.002105 | DENND2A |
| 154091_at | -10.796 | 0.00041747 | 3.3794 | 0.0021079 | SLC2A12 |
| 50863_at | -10.794 | 0.00041774 | 3.3791 | 0.0021079 | NTM |
| 23013_at | -10.794 | 0.00041786 | 3.379 | 0.0021079 | SPEN |
| 10497_at | 10.79 | 0.0004184 | 3.3784 | 0.0021085 | UNC13B |
| 9672_at | 10.786 | 0.00041897 | 3.3778 | 0.0021087 | SDC3 |
| 9180_at | 10.783 | 0.00041942 | 3.3773 | 0.0021087 | OSMR |
| 121551_at | 10.781 | 0.00041972 | 3.377 | 0.0021087 | BTBD11 |
| 93986_at | -10.766 | 0.00042208 | 3.3746 | 0.0021169 | FOXP2 |
| 4233_at | -10.765 | 0.00042218 | 3.3745 | 0.0021169 | MET |
| 463_at | -10.755 | 0.00042374 | 3.3729 | 0.0021225 | ZFHX3 |
| 3399_at | 10.749 | 0.00042458 | 3.372 | 0.0021225 | ID3 |
| 4616_at | -10.749 | 0.00042459 | 3.372 | 0.0021225 | GADD45B |
| 162394_at | -10.731 | 0.00042741 | 3.3692 | 0.0021345 | SLFN5 |
| 57720_at | -10.725 | 0.00042843 | 3.3681 | 0.0021374 | GPR107 |
| 28937_at | -10.715 | 0.00042991 | 3.3666 | 0.0021422 | IGKV1-17 |
| 57561_at | 10.713 | 0.00043027 | 3.3663 | 0.0021422 | ARRDC3 |
| 11099_at | -10.71 | 0.00043068 | 3.3658 | 0.0021422 | PTPN21 |
| 114548_at | -10.682 | 0.00043509 | 3.3614 | 0.002162 | NLRP3 |
| 7059_at | 10.671 | 0.00043683 | 3.3597 | 0.0021667 | THBS3 |
| 8239_at | -10.667 | 0.00043742 | 3.3591 | 0.0021667 | USP9X |
| 31_at | -10.663 | 0.00043816 | 3.3584 | 0.0021667 | ACACA |
| 144535_at | 10.662 | 0.00043829 | 3.3582 | 0.0021667 | CFAP54 |
| 1191_at | 10.661 | 0.00043839 | 3.3581 | 0.0021667 | CLU |
| 29956_at | 10.66 | 0.00043862 | 3.3579 | 0.0021667 | CERS2 |
| 7465_at | -10.651 | 0.00044001 | 3.3565 | 0.0021714 | WEE1 |
| 10194_at | 10.643 | 0.00044139 | 3.3552 | 0.0021744 | TSHZ1 |
| 727936_at | 10.641 | 0.00044168 | 3.3549 | 0.0021744 | GXYLT2 |
| 80339_at | -10.639 | 0.00044192 | 3.3547 | 0.0021744 | PNPLA3 |
| 30011_at | -10.621 | 0.00044496 | 3.3517 | 0.0021872 | SH3KBP1 |
| 50484_at | -10.598 | 0.00044869 | 3.3481 | 0.0022033 | RRM2B |
| 8794_at | -10.594 | 0.00044929 | 3.3475 | 0.0022041 | TNFRSF10C |
| 201475_at | -10.587 | 0.00045047 | 3.3463 | 0.0022077 | RAB12 |
| 4645_at | -10.577 | 0.00045204 | 3.3448 | 0.0022133 | MYO5B |
| 7389_at | 10.574 | 0.00045256 | 3.3443 | 0.0022136 | UROD |
| 55161_at | -10.56 | 0.000455 | 3.342 | 0.0022232 | TMEM33 |
| 54933_at | -10.557 | 0.0004554 | 3.3416 | 0.0022232 | RHBDL2 |
| 219_at | -10.548 | 0.00045697 | 3.3401 | 0.0022287 | ALDH1B1 |
| 5055_at | -10.545 | 0.00045741 | 3.3397 | 0.0022287 | SERPINB2 |
| 92370_at | -10.542 | 0.00045805 | 3.3391 | 0.0022296 | PXYLP1 |
| 376267_at | -10.535 | 0.0004591 | 3.3381 | 0.0022325 | RAB15 |
| 4783_at | 10.524 | 0.00046109 | 3.3362 | 0.0022366 | NFIL3 |
| 343450_at | 10.523 | 0.0004612 | 3.3361 | 0.0022366 | KCNT2 |
| 9929_at | -10.522 | 0.0004613 | 3.336 | 0.0022366 | JOSD1 |
| 91749_at | 10.52 | 0.00046173 | 3.3356 | 0.0022366 | KIAA1919 |
| 6272_at | -10.512 | 0.00046305 | 3.3344 | 0.0022405 | SORT1 |
| 22943_at | -10.51 | 0.00046342 | 3.334 | 0.0022405 | DKK1 |
| 5457_at | -10.501 | 0.00046495 | 3.3326 | 0.0022457 | POU4F1 |
| 3569_at | -10.489 | 0.00046709 | 3.3306 | 0.0022538 | IL6 |
| 154043_at | 10.483 | 0.00046805 | 3.3297 | 0.0022563 | CNKSR3 |
| 54069_at | -10.476 | 0.00046937 | 3.3285 | 0.0022605 | MIS18A |
| 285834_at | -10.459 | 0.00047225 | 3.3258 | 0.0022722 | HCG22 |
| 3887_at | -10.455 | 0.00047292 | 3.3252 | 0.0022732 | KRT81 |
| 6786_at | 10.44 | 0.00047559 | 3.3228 | 0.0022838 | STIM1 |
| 100507421_at | 10.436 | 0.00047638 | 3.3221 | 0.0022854 | TMEM178B |
| 64114_at | -10.432 | 0.00047695 | 3.3215 | 0.0022858 | TMBIM1 |
| 10014_at | -10.43 | 0.00047737 | 3.3211 | 0.0022858 | HDAC5 |
| 54414_at | 10.423 | 0.00047859 | 3.32 | 0.0022894 | SIAE |
| 3037_at | -10.412 | 0.00048051 | 3.3183 | 0.0022964 | HAS2 |
| 2888_at | 10.403 | 0.00048225 | 3.3167 | 0.0023025 | GRB14 |
| 154141_at | -10.399 | 0.00048294 | 3.3161 | 0.0023037 | MBOAT1 |
| 80021_at | -10.395 | 0.00048369 | 3.3154 | 0.002305 | TMEM62 |
| 353189_at | 10.392 | 0.00048419 | 3.315 | 0.0023052 | SLCO4C1 |
| 51523_at | 10.377 | 0.00048688 | 3.3126 | 0.002314 | CXXC5 |
| 101929771_at | 10.377 | 0.00048696 | 3.3125 | 0.002314 | LOC101929771 |
| 259173_at | -10.372 | 0.00048779 | 3.3118 | 0.0023157 | ALS2CL |
| 883_at | -10.364 | 0.00048932 | 3.3104 | 0.0023208 | CCBL1 |
| 5431_at | -10.342 | 0.00049327 | 3.3069 | 0.0023355 | POLR2B |
| 57538_at | -10.342 | 0.00049335 | 3.3068 | 0.0023355 | ALPK3 |
| 66008_at | 10.333 | 0.00049509 | 3.3053 | 0.0023415 | TRAK2 |
| 101928262_at | -10.327 | 0.00049613 | 3.3044 | 0.0023442 | LOC101928262 |
| 57715_at | 10.316 | 0.00049812 | 3.3027 | 0.0023511 | SEMA4G |
| 3242_at | 10.311 | 0.00049907 | 3.3018 | 0.0023511 | HPD |
| 100506548_at | -10.307 | 0.00049984 | 3.3012 | 0.0023511 | LOC100506548 |
| 54097_at | -10.306 | 0.00050014 | 3.3009 | 0.0023511 | FAM3B |
| 114991_at | -10.305 | 0.00050028 | 3.3008 | 0.0023511 | ZNF618 |
| 100506691_at | 10.304 | 0.00050041 | 3.3007 | 0.0023511 | LOC100506691 |
| 5308_at | 10.3 | 0.00050115 | 3.3 | 0.0023524 | PITX2 |
| 10717_at | -10.295 | 0.00050209 | 3.2992 | 0.0023545 | AP4B1 |
| 11245_at | -10.29 | 0.00050302 | 3.2984 | 0.0023567 | GPR176 |
| 101926978_at | 10.284 | 0.00050414 | 3.2975 | 0.0023597 | LINC01111 |
| 10783_at | 10.257 | 0.00050946 | 3.2929 | 0.0023807 | NEK6 |
| 10398_at | 10.252 | 0.00051028 | 3.2922 | 0.0023807 | MYL9 |
| 23657_at | -10.252 | 0.00051033 | 3.2921 | 0.0023807 | SLC7A11 |
| 57139_at | -10.249 | 0.00051087 | 3.2917 | 0.0023807 | RGL3 |
| 8031_at | 10.247 | 0.00051128 | 3.2913 | 0.0023807 | NCOA4 |
| 1717_at | -10.246 | 0.00051148 | 3.2912 | 0.0023807 | DHCR7 |
| 84951_at | -10.24 | 0.00051257 | 3.2902 | 0.0023827 | TNS4 |
| 23529_at | 10.239 | 0.00051286 | 3.29 | 0.0023827 | CLCF1 |
| 1655_at | -10.233 | 0.00051397 | 3.2891 | 0.0023856 | DDX5 |
| 55287_at | -10.221 | 0.00051631 | 3.2871 | 0.0023935 | TMEM40 |
| 54657_at | 10.22 | 0.00051662 | 3.2868 | 0.0023935 | UGT1A4 |
| 27242_at | 10.217 | 0.00051714 | 3.2864 | 0.0023937 | TNFRSF21 |
| 730005_at | 10.208 | 0.00051898 | 3.2849 | 0.0024 | SEC14L6 |
| 677802_at | -10.196 | 0.00052123 | 3.283 | 0.0024066 | SNORA14B |
| 200958_at | -10.196 | 0.00052137 | 3.2829 | 0.0024066 | MUC20 |
| 10243_at | 10.179 | 0.00052473 | 3.2801 | 0.0024183 | GPHN |
| 387496_at | 10.177 | 0.00052515 | 3.2797 | 0.0024183 | RASL11A |
| 9540_at | 10.176 | 0.00052536 | 3.2795 | 0.0024183 | TP53I3 |
| 55920_at | -10.168 | 0.00052689 | 3.2783 | 0.0024227 | RCC2 |
| 222584_at | -10.166 | 0.00052729 | 3.278 | 0.0024227 | FAM83B |
| 643314_at | -10.147 | 0.00053109 | 3.2748 | 0.002437 | KIAA0754 |
| 8560_at | 10.146 | 0.00053137 | 3.2746 | 0.002437 | DEGS1 |
| 56257_at | 10.131 | 0.00053437 | 3.2722 | 0.0024485 | MEPCE |
| 165215_at | -10.128 | 0.00053494 | 3.2717 | 0.0024489 | FAM171B |
| 348938_at | 10.122 | 0.00053618 | 3.2707 | 0.0024503 | NIPAL4 |
| 1238_at | -10.122 | 0.00053623 | 3.2706 | 0.0024503 | ACKR2 |
| 4982_at | -10.117 | 0.00053729 | 3.2698 | 0.0024529 | TNFRSF11B |
| 4763_at | -10.114 | 0.00053792 | 3.2693 | 0.0024535 | NF1 |
| 56110_at | 10.107 | 0.00053924 | 3.2682 | 0.0024551 | PCDHGA5 |
| 27324_at | -10.107 | 0.00053925 | 3.2682 | 0.0024551 | TOX3 |
| 5797_at | 10.09 | 0.00054286 | 3.2653 | 0.0024693 | PTPRM |
| 253959_at | -10.083 | 0.00054435 | 3.2641 | 0.0024738 | RALGAPA1 |
| 100506901_at | 10.076 | 0.00054581 | 3.263 | 0.0024782 | LOC100506901 |
| 23505_at | 10.072 | 0.00054664 | 3.2623 | 0.0024782 | TMEM131 |
| 80184_at | -10.071 | 0.00054681 | 3.2622 | 0.0024782 | CEP290 |
| 55125_at | -10.06 | 0.0005492 | 3.2603 | 0.0024868 | CEP192 |
| 9988_at | -10.043 | 0.00055276 | 3.2575 | 0.0025004 | DMTF1 |
| 53335_at | -10.041 | 0.0005532 | 3.2571 | 0.0025004 | BCL11A |
| 93099_at | -10.02 | 0.00055765 | 3.2536 | 0.0025173 | DMKN |
| 7093_at | 10.019 | 0.00055794 | 3.2534 | 0.0025173 | TLL2 |
| 65999_at | 10.013 | 0.00055914 | 3.2525 | 0.0025204 | LRRC61 |
| 91947_at | 10.005 | 0.00056098 | 3.2511 | 0.0025264 | ARRDC4 |
| 51742_at | -10.001 | 0.00056181 | 3.2504 | 0.0025279 | ARID4B |
| 5125_at | 9.9983 | 0.00056237 | 3.25 | 0.0025282 | PCSK5 |
| 57600_at | 9.9876 | 0.00056471 | 3.2482 | 0.0025364 | FNIP2 |
| 10903_at | -9.9774 | 0.00056695 | 3.2465 | 0.0025441 | MTMR11 |
| 54908_at | 9.9707 | 0.00056841 | 3.2453 | 0.0025484 | SPDL1 |
| 84251_at | -9.9628 | 0.00057017 | 3.244 | 0.002554 | SGIP1 |
| 4312_at | -9.9603 | 0.00057072 | 3.2436 | 0.0025542 | MMP1 |
| 3656_at | -9.9415 | 0.0005749 | 3.2404 | 0.0025706 | IRAK2 |
| 56261_at | -9.9389 | 0.0005755 | 3.24 | 0.0025707 | GPCPD1 |
| 3216_at | -9.9342 | 0.00057654 | 3.2392 | 0.0025707 | HOXB6 |
| 192670_at | -9.9338 | 0.00057663 | 3.2391 | 0.0025707 | AGO4 |
| 644192_at | 9.9323 | 0.00057697 | 3.2388 | 0.0025707 | NR2F2-AS1 |
| 9516_at | -9.925 | 0.00057861 | 3.2376 | 0.0025757 | LITAF |
| 79852_at | -9.9082 | 0.00058242 | 3.2348 | 0.0025878 | EPHX3 |
| 10608_at | 9.904 | 0.00058339 | 3.234 | 0.0025878 | MXD4 |
| 83872_at | 9.9027 | 0.00058368 | 3.2338 | 0.0025878 | HMCN1 |
| 51701_at | 9.9025 | 0.00058372 | 3.2338 | 0.0025878 | NLK |
| 83992_at | 9.9016 | 0.00058393 | 3.2336 | 0.0025878 | CTTNBP2 |
| 9577_at | 9.899 | 0.00058452 | 3.2332 | 0.0025881 | BRE |
| 80144_at | -9.8951 | 0.00058541 | 3.2325 | 0.0025892 | FRAS1 |
| 7153_at | -9.8935 | 0.00058579 | 3.2323 | 0.0025892 | TOP2A |
| 10046_at | -9.8746 | 0.00059014 | 3.229 | 0.002605 | MAMLD1 |
| 55970_at | 9.8725 | 0.00059062 | 3.2287 | 0.002605 | GNG12 |
| 6495_at | 9.8698 | 0.00059125 | 3.2282 | 0.002605 | SIX1 |
| 79858_at | 9.8689 | 0.00059146 | 3.2281 | 0.002605 | NEK11 |
| 113419_at | 9.8593 | 0.00059368 | 3.2264 | 0.0026125 | TEX261 |
| 149773_at | -9.8496 | 0.00059594 | 3.2248 | 0.0026202 | APCDD1L-AS1 |
| 729970_at | -9.846 | 0.0005968 | 3.2242 | 0.0026209 | LOC729970 |
| 10670_at | 9.8445 | 0.00059715 | 3.2239 | 0.0026209 | RRAGA |
| 11149_at | -9.8349 | 0.00059939 | 3.2223 | 0.0026275 | BVES |
| 669_at | -9.8336 | 0.00059972 | 3.2221 | 0.0026275 | BPGM |
| 266655_at | 9.8246 | 0.00060184 | 3.2205 | 0.0026345 | LINC00094 |
| 688_at | -9.8156 | 0.00060398 | 3.219 | 0.0026415 | KLF5 |
| 27315_at | 9.8076 | 0.00060588 | 3.2176 | 0.0026448 | PGAP2 |
| 406991_at | -9.8059 | 0.00060628 | 3.2173 | 0.0026448 | MIR21 |
| 118980_at | 9.8058 | 0.00060631 | 3.2173 | 0.0026448 | SFXN2 |
| 6196_at | -9.7925 | 0.00060951 | 3.215 | 0.0026525 | RPS6KA2 |
| 101929206_at | -9.7916 | 0.00060971 | 3.2149 | 0.0026525 | LOC101929206 |
| 5921_at | -9.7914 | 0.00060975 | 3.2148 | 0.0026525 | RASA1 |
| 1002_at | -9.7896 | 0.0006102 | 3.2145 | 0.0026525 | CDH4 |
| 79784_at | -9.7861 | 0.00061105 | 3.2139 | 0.0026539 | MYH14 |
| 7802_at | 9.7808 | 0.00061233 | 3.213 | 0.0026563 | DNALI1 |
| 10775_at | 9.7777 | 0.00061307 | 3.2125 | 0.0026563 | POP4 |
| 9123_at | 9.7772 | 0.00061319 | 3.2124 | 0.0026563 | SLC16A3 |
| 29842_at | -9.7689 | 0.00061521 | 3.211 | 0.0026627 | TFCP2L1 |
| 26539_at | -9.7546 | 0.00061871 | 3.2085 | 0.0026755 | OR10H1 |
| 9749_at | -9.7473 | 0.00062049 | 3.2073 | 0.0026791 | PHACTR2 |
| 9760_at | -9.7468 | 0.00062061 | 3.2072 | 0.0026791 | TOX |
| 3475_at | -9.7365 | 0.00062316 | 3.2054 | 0.0026878 | IFRD1 |
| 3988_at | 9.719 | 0.0006275 | 3.2024 | 0.0027019 | LIPA |
| 3659_at | -9.719 | 0.00062752 | 3.2024 | 0.0027019 | IRF1 |
| 84171_at | -9.7125 | 0.00062914 | 3.2013 | 0.0027066 | LOXL4 |
| 57017_at | 9.7067 | 0.00063057 | 3.2003 | 0.0027081 | COQ9 |
| 80321_at | -9.7067 | 0.00063057 | 3.2003 | 0.0027081 | CEP70 |
| 22999_at | -9.7035 | 0.00063139 | 3.1997 | 0.0027093 | RIMS1 |
| 53836_at | -9.6921 | 0.00063428 | 3.1977 | 0.0027177 | GPR87 |
| 64900_at | 9.6914 | 0.00063443 | 3.1976 | 0.0027177 | LPIN3 |
| 101928389_at | 9.6868 | 0.00063561 | 3.1968 | 0.0027181 | LOC101928389 |
| 490_at | -9.6867 | 0.00063563 | 3.1968 | 0.0027181 | ATP2B1 |
| 85439_at | 9.6821 | 0.0006368 | 3.196 | 0.0027208 | STON2 |
| 6385_at | 9.6733 | 0.00063904 | 3.1945 | 0.0027281 | SDC4 |
| 11119_at | -9.659 | 0.0006427 | 3.192 | 0.0027414 | BTN3A1 |
| 23002_at | -9.6568 | 0.00064327 | 3.1916 | 0.0027414 | DAAM1 |
| 25805_at | 9.6365 | 0.00064853 | 3.1881 | 0.0027615 | BAMBI |
| 100506498_at | 9.6329 | 0.00064944 | 3.1875 | 0.0027628 | LOC100506498 |
| 6284_at | 9.631 | 0.00064994 | 3.1871 | 0.0027628 | S100A13 |
| 84665_at | -9.6214 | 0.00065246 | 3.1854 | 0.0027712 | MYPN |
| 55205_at | -9.6088 | 0.00065576 | 3.1833 | 0.0027828 | ZNF532 |
| 3899_at | 9.6052 | 0.00065672 | 3.1826 | 0.0027831 | AFF3 |
| 22982_at | 9.6043 | 0.00065695 | 3.1825 | 0.0027831 | DIP2C |
| 3777_at | 9.5936 | 0.0006598 | 3.1806 | 0.0027928 | KCNK3 |
| 56255_at | 9.5839 | 0.00066236 | 3.1789 | 0.0027983 | TMX4 |
| 131544_at | 9.5806 | 0.00066325 | 3.1783 | 0.0027983 | CRYBG3 |
| 8273_at | 9.5805 | 0.00066328 | 3.1783 | 0.0027983 | SLC10A3 |
| 23157_at | -9.5796 | 0.00066353 | 3.1781 | 0.0027983 | 6-Sep |
| 83959_at | -9.5782 | 0.00066389 | 3.1779 | 0.0027983 | SLC4A11 |
| 6271_at | 9.5733 | 0.00066521 | 3.177 | 0.0028015 | S100A1 |
| 374395_at | 9.5654 | 0.00066733 | 3.1757 | 0.0028035 | TMEM179B |
| 7088_at | 9.5652 | 0.00066737 | 3.1756 | 0.0028035 | TLE1 |
| 51573_at | -9.5652 | 0.00066738 | 3.1756 | 0.0028035 | GDE1 |
| 1102_at | 9.553 | 0.00067066 | 3.1735 | 0.002815 | RCBTB2 |
| 2908_at | 9.538 | 0.00067475 | 3.1709 | 0.0028295 | NR3C1 |
| 9075_at | 9.5362 | 0.00067525 | 3.1705 | 0.0028295 | CLDN2 |
| 5243_at | 9.5278 | 0.00067756 | 3.1691 | 0.0028304 | ABCB1 |
| 5286_at | -9.5277 | 0.00067757 | 3.169 | 0.0028304 | PIK3C2A |
| 50618_at | -9.5268 | 0.00067783 | 3.1689 | 0.0028304 | ITSN2 |
| 23639_at | 9.5257 | 0.00067812 | 3.1687 | 0.0028304 | LRRC6 |
| 57820_at | -9.5251 | 0.0006783 | 3.1686 | 0.0028304 | CCNB1IP1 |
| 6920_at | 9.5179 | 0.00068026 | 3.1673 | 0.0028362 | TCEA3 |
| 64393_at | -9.5099 | 0.0006825 | 3.1659 | 0.0028382 | ZMAT3 |
| 9990_at | -9.5096 | 0.00068257 | 3.1659 | 0.0028382 | SLC12A6 |
| 10424_at | 9.5083 | 0.00068294 | 3.1656 | 0.0028382 | PGRMC2 |
| 283349_at | -9.5076 | 0.00068313 | 3.1655 | 0.0028382 | RASSF3 |
| 91_at | 9.5038 | 0.00068417 | 3.1648 | 0.0028382 | ACVR1B |
| 116328_at | -9.5001 | 0.0006852 | 3.1642 | 0.0028382 | C8orf34 |
| 252983_at | 9.5 | 0.00068522 | 3.1642 | 0.0028382 | STXBP4 |
| 79183_at | 9.4997 | 0.00068531 | 3.1641 | 0.0028382 | TTPAL |
| 710_at | 9.4978 | 0.00068584 | 3.1638 | 0.0028382 | SERPING1 |
| 23637_at | -9.4898 | 0.00068807 | 3.1624 | 0.0028442 | RABGAP1 |
| 133688_at | 9.4885 | 0.00068845 | 3.1621 | 0.0028442 | UGT3A1 |
| 2250_at | -9.4613 | 0.00069611 | 3.1573 | 0.0028735 | FGF5 |
| 401548_at | 9.4541 | 0.00069815 | 3.1561 | 0.0028796 | SNX30 |
| 25939_at | -9.451 | 0.00069904 | 3.1555 | 0.0028809 | SAMHD1 |
| 5493_at | -9.4467 | 0.00070027 | 3.1547 | 0.0028836 | PPL |
| 488_at | -9.4428 | 0.00070137 | 3.1541 | 0.0028857 | ATP2A2 |
| 100130776_at | 9.4349 | 0.00070364 | 3.1526 | 0.0028912 | AGAP2-AS1 |
| 114659_at | -9.4342 | 0.00070385 | 3.1525 | 0.0028912 | LRRC37B |
| 152110_at | 9.425 | 0.00070651 | 3.1509 | 0.0028974 | NEK10 |
| 10103_at | -9.4249 | 0.00070653 | 3.1509 | 0.0028974 | TSPAN1 |
| 285590_at | -9.4222 | 0.00070733 | 3.1504 | 0.0028983 | SH3PXD2B |
| 8825_at | 9.4181 | 0.0007085 | 3.1497 | 0.0029007 | LIN7A |
| 152579_at | 9.409 | 0.00071116 | 3.148 | 0.0029092 | SCFD2 |
| 80763_at | 9.3998 | 0.00071382 | 3.1464 | 0.0029177 | SPX |
| 26958_at | -9.3952 | 0.00071517 | 3.1456 | 0.00292 | COPG2 |
| 81565_at | -9.3934 | 0.00071571 | 3.1453 | 0.00292 | NDEL1 |
| 375449_at | -9.392 | 0.00071612 | 3.145 | 0.00292 | MAST4 |
| 254427_at | -9.3877 | 0.00071739 | 3.1442 | 0.0029228 | PROSER2 |
| 6674_at | -9.3803 | 0.00071959 | 3.1429 | 0.002925 | SPAG1 |
| 22898_at | -9.38 | 0.00071965 | 3.1429 | 0.002925 | DENND3 |
| 8761_at | 9.3779 | 0.00072029 | 3.1425 | 0.002925 | PABPC4 |
| 10082_at | 9.3779 | 0.00072029 | 3.1425 | 0.002925 | GPC6 |
| 7106_at | 9.3749 | 0.00072117 | 3.142 | 0.0029263 | TSPAN4 |
| 2014_at | 9.3705 | 0.00072249 | 3.1412 | 0.0029292 | EMP3 |
| 51088_at | -9.3679 | 0.00072326 | 3.1407 | 0.0029299 | KLHL5 |
| 10440_at | 9.3626 | 0.00072484 | 3.1398 | 0.002934 | TIMM17A |
| 113791_at | -9.3535 | 0.00072756 | 3.1381 | 0.0029426 | PIK3IP1 |
| 3486_at | 9.3507 | 0.00072839 | 3.1376 | 0.0029426 | IGFBP3 |
| 9444_at | -9.3476 | 0.00072933 | 3.1371 | 0.0029426 | QKI |
| 10042_at | -9.3475 | 0.00072936 | 3.1371 | 0.0029426 | HMGXB4 |
| 84101_at | -9.3456 | 0.00072992 | 3.1367 | 0.0029426 | USP44 |
| 56181_at | 9.3383 | 0.00073213 | 3.1354 | 0.0029487 | MTFR1L |
| 80209_at | -9.3367 | 0.0007326 | 3.1351 | 0.0029487 | PROSER1 |
| 56256_at | -9.3264 | 0.00073574 | 3.1333 | 0.0029552 | SERTAD4 |
| 2762_at | -9.3261 | 0.00073582 | 3.1332 | 0.0029552 | GMDS |
| 25897_at | -9.3249 | 0.00073618 | 3.133 | 0.0029552 | RNF19A |
| 285440_at | 9.3204 | 0.00073756 | 3.1322 | 0.0029552 | CYP4V2 |
| 56474_at | -9.3201 | 0.00073766 | 3.1321 | 0.0029552 | CTPS2 |
| 144132_at | -9.3196 | 0.00073779 | 3.1321 | 0.0029552 | DNHD1 |
| 150696_at | -9.3178 | 0.00073837 | 3.1317 | 0.0029552 | PROM2 |
| 399474_at | 9.3131 | 0.00073981 | 3.1309 | 0.0029586 | TMEM200B |
| 2525_at | -9.3057 | 0.00074207 | 3.1296 | 0.0029653 | FUT3 |
| 80157_at | -9.2991 | 0.00074411 | 3.1284 | 0.0029665 | CWH43 |
| 7035_at | 9.2985 | 0.00074428 | 3.1283 | 0.0029665 | TFPI |
| 25963_at | -9.297 | 0.00074474 | 3.128 | 0.0029665 | TMEM87A |
| 55088_at | -9.297 | 0.00074475 | 3.128 | 0.0029665 | CCDC186 |
| 64598_at | 9.2931 | 0.00074595 | 3.1273 | 0.002969 | MOSPD3 |
| 7343_at | 9.2887 | 0.00074729 | 3.1265 | 0.0029719 | UBTF |
| 219285_at | -9.2821 | 0.00074935 | 3.1253 | 0.0029775 | SAMD9L |
| 6513_at | 9.2804 | 0.00074988 | 3.125 | 0.0029775 | SLC2A1 |
| 221037_at | -9.2744 | 0.00075174 | 3.1239 | 0.0029818 | JMJD1C |
| 51667_at | -9.2731 | 0.00075217 | 3.1237 | 0.0029818 | NUB1 |
| 24147_at | 9.2703 | 0.00075302 | 3.1232 | 0.0029829 | FJX1 |
| 79867_at | 9.2672 | 0.00075402 | 3.1226 | 0.0029844 | TCTN2 |
| 84193_at | 9.2635 | 0.00075515 | 3.122 | 0.0029866 | SETD3 |
| 2651_at | -9.2589 | 0.0007566 | 3.1211 | 0.0029877 | GCNT2 |
| 79971_at | -9.2588 | 0.00075664 | 3.1211 | 0.0029877 | WLS |
| 6641_at | 9.2373 | 0.00076346 | 3.1172 | 0.0030107 | SNTB1 |
| 168667_at | 9.2366 | 0.00076368 | 3.1171 | 0.0030107 | BMPER |
| 91543_at | -9.2338 | 0.00076457 | 3.1166 | 0.0030119 | RSAD2 |
| 9185_at | 9.2232 | 0.00076797 | 3.1147 | 0.0030224 | REPS2 |
| 100506190_at | 9.2217 | 0.00076845 | 3.1144 | 0.0030224 | LINC00963 |
| 78987_at | -9.2138 | 0.00077096 | 3.113 | 0.003029 | CRELD1 |
| 102724630_at | -9.2126 | 0.00077135 | 3.1127 | 0.003029 | LOC102724630 |
| 53405_at | -9.2095 | 0.00077236 | 3.1122 | 0.0030307 | CLIC5 |
| 152926_at | -9.2049 | 0.00077383 | 3.1114 | 0.003034 | PPM1K |
| 254887_at | 9.2003 | 0.00077534 | 3.1105 | 0.0030358 | ZDHHC23 |
| 6446_at | 9.1998 | 0.00077549 | 3.1104 | 0.0030358 | SGK1 |
| 79819_at | 9.1744 | 0.0007838 | 3.1058 | 0.0030659 | WDR78 |
| 26011_at | 9.1568 | 0.0007896 | 3.1026 | 0.0030862 | TENM4 |
| 25885_at | 9.155 | 0.00079021 | 3.1023 | 0.0030862 | POLR1A |
| 9871_at | -9.1508 | 0.00079161 | 3.1015 | 0.0030883 | SEC24D |
| 9846_at | -9.1496 | 0.000792 | 3.1013 | 0.0030883 | GAB2 |
| 645455_at | -9.144 | 0.00079388 | 3.1002 | 0.0030932 | CEP170P1 |
| 7348_at | 9.1399 | 0.00079524 | 3.0995 | 0.003094 | UPK1B |
| 8321_at | 9.1396 | 0.00079533 | 3.0995 | 0.003094 | FZD1 |
| 55619_at | -9.1302 | 0.00079848 | 3.0977 | 0.0031039 | DOCK10 |
| 9673_at | 9.1178 | 0.00080269 | 3.0955 | 0.0031178 | SLC25A44 |
| 4495_at | 9.1062 | 0.00080663 | 3.0933 | 0.0031307 | MT1G |
| 147081_at | -9.0883 | 0.00081275 | 3.09 | 0.0031519 | CRHR1-IT1 |
| 55137_at | 9.0853 | 0.00081378 | 3.0895 | 0.0031519 | FIGN |
| 23225_at | -9.0847 | 0.00081399 | 3.0894 | 0.0031519 | NUP210 |
| 80830_at | -9.0664 | 0.00082031 | 3.086 | 0.0031739 | APOL6 |
| 57534_at | -9.0524 | 0.00082521 | 3.0834 | 0.0031904 | MIB1 |
| 139065_at | -9.0498 | 0.00082612 | 3.083 | 0.0031912 | SLITRK4 |
| 55222_at | -9.0482 | 0.00082668 | 3.0827 | 0.0031912 | LRRC20 |
| 55117_at | -9.0301 | 0.00083308 | 3.0793 | 0.003211 | SLC6A15 |
| 9590_at | -9.0295 | 0.00083327 | 3.0792 | 0.003211 | AKAP12 |
| 1268_at | 9.0275 | 0.00083401 | 3.0788 | 0.003211 | CNR1 |
| 152273_at | 9.0264 | 0.00083438 | 3.0786 | 0.003211 | FGD5 |
| 122402_at | 9.0232 | 0.00083554 | 3.078 | 0.003213 | TDRD9 |
| 6734_at | -9.0211 | 0.00083629 | 3.0776 | 0.0032134 | SRPR |
| 947_at | -9.0187 | 0.00083712 | 3.0772 | 0.0032141 | CD34 |
| 5329_at | -9.0114 | 0.00083973 | 3.0759 | 0.0032206 | PLAUR |
| 79573_at | -9.01 | 0.00084024 | 3.0756 | 0.0032206 | TTC13 |
| 9743_at | -9.0086 | 0.00084075 | 3.0753 | 0.0032206 | ARHGAP32 |
| 374918_at | -9.0064 | 0.00084154 | 3.0749 | 0.0032212 | IGFL1 |
| 117153_at | 9.0003 | 0.00084372 | 3.0738 | 0.0032271 | MIA2 |
| 6536_at | -8.9941 | 0.00084595 | 3.0727 | 0.0032289 | SLC6A9 |
| 55277_at | 8.9931 | 0.00084631 | 3.0725 | 0.0032289 | FGGY |
| 196740_at | 8.991 | 0.00084707 | 3.0721 | 0.0032289 | VSTM4 |
| 145567_at | -8.9905 | 0.00084728 | 3.072 | 0.0032289 | TTC7B |
| 222643_at | 8.9884 | 0.00084804 | 3.0716 | 0.0032289 | UNC5CL |
| 7486_at | -8.9883 | 0.00084807 | 3.0716 | 0.0032289 | WRN |
| 57688_at | -8.9784 | 0.00085165 | 3.0697 | 0.0032373 | ZSWIM6 |
| 5230_at | 8.9784 | 0.00085167 | 3.0697 | 0.0032373 | PGK1 |
| 84159_at | 8.9768 | 0.00085224 | 3.0694 | 0.0032373 | ARID5B |
| 127544_at | -8.9722 | 0.00085391 | 3.0686 | 0.0032412 | RNF19B |
| 23532_at | 8.9535 | 0.00086078 | 3.0651 | 0.0032648 | PRAME |
| 283345_at | 8.9493 | 0.00086236 | 3.0643 | 0.0032669 | RPL13P5 |
| 11226_at | -8.9461 | 0.00086352 | 3.0637 | 0.0032669 | GALNT6 |
| 781_at | 8.9452 | 0.00086387 | 3.0636 | 0.0032669 | CACNA2D1 |
| 8504_at | 8.944 | 0.00086431 | 3.0633 | 0.0032669 | PEX3 |
| 147166_at | -8.9432 | 0.0008646 | 3.0632 | 0.0032669 | TRIM16L |
| 23235_at | 8.939 | 0.00086618 | 3.0624 | 0.0032701 | SIK2 |
| 729987_at | -8.935 | 0.00086768 | 3.0616 | 0.0032701 | LOC729987 |
| 147463_at | 8.934 | 0.00086802 | 3.0615 | 0.0032701 | ANKRD29 |
| 2937_at | 8.9339 | 0.00086806 | 3.0614 | 0.0032701 | GSS |
| 5210_at | 8.9306 | 0.00086931 | 3.0608 | 0.0032705 | PFKFB4 |
| 10687_at | 8.9301 | 0.00086948 | 3.0607 | 0.0032705 | PNMA2 |
| 56243_at | -8.9283 | 0.00087017 | 3.0604 | 0.0032707 | KIAA1217 |
| 201191_at | 8.9214 | 0.00087274 | 3.0591 | 0.0032778 | SAMD14 |
| 4758_at | 8.919 | 0.00087366 | 3.0587 | 0.0032788 | NEU1 |
| 6692_at | -8.9037 | 0.00087943 | 3.0558 | 0.003298 | SPINT1 |
| 7057_at | 8.8927 | 0.0008836 | 3.0537 | 0.0033107 | THBS1 |
| 64061_at | -8.8875 | 0.0008856 | 3.0528 | 0.0033107 | TSPYL2 |
| 8076_at | 8.8862 | 0.00088608 | 3.0525 | 0.0033107 | MFAP5 |
| 121601_at | 8.8854 | 0.0008864 | 3.0524 | 0.0033107 | ANO4 |
| 860_at | 8.8852 | 0.0008865 | 3.0523 | 0.0033107 | RUNX2 |
| 10000_at | -8.8844 | 0.00088677 | 3.0522 | 0.0033107 | AKT3 |
| 7045_at | 8.866 | 0.00089386 | 3.0487 | 0.003331 | TGFBI |
| 323_at | -8.8654 | 0.00089411 | 3.0486 | 0.003331 | APBB2 |
| 54682_at | -8.8651 | 0.00089423 | 3.0485 | 0.003331 | MANSC1 |
| 79850_at | 8.8539 | 0.00089856 | 3.0465 | 0.0033447 | FAM57A |
| 84529_at | 8.852 | 0.00089933 | 3.0461 | 0.003345 | C15orf41 |
| 9588_at | 8.8389 | 0.00090444 | 3.0436 | 0.0033606 | PRDX6 |
| 79134_at | 8.8355 | 0.00090576 | 3.043 | 0.0033606 | TMEM185B |
| 283208_at | 8.8353 | 0.00090586 | 3.0429 | 0.0033606 | P4HA3 |
| 4179_at | -8.8328 | 0.00090684 | 3.0425 | 0.0033606 | CD46 |
| 147710_at | 8.8327 | 0.00090687 | 3.0425 | 0.0033606 | IGSF23 |
| 79746_at | 8.8295 | 0.00090812 | 3.0419 | 0.0033606 | ECHDC3 |
| 8399_at | -8.8293 | 0.00090822 | 3.0418 | 0.0033606 | PLA2G10 |
| 1014_at | 8.8233 | 0.00091061 | 3.0407 | 0.003367 | CDH16 |
| 254531_at | -8.8064 | 0.00091732 | 3.0375 | 0.0033861 | LPCAT4 |
| 2886_at | -8.8048 | 0.00091797 | 3.0372 | 0.0033861 | GRB7 |
| 23015_at | 8.8038 | 0.00091837 | 3.037 | 0.0033861 | GOLGA8A |
| 3613_at | 8.8006 | 0.00091964 | 3.0364 | 0.0033861 | IMPA2 |
| 516_at | 8.7999 | 0.00091992 | 3.0363 | 0.0033861 | ATP5G1 |
| 199857_at | 8.7986 | 0.00092043 | 3.036 | 0.0033861 | ALG14 |
| 8565_at | -8.7984 | 0.00092052 | 3.036 | 0.0033861 | YARS |
| 54583_at | 8.7926 | 0.00092285 | 3.0349 | 0.0033915 | EGLN1 |
| 389015_at | -8.7913 | 0.00092336 | 3.0346 | 0.0033915 | SLC9A4 |
| 2537_at | -8.7878 | 0.00092479 | 3.034 | 0.0033943 | IFI6 |
| 5420_at | -8.7835 | 0.00092651 | 3.0332 | 0.0033981 | PODXL |
| 729920_at | 8.7815 | 0.00092732 | 3.0328 | 0.0033986 | ISPD |
| 287_at | 8.7794 | 0.00092818 | 3.0324 | 0.0033993 | ANK2 |
| 55349_at | 8.7728 | 0.00093088 | 3.0311 | 0.0034066 | CHDH |
| 2707_at | -8.771 | 0.0009316 | 3.0308 | 0.0034068 | GJB3 |
| 5939_at | -8.7639 | 0.00093448 | 3.0294 | 0.0034148 | RBMS2 |
| 23516_at | 8.7583 | 0.00093677 | 3.0284 | 0.0034207 | SLC39A14 |
| 729013_at | 8.7479 | 0.00094107 | 3.0264 | 0.0034339 | ZBED5-AS1 |
| 10631_at | 8.7438 | 0.00094277 | 3.0256 | 0.0034376 | POSTN |
| 55771_at | -8.7321 | 0.0009476 | 3.0234 | 0.0034527 | PRR11 |
| 100129034_at | 8.7237 | 0.00095113 | 3.0218 | 0.0034616 | LOC100129034 |
| 535_at | 8.7191 | 0.00095301 | 3.0209 | 0.0034616 | ATP6V0A1 |
| 101926931_at | 8.7187 | 0.00095319 | 3.0208 | 0.0034616 | LOC101926931 |
| 116496_at | 8.7186 | 0.00095323 | 3.0208 | 0.0034616 | FAM129A |
| 11240_at | 8.7174 | 0.00095376 | 3.0206 | 0.0034616 | PADI2 |
| 23659_at | 8.7163 | 0.00095421 | 3.0204 | 0.0034616 | PLA2G15 |
| 28996_at | 8.7063 | 0.0009584 | 3.0185 | 0.0034743 | HIPK2 |
| 8482_at | -8.7021 | 0.0009602 | 3.0176 | 0.0034781 | SEMA7A |
| 2289_at | 8.7006 | 0.00096083 | 3.0174 | 0.0034781 | FKBP5 |
| 2921_at | -8.6939 | 0.00096368 | 3.0161 | 0.0034858 | CXCL3 |
| 11274_at | -8.6872 | 0.0009665 | 3.0148 | 0.0034935 | USP18 |
| 144455_at | -8.6848 | 0.00096755 | 3.0143 | 0.0034948 | E2F7 |
| 10954_at | 8.6831 | 0.00096828 | 3.014 | 0.0034949 | PDIA5 |
| 8925_at | -8.6809 | 0.00096921 | 3.0136 | 0.0034957 | HERC1 |
| 57718_at | -8.676 | 0.0009713 | 3.0126 | 0.0034999 | PPP4R4 |
| 56898_at | 8.6748 | 0.00097183 | 3.0124 | 0.0034999 | BDH2 |
| 10474_at | 8.6733 | 0.00097248 | 3.0121 | 0.0034999 | TADA3 |
| 6917_at | -8.6675 | 0.00097496 | 3.011 | 0.0035063 | TCEA1 |
| 257397_at | -8.649 | 0.00098297 | 3.0075 | 0.0035326 | TAB3 |
| 10326_at | 8.6444 | 0.00098499 | 3.0066 | 0.0035373 | SIRPB1 |
| 220963_at | 8.6426 | 0.00098577 | 3.0062 | 0.0035375 | SLC16A9 |
| 221883_at | -8.641 | 0.00098645 | 3.0059 | 0.0035375 | HOXA11-AS |
| 100507002_at | 8.6352 | 0.00098901 | 3.0048 | 0.0035441 | LOC100507002 |
| 7804_at | -8.6224 | 0.00099466 | 3.0023 | 0.0035571 | LRP8 |
| 340120_at | -8.6223 | 0.00099467 | 3.0023 | 0.0035571 | ANKRD34B |
| 80313_at | 8.622 | 0.00099484 | 3.0022 | 0.0035571 | LRRC27 |
| 83988_at | -8.6205 | 0.00099548 | 3.002 | 0.0035571 | NCALD |
| 85462_at | -8.6156 | 0.00099763 | 3.001 | 0.00356 | FHDC1 |
| 586_at | -8.6154 | 0.00099773 | 3.001 | 0.00356 | BCAT1 |
| 2634_at | -8.6075 | 0.0010012 | 2.9995 | 0.00357 | GBP2 |
| 28_at | 8.6035 | 0.001003 | 2.9987 | 0.0035733 | ABO |
| 3920_at | 8.5992 | 0.0010049 | 2.9979 | 0.0035733 | LAMP2 |
| 8641_at | 8.599 | 0.001005 | 2.9978 | 0.0035733 | PCDHGB4 |
| 780_at | -8.597 | 0.0010059 | 2.9974 | 0.0035733 | DDR1 |
| 340485_at | -8.5962 | 0.0010063 | 2.9973 | 0.0035733 | ACER2 |
| 8612_at | -8.5959 | 0.0010064 | 2.9972 | 0.0035733 | PPAP2C |
| 2009_at | 8.5925 | 0.0010079 | 2.9966 | 0.0035761 | EML1 |
| 79567_at | 8.5811 | 0.0010131 | 2.9944 | 0.0035918 | FAM65A |
| 57189_at | -8.5777 | 0.0010146 | 2.9937 | 0.0035919 | KIAA1147 |
| 406896_at | -8.5775 | 0.0010147 | 2.9937 | 0.0035919 | MIR103A2 |
| 1612_at | 8.5763 | 0.0010153 | 2.9934 | 0.0035919 | DAPK1 |
| 439990_at | -8.5563 | 0.0010244 | 2.9895 | 0.00362 | LINC00857 |
| 84750_at | -8.5542 | 0.0010253 | 2.9891 | 0.00362 | FUT10 |
| 113263_at | -8.5541 | 0.0010254 | 2.9891 | 0.00362 | GLCCI1 |
| 51363_at | -8.5514 | 0.0010266 | 2.9886 | 0.0036218 | CHST15 |
| 3096_at | -8.5473 | 0.0010285 | 2.9878 | 0.0036258 | HIVEP1 |
| 79098_at | -8.5401 | 0.0010318 | 2.9864 | 0.0036326 | C1orf116 |
| 159013_at | -8.54 | 0.0010319 | 2.9864 | 0.0036326 | CXorf38 |
| 112464_at | 8.5335 | 0.0010349 | 2.9851 | 0.0036382 | PRKCDBP |
| 57537_at | 8.5319 | 0.0010356 | 2.9848 | 0.0036382 | SORCS2 |
| 9229_at | 8.5319 | 0.0010356 | 2.9848 | 0.0036382 | DLGAP1 |
| 375035_at | 8.5279 | 0.0010375 | 2.984 | 0.0036415 | SFT2D2 |
| 8660_at | -8.5267 | 0.001038 | 2.9838 | 0.0036415 | IRS2 |
| 2300_at | -8.5215 | 0.0010405 | 2.9828 | 0.0036475 | FOXL1 |
| 148979_at | 8.5194 | 0.0010415 | 2.9824 | 0.0036484 | GLIS1 |
| 6405_at | -8.5115 | 0.0010452 | 2.9808 | 0.003655 | SEMA3F |
| 10437_at | -8.51 | 0.0010458 | 2.9805 | 0.003655 | IFI30 |
| 114625_at | 8.5088 | 0.0010464 | 2.9803 | 0.003655 | ERMAP |
| 79605_at | -8.5082 | 0.0010467 | 2.9802 | 0.003655 | PGBD5 |
| 79411_at | 8.5053 | 0.0010481 | 2.9796 | 0.003655 | GLB1L |
| 57478_at | -8.5047 | 0.0010484 | 2.9795 | 0.003655 | USP31 |
| 27032_at | -8.5034 | 0.001049 | 2.9792 | 0.003655 | ATP2C1 |
| 22871_at | 8.5028 | 0.0010492 | 2.9791 | 0.003655 | NLGN1 |
| 871_at | 8.5014 | 0.0010499 | 2.9788 | 0.003655 | SERPINH1 |
| 5891_at | -8.4995 | 0.0010508 | 2.9785 | 0.0036556 | MOK |
| 55824_at | 8.4964 | 0.0010523 | 2.9779 | 0.003658 | PAG1 |
| 159371_at | 8.4918 | 0.0010545 | 2.977 | 0.0036631 | SLC35G1 |
| 79776_at | 8.4784 | 0.0010609 | 2.9743 | 0.0036828 | ZFHX4 |
| 166336_at | 8.4735 | 0.0010632 | 2.9734 | 0.0036868 | PRICKLE2 |
| 100302179_at | 8.4729 | 0.0010635 | 2.9733 | 0.0036868 | MIR1270 |
| 5266_at | -8.4693 | 0.0010652 | 2.9726 | 0.0036902 | PI3 |
| 9627_at | -8.4667 | 0.0010665 | 2.9721 | 0.003692 | SNCAIP |
| 397_at | -8.4634 | 0.0010681 | 2.9714 | 0.003695 | ARHGDIB |
| 2571_at | -8.4562 | 0.0010715 | 2.97 | 0.003703 | GAD1 |
| 55288_at | -8.4555 | 0.0010719 | 2.9699 | 0.003703 | RHOT1 |
| 9901_at | -8.4516 | 0.0010738 | 2.9691 | 0.0037049 | SRGAP3 |
| 6776_at | -8.4513 | 0.0010739 | 2.969 | 0.0037049 | STAT5A |
| 114818_at | 8.4498 | 0.0010746 | 2.9687 | 0.0037049 | KLHL29 |
| 80853_at | -8.4463 | 0.0010764 | 2.968 | 0.0037068 | KDM7A |
| 91252_at | 8.445 | 0.001077 | 2.9678 | 0.0037068 | SLC39A13 |
| 5176_at | 8.4441 | 0.0010774 | 2.9676 | 0.0037068 | SERPINF1 |
| 5076_at | 8.4397 | 0.0010796 | 2.9667 | 0.0037118 | PAX2 |
| 51491_at | 8.429 | 0.0010848 | 2.9646 | 0.0037272 | NOP16 |
| 7538_at | 8.4071 | 0.0010957 | 2.9603 | 0.0037618 | ZFP36 |
| 567_at | -8.4034 | 0.0010975 | 2.9596 | 0.0037644 | B2M |
| 6843_at | 8.402 | 0.0010982 | 2.9593 | 0.0037644 | VAMP1 |
| 7702_at | -8.401 | 0.0010987 | 2.9591 | 0.0037644 | ZNF143 |
| 8648_at | 8.3995 | 0.0010994 | 2.9588 | 0.0037644 | NCOA1 |
| 677847_at | -8.3802 | 0.0011092 | 2.955 | 0.0037935 | SNORA81 |
| 22930_at | -8.3795 | 0.0011095 | 2.9549 | 0.0037935 | RAB3GAP1 |
| 389376_at | -8.3775 | 0.0011105 | 2.9545 | 0.0037935 | SFTA2 |
| 79001_at | 8.3766 | 0.001111 | 2.9543 | 0.0037935 | VKORC1 |
| 65056_at | -8.3729 | 0.0011129 | 2.9536 | 0.0037974 | GPBP1 |
| 23243_at | -8.3692 | 0.0011148 | 2.9528 | 0.0037994 | ANKRD28 |
| 1847_at | -8.3687 | 0.001115 | 2.9527 | 0.0037994 | DUSP5 |
| 1571_at | -8.3667 | 0.001116 | 2.9523 | 0.0038003 | CYP2E1 |
| 100271927_at | -8.3591 | 0.0011199 | 2.9508 | 0.0038109 | RASA4B |
| 10516_at | 8.3535 | 0.0011228 | 2.9497 | 0.0038182 | FBLN5 |
| 7464_at | -8.3519 | 0.0011236 | 2.9494 | 0.0038183 | CORO2A |
| 54579_at | 8.3408 | 0.0011293 | 2.9472 | 0.0038352 | UGT1A5 |
| 9966_at | -8.3353 | 0.0011321 | 2.9461 | 0.0038422 | TNFSF15 |
| 1829_at | -8.3281 | 0.0011359 | 2.9447 | 0.0038523 | DSG2 |
| 91937_at | 8.321 | 0.0011396 | 2.9432 | 0.0038623 | TIMD4 |
| 5366_at | -8.3125 | 0.001144 | 2.9416 | 0.0038747 | PMAIP1 |
| 23245_at | 8.3101 | 0.0011453 | 2.9411 | 0.0038764 | ASTN2 |
| 57211_at | -8.306 | 0.0011475 | 2.9403 | 0.003881 | GPR126 |
| 29841_at | -8.2968 | 0.0011523 | 2.9384 | 0.0038948 | GRHL1 |
| 3204_at | 8.2885 | 0.0011567 | 2.9368 | 0.0039071 | HOXA7 |
| 401474_at | -8.2858 | 0.0011582 | 2.9362 | 0.0039093 | SAMD12 |
| 3433_at | -8.2797 | 0.0011614 | 2.935 | 0.0039177 | IFIT2 |
| 133418_at | -8.2712 | 0.001166 | 2.9333 | 0.0039284 | EMB |
| 90639_at | -8.2708 | 0.0011662 | 2.9332 | 0.0039284 | COX19 |
| 92579_at | 8.2661 | 0.0011687 | 2.9323 | 0.0039343 | G6PC3 |
| 2151_at | -8.2642 | 0.0011697 | 2.9319 | 0.0039351 | F2RL2 |
| 7508_at | 8.26 | 0.001172 | 2.9311 | 0.0039402 | XPC |
| 6383_at | -8.2575 | 0.0011734 | 2.9306 | 0.0039421 | SDC2 |
| 3885_at | -8.2521 | 0.0011764 | 2.9295 | 0.0039494 | KRT34 |
| 440603_at | -8.2506 | 0.0011772 | 2.9292 | 0.0039494 | BCL2L15 |
| 6840_at | -8.2359 | 0.0011852 | 2.9262 | 0.0039725 | SVIL |
| 494115_at | -8.2334 | 0.0011866 | 2.9257 | 0.0039725 | RBMXL1 |
| 7127_at | -8.231 | 0.0011879 | 2.9252 | 0.0039725 | TNFAIP2 |
| 23597_at | -8.231 | 0.0011879 | 2.9252 | 0.0039725 | ACOT9 |
| 3231_at | 8.2308 | 0.001188 | 2.9252 | 0.0039725 | HOXD1 |
| 11067_at | -8.2167 | 0.0011958 | 2.9224 | 0.0039942 | C10orf10 |
| 10536_at | -8.2161 | 0.0011961 | 2.9222 | 0.0039942 | LEPREL2 |
| 3306_at | -8.2143 | 0.0011971 | 2.9219 | 0.0039943 | HSPA2 |
| 5290_at | -8.2112 | 0.0011988 | 2.9213 | 0.0039943 | PIK3CA |
| 157769_at | -8.2064 | 0.0012015 | 2.9203 | 0.0039943 | FAM91A1 |
| 254065_at | -8.2061 | 0.0012017 | 2.9202 | 0.0039943 | BRWD3 |
| 1718_at | 8.2055 | 0.001202 | 2.9201 | 0.0039943 | DHCR24 |
| 79642_at | 8.2049 | 0.0012024 | 2.92 | 0.0039943 | ARSJ |
| 29121_at | -8.2046 | 0.0012025 | 2.9199 | 0.0039943 | CLEC2D |
| 6868_at | -8.2033 | 0.0012032 | 2.9197 | 0.0039943 | ADAM17 |
| 65983_at | -8.2031 | 0.0012033 | 2.9196 | 0.0039943 | GRAMD3 |
| 140809_at | 8.1915 | 0.0012098 | 2.9173 | 0.0040133 | SRXN1 |
| 167227_at | -8.1875 | 0.0012121 | 2.9165 | 0.0040182 | DCP2 |
| 79962_at | 8.1827 | 0.0012148 | 2.9155 | 0.0040244 | DNAJC22 |
| 5090_at | 8.1724 | 0.0012207 | 2.9134 | 0.0040411 | PBX3 |
| 1955_at | -8.1677 | 0.0012234 | 2.9124 | 0.0040474 | MEGF9 |
| 643401_at | -8.1659 | 0.0012244 | 2.9121 | 0.0040481 | LINC01021 |
| 814_at | -8.1645 | 0.0012252 | 2.9118 | 0.0040481 | CAMK4 |
| 100505576_at | -8.1536 | 0.0012314 | 2.9096 | 0.0040659 | LINC00672 |
| 91227_at | 8.1466 | 0.0012355 | 2.9082 | 0.0040767 | GGTLC2 |
| 1543_at | -8.1351 | 0.0012422 | 2.9058 | 0.00409 | CYP1A1 |
| 29094_at | -8.1347 | 0.0012424 | 2.9057 | 0.00409 | LGALSL |
| 57209_at | 8.1337 | 0.0012429 | 2.9055 | 0.00409 | ZNF248 |
| 4141_at | -8.1331 | 0.0012433 | 2.9054 | 0.00409 | MARS |
| 3655_at | -8.1326 | 0.0012436 | 2.9053 | 0.00409 | ITGA6 |
| 9781_at | -8.1273 | 0.0012467 | 2.9042 | 0.0040975 | RNF144A |
| 84947_at | -8.122 | 0.0012498 | 2.9032 | 0.0041047 | SERAC1 |
| 54839_at | -8.1204 | 0.0012507 | 2.9028 | 0.0041047 | LRRC49 |
| 51816_at | 8.1193 | 0.0012514 | 2.9026 | 0.0041047 | CECR1 |
| 27346_at | -8.1106 | 0.0012565 | 2.9008 | 0.0041189 | TMEM97 |
| 153684_at | 8.1058 | 0.0012593 | 2.8999 | 0.0041254 | LOC153684 |
| 286343_at | -8.1024 | 0.0012613 | 2.8992 | 0.004127 | LURAP1L |
| 2059_at | 8.1022 | 0.0012615 | 2.8991 | 0.004127 | EPS8 |
| 1346_at | 8.0989 | 0.0012634 | 2.8984 | 0.0041307 | COX7A1 |
| 1647_at | -8.0932 | 0.0012668 | 2.8973 | 0.004139 | GADD45A |
| 399715_at | -8.0915 | 0.0012678 | 2.8969 | 0.0041397 | LOC399715 |
| 9308_at | -8.0876 | 0.0012702 | 2.8961 | 0.0041446 | CD83 |
| 4584_at | 8.0816 | 0.0012737 | 2.8949 | 0.0041535 | MUC3A |
| 79961_at | -8.0773 | 0.0012764 | 2.894 | 0.0041587 | DENND2D |
| 85415_at | 8.0762 | 0.001277 | 2.8938 | 0.0041587 | RHPN2 |
| 55604_at | 8.0708 | 0.0012802 | 2.8927 | 0.0041653 | LRRC16A |
| 81606_at | -8.0701 | 0.0012807 | 2.8926 | 0.0041653 | LBH |
| 2026_at | 8.0665 | 0.0012828 | 2.8918 | 0.0041696 | ENO2 |
| 81562_at | 8.0607 | 0.0012863 | 2.8906 | 0.0041758 | LMAN2L |
| 2625_at | -8.0606 | 0.0012864 | 2.8906 | 0.0041758 | GATA3 |
| 4430_at | -8.0547 | 0.00129 | 2.8894 | 0.004183 | MYO1B |
| 2072_at | -8.0542 | 0.0012903 | 2.8893 | 0.004183 | ERCC4 |
| 8345_at | -8.0506 | 0.0012925 | 2.8886 | 0.0041874 | HIST1H2BH |
| 9334_at | -8.0471 | 0.0012946 | 2.8879 | 0.0041916 | B4GALT5 |
| 51306_at | -8.0435 | 0.0012969 | 2.8871 | 0.0041961 | FAM13B |
| 54477_at | 8.0414 | 0.0012981 | 2.8867 | 0.0041975 | PLEKHA5 |
| 51506_at | 8.0328 | 0.0013034 | 2.8849 | 0.0042119 | UFC1 |
| 8492_at | -8.0314 | 0.0013043 | 2.8846 | 0.0042119 | PRSS12 |
| 23335_at | -8.0301 | 0.0013051 | 2.8844 | 0.0042119 | WDR7 |
| 389941_at | 8.0219 | 0.0013102 | 2.8827 | 0.0042237 | C1QL3 |
| 5165_at | 8.0204 | 0.0013112 | 2.8823 | 0.0042237 | PDK3 |
| 4907_at | -8.0201 | 0.0013113 | 2.8823 | 0.0042237 | NT5E |
| 2202_at | 8.0158 | 0.001314 | 2.8814 | 0.0042277 | EFEMP1 |
| 60312_at | -8.0152 | 0.0013143 | 2.8813 | 0.0042277 | AFAP1 |
| 60678_at | 8.0132 | 0.0013156 | 2.8809 | 0.0042277 | EEFSEC |
| 150465_at | 8.0127 | 0.0013159 | 2.8808 | 0.0042277 | TTL |
| 10052_at | -8.0106 | 0.0013172 | 2.8803 | 0.0042292 | GJC1 |
| 146223_at | 8.0062 | 0.00132 | 2.8794 | 0.0042349 | CMTM4 |
| 7453_at | -8.0047 | 0.001321 | 2.8791 | 0.0042349 | WARS |
| 25938_at | -8.0037 | 0.0013215 | 2.8789 | 0.0042349 | HEATR5A |
| 3017_at | 7.9975 | 0.0013255 | 2.8776 | 0.0042421 | HIST1H2BD |
| 5429_at | -7.9975 | 0.0013255 | 2.8776 | 0.0042421 | POLH |
| 10098_at | -7.9955 | 0.0013267 | 2.8772 | 0.0042432 | TSPAN5 |
| 10512_at | -7.9897 | 0.0013304 | 2.876 | 0.0042516 | SEMA3C |
| 64840_at | -7.9887 | 0.001331 | 2.8758 | 0.0042516 | PORCN |
| 5918_at | -7.985 | 0.0013334 | 2.8751 | 0.0042523 | RARRES1 |
| 84302_at | 7.9849 | 0.0013334 | 2.875 | 0.0042523 | TMEM246 |
| 9173_at | -7.9843 | 0.0013338 | 2.8749 | 0.0042523 | IL1RL1 |
| 55692_at | -7.9828 | 0.0013348 | 2.8746 | 0.0042527 | LUC7L |
| 27247_at | 7.9785 | 0.0013375 | 2.8737 | 0.0042567 | NFU1 |
| 51166_at | 7.9782 | 0.0013377 | 2.8736 | 0.0042567 | AADAT |
| 253832_at | -7.9744 | 0.0013402 | 2.8728 | 0.0042617 | ZDHHC20 |
| 58497_at | 7.9701 | 0.0013429 | 2.872 | 0.0042678 | PRUNE |
| 5912_at | -7.9633 | 0.0013472 | 2.8706 | 0.0042788 | RAP2B |
| 10984_at | 7.9601 | 0.0013493 | 2.8699 | 0.0042804 | KCNQ1OT1 |
| 51310_at | 7.9595 | 0.0013497 | 2.8698 | 0.0042804 | SLC22A17 |
| 1069_at | 7.9574 | 0.0013511 | 2.8693 | 0.0042804 | CETN2 |
| 284702_at | -7.9566 | 0.0013516 | 2.8692 | 0.0042804 | HNRNPU-AS1 |
| 79649_at | 7.9559 | 0.001352 | 2.869 | 0.0042804 | MAP7D3 |
| 645431_at | -7.9522 | 0.0013544 | 2.8682 | 0.0042838 | FUT8-AS1 |
| 23341_at | 7.9506 | 0.0013555 | 2.8679 | 0.0042838 | DNAJC16 |
| 11264_at | 7.9503 | 0.0013557 | 2.8678 | 0.0042838 | PXMP4 |
| 647859_at | -7.9487 | 0.0013567 | 2.8675 | 0.0042843 | LOC647859 |
| 161291_at | -7.9443 | 0.0013595 | 2.8666 | 0.0042906 | TMEM30B |
| 7871_at | -7.9412 | 0.0013615 | 2.866 | 0.0042942 | SLMAP |
| 4809_at | 7.9378 | 0.0013637 | 2.8653 | 0.0042963 | NHP2L1 |
| 406956_at | -7.9366 | 0.0013646 | 2.865 | 0.0042963 | MIR181B2 |
| 23621_at | 7.9363 | 0.0013648 | 2.8649 | 0.0042963 | BACE1 |
| 175_at | 7.9336 | 0.0013665 | 2.8644 | 0.004299 | AGA |
| 51734_at | 7.9317 | 0.0013677 | 2.864 | 0.004299 | MSRB1 |
| 10232_at | -7.931 | 0.0013682 | 2.8638 | 0.004299 | MSLN |
| 23268_at | -7.9276 | 0.0013705 | 2.8631 | 0.0043034 | DNMBP |
| 11177_at | -7.9226 | 0.0013737 | 2.8621 | 0.0043096 | BAZ1A |
| 6453_at | 7.9219 | 0.0013742 | 2.862 | 0.0043096 | ITSN1 |
| 9526_at | 7.9135 | 0.0013798 | 2.8602 | 0.0043245 | MPDU1 |
| 4286_at | 7.9048 | 0.0013855 | 2.8584 | 0.0043398 | MITF |
| 23007_at | -7.9033 | 0.0013865 | 2.8581 | 0.0043402 | PLCH1 |
| 5547_at | 7.8984 | 0.0013898 | 2.857 | 0.0043478 | PRCP |
| 4814_at | 7.8799 | 0.0014022 | 2.8532 | 0.0043839 | NINJ1 |
| 10140_at | -7.8778 | 0.0014036 | 2.8527 | 0.0043855 | TOB1 |
| 401884_at | -7.8573 | 0.0014176 | 2.8484 | 0.0044264 | MGC57346 |
| 23417_at | 7.8552 | 0.0014191 | 2.848 | 0.0044281 | MLYCD |
| 6615_at | 7.845 | 0.0014261 | 2.8459 | 0.0044473 | SNAI1 |
| 54834_at | -7.8402 | 0.0014294 | 2.8449 | 0.0044485 | GDAP2 |
| 56993_at | 7.8385 | 0.0014305 | 2.8445 | 0.0044485 | TOMM22 |
| 102723465_at | -7.8374 | 0.0014313 | 2.8443 | 0.0044485 | LOC102723465 |
| 79611_at | 7.8364 | 0.0014321 | 2.844 | 0.0044485 | ACSS3 |
| 101954264_at | -7.8359 | 0.0014324 | 2.8439 | 0.0044485 | RNVU1-4 |
| 8569_at | -7.8356 | 0.0014326 | 2.8439 | 0.0044485 | MKNK1 |
| 84632_at | -7.8339 | 0.0014338 | 2.8435 | 0.0044485 | AFAP1L2 |
| 23382_at | 7.8336 | 0.0014339 | 2.8435 | 0.0044485 | AHCYL2 |
| 84181_at | -7.8314 | 0.0014355 | 2.843 | 0.0044485 | CHD6 |
| 100422979_at | -7.8307 | 0.001436 | 2.8429 | 0.0044485 | MIR4324 |
| 7110_at | -7.8303 | 0.0014363 | 2.8428 | 0.0044485 | TMF1 |
| 10826_at | -7.827 | 0.0014386 | 2.8421 | 0.0044528 | FAXDC2 |
| 177_at | -7.82 | 0.0014434 | 2.8406 | 0.0044651 | AGER |
| 11098_at | 7.8163 | 0.001446 | 2.8398 | 0.0044704 | PRSS23 |
| 83999_at | 7.811 | 0.0014498 | 2.8387 | 0.0044777 | KREMEN1 |
| 25828_at | 7.8104 | 0.0014502 | 2.8386 | 0.0044777 | TXN2 |
| 5328_at | -7.8039 | 0.0014548 | 2.8372 | 0.0044892 | PLAU |
| 139067_at | 7.8011 | 0.0014568 | 2.8366 | 0.0044925 | SPANXN3 |
| 406_at | 7.797 | 0.0014597 | 2.8357 | 0.0044966 | ARNTL |
| 6928_at | 7.7967 | 0.0014599 | 2.8357 | 0.0044966 | HNF1B |
| 83451_at | -7.7954 | 0.0014608 | 2.8354 | 0.0044966 | ABHD11 |
| 23057_at | -7.786 | 0.0014675 | 2.8334 | 0.0045145 | NMNAT2 |
| 55850_at | 7.7783 | 0.001473 | 2.8318 | 0.004524 | USE1 |
| 6654_at | -7.7782 | 0.0014731 | 2.8318 | 0.004524 | SOS1 |
| 4038_at | 7.7779 | 0.0014733 | 2.8317 | 0.004524 | LRP4 |
| 101929623_at | -7.7698 | 0.0014791 | 2.83 | 0.0045313 | LINC01215 |
| 650_at | 7.7687 | 0.00148 | 2.8297 | 0.0045313 | BMP2 |
| 64081_at | 7.7674 | 0.0014809 | 2.8295 | 0.0045313 | PBLD |
| 27133_at | -7.7667 | 0.0014814 | 2.8293 | 0.0045313 | KCNH5 |
| 2948_at | -7.7657 | 0.0014821 | 2.8291 | 0.0045313 | GSTM4 |
| 10950_at | -7.7649 | 0.0014827 | 2.8289 | 0.0045313 | BTG3 |
| 23413_at | 7.7647 | 0.0014828 | 2.8289 | 0.0045313 | NCS1 |
| 5395_at | 7.7646 | 0.0014829 | 2.8289 | 0.0045313 | PMS2 |
| 51304_at | -7.7625 | 0.0014845 | 2.8284 | 0.0045319 | ZDHHC3 |
| 10056_at | 7.7618 | 0.001485 | 2.8283 | 0.0045319 | FARSB |
| 5465_at | -7.7568 | 0.0014886 | 2.8272 | 0.0045402 | PPARA |
| 29062_at | 7.7479 | 0.0014951 | 2.8253 | 0.0045559 | WDR91 |
| 392_at | 7.7473 | 0.0014955 | 2.8252 | 0.0045559 | ARHGAP1 |
| 26355_at | 7.7457 | 0.0014967 | 2.8249 | 0.0045567 | FAM162A |
| 200058_at | -7.743 | 0.0014987 | 2.8243 | 0.0045599 | FLJ23867 |
| 6583_at | 7.7413 | 0.0014999 | 2.8239 | 0.0045608 | SLC22A4 |
| 55359_at | -7.7327 | 0.0015062 | 2.8221 | 0.0045773 | STYK1 |
| 100507032_at | 7.7311 | 0.0015074 | 2.8218 | 0.004578 | TMCC1-AS1 |
| 147372_at | -7.7285 | 0.0015093 | 2.8212 | 0.0045812 | CCBE1 |
| 7903_at | 7.7262 | 0.0015111 | 2.8207 | 0.0045836 | ST8SIA4 |
| 55220_at | -7.7216 | 0.0015145 | 2.8197 | 0.0045911 | KLHDC8A |
| 57178_at | 7.7156 | 0.0015189 | 2.8185 | 0.0046018 | ZMIZ1 |
| 55876_at | -7.7127 | 0.0015211 | 2.8178 | 0.0046056 | GSDMB |
| 117854_at | 7.7086 | 0.0015241 | 2.817 | 0.0046121 | TRIM6 |
| 442213_at | -7.7046 | 0.0015272 | 2.8161 | 0.0046185 | PTCHD4 |
| 9887_at | -7.7018 | 0.0015293 | 2.8155 | 0.004622 | SMG7 |
| 66005_at | 7.7005 | 0.0015302 | 2.8152 | 0.0046221 | CHID1 |
| 23331_at | 7.694 | 0.0015352 | 2.8138 | 0.0046342 | TTC28 |
| 1308_at | -7.6907 | 0.0015377 | 2.8131 | 0.004639 | COL17A1 |
| 1522_at | 7.6881 | 0.0015396 | 2.8126 | 0.0046422 | CTSZ |
| 5226_at | -7.6838 | 0.0015429 | 2.8117 | 0.0046491 | PGD |
| 100422737_at | 7.6743 | 0.0015501 | 2.8096 | 0.0046681 | LOC100422737 |
| 10965_at | 7.6683 | 0.0015547 | 2.8083 | 0.0046792 | ACOT2 |
| 375295_at | 7.6659 | 0.0015566 | 2.8078 | 0.0046798 | LINC01116 |
| 349565_at | 7.6656 | 0.0015568 | 2.8078 | 0.0046798 | NMNAT3 |
| 154_at | -7.6621 | 0.0015595 | 2.807 | 0.004685 | ADRB2 |
| 1652_at | 7.6602 | 0.001561 | 2.8066 | 0.0046867 | DDT |
| 587_at | 7.6581 | 0.0015626 | 2.8062 | 0.0046871 | BCAT2 |
| 55553_at | -7.657 | 0.0015634 | 2.8059 | 0.0046871 | SOX6 |
| 284071_at | -7.6563 | 0.0015639 | 2.8058 | 0.0046871 | C17orf104 |
| 5245_at | 7.6508 | 0.0015682 | 2.8046 | 0.004689 | PHB |
| 4486_at | -7.6501 | 0.0015688 | 2.8044 | 0.004689 | MST1R |
| 440482_at | -7.6491 | 0.0015695 | 2.8042 | 0.004689 | ANKRD20A5P |
| 3198_at | 7.6479 | 0.0015705 | 2.804 | 0.004689 | HOXA1 |
| 57167_at | -7.6471 | 0.0015711 | 2.8038 | 0.004689 | SALL4 |
| 64397_at | -7.646 | 0.0015719 | 2.8036 | 0.004689 | ZNF106 |
| 163404_at | 7.6457 | 0.0015722 | 2.8035 | 0.004689 | LPPR5 |
| 1277_at | 7.6447 | 0.0015729 | 2.8033 | 0.004689 | COL1A1 |
| 28987_at | 7.6447 | 0.001573 | 2.8033 | 0.004689 | NOB1 |
| 1889_at | -7.6399 | 0.0015767 | 2.8023 | 0.0046972 | ECE1 |
| 596_at | 7.6371 | 0.0015789 | 2.8016 | 0.0047011 | BCL2 |
| 100270746_at | -7.6339 | 0.0015814 | 2.801 | 0.0047057 | LOC100270746 |
| 57511_at | -7.6243 | 0.001589 | 2.7989 | 0.0047233 | COG6 |
| 135932_at | 7.624 | 0.0015892 | 2.7988 | 0.0047233 | TMEM139 |
| 53838_at | 7.6213 | 0.0015913 | 2.7982 | 0.0047266 | C11orf24 |
| 9179_at | 7.6196 | 0.0015927 | 2.7979 | 0.004728 | AP4M1 |
| 2146_at | -7.6138 | 0.0015973 | 2.7966 | 0.0047349 | EZH2 |
| 5017_at | -7.6126 | 0.0015982 | 2.7964 | 0.0047349 | OVOL1 |
| 30817_at | -7.6126 | 0.0015983 | 2.7964 | 0.0047349 | EMR2 |
| 518_at | 7.6118 | 0.0015988 | 2.7962 | 0.0047349 | ATP5G3 |
| 8614_at | 7.6078 | 0.001602 | 2.7953 | 0.0047415 | STC2 |
| 84329_at | -7.6014 | 0.0016072 | 2.7939 | 0.0047539 | HVCN1 |
| 8458_at | -7.5989 | 0.0016092 | 2.7934 | 0.0047571 | TTF2 |
| 26268_at | 7.593 | 0.0016139 | 2.7921 | 0.0047681 | FBXO9 |
| 1292_at | -7.5886 | 0.0016174 | 2.7912 | 0.0047742 | COL6A2 |
| 55536_at | -7.587 | 0.0016188 | 2.7908 | 0.0047742 | CDCA7L |
| 9265_at | -7.5858 | 0.0016197 | 2.7906 | 0.0047742 | CYTH3 |
| 55357_at | 7.5857 | 0.0016198 | 2.7905 | 0.0047742 | TBC1D2 |
| 8974_at | 7.5846 | 0.0016207 | 2.7903 | 0.0047742 | P4HA2 |
| 4756_at | 7.5819 | 0.0016228 | 2.7897 | 0.0047777 | NEO1 |
| 28232_at | -7.5796 | 0.0016247 | 2.7892 | 0.0047789 | SLCO3A1 |
| 23062_at | 7.5791 | 0.0016252 | 2.7891 | 0.0047789 | GGA2 |
| 10413_at | 7.5764 | 0.0016273 | 2.7885 | 0.0047824 | YAP1 |
| 3421_at | 7.5694 | 0.001633 | 2.787 | 0.0047963 | IDH3G |
| 10218_at | 7.5662 | 0.0016356 | 2.7863 | 0.0047994 | ANGPTL7 |
| 9213_at | 7.5658 | 0.001636 | 2.7862 | 0.0047994 | XPR1 |
| 387263_at | 7.564 | 0.0016374 | 2.7858 | 0.0048008 | C6orf120 |
| 51762_at | 7.5569 | 0.0016433 | 2.7843 | 0.0048147 | RAB8B |
| 25902_at | -7.5553 | 0.0016445 | 2.784 | 0.0048147 | MTHFD1L |
| 3437_at | -7.5547 | 0.001645 | 2.7838 | 0.0048147 | IFIT3 |
| 928_at | -7.5514 | 0.0016478 | 2.7831 | 0.00482 | CD9 |
| 57458_at | 7.5308 | 0.0016649 | 2.7786 | 0.004864 | TMCC3 |
| 56975_at | 7.5304 | 0.0016652 | 2.7785 | 0.004864 | FAM20C |
| 29968_at | 7.5297 | 0.0016658 | 2.7784 | 0.004864 | PSAT1 |
| 10654_at | 7.5282 | 0.0016671 | 2.778 | 0.004865 | PMVK |
| 83938_at | 7.5263 | 0.0016686 | 2.7776 | 0.0048667 | C10orf11 |
| 55602_at | -7.5247 | 0.00167 | 2.7773 | 0.0048678 | CDKN2AIP |
| 10724_at | -7.5218 | 0.0016724 | 2.7767 | 0.004872 | MGEA5 |
| 64065_at | -7.5102 | 0.0016822 | 2.7741 | 0.0048978 | PERP |
| 120_at | 7.5064 | 0.0016854 | 2.7733 | 0.0049041 | ADD3 |
| 3431_at | 7.5045 | 0.001687 | 2.7729 | 0.004906 | SP110 |
| 2181_at | -7.5008 | 0.0016902 | 2.7721 | 0.0049123 | ACSL3 |
| 5745_at | 7.4942 | 0.0016958 | 2.7706 | 0.0049259 | PTH1R |
| 833_at | -7.4864 | 0.0017025 | 2.7689 | 0.0049425 | CARS |
| 1185_at | -7.4813 | 0.0017069 | 2.7678 | 0.0049523 | CLCN6 |
| 5336_at | -7.4794 | 0.0017086 | 2.7674 | 0.0049542 | PLCG2 |
| 84856_at | 7.4717 | 0.0017152 | 2.7657 | 0.0049707 | LINC00839 |
| 101929604_at | 7.4683 | 0.0017181 | 2.7649 | 0.0049762 | LOC101929604 |
| 9957_at | -7.4492 | 0.0017348 | 2.7608 | 0.0050201 | HS3ST1 |
| 29914_at | 7.4487 | 0.0017353 | 2.7606 | 0.0050201 | UBIAD1 |
| 128414_at | 7.4468 | 0.001737 | 2.7602 | 0.0050221 | NKAIN4 |
| 440107_at | -7.4416 | 0.0017415 | 2.7591 | 0.0050323 | PLEKHG7 |
| 55742_at | 7.4364 | 0.0017462 | 2.7579 | 0.0050428 | PARVA |
| 1893_at | -7.4328 | 0.0017494 | 2.7571 | 0.0050491 | ECM1 |
| 26091_at | -7.4288 | 0.0017529 | 2.7563 | 0.0050563 | HERC4 |
| 101927412_at | 7.425 | 0.0017563 | 2.7554 | 0.0050633 | LOC101927412 |
| 5324_at | -7.4234 | 0.0017577 | 2.755 | 0.0050645 | PLAG1 |
| 171568_at | 7.4185 | 0.001762 | 2.754 | 0.0050726 | POLR3H |
| 28984_at | -7.418 | 0.0017626 | 2.7539 | 0.0050726 | RGCC |
| 56104_at | 7.4139 | 0.0017662 | 2.753 | 0.0050765 | PCDHGB1 |
| 5430_at | 7.4135 | 0.0017665 | 2.7529 | 0.0050765 | POLR2A |
| 1491_at | -7.413 | 0.001767 | 2.7528 | 0.0050765 | CTH |
| 3065_at | 7.4082 | 0.0017713 | 2.7517 | 0.0050825 | HDAC1 |
| 164781_at | -7.4081 | 0.0017715 | 2.7517 | 0.0050825 | DAW1 |
| 677825_at | 7.4073 | 0.0017721 | 2.7515 | 0.0050825 | SNORA44 |
| 1477_at | 7.3987 | 0.0017799 | 2.7496 | 0.0051007 | CSTF1 |
| 728609_at | 7.3981 | 0.0017805 | 2.7495 | 0.0051007 | SDHAP3 |
| 140606_at | 7.3897 | 0.0017882 | 2.7476 | 0.0051175 | SELM |
| 51537_at | 7.3894 | 0.0017884 | 2.7475 | 0.0051175 | MTFP1 |
| 286189_at | -7.3877 | 0.00179 | 2.7472 | 0.0051181 | LOC286189 |
| 9074_at | 7.3869 | 0.0017907 | 2.747 | 0.0051181 | CLDN6 |
| 102723361_at | -7.3851 | 0.0017923 | 2.7466 | 0.0051199 | LOC102723361 |
| 93129_at | 7.3827 | 0.0017946 | 2.746 | 0.0051234 | ORAI3 |
| 338069_at | -7.375 | 0.0018016 | 2.7443 | 0.0051406 | ST7-OT4 |
| 28951_at | -7.3702 | 0.001806 | 2.7433 | 0.0051502 | TRIB2 |
| 51351_at | -7.3648 | 0.001811 | 2.7421 | 0.0051615 | ZNF117 |
| 22941_at | 7.3628 | 0.0018129 | 2.7416 | 0.005164 | SHANK2 |
| 57556_at | 7.3576 | 0.0018177 | 2.7405 | 0.0051729 | SEMA6A |
| 339761_at | -7.3563 | 0.0018189 | 2.7402 | 0.0051729 | CYP27C1 |
| 56937_at | -7.3561 | 0.0018192 | 2.7401 | 0.0051729 | PMEPA1 |
| 80351_at | -7.3453 | 0.0018293 | 2.7377 | 0.0051987 | TNKS2 |
| 5382_at | 7.3421 | 0.0018323 | 2.737 | 0.0052043 | PMS2P4 |
| 2201_at | -7.3396 | 0.0018346 | 2.7365 | 0.0052079 | FBN2 |
| 27347_at | -7.3377 | 0.0018364 | 2.736 | 0.0052101 | STK39 |
| 3728_at | -7.3347 | 0.0018392 | 2.7354 | 0.0052151 | JUP |
| 10336_at | -7.3329 | 0.001841 | 2.735 | 0.0052171 | PCGF3 |
| 63910_at | -7.3306 | 0.0018431 | 2.7344 | 0.0052187 | SLC17A9 |
| 100129543_at | -7.3293 | 0.0018443 | 2.7342 | 0.0052187 | ZNF730 |
| 57834_at | -7.3289 | 0.0018447 | 2.7341 | 0.0052187 | CYP4F11 |
| 5899_at | 7.325 | 0.0018484 | 2.7332 | 0.0052265 | RALB |
| 7779_at | 7.3199 | 0.0018533 | 2.7321 | 0.0052372 | SLC30A1 |
| 2997_at | 7.3178 | 0.0018553 | 2.7316 | 0.0052381 | GYS1 |
| 54986_at | 7.3174 | 0.0018557 | 2.7315 | 0.0052381 | ULK4 |
| 5351_at | 7.314 | 0.0018589 | 2.7307 | 0.0052443 | PLOD1 |
| 8804_at | 7.3116 | 0.0018612 | 2.7302 | 0.0052473 | CREG1 |
| 200373_at | 7.3106 | 0.0018621 | 2.73 | 0.0052473 | CFAP221 |
| 8425_at | -7.3088 | 0.0018639 | 2.7296 | 0.0052493 | LTBP4 |
| 3298_at | -7.3044 | 0.0018681 | 2.7286 | 0.0052582 | HSF2 |
| 79180_at | -7.3023 | 0.0018701 | 2.7281 | 0.0052601 | EFHD2 |
| 55819_at | 7.3016 | 0.0018709 | 2.728 | 0.0052601 | RNF130 |
| 6455_at | 7.2991 | 0.0018733 | 2.7274 | 0.0052639 | SH3GL1 |
| 80235_at | 7.2844 | 0.0018875 | 2.7241 | 0.005301 | PIGZ |
| 407035_at | -7.2772 | 0.0018945 | 2.7225 | 0.0053177 | MIR31 |
| 11230_at | 7.2753 | 0.0018965 | 2.7221 | 0.0053201 | PRAF2 |
| 284266_at | 7.2709 | 0.0019007 | 2.7211 | 0.0053265 | SIGLEC15 |
| 80169_at | -7.2697 | 0.001902 | 2.7208 | 0.0053265 | CTC1 |
| 2738_at | 7.2697 | 0.001902 | 2.7208 | 0.0053265 | GLI4 |
| 2801_at | -7.2678 | 0.0019038 | 2.7204 | 0.0053279 | GOLGA2 |
| 151050_at | 7.267 | 0.0019046 | 2.7202 | 0.0053279 | KANSL1L |
| 5155_at | -7.2625 | 0.001909 | 2.7192 | 0.0053359 | PDGFB |
| 692211_at | -7.262 | 0.0019096 | 2.7191 | 0.0053359 | SNORD98 |
| 102723596_at | -7.2607 | 0.0019109 | 2.7188 | 0.0053365 | LOC102723596 |
| 7128_at | -7.255 | 0.0019164 | 2.7175 | 0.0053491 | TNFAIP3 |
| 3799_at | -7.2503 | 0.0019212 | 2.7164 | 0.0053594 | KIF5B |
| 100873920_at | 7.2452 | 0.0019262 | 2.7153 | 0.0053704 | NHS-AS1 |
| 79465_at | 7.2439 | 0.0019275 | 2.715 | 0.0053706 | ULBP3 |
| 60489_at | -7.243 | 0.0019284 | 2.7148 | 0.0053706 | APOBEC3G |
| 2004_at | -7.2408 | 0.0019306 | 2.7143 | 0.0053737 | ELK3 |
| 256364_at | 7.2374 | 0.0019341 | 2.7135 | 0.0053788 | EML3 |
| 3087_at | -7.2369 | 0.0019346 | 2.7134 | 0.0053788 | HHEX |
| 166968_at | -7.2354 | 0.0019361 | 2.7131 | 0.00538 | MIER3 |
| 9330_at | -7.2316 | 0.00194 | 2.7122 | 0.0053852 | GTF3C3 |
| 94134_at | -7.2314 | 0.0019401 | 2.7122 | 0.0053852 | ARHGAP12 |
| 9683_at | -7.2288 | 0.0019427 | 2.7116 | 0.0053869 | N4BP1 |
| 2122_at | 7.2282 | 0.0019434 | 2.7114 | 0.0053869 | MECOM |
| 8613_at | 7.2266 | 0.001945 | 2.7111 | 0.0053869 | PPAP2B |
| 55968_at | -7.2265 | 0.0019451 | 2.7111 | 0.0053869 | NSFL1C |
| 83696_at | -7.2252 | 0.0019464 | 2.7108 | 0.0053876 | TRAPPC9 |
| 9567_at | -7.2231 | 0.0019486 | 2.7103 | 0.0053906 | GTPBP1 |
| 9240_at | 7.2217 | 0.0019499 | 2.71 | 0.0053914 | PNMA1 |
| 8418_at | 7.2205 | 0.0019512 | 2.7097 | 0.0053919 | CMAHP |
| 102723542_at | -7.2158 | 0.001956 | 2.7086 | 0.0054022 | LOC102723542 |
| 55818_at | 7.2137 | 0.0019581 | 2.7082 | 0.0054049 | KDM3A |
| 57719_at | -7.2094 | 0.0019624 | 2.7072 | 0.0054141 | ANO8 |
| 389906_at | -7.2054 | 0.0019666 | 2.7063 | 0.0054212 | LOC389906 |
| 1894_at | -7.204 | 0.001968 | 2.706 | 0.0054212 | ECT2 |
| 64108_at | -7.2027 | 0.0019693 | 2.7057 | 0.0054212 | RTP4 |
| 9821_at | -7.2027 | 0.0019694 | 2.7057 | 0.0054212 | RB1CC1 |
| 23765_at | 7.1994 | 0.0019728 | 2.7049 | 0.0054276 | IL17RA |
| 23515_at | -7.1903 | 0.0019822 | 2.7029 | 0.0054504 | MORC3 |
| 92521_at | -7.1867 | 0.0019859 | 2.702 | 0.0054553 | SPECC1 |
| 101929475_at | 7.1865 | 0.0019861 | 2.702 | 0.0054553 | LOC101929475 |
| 220164_at | 7.1851 | 0.0019876 | 2.7017 | 0.0054562 | DOK6 |
| 2821_at | 7.1736 | 0.0019996 | 2.6991 | 0.0054862 | GPI |
| 6652_at | 7.1694 | 0.0020039 | 2.6981 | 0.0054951 | SORD |
| 5349_at | -7.1674 | 0.002006 | 2.6977 | 0.0054959 | FXYD3 |
| 6723_at | 7.1671 | 0.0020064 | 2.6976 | 0.0054959 | SRM |
| 54518_at | 7.165 | 0.0020086 | 2.6971 | 0.0054988 | APBB1IP |
| 59339_at | 7.1636 | 0.0020101 | 2.6968 | 0.0055 | PLEKHA2 |
| 148789_at | -7.162 | 0.0020117 | 2.6964 | 0.0055014 | B3GALNT2 |
| 64220_at | -7.1592 | 0.0020147 | 2.6958 | 0.0055066 | STRA6 |
| 113829_at | 7.1545 | 0.0020197 | 2.6947 | 0.0055165 | SLC35A4 |
| 5167_at | 7.1537 | 0.0020205 | 2.6945 | 0.0055165 | ENPP1 |
| 151195_at | -7.1493 | 0.0020252 | 2.6935 | 0.0055238 | CCNYL1 |
| 2892_at | 7.149 | 0.0020255 | 2.6935 | 0.0055238 | GRIA3 |
| 9975_at | -7.1477 | 0.0020269 | 2.6932 | 0.0055238 | NR1D2 |
| 1632_at | 7.147 | 0.0020277 | 2.693 | 0.0055238 | ECI1 |
| 54843_at | 7.1445 | 0.0020304 | 2.6924 | 0.0055238 | SYTL2 |
| 22974_at | -7.1444 | 0.0020305 | 2.6924 | 0.0055238 | TPX2 |
| 406955_at | -7.1434 | 0.0020315 | 2.6922 | 0.0055238 | MIR181B1 |
| 400569_at | 7.1429 | 0.002032 | 2.6921 | 0.0055238 | MED11 |
| 11320_at | -7.1412 | 0.0020338 | 2.6917 | 0.0055256 | MGAT4A |
| 5376_at | 7.1394 | 0.0020358 | 2.6913 | 0.0055279 | PMP22 |
| 8609_at | 7.1347 | 0.0020409 | 2.6902 | 0.005536 | KLF7 |
| 79991_at | -7.1346 | 0.002041 | 2.6902 | 0.005536 | OBFC1 |
| 50804_at | -7.1323 | 0.0020435 | 2.6896 | 0.0055398 | MYEF2 |
| 26230_at | -7.1294 | 0.0020466 | 2.689 | 0.0055452 | TIAM2 |
| 9609_at | 7.1276 | 0.0020485 | 2.6886 | 0.0055465 | RAB36 |
| 6867_at | -7.1269 | 0.0020493 | 2.6884 | 0.0055465 | TACC1 |
| 25903_at | 7.123 | 0.0020535 | 2.6875 | 0.0055526 | OLFML2B |
| 604_at | 7.1227 | 0.0020538 | 2.6874 | 0.0055526 | BCL6 |
| 55915_at | 7.1184 | 0.0020584 | 2.6865 | 0.005561 | LANCL2 |
| 10549_at | 7.1178 | 0.0020591 | 2.6863 | 0.005561 | PRDX4 |
| 85458_at | 7.1144 | 0.0020628 | 2.6855 | 0.0055662 | DIXDC1 |
| 2872_at | -7.114 | 0.0020632 | 2.6855 | 0.0055662 | MKNK2 |
| 55145_at | 7.1092 | 0.0020684 | 2.6844 | 0.0055772 | THAP1 |
| 170959_at | -7.107 | 0.0020709 | 2.6838 | 0.0055808 | ZNF431 |
| 25874_at | 7.1047 | 0.0020734 | 2.6833 | 0.0055838 | MPC2 |
| 8476_at | -7.1037 | 0.0020744 | 2.6831 | 0.0055838 | CDC42BPA |
| 154881_at | 7.1029 | 0.0020754 | 2.6829 | 0.0055838 | KCTD7 |
| 9649_at | -7.0996 | 0.002079 | 2.6821 | 0.0055907 | RALGPS1 |
| 196051_at | 7.0951 | 0.0020839 | 2.6811 | 0.0055972 | PPAPDC1A |
| 53630_at | 7.0944 | 0.0020847 | 2.6809 | 0.0055972 | BCO1 |
| 340267_at | -7.0935 | 0.0020858 | 2.6807 | 0.0055972 | COL28A1 |
| 94240_at | -7.0921 | 0.0020872 | 2.6804 | 0.0055972 | EPSTI1 |
| 4281_at | -7.091 | 0.0020885 | 2.6802 | 0.0055972 | MID1 |
| 6183_at | 7.0908 | 0.0020887 | 2.6801 | 0.0055972 | MRPS12 |
| 1474_at | -7.0901 | 0.0020895 | 2.68 | 0.0055972 | CST6 |
| 3631_at | -7.0892 | 0.0020904 | 2.6798 | 0.0055972 | INPP4A |
| 8724_at | 7.0731 | 0.0021084 | 2.676 | 0.0056423 | SNX3 |
| 84958_at | -7.0692 | 0.0021127 | 2.6752 | 0.005651 | SYTL1 |
| 160851_at | -7.0642 | 0.0021183 | 2.674 | 0.0056628 | DGKH |
| 9735_at | -7.0607 | 0.0021223 | 2.6732 | 0.0056674 | KNTC1 |
| 5495_at | -7.0597 | 0.0021234 | 2.673 | 0.0056674 | PPM1B |
| 3783_at | -7.0596 | 0.0021236 | 2.6729 | 0.0056674 | KCNN4 |
| 2863_at | -7.0587 | 0.0021246 | 2.6727 | 0.0056674 | GPR39 |
| 91663_at | -7.0577 | 0.0021257 | 2.6725 | 0.0056674 | MYADM |
| 10486_at | 7.0529 | 0.0021312 | 2.6714 | 0.0056789 | CAP2 |
| 8038_at | 7.049 | 0.0021355 | 2.6705 | 0.0056876 | ADAM12 |
| 80221_at | -7.0456 | 0.0021394 | 2.6697 | 0.0056922 | ACSF2 |
| 29099_at | 7.0455 | 0.0021396 | 2.6697 | 0.0056922 | COMMD9 |
| 101930033_at | -7.0404 | 0.0021454 | 2.6685 | 0.0057047 | 101930033_at |
| 388559_at | -7.0354 | 0.0021511 | 2.6673 | 0.0057159 | ZNF888 |
| 132_at | -7.0347 | 0.0021519 | 2.6672 | 0.0057159 | ADK |
| 692063_at | 7.0324 | 0.0021546 | 2.6666 | 0.0057199 | SNORA32 |
| 10999_at | 7.0286 | 0.0021588 | 2.6658 | 0.0057283 | SLC27A4 |
| 23102_at | -7.0242 | 0.0021639 | 2.6648 | 0.0057387 | TBC1D2B |
| 10411_at | 7.0162 | 0.0021733 | 2.6629 | 0.0057604 | RAPGEF3 |
| 3775_at | -7.0137 | 0.0021761 | 2.6623 | 0.0057618 | KCNK1 |
| 23213_at | 7.0137 | 0.0021761 | 2.6623 | 0.0057618 | SULF1 |
| 401145_at | -7.0114 | 0.0021788 | 2.6618 | 0.0057659 | CCSER1 |
| 79760_at | 7.0097 | 0.0021807 | 2.6614 | 0.005768 | GEMIN7 |
| 201161_at | -7.0031 | 0.0021884 | 2.6599 | 0.0057853 | CENPV |
| 1410_at | 7.0017 | 0.0021901 | 2.6595 | 0.0057867 | CRYAB |
| 1950_at | -6.9917 | 0.0022019 | 2.6572 | 0.0058149 | EGF |
| 157_at | -6.9838 | 0.0022113 | 2.6554 | 0.005836 | ADRBK2 |
| 23576_at | -6.9829 | 0.0022123 | 2.6552 | 0.005836 | DDAH1 |
| 51099_at | 6.9805 | 0.0022152 | 2.6546 | 0.005839 | ABHD5 |
| 102724129_at | 6.98 | 0.0022157 | 2.6545 | 0.005839 | LOC102724129 |
| 26995_at | 6.9709 | 0.0022266 | 2.6524 | 0.0058646 | TRUB2 |
| 7076_at | 6.9662 | 0.0022323 | 2.6513 | 0.0058763 | TIMP1 |
| 83700_at | -6.9623 | 0.0022369 | 2.6503 | 0.0058855 | JAM3 |
| 55755_at | -6.9589 | 0.002241 | 2.6496 | 0.005893 | CDK5RAP2 |
| 647309_at | 6.9522 | 0.0022491 | 2.648 | 0.0059113 | GMNC |
| 9411_at | -6.9417 | 0.0022619 | 2.6455 | 0.0059419 | ARHGAP29 |
| 5271_at | 6.9371 | 0.0022676 | 2.6444 | 0.0059536 | SERPINB8 |
| 55026_at | 6.9308 | 0.0022753 | 2.643 | 0.0059707 | TMEM255A |
| 4084_at | -6.924 | 0.0022836 | 2.6414 | 0.0059892 | MXD1 |
| 10434_at | -6.9025 | 0.0023104 | 2.6363 | 0.0060564 | LYPLA1 |
| 79783_at | 6.8947 | 0.0023201 | 2.6345 | 0.0060767 | SUGCT |
| 6421_at | -6.8944 | 0.0023206 | 2.6344 | 0.0060767 | SFPQ |
| 148304_at | -6.8885 | 0.0023279 | 2.633 | 0.0060889 | C1orf74 |
| 64895_at | -6.8881 | 0.0023285 | 2.6329 | 0.0060889 | PAPOLG |
| 26960_at | 6.8878 | 0.0023289 | 2.6329 | 0.0060889 | NBEA |
| 2632_at | 6.8846 | 0.0023329 | 2.6321 | 0.0060935 | GBE1 |
| 2175_at | -6.8845 | 0.002333 | 2.6321 | 0.0060935 | FANCA |
| 339166_at | -6.8819 | 0.0023363 | 2.6315 | 0.0060988 | LOC339166 |
| 3182_at | 6.8801 | 0.0023385 | 2.6311 | 0.0061009 | HNRNPAB |
| 9657_at | -6.8793 | 0.0023396 | 2.6309 | 0.0061009 | IQCB1 |
| 9262_at | -6.865 | 0.0023579 | 2.6275 | 0.0061456 | STK17B |
| 10628_at | -6.8606 | 0.0023636 | 2.6264 | 0.0061571 | TXNIP |
| 8372_at | 6.8594 | 0.0023651 | 2.6262 | 0.0061578 | HYAL3 |
| 23507_at | -6.8582 | 0.0023667 | 2.6259 | 0.0061588 | LRRC8B |
| 7593_at | 6.856 | 0.0023695 | 2.6253 | 0.006163 | MZF1 |
| 10001_at | -6.8531 | 0.0023733 | 2.6247 | 0.0061695 | MED6 |
| 56241_at | -6.8511 | 0.0023759 | 2.6242 | 0.006173 | SUSD2 |
| 84790_at | 6.8473 | 0.0023807 | 2.6233 | 0.0061822 | TUBA1C |
| 10799_at | 6.8465 | 0.0023818 | 2.6231 | 0.0061822 | RPP40 |
| 102723409_at | -6.843 | 0.0023863 | 2.6223 | 0.0061906 | LOC102723409 |
| 5091_at | 6.8406 | 0.0023895 | 2.6217 | 0.0061957 | PC |
| 83548_at | -6.8381 | 0.0023927 | 2.6211 | 0.0062008 | COG3 |
| 6664_at | -6.8346 | 0.0023973 | 2.6203 | 0.0062095 | SOX11 |
| 23216_at | -6.8318 | 0.002401 | 2.6196 | 0.0062157 | TBC1D1 |
| 7905_at | 6.8274 | 0.0024068 | 2.6186 | 0.0062275 | REEP5 |
| 3215_at | -6.8238 | 0.0024115 | 2.6177 | 0.0062365 | HOXB5 |
| 3638_at | -6.8208 | 0.0024155 | 2.617 | 0.0062437 | INSIG1 |
| 23176_at | -6.813 | 0.0024258 | 2.6151 | 0.006267 | 8-Sep |
| 10234_at | 6.8087 | 0.0024316 | 2.6141 | 0.0062787 | LRRC17 |
| 93487_at | 6.805 | 0.0024366 | 2.6132 | 0.0062882 | MAPK1IP1L |
| 9169_at | -6.7994 | 0.002444 | 2.6119 | 0.0063041 | SCAF11 |
| 10144_at | 6.7985 | 0.0024452 | 2.6117 | 0.0063041 | FAM13A |
| 288_at | -6.7975 | 0.0024465 | 2.6114 | 0.0063042 | ANK3 |
| 6535_at | 6.7935 | 0.0024519 | 2.6105 | 0.0063148 | SLC6A8 |
| 6609_at | 6.7917 | 0.0024543 | 2.6101 | 0.0063178 | SMPD1 |
| 159686_at | -6.7888 | 0.0024582 | 2.6094 | 0.0063246 | CFAP58 |
| 8543_at | -6.7871 | 0.0024606 | 2.609 | 0.0063273 | LMO4 |
| 94121_at | -6.7801 | 0.0024701 | 2.6073 | 0.0063468 | SYTL4 |
| 23394_at | -6.7792 | 0.0024713 | 2.6071 | 0.0063468 | ADNP |
| 10849_at | 6.7787 | 0.002472 | 2.607 | 0.0063468 | CD3EAP |
| 51253_at | 6.7765 | 0.002475 | 2.6064 | 0.0063514 | MRPL37 |
| 1576_at | 6.7741 | 0.0024782 | 2.6059 | 0.0063548 | CYP3A4 |
| 4052_at | -6.7732 | 0.0024795 | 2.6056 | 0.0063548 | LTBP1 |
| 2901_at | -6.7727 | 0.0024801 | 2.6055 | 0.0063548 | GRIK5 |
| 7351_at | -6.7686 | 0.0024858 | 2.6045 | 0.006366 | UCP2 |
| 29940_at | -6.7668 | 0.0024882 | 2.6041 | 0.006369 | DSE |
| 3714_at | 6.7656 | 0.0024899 | 2.6038 | 0.0063701 | JAG2 |
| 4494_at | 6.7635 | 0.0024928 | 2.6033 | 0.0063743 | MT1F |
| 124637_at | 6.7605 | 0.0024969 | 2.6026 | 0.0063785 | CYB5D1 |
| 81619_at | -6.7598 | 0.0024979 | 2.6024 | 0.0063785 | TSPAN14 |
| 284119_at | 6.7595 | 0.0024983 | 2.6024 | 0.0063785 | PTRF |
| 4830_at | 6.7582 | 0.0025001 | 2.602 | 0.0063791 | NME1 |
| 9021_at | 6.7574 | 0.0025011 | 2.6019 | 0.0063791 | SOCS3 |
| 11035_at | -6.7539 | 0.002506 | 2.601 | 0.0063883 | RIPK3 |
| 23406_at | 6.7497 | 0.0025118 | 2.6 | 0.0064 | COTL1 |
| 693201_at | -6.745 | 0.0025184 | 2.5989 | 0.0064134 | MIR616 |
| 220323_at | 6.7374 | 0.0025289 | 2.5971 | 0.0064349 | OAF |
| 1062_at | -6.7371 | 0.0025294 | 2.597 | 0.0064349 | CENPE |
| 256714_at | 6.735 | 0.0025324 | 2.5965 | 0.0064392 | MAP7D2 |
| 441054_at | 6.7336 | 0.0025343 | 2.5961 | 0.0064406 | C4orf47 |
| 10537_at | -6.7325 | 0.0025358 | 2.5959 | 0.0064413 | UBD |
| 6482_at | 6.7283 | 0.0025418 | 2.5949 | 0.0064502 | ST3GAL1 |
| 1173_at | 6.7282 | 0.0025419 | 2.5948 | 0.0064502 | AP2M1 |
| 84879_at | 6.7239 | 0.002548 | 2.5938 | 0.0064624 | MFSD2A |
| 100302287_at | -6.7217 | 0.002551 | 2.5933 | 0.0064669 | MIR548H3 |
| 8884_at | 6.7187 | 0.0025553 | 2.5926 | 0.0064744 | SLC5A6 |
| 399959_at | -6.7166 | 0.0025583 | 2.5921 | 0.0064786 | MIR100HG |
| 58494_at | -6.715 | 0.0025605 | 2.5917 | 0.0064811 | JAM2 |
| 9415_at | -6.6948 | 0.0025895 | 2.5868 | 0.0065479 | FADS2 |
| 55274_at | -6.6947 | 0.0025896 | 2.5868 | 0.0065479 | PHF10 |
| 64080_at | 6.6816 | 0.0026087 | 2.5836 | 0.0065929 | RBKS |
| 7096_at | -6.6794 | 0.0026119 | 2.583 | 0.0065963 | TLR1 |
| 57415_at | 6.6788 | 0.0026127 | 2.5829 | 0.0065963 | C3orf14 |
| 30_at | 6.6772 | 0.0026151 | 2.5825 | 0.0065985 | ACAA1 |
| 101927513_at | -6.6764 | 0.0026162 | 2.5823 | 0.0065985 | LOC101927513 |
| 9342_at | 6.6696 | 0.0026261 | 2.5807 | 0.0066201 | SNAP29 |
| 79799_at | 6.663 | 0.0026358 | 2.5791 | 0.0066413 | UGT2A3 |
| 56109_at | 6.6609 | 0.0026389 | 2.5786 | 0.0066457 | PCDHGA6 |
| 64708_at | -6.6547 | 0.0026481 | 2.5771 | 0.0066656 | COPS7B |
| 128338_at | -6.6481 | 0.0026578 | 2.5755 | 0.0066867 | DRAM2 |
| 353322_at | 6.6399 | 0.0026701 | 2.5735 | 0.0067141 | ANKRD37 |
| 10893_at | 6.6364 | 0.0026753 | 2.5726 | 0.0067206 | MMP24 |
| 7185_at | -6.6364 | 0.0026754 | 2.5726 | 0.0067206 | TRAF1 |
| 79101_at | -6.6336 | 0.0026795 | 2.5719 | 0.0067278 | TAF1D |
| 2081_at | -6.6317 | 0.0026824 | 2.5715 | 0.0067315 | ERN1 |
| 10797_at | -6.6292 | 0.0026862 | 2.5709 | 0.0067378 | MTHFD2 |
| 79029_at | -6.6275 | 0.0026887 | 2.5705 | 0.0067386 | SPATA5L1 |
| 652276_at | 6.6272 | 0.0026892 | 2.5704 | 0.0067386 | LOC652276 |
| 83892_at | 6.6242 | 0.0026938 | 2.5696 | 0.0067465 | KCTD10 |
| 5218_at | -6.6187 | 0.002702 | 2.5683 | 0.0067638 | CDK14 |
| 1974_at | -6.6148 | 0.002708 | 2.5674 | 0.0067754 | EIF4A2 |
| 140564_at | -6.6127 | 0.0027112 | 2.5668 | 0.0067801 | APOBEC3D |
| 7850_at | -6.6092 | 0.0027165 | 2.566 | 0.00679 | IL1R2 |
| 79170_at | -6.6077 | 0.0027188 | 2.5656 | 0.0067923 | PRR15L |
| 9424_at | -6.6062 | 0.0027212 | 2.5652 | 0.0067948 | KCNK6 |
| 92912_at | 6.604 | 0.0027246 | 2.5647 | 0.0067973 | UBE2Q2 |
| 102723946_at | 6.6034 | 0.0027255 | 2.5646 | 0.0067973 | LOC102723946 |
| 151556_at | 6.6029 | 0.0027263 | 2.5644 | 0.0067973 | GPR155 |
| 129607_at | -6.5982 | 0.0027335 | 2.5633 | 0.006812 | CMPK2 |
| 7984_at | -6.5859 | 0.0027524 | 2.5603 | 0.0068557 | ARHGEF5 |
| 101926917_at | 6.5844 | 0.0027548 | 2.5599 | 0.0068582 | LOC101926917 |
| 3653_at | -6.5758 | 0.0027682 | 2.5578 | 0.0068842 | IPW |
| 643650_at | -6.5753 | 0.002769 | 2.5577 | 0.0068842 | LINC00842 |
| 54407_at | -6.5751 | 0.0027694 | 2.5576 | 0.0068842 | SLC38A2 |
| 2831_at | 6.5724 | 0.0027735 | 2.557 | 0.0068906 | NPBWR1 |
| 2023_at | 6.5716 | 0.0027747 | 2.5568 | 0.0068906 | ENO1 |
| 100874194_at | 6.5678 | 0.0027807 | 2.5558 | 0.0069022 | CLDN10-AS1 |
| 90871_at | 6.5669 | 0.0027822 | 2.5556 | 0.0069025 | TMEM261 |
| 3339_at | -6.5598 | 0.0027933 | 2.5539 | 0.0069265 | HSPG2 |
| 113763_at | 6.5589 | 0.0027948 | 2.5536 | 0.0069268 | ZBED6CL |
| 100287144_at | 6.5553 | 0.0028005 | 2.5528 | 0.0069342 | USP17L10 |
| 28731_at | 6.5553 | 0.0028006 | 2.5527 | 0.0069342 | TRAJ24 |
| 51768_at | 6.5508 | 0.0028077 | 2.5516 | 0.0069484 | TM7SF3 |
| 222235_at | 6.5497 | 0.0028094 | 2.5514 | 0.006949 | FBXL13 |
| 29028_at | -6.5483 | 0.0028116 | 2.5511 | 0.0069511 | ATAD2 |
| 151760_at | -6.5417 | 0.0028222 | 2.5494 | 0.0069738 | LOC151760 |
| 4700_at | 6.5358 | 0.0028317 | 2.548 | 0.0069922 | NDUFA6 |
| 118932_at | -6.5353 | 0.0028324 | 2.5478 | 0.0069922 | ANKRD22 |
| 170692_at | -6.5332 | 0.0028359 | 2.5473 | 0.0069975 | ADAMTS18 |
| 163351_at | -6.5304 | 0.0028403 | 2.5466 | 0.0070038 | GBP6 |
| 5660_at | 6.5298 | 0.0028413 | 2.5465 | 0.0070038 | PSAP |
| 4953_at | 6.528 | 0.0028443 | 2.546 | 0.0070071 | ODC1 |
| 55226_at | 6.5273 | 0.0028455 | 2.5458 | 0.0070071 | NAT10 |
| 8848_at | 6.5242 | 0.0028505 | 2.5451 | 0.0070138 | TSC22D1 |
| 10494_at | 6.5239 | 0.002851 | 2.545 | 0.0070138 | STK25 |
| 26782_at | -6.5226 | 0.0028531 | 2.5447 | 0.0070155 | SNORA66 |
| 7318_at | -6.5212 | 0.0028553 | 2.5444 | 0.0070174 | UBA7 |
| 387890_at | 6.5163 | 0.0028632 | 2.5431 | 0.0070312 | TMEM233 |
| 84803_at | 6.5161 | 0.0028637 | 2.5431 | 0.0070312 | AGPAT9 |
| 118429_at | -6.5143 | 0.0028666 | 2.5426 | 0.0070349 | ANTXR2 |
| 10157_at | -6.5076 | 0.0028776 | 2.541 | 0.0070551 | AASS |
| 1454_at | -6.5075 | 0.0028776 | 2.541 | 0.0070551 | CSNK1E |
| 80256_at | 6.5026 | 0.0028857 | 2.5397 | 0.0070715 | FAM214B |
| 1030_at | -6.5011 | 0.0028882 | 2.5394 | 0.007074 | CDKN2B |
| 28378_at | -6.4996 | 0.0028907 | 2.539 | 0.0070767 | IGHV7-81 |
| 81849_at | -6.4959 | 0.0028967 | 2.5381 | 0.0070838 | ST6GALNAC5 |
| 80345_at | -6.4954 | 0.0028976 | 2.538 | 0.0070838 | ZSCAN16 |
| 2043_at | 6.4952 | 0.0028979 | 2.5379 | 0.0070838 | EPHA4 |
| 6925_at | -6.4929 | 0.0029018 | 2.5373 | 0.00709 | TCF4 |
| 26025_at | 6.4904 | 0.0029058 | 2.5367 | 0.0070963 | PCDHGA12 |
| 254225_at | -6.4887 | 0.0029087 | 2.5363 | 0.0070999 | RNF169 |
| 4292_at | 6.4869 | 0.0029118 | 2.5358 | 0.0071031 | MLH1 |
| 10134_at | 6.4862 | 0.0029129 | 2.5357 | 0.0071031 | BCAP31 |
| 7068_at | 6.4786 | 0.0029256 | 2.5338 | 0.0071261 | THRB |
| 55294_at | -6.4783 | 0.002926 | 2.5337 | 0.0071261 | FBXW7 |
| 1875_at | -6.4771 | 0.002928 | 2.5334 | 0.0071261 | E2F5 |
| 9545_at | -6.4765 | 0.0029291 | 2.5333 | 0.0071261 | RAB3D |
| 207063_at | -6.4763 | 0.0029294 | 2.5332 | 0.0071261 | DHRSX |
| 4881_at | 6.4731 | 0.0029348 | 2.5324 | 0.0071302 | NPR1 |
| 9848_at | 6.473 | 0.0029349 | 2.5324 | 0.0071302 | MFAP3L |
| 6581_at | 6.4724 | 0.002936 | 2.5322 | 0.0071302 | SLC22A3 |
| 7448_at | 6.4719 | 0.0029368 | 2.5321 | 0.0071302 | VTN |
| 6331_at | -6.4643 | 0.0029497 | 2.5302 | 0.0071581 | SCN5A |
| 55802_at | -6.4625 | 0.0029527 | 2.5298 | 0.0071619 | DCP1A |
| 200150_at | -6.4613 | 0.0029547 | 2.5295 | 0.0071632 | PLD5 |
| 55841_at | -6.4598 | 0.0029572 | 2.5291 | 0.0071658 | WWC3 |
| 84284_at | 6.454 | 0.0029672 | 2.5276 | 0.0071866 | NTPCR |
| 5602_at | 6.4503 | 0.0029735 | 2.5267 | 0.0071984 | MAPK10 |
| 101928075_at | -6.4445 | 0.0029833 | 2.5253 | 0.0072177 | LOC101928075 |
| 1801_at | 6.4439 | 0.0029844 | 2.5251 | 0.0072177 | DPH1 |
| 7750_at | -6.4418 | 0.002988 | 2.5246 | 0.007223 | ZMYM2 |
| 715_at | 6.4368 | 0.0029966 | 2.5234 | 0.0072357 | C1R |
| 9670_at | 6.4358 | 0.0029983 | 2.5231 | 0.0072357 | IPO13 |
| 283450_at | -6.435 | 0.0029998 | 2.5229 | 0.0072357 | HECTD4 |
| 9331_at | -6.4343 | 0.003001 | 2.5227 | 0.0072357 | B4GALT6 |
| 26503_at | -6.4331 | 0.0030031 | 2.5224 | 0.0072357 | SLC17A5 |
| 57162_at | -6.433 | 0.0030033 | 2.5224 | 0.0072357 | PELI1 |
| 2589_at | 6.4329 | 0.0030034 | 2.5224 | 0.0072357 | GALNT1 |
| 1349_at | 6.4279 | 0.0030121 | 2.5211 | 0.0072467 | COX7B |
| 3672_at | 6.4275 | 0.0030126 | 2.5211 | 0.0072467 | ITGA1 |
| 101928461_at | -6.4272 | 0.0030132 | 2.521 | 0.0072467 | LOC101928461 |
| 100507459_at | 6.4269 | 0.0030138 | 2.5209 | 0.0072467 | NRSN2-AS1 |
| 285600_at | 6.4253 | 0.0030165 | 2.5205 | 0.0072499 | KIAA0825 |
| 57402_at | -6.4238 | 0.0030191 | 2.5201 | 0.0072525 | S100A14 |
| 56548_at | -6.411 | 0.0030416 | 2.5169 | 0.007303 | CHST7 |
| 11260_at | -6.4081 | 0.0030466 | 2.5162 | 0.0073115 | XPOT |
| 7754_at | -6.4073 | 0.0030481 | 2.516 | 0.0073115 | ZNF204P |
| 3174_at | 6.4041 | 0.0030537 | 2.5152 | 0.0073191 | HNF4G |
| 102723834_at | 6.4039 | 0.0030541 | 2.5151 | 0.0073191 | LOC102723834 |
| 3280_at | -6.4016 | 0.0030582 | 2.5145 | 0.0073253 | HES1 |
| 55157_at | 6.4001 | 0.0030608 | 2.5142 | 0.0073274 | DARS2 |
| 84725_at | -6.3994 | 0.003062 | 2.514 | 0.0073274 | PLEKHA8 |
| 64968_at | 6.3976 | 0.0030651 | 2.5136 | 0.0073314 | MRPS6 |
| 94104_at | -6.3946 | 0.0030706 | 2.5128 | 0.0073408 | PAXBP1 |
| 84281_at | 6.3922 | 0.0030748 | 2.5122 | 0.0073476 | C2orf88 |
| 51071_at | 6.3861 | 0.0030858 | 2.5106 | 0.0073702 | DERA |
| 1465_at | -6.3737 | 0.0031079 | 2.5075 | 0.0074195 | CSRP1 |
| 51727_at | -6.3667 | 0.0031207 | 2.5057 | 0.0074465 | CMPK1 |
| 317760_at | -6.3579 | 0.0031366 | 2.5035 | 0.0074807 | ADAM20P1 |
| 23551_at | 6.3568 | 0.0031386 | 2.5033 | 0.0074821 | RASD2 |
| 57161_at | -6.3487 | 0.0031535 | 2.5012 | 0.0075058 | PELI2 |
| 51191_at | -6.3482 | 0.0031544 | 2.5011 | 0.0075058 | HERC5 |
| 9455_at | 6.3475 | 0.0031556 | 2.5009 | 0.0075058 | HOMER2 |
| 9896_at | -6.3467 | 0.0031571 | 2.5007 | 0.0075058 | FIG4 |
| 143458_at | 6.3467 | 0.0031571 | 2.5007 | 0.0075058 | LDLRAD3 |
| 401585_at | -6.3465 | 0.0031576 | 2.5006 | 0.0075058 | LOC401585 |
| 79861_at | 6.3441 | 0.003162 | 2.5 | 0.0075127 | TUBAL3 |
| 101928896_at | -6.3418 | 0.0031661 | 2.4995 | 0.0075156 | LOC101928896 |
| 4507_at | -6.3418 | 0.0031662 | 2.4995 | 0.0075156 | MTAP |
| 122060_at | -6.3409 | 0.0031678 | 2.4992 | 0.0075159 | SLAIN1 |
| 10741_at | 6.3352 | 0.0031785 | 2.4978 | 0.0075375 | RBBP9 |
| 65109_at | -6.3341 | 0.0031803 | 2.4975 | 0.0075384 | UPF3B |
| 54865_at | 6.3312 | 0.0031858 | 2.4968 | 0.0075479 | GPATCH4 |
| 8263_at | 6.3288 | 0.0031904 | 2.4962 | 0.0075534 | F8A1 |
| 6515_at | 6.3283 | 0.0031912 | 2.496 | 0.0075534 | SLC2A3 |
| 767580_at | -6.326 | 0.0031955 | 2.4955 | 0.0075566 | SNORD114-4 |
| 554282_at | -6.326 | 0.0031956 | 2.4955 | 0.0075566 | FAM72C |
| 51150_at | 6.3237 | 0.0031998 | 2.4949 | 0.007563 | SDF4 |
| 204_at | 6.3197 | 0.0032072 | 2.4939 | 0.007577 | AK2 |
| 4851_at | -6.3164 | 0.0032134 | 2.493 | 0.007588 | NOTCH1 |
| 8655_at | 6.3151 | 0.0032159 | 2.4927 | 0.0075904 | DYNLL1 |
| 3429_at | -6.313 | 0.0032198 | 2.4922 | 0.0075933 | IFI27 |
| 7132_at | 6.3128 | 0.0032202 | 2.4921 | 0.0075933 | TNFRSF1A |
| 5518_at | 6.3098 | 0.003226 | 2.4913 | 0.0076034 | PPP2R1A |
| 171586_at | -6.3017 | 0.0032412 | 2.4893 | 0.0076357 | ABHD3 |
| 10682_at | -6.2928 | 0.0032582 | 2.487 | 0.007672 | EBP |
| 415_at | 6.2906 | 0.0032624 | 2.4865 | 0.0076782 | ARSE |
| 3841_at | -6.2882 | 0.003267 | 2.4859 | 0.0076855 | KPNA5 |
| 7552_at | -6.2847 | 0.0032737 | 2.485 | 0.0076977 | ZNF711 |
| 8802_at | 6.2816 | 0.0032798 | 2.4841 | 0.0077053 | SUCLG1 |
| 51027_at | 6.2814 | 0.0032801 | 2.4841 | 0.0077053 | BOLA1 |
| 10039_at | 6.2774 | 0.0032879 | 2.4831 | 0.0077179 | PARP3 |
| 51761_at | -6.2771 | 0.0032885 | 2.483 | 0.0077179 | ATP8A2 |
| 7112_at | -6.2757 | 0.0032912 | 2.4826 | 0.0077206 | TMPO |
| 100874323_at | 6.2734 | 0.0032956 | 2.4821 | 0.0077273 | HOXA10-AS |
| 23218_at | -6.2667 | 0.0033086 | 2.4804 | 0.0077541 | NBEAL2 |
| 143686_at | 6.2623 | 0.0033172 | 2.4792 | 0.0077707 | SESN3 |
| 5934_at | -6.2568 | 0.0033279 | 2.4778 | 0.0077898 | RBL2 |
| 80017_at | 6.2558 | 0.0033299 | 2.4776 | 0.0077898 | C14orf159 |
| 26610_at | 6.2558 | 0.00333 | 2.4776 | 0.0077898 | ELP4 |
| 1437_at | -6.2511 | 0.0033391 | 2.4764 | 0.0078041 | CSF2 |
| 794_at | -6.2511 | 0.0033393 | 2.4764 | 0.0078041 | CALB2 |
| 256130_at | 6.2466 | 0.003348 | 2.4752 | 0.0078209 | TMEM196 |
| 2224_at | -6.2435 | 0.0033542 | 2.4744 | 0.0078287 | FDPS |
| 81037_at | 6.2434 | 0.0033545 | 2.4744 | 0.0078287 | CLPTM1L |
| 4680_at | -6.2371 | 0.0033669 | 2.4728 | 0.0078541 | CEACAM6 |
| 55315_at | 6.2296 | 0.0033819 | 2.4708 | 0.0078822 | SLC29A3 |
| 4898_at | -6.2295 | 0.0033822 | 2.4708 | 0.0078822 | NRD1 |
| 5582_at | -6.2262 | 0.0033887 | 2.47 | 0.0078938 | PRKCG |
| 10318_at | -6.2224 | 0.0033964 | 2.469 | 0.0079052 | TNIP1 |
| 79054_at | 6.2213 | 0.0033985 | 2.4687 | 0.0079052 | TRPM8 |
| 340348_at | 6.2208 | 0.0033996 | 2.4686 | 0.0079052 | TSPAN33 |
| 4092_at | 6.2206 | 0.0033999 | 2.4685 | 0.0079052 | SMAD7 |
| 157869_at | 6.2135 | 0.0034144 | 2.4667 | 0.0079352 | SBSPON |
| 4609_at | 6.2102 | 0.003421 | 2.4659 | 0.0079468 | MYC |
| 40_at | 6.2073 | 0.0034268 | 2.4651 | 0.0079567 | ASIC2 |
| 55757_at | -6.2061 | 0.0034293 | 2.4648 | 0.0079587 | UGGT2 |
| 643911_at | -6.2032 | 0.0034353 | 2.464 | 0.007969 | CRNDE |
| 101928123_at | -6.1966 | 0.0034488 | 2.4623 | 0.0079966 | LOC101928123 |
| 100874032_at | 6.195 | 0.0034521 | 2.4619 | 0.0080004 | PRRT3-AS1 |
| 64750_at | -6.1871 | 0.0034683 | 2.4599 | 0.0080342 | SMURF2 |
| 55191_at | 6.1824 | 0.0034781 | 2.4587 | 0.0080529 | NADSYN1 |
| 9344_at | 6.1817 | 0.0034796 | 2.4585 | 0.0080529 | TAOK2 |
| 80196_at | 6.1755 | 0.0034923 | 2.4569 | 0.0080787 | RNF34 |
| 8200_at | 6.1717 | 0.0035003 | 2.4559 | 0.0080934 | GDF5 |
| 83481_at | -6.169 | 0.0035059 | 2.4552 | 0.0081027 | EPPK1 |
| 10273_at | 6.167 | 0.0035101 | 2.4547 | 0.0081085 | STUB1 |
| 9534_at | -6.1654 | 0.0035134 | 2.4543 | 0.0081125 | ZNF254 |
| 26235_at | 6.1569 | 0.0035314 | 2.4521 | 0.0081503 | FBXL4 |
| 147179_at | 6.1515 | 0.0035427 | 2.4507 | 0.0081711 | WIPF2 |
| 57643_at | 6.1509 | 0.0035441 | 2.4505 | 0.0081711 | ZSWIM5 |
| 50486_at | -6.1503 | 0.0035453 | 2.4503 | 0.0081711 | G0S2 |
| 134549_at | -6.1471 | 0.003552 | 2.4495 | 0.0081827 | SHROOM1 |
| 55654_at | 6.1446 | 0.0035574 | 2.4489 | 0.0081913 | TMEM127 |
| 347273_at | -6.1412 | 0.0035646 | 2.448 | 0.0082041 | MURC |
| 83604_at | 6.14 | 0.0035673 | 2.4477 | 0.0082065 | TMEM47 |
| 55034_at | -6.1313 | 0.0035858 | 2.4454 | 0.0082455 | MOCOS |
| 51608_at | -6.1288 | 0.0035913 | 2.4447 | 0.0082542 | GET4 |
| 89932_at | 6.1275 | 0.0035941 | 2.4444 | 0.0082568 | PAPLN |
| 10488_at | 6.124 | 0.0036015 | 2.4435 | 0.0082696 | CREB3 |
| 57018_at | -6.1234 | 0.003603 | 2.4433 | 0.0082696 | CCNL1 |
| 4237_at | -6.1226 | 0.0036046 | 2.4431 | 0.0082696 | MFAP2 |
| 56912_at | 6.12 | 0.0036103 | 2.4425 | 0.0082788 | IFT46 |
| 113675_at | 6.1115 | 0.0036288 | 2.4402 | 0.0083174 | SDSL |
| 80149_at | -6.1102 | 0.0036316 | 2.4399 | 0.00832 | ZC3H12A |
| 55686_at | -6.1075 | 0.0036375 | 2.4392 | 0.0083297 | MREG |
| 9510_at | 6.1053 | 0.0036423 | 2.4386 | 0.0083345 | ADAMTS1 |
| 79659_at | 6.1051 | 0.0036429 | 2.4386 | 0.0083345 | DYNC2H1 |
| 54534_at | 6.1007 | 0.0036526 | 2.4374 | 0.0083528 | MRPL50 |
| 55589_at | 6.0962 | 0.0036624 | 2.4362 | 0.0083678 | BMP2K |
| 285362_at | 6.0962 | 0.0036625 | 2.4362 | 0.0083678 | SUMF1 |
| 129642_at | -6.0923 | 0.0036711 | 2.4352 | 0.0083836 | MBOAT2 |
| 253650_at | 6.0901 | 0.0036759 | 2.4346 | 0.0083878 | ANKRD18A |
| 84899_at | 6.0899 | 0.0036762 | 2.4346 | 0.0083878 | TMTC4 |
| 9761_at | 6.087 | 0.0036827 | 2.4338 | 0.0083986 | MLEC |
| 9699_at | 6.0847 | 0.0036878 | 2.4332 | 0.0084065 | RIMS2 |
| 6909_at | 6.0805 | 0.0036971 | 2.4321 | 0.0084239 | TBX2 |
| 9638_at | -6.0747 | 0.0037103 | 2.4306 | 0.0084499 | FEZ1 |
| 84929_at | 6.0736 | 0.0037126 | 2.4303 | 0.0084515 | FIBCD1 |
| 55803_at | 6.0717 | 0.0037169 | 2.4298 | 0.0084561 | ADAP2 |
| 57406_at | 6.0708 | 0.003719 | 2.4296 | 0.0084561 | ABHD6 |
| 6988_at | 6.0705 | 0.0037198 | 2.4295 | 0.0084561 | TCTA |
| 2395_at | 6.0652 | 0.0037315 | 2.4281 | 0.0084791 | FXN |
| 81493_at | 6.0619 | 0.003739 | 2.4272 | 0.0084921 | SYNC |
| 81831_at | 6.0606 | 0.003742 | 2.4269 | 0.0084952 | NETO2 |
| 60682_at | 6.0595 | 0.0037445 | 2.4266 | 0.0084968 | SMAP1 |
| 253827_at | 6.0548 | 0.0037551 | 2.4254 | 0.0085172 | MSRB3 |
| 634_at | -6.0509 | 0.003764 | 2.4243 | 0.0085335 | CEACAM1 |
| 10643_at | -6.0494 | 0.0037675 | 2.4239 | 0.008535 | IGF2BP3 |
| 10721_at | -6.0485 | 0.0037695 | 2.4237 | 0.008535 | POLQ |
| 22836_at | -6.0484 | 0.0037698 | 2.4237 | 0.008535 | RHOBTB3 |
| 54988_at | 6.0468 | 0.0037736 | 2.4232 | 0.0085397 | ACSM5 |
| 22925_at | 6.0445 | 0.0037788 | 2.4226 | 0.0085477 | PLA2R1 |
| 7203_at | 6.04 | 0.0037891 | 2.4215 | 0.0085671 | CCT3 |
| 9824_at | -6.0357 | 0.0037991 | 2.4203 | 0.0085857 | ARHGAP11A |
| 7091_at | -6.0348 | 0.003801 | 2.4201 | 0.0085862 | TLE4 |
| 5919_at | 6.0323 | 0.0038068 | 2.4194 | 0.0085954 | RARRES2 |
| 83699_at | -6.0311 | 0.0038097 | 2.4191 | 0.008598 | SH3BGRL2 |
| 10813_at | 6.0292 | 0.003814 | 2.4186 | 0.0086039 | UTP14A |
| 6242_at | 6.0258 | 0.0038219 | 2.4177 | 0.0086141 | RTKN |
| 728262_at | -6.0258 | 0.003822 | 2.4177 | 0.0086141 | FAM157A |
| 9767_at | 6.0247 | 0.0038244 | 2.4174 | 0.0086157 | JADE3 |
| 100128750_at | 6.0202 | 0.003835 | 2.4162 | 0.0086247 | RBPMS-AS1 |
| 7167_at | 6.0201 | 0.0038353 | 2.4162 | 0.0086247 | TPI1 |
| 55437_at | 6.0199 | 0.0038357 | 2.4162 | 0.0086247 | STRADB |
| 353219_at | 6.0194 | 0.0038369 | 2.416 | 0.0086247 | KAAG1 |
| 2184_at | 6.019 | 0.0038378 | 2.4159 | 0.0086247 | FAH |
| 85027_at | 6.0186 | 0.0038388 | 2.4158 | 0.0086247 | SMIM3 |
| 9710_at | -6.0158 | 0.0038453 | 2.4151 | 0.0086355 | KIAA0355 |
| 11329_at | 6.0127 | 0.0038525 | 2.4143 | 0.0086411 | STK38 |
| 11321_at | 6.0127 | 0.0038526 | 2.4142 | 0.0086411 | GPN1 |
| 80176_at | 6.0125 | 0.003853 | 2.4142 | 0.0086411 | SPSB1 |
| 285464_at | -6.0087 | 0.003862 | 2.4132 | 0.0086575 | CRIPAK |
| 11338_at | 6.0071 | 0.0038656 | 2.4128 | 0.0086617 | U2AF2 |
| 3717_at | -6.0051 | 0.0038705 | 2.4122 | 0.0086687 | JAK2 |
| 90416_at | 5.9944 | 0.003896 | 2.4094 | 0.0087219 | C15orf57 |
| 145624_at | -5.9931 | 0.0038989 | 2.4091 | 0.0087244 | PWAR1 |
| 286122_at | -5.9917 | 0.0039022 | 2.4087 | 0.008728 | C8orf31 |
| 84815_at | -5.9854 | 0.0039173 | 2.407 | 0.0087578 | MGC12916 |
| 1795_at | -5.9846 | 0.0039192 | 2.4068 | 0.0087581 | DOCK3 |
| 595100_at | -5.9828 | 0.0039237 | 2.4063 | 0.008761 | SNORD18C |
| 6913_at | 5.9827 | 0.0039239 | 2.4063 | 0.008761 | TBX15 |
| 1785_at | 5.9793 | 0.003932 | 2.4054 | 0.008775 | DNM2 |
| 5287_at | -5.9765 | 0.0039387 | 2.4046 | 0.0087858 | PIK3C2B |
| 8570_at | 5.9758 | 0.0039403 | 2.4045 | 0.0087858 | KHSRP |
| 79152_at | -5.9719 | 0.0039498 | 2.4034 | 0.008803 | FA2H |
| 23037_at | -5.9686 | 0.0039579 | 2.4025 | 0.0088136 | PDZD2 |
| 80024_at | -5.9685 | 0.0039581 | 2.4025 | 0.0088136 | SLC8B1 |
| 130271_at | -5.9622 | 0.0039734 | 2.4008 | 0.0088437 | PLEKHH2 |
| 23133_at | -5.9612 | 0.0039759 | 2.4006 | 0.0088453 | PHF8 |
| 83439_at | -5.9586 | 0.0039824 | 2.3999 | 0.0088558 | TCF7L1 |
| 54664_at | -5.9548 | 0.0039915 | 2.3989 | 0.0088722 | TMEM106B |
| 10038_at | -5.9484 | 0.0040072 | 2.3972 | 0.0089032 | PARP2 |
| 55893_at | 5.9475 | 0.0040095 | 2.3969 | 0.0089043 | ZNF395 |
| 9514_at | 5.9456 | 0.0040143 | 2.3964 | 0.0089103 | GAL3ST1 |
| 55248_at | -5.945 | 0.0040158 | 2.3962 | 0.0089103 | TMEM206 |
| 22797_at | 5.9423 | 0.0040224 | 2.3955 | 0.008921 | TFEC |
| 201232_at | -5.94 | 0.004028 | 2.3949 | 0.0089295 | SLC16A13 |
| 64577_at | 5.9378 | 0.0040335 | 2.3943 | 0.0089378 | ALDH8A1 |
| 83539_at | -5.9364 | 0.0040369 | 2.3939 | 0.0089413 | CHST9 |
| 192286_at | 5.9349 | 0.0040409 | 2.3935 | 0.0089462 | HIGD2A |
| 6300_at | 5.9336 | 0.004044 | 2.3932 | 0.0089491 | MAPK12 |
| 390649_at | 5.9298 | 0.0040534 | 2.3922 | 0.0089621 | OR4F15 |
| 60485_at | -5.9298 | 0.0040535 | 2.3922 | 0.0089621 | SAV1 |
| 81889_at | 5.9261 | 0.0040627 | 2.3912 | 0.0089785 | FAHD1 |
| 102723766_at | -5.9243 | 0.0040672 | 2.3907 | 0.0089845 | LOC102723766 |
| 1595_at | -5.9227 | 0.0040713 | 2.3903 | 0.0089895 | CYP51A1 |
| 1365_at | -5.9171 | 0.0040855 | 2.3888 | 0.0090131 | CLDN3 |
| 285966_at | 5.9171 | 0.0040855 | 2.3888 | 0.0090131 | FAM115C |
| 3205_at | 5.9139 | 0.0040934 | 2.3879 | 0.0090265 | HOXA9 |
| 57565_at | 5.9126 | 0.0040967 | 2.3876 | 0.0090298 | KLHL14 |
| 84804_at | -5.9113 | 0.0041002 | 2.3872 | 0.0090334 | MFSD9 |
| 57684_at | 5.9088 | 0.0041065 | 2.3865 | 0.0090434 | ZBTB26 |
| 27233_at | -5.9059 | 0.0041138 | 2.3858 | 0.0090554 | SULT1C4 |
| 150468_at | -5.9009 | 0.0041267 | 2.3844 | 0.0090798 | CKAP2L |
| 94241_at | -5.8987 | 0.0041321 | 2.3838 | 0.0090877 | TP53INP1 |
| 51379_at | -5.8871 | 0.004162 | 2.3807 | 0.0091481 | CRLF3 |
| 127845_at | -5.8866 | 0.0041632 | 2.3806 | 0.0091481 | GOLT1A |
| 4050_at | -5.8852 | 0.004167 | 2.3802 | 0.0091524 | LTB |
| 79026_at | 5.8833 | 0.0041719 | 2.3797 | 0.0091569 | AHNAK |
| 26520_at | 5.883 | 0.0041727 | 2.3796 | 0.0091569 | TIMM9 |
| 27063_at | -5.8802 | 0.00418 | 2.3788 | 0.0091688 | ANKRD1 |
| 6038_at | 5.8773 | 0.0041874 | 2.3781 | 0.0091811 | RNASE4 |
| 5598_at | 5.8743 | 0.0041952 | 2.3772 | 0.0091941 | MAPK7 |
| 1948_at | -5.8698 | 0.004207 | 2.376 | 0.0092136 | EFNB2 |
| 9633_at | -5.8695 | 0.0042078 | 2.3759 | 0.0092136 | MTL5 |
| 93010_at | -5.8633 | 0.004224 | 2.3743 | 0.0092429 | B3GNT7 |
| 25819_at | 5.863 | 0.0042248 | 2.3742 | 0.0092429 | CCRN4L |
| 126353_at | -5.8622 | 0.0042271 | 2.374 | 0.0092437 | MISP |
| 9170_at | -5.8576 | 0.004239 | 2.3727 | 0.0092658 | LPAR2 |
| 57187_at | -5.8533 | 0.0042505 | 2.3716 | 0.009283 | THOC2 |
| 192669_at | -5.8533 | 0.0042506 | 2.3716 | 0.009283 | AGO3 |
| 84376_at | -5.8445 | 0.0042738 | 2.3692 | 0.0093295 | HOOK3 |
| 89927_at | 5.8389 | 0.0042888 | 2.3677 | 0.0093582 | C16orf45 |
| 115019_at | -5.8373 | 0.0042931 | 2.3672 | 0.0093634 | SLC26A9 |
| 51530_at | 5.8295 | 0.0043141 | 2.3651 | 0.0094053 | ZC3HC1 |
| 154313_at | 5.8242 | 0.0043285 | 2.3637 | 0.0094294 | C6orf165 |
| 55735_at | 5.824 | 0.004329 | 2.3636 | 0.0094294 | DNAJC11 |
| 143689_at | -5.817 | 0.0043481 | 2.3617 | 0.009467 | PIWIL4 |
| 80018_at | -5.8137 | 0.004357 | 2.3608 | 0.0094814 | NAA25 |
| 11113_at | -5.8132 | 0.0043585 | 2.3607 | 0.0094814 | CIT |
| 54918_at | -5.7999 | 0.004395 | 2.357 | 0.0095565 | CMTM6 |
| 79590_at | 5.7985 | 0.0043987 | 2.3567 | 0.0095574 | MRPL24 |
| 100289019_at | -5.7984 | 0.0043992 | 2.3566 | 0.0095574 | SLC25A25-AS1 |
| 63898_at | -5.7939 | 0.0044117 | 2.3554 | 0.0095767 | SH2D4A |
| 54904_at | -5.7938 | 0.0044119 | 2.3554 | 0.0095767 | WHSC1L1 |
| 699_at | -5.7889 | 0.0044254 | 2.3541 | 0.0096017 | BUB1 |
| 54797_at | 5.7857 | 0.0044343 | 2.3532 | 0.009617 | MED18 |
| 1874_at | 5.7798 | 0.0044509 | 2.3516 | 0.0096488 | E2F4 |
| 8536_at | 5.7787 | 0.004454 | 2.3513 | 0.0096512 | CAMK1 |
| 90799_at | -5.7763 | 0.0044607 | 2.3506 | 0.0096617 | CEP95 |
| 80306_at | -5.7745 | 0.0044657 | 2.3501 | 0.009667 | MED28 |
| 1540_at | -5.774 | 0.0044673 | 2.35 | 0.009667 | CYLD |
| 5284_at | -5.7734 | 0.004469 | 2.3498 | 0.009667 | PIGR |
| 26040_at | 5.7706 | 0.0044767 | 2.349 | 0.0096795 | SETBP1 |
| 10263_at | 5.7697 | 0.0044793 | 2.3488 | 0.0096809 | CDK2AP2 |
| 970_at | 5.763 | 0.0044984 | 2.3469 | 0.009718 | CD70 |
| 51678_at | 5.7557 | 0.0045191 | 2.345 | 0.0097585 | MPP6 |
| 11267_at | 5.7504 | 0.0045342 | 2.3435 | 0.0097868 | SNF8 |
| 79820_at | -5.7493 | 0.0045373 | 2.3432 | 0.0097895 | CATSPERB |
| 5584_at | -5.7482 | 0.0045407 | 2.3429 | 0.0097924 | PRKCI |
| 57584_at | -5.7457 | 0.0045478 | 2.3422 | 0.0098036 | ARHGAP21 |
| 26798_at | -5.7343 | 0.0045809 | 2.3391 | 0.0098695 | SNORD51 |
| 3241_at | 5.7338 | 0.0045823 | 2.3389 | 0.0098695 | HPCAL1 |
| 54014_at | -5.7321 | 0.0045872 | 2.3385 | 0.0098758 | BRWD1 |
| 130013_at | 5.7289 | 0.0045966 | 2.3376 | 0.0098825 | ACMSD |
| 55501_at | 5.7288 | 0.0045968 | 2.3375 | 0.0098825 | CHST12 |
| 10195_at | 5.7282 | 0.0045985 | 2.3374 | 0.0098825 | ALG3 |
| 693189_at | -5.7276 | 0.0046001 | 2.3372 | 0.0098825 | MIR604 |
| 84886_at | -5.7276 | 0.0046002 | 2.3372 | 0.0098825 | C1orf198 |
| 143888_at | 5.7252 | 0.0046073 | 2.3366 | 0.0098935 | KDELC2 |
| 64849_at | 5.721 | 0.0046194 | 2.3354 | 0.0099153 | SLC13A3 |
| 78996_at | 5.7201 | 0.0046222 | 2.3351 | 0.009917 | C7orf49 |
| 22936_at | 5.7137 | 0.0046412 | 2.3334 | 0.0099533 | ELL2 |
| 84440_at | -5.7083 | 0.004657 | 2.3319 | 0.0099789 | RAB11FIP4 |
| 83743_at | 5.7083 | 0.0046571 | 2.3319 | 0.0099789 | GRWD1 |
| 678_at | 5.707 | 0.004661 | 2.3315 | 0.0099802 | ZFP36L2 |
| 55680_at | -5.7067 | 0.0046617 | 2.3315 | 0.0099802 | RUFY2 |
| 23310_at | -5.704 | 0.0046699 | 2.3307 | 0.0099935 | NCAPD3 |
| 192683_at | 5.7009 | 0.004679 | 2.3298 | 0.010009 | SCAMP5 |
| 10203_at | 5.6995 | 0.0046832 | 2.3295 | 0.010013 | CALCRL |
| 728780_at | 5.6975 | 0.0046892 | 2.3289 | 0.010022 | ANKDD1B |
| 9859_at | -5.6962 | 0.0046932 | 2.3285 | 0.010025 | CEP170 |
| 96764_at | -5.6957 | 0.0046946 | 2.3284 | 0.010025 | TGS1 |
| 26512_at | -5.6938 | 0.0047002 | 2.3279 | 0.010033 | INTS6 |
| 6574_at | 5.6906 | 0.00471 | 2.327 | 0.010047 | SLC20A1 |
| 399716_at | -5.6903 | 0.0047109 | 2.3269 | 0.010047 | DKFZp667F0711 |
| 256356_at | -5.6893 | 0.0047139 | 2.3266 | 0.010049 | GK5 |
| 1947_at | -5.6869 | 0.0047212 | 2.3259 | 0.01006 | EFNB1 |
| 65981_at | -5.6821 | 0.0047356 | 2.3246 | 0.010087 | CAPRIN2 |
| 57619_at | -5.681 | 0.0047389 | 2.3243 | 0.010089 | SHROOM3 |
| 3669_at | -5.6804 | 0.0047405 | 2.3242 | 0.010089 | ISG20 |
| 8291_at | 5.6783 | 0.004747 | 2.3236 | 0.010098 | DYSF |
| 259282_at | -5.6751 | 0.0047567 | 2.3227 | 0.010114 | BOD1L1 |
| 134147_at | 5.6738 | 0.0047606 | 2.3223 | 0.010118 | CMBL |
| 4853_at | 5.673 | 0.0047633 | 2.3221 | 0.01012 | NOTCH2 |
| 54578_at | 5.6698 | 0.004773 | 2.3212 | 0.010136 | UGT1A6 |
| 27258_at | 5.6691 | 0.004775 | 2.321 | 0.010136 | LSM3 |
| 112752_at | 5.6684 | 0.0047772 | 2.3208 | 0.010136 | IFT43 |
| 9422_at | -5.6636 | 0.0047919 | 2.3195 | 0.010163 | ZNF264 |
| 55640_at | 5.662 | 0.0047969 | 2.319 | 0.01017 | FLVCR2 |
| 55765_at | -5.6596 | 0.0048042 | 2.3184 | 0.010181 | C1orf106 |
| 131405_at | 5.6541 | 0.0048212 | 2.3168 | 0.010212 | TRIM71 |
| 6433_at | -5.6523 | 0.0048266 | 2.3164 | 0.01022 | SFSWAP |
| 80150_at | 5.6515 | 0.0048293 | 2.3161 | 0.010221 | ASRGL1 |
| 1643_at | -5.6483 | 0.0048392 | 2.3152 | 0.010237 | DDB2 |
| 25870_at | 5.6477 | 0.004841 | 2.3151 | 0.010237 | SUMF2 |
| 9039_at | -5.6432 | 0.0048549 | 2.3138 | 0.01026 | UBA3 |
| 55814_at | -5.6429 | 0.0048559 | 2.3137 | 0.01026 | BDP1 |
| 114791_at | 5.6399 | 0.0048654 | 2.3129 | 0.010276 | TUBGCP5 |
| 2673_at | -5.6351 | 0.0048804 | 2.3115 | 0.010303 | GFPT1 |
| 161582_at | 5.6306 | 0.0048944 | 2.3103 | 0.010328 | DYX1C1 |
| 5586_at | -5.6286 | 0.0049008 | 2.3097 | 0.010337 | PKN2 |
| 25987_at | 5.627 | 0.0049059 | 2.3093 | 0.010337 | TSKU |
| 3993_at | -5.6264 | 0.0049079 | 2.3091 | 0.010337 | LLGL2 |
| 23371_at | 5.6263 | 0.0049083 | 2.3091 | 0.010337 | TENC1 |
| 129787_at | 5.6254 | 0.0049109 | 2.3088 | 0.010337 | TMEM18 |
| 144501_at | -5.6254 | 0.004911 | 2.3088 | 0.010337 | KRT80 |
| 747_at | -5.6233 | 0.0049177 | 2.3082 | 0.010343 | DAGLA |
| 3224_at | 5.6231 | 0.0049182 | 2.3082 | 0.010343 | HOXC8 |
| 1203_at | 5.62 | 0.0049282 | 2.3073 | 0.01036 | CLN5 |
| 23122_at | -5.6126 | 0.0049517 | 2.3052 | 0.010404 | CLASP2 |
| 51696_at | -5.6121 | 0.0049532 | 2.3051 | 0.010404 | HECA |
| 6873_at | -5.6101 | 0.0049595 | 2.3046 | 0.010413 | TAF2 |
| 50999_at | 5.6093 | 0.0049622 | 2.3043 | 0.010414 | TMED5 |
| 93594_at | -5.6069 | 0.0049699 | 2.3037 | 0.010426 | TBC1D31 |
| 400533_at | 5.6052 | 0.0049754 | 2.3032 | 0.010431 | FLJ26245 |
| 5067_at | -5.6048 | 0.0049766 | 2.3031 | 0.010431 | CNTN3 |
| 5031_at | 5.6019 | 0.0049861 | 2.3022 | 0.010446 | P2RY6 |
| 7104_at | 5.5965 | 0.0050034 | 2.3007 | 0.010478 | TM4SF4 |
| 124976_at | 5.5926 | 0.005016 | 2.2996 | 0.0105 | SPNS2 |
| 2296_at | -5.5895 | 0.0050262 | 2.2988 | 0.010517 | FOXC1 |
| 760_at | 5.5889 | 0.0050282 | 2.2986 | 0.010517 | CA2 |
| 222194_at | -5.5875 | 0.0050328 | 2.2982 | 0.010522 | RSBN1L |
| 115362_at | -5.5858 | 0.0050384 | 2.2977 | 0.01053 | GBP5 |
| 5796_at | -5.5823 | 0.0050497 | 2.2967 | 0.010549 | PTPRK |
| 55620_at | -5.5798 | 0.0050577 | 2.296 | 0.010561 | STAP2 |
| 51249_at | 5.5777 | 0.0050648 | 2.2954 | 0.010569 | TMEM69 |
| 100885782_at | -5.5774 | 0.0050657 | 2.2954 | 0.010569 | MYO16-AS1 |
| 3176_at | 5.5757 | 0.0050714 | 2.2949 | 0.010576 | HNMT |
| 57475_at | 5.5682 | 0.0050959 | 2.2928 | 0.010623 | PLEKHH1 |
| 7071_at | 5.5666 | 0.0051014 | 2.2923 | 0.01063 | KLF10 |
| 6646_at | 5.5629 | 0.0051138 | 2.2913 | 0.010652 | SOAT1 |
| 65117_at | -5.5563 | 0.0051355 | 2.2894 | 0.010692 | RSRC2 |
| 9787_at | -5.5553 | 0.0051392 | 2.2891 | 0.010696 | DLGAP5 |
| 7782_at | 5.5543 | 0.0051423 | 2.2888 | 0.010698 | SLC30A4 |
| 51116_at | 5.552 | 0.00515 | 2.2882 | 0.010709 | MRPS2 |
| 129303_at | 5.5491 | 0.0051598 | 2.2874 | 0.010723 | TMEM150A |
| 26108_at | -5.5487 | 0.005161 | 2.2873 | 0.010723 | PYGO1 |
| 201799_at | -5.5473 | 0.005166 | 2.2868 | 0.010729 | TMEM154 |
| 5209_at | 5.5458 | 0.0051709 | 2.2864 | 0.010735 | PFKFB3 |
| 8540_at | 5.5434 | 0.0051789 | 2.2858 | 0.010743 | AGPS |
| 28978_at | 5.5433 | 0.0051794 | 2.2857 | 0.010743 | TMEM14A |
| 402055_at | -5.5419 | 0.0051839 | 2.2853 | 0.010747 | SRRD |
| 2041_at | -5.5415 | 0.0051853 | 2.2852 | 0.010747 | EPHA1 |
| 7532_at | 5.5376 | 0.0051987 | 2.2841 | 0.01077 | YWHAG |
| 4128_at | 5.5336 | 0.0052124 | 2.283 | 0.010794 | MAOA |
| 55057_at | -5.5277 | 0.0052322 | 2.2813 | 0.010831 | AIM1L |
| 221143_at | 5.5259 | 0.0052385 | 2.2808 | 0.010839 | N6AMT2 |
| 90271_at | -5.5213 | 0.0052543 | 2.2795 | 0.010867 | LINC00263 |
| 440854_at | -5.5163 | 0.0052715 | 2.2781 | 0.010896 | CAPN14 |
| 92935_at | 5.5161 | 0.0052723 | 2.278 | 0.010896 | 2-Mar |
| 22821_at | -5.5128 | 0.0052835 | 2.2771 | 0.010914 | RASA3 |
| 148223_at | 5.509 | 0.0052967 | 2.276 | 0.010937 | C19orf25 |
| 10509_at | 5.5077 | 0.0053012 | 2.2756 | 0.010937 | SEMA4B |
| 6542_at | -5.5077 | 0.0053013 | 2.2756 | 0.010937 | SLC7A2 |
| 23608_at | -5.5031 | 0.0053174 | 2.2743 | 0.010966 | MKRN1 |
| 101927497_at | 5.5014 | 0.0053231 | 2.2738 | 0.010973 | LOC101927497 |
| 55197_at | -5.5005 | 0.0053263 | 2.2736 | 0.010975 | RPRD1A |
| 85315_at | -5.4991 | 0.0053313 | 2.2732 | 0.010981 | PAQR8 |
| 5211_at | 5.4926 | 0.005354 | 2.2713 | 0.011023 | PFKL |
| 84287_at | 5.4917 | 0.0053573 | 2.2711 | 0.011024 | ZDHHC16 |
| 79989_at | 5.4913 | 0.0053586 | 2.2709 | 0.011024 | TTC26 |
| 202374_at | 5.4798 | 0.0053994 | 2.2677 | 0.011103 | STK32A |
| 3081_at | 5.478 | 0.0054057 | 2.2671 | 0.011111 | HGD |
| 92421_at | -5.4773 | 0.0054081 | 2.267 | 0.011111 | CHMP4C |
| 55119_at | -5.4761 | 0.0054124 | 2.2666 | 0.011111 | PRPF38B |
| 126321_at | 5.4756 | 0.0054144 | 2.2665 | 0.011111 | MFSD12 |
| 3265_at | 5.4756 | 0.0054144 | 2.2665 | 0.011111 | HRAS |
| 91860_at | 5.4739 | 0.0054203 | 2.266 | 0.011119 | CALML4 |
| 199_at | -5.4717 | 0.0054282 | 2.2653 | 0.01113 | AIF1 |
| 101928054_at | -5.4697 | 0.0054352 | 2.2648 | 0.01114 | LOC101928054 |
| 2109_at | 5.469 | 0.005438 | 2.2646 | 0.011141 | ETFB |
| 2098_at | 5.4623 | 0.005462 | 2.2626 | 0.011186 | ESD |
| 101929307_at | -5.4615 | 0.005465 | 2.2624 | 0.011187 | LOC101929307 |
| 4854_at | -5.4569 | 0.0054814 | 2.2611 | 0.011216 | NOTCH3 |
| 100422943_at | -5.4545 | 0.0054903 | 2.2604 | 0.01123 | MIR3189 |
| 25840_at | 5.4522 | 0.0054984 | 2.2598 | 0.011242 | METTL7A |
| 595099_at | -5.4511 | 0.0055025 | 2.2594 | 0.011246 | SNORD18B |
| 7186_at | -5.4463 | 0.0055202 | 2.258 | 0.011275 | TRAF2 |
| 29899_at | -5.4454 | 0.0055233 | 2.2578 | 0.011275 | GPSM2 |
| 126917_at | -5.4451 | 0.0055244 | 2.2577 | 0.011275 | IFFO2 |
| 55845_at | 5.4446 | 0.0055261 | 2.2576 | 0.011275 | BRK1 |
| 23031_at | 5.4441 | 0.005528 | 2.2574 | 0.011275 | MAST3 |
| 55184_at | 5.4415 | 0.0055375 | 2.2567 | 0.011289 | DZANK1 |
| 259266_at | -5.4404 | 0.0055416 | 2.2564 | 0.011293 | ASPM |
| 83666_at | -5.4329 | 0.0055691 | 2.2542 | 0.011343 | PARP9 |
| 1979_at | 5.4326 | 0.0055703 | 2.2541 | 0.011343 | EIF4EBP2 |
| 23347_at | -5.4293 | 0.0055824 | 2.2532 | 0.011362 | SMCHD1 |
| 729967_at | 5.4278 | 0.0055882 | 2.2527 | 0.01137 | MORN2 |
| 54587_at | 5.4236 | 0.0056036 | 2.2515 | 0.011396 | MXRA8 |
| 51226_at | 5.4223 | 0.0056083 | 2.2512 | 0.011396 | COPZ2 |
| 4045_at | -5.4221 | 0.0056092 | 2.2511 | 0.011396 | LSAMP |
| 57616_at | -5.4219 | 0.0056101 | 2.251 | 0.011396 | TSHZ3 |
| 11168_at | -5.4155 | 0.0056338 | 2.2492 | 0.011439 | PSIP1 |
| 57447_at | -5.4135 | 0.0056412 | 2.2486 | 0.011449 | NDRG2 |
| 7421_at | -5.413 | 0.0056433 | 2.2485 | 0.011449 | VDR |
| 868_at | 5.4118 | 0.0056476 | 2.2481 | 0.011453 | CBLB |
| 408_at | -5.4111 | 0.0056503 | 2.2479 | 0.011454 | ARRB1 |
| 80739_at | -5.4073 | 0.0056648 | 2.2468 | 0.011479 | C6orf25 |
| 8503_at | -5.4023 | 0.0056834 | 2.2454 | 0.011504 | PIK3R3 |
| 7993_at | 5.4021 | 0.0056842 | 2.2453 | 0.011504 | UBXN8 |
| 729291_at | 5.4021 | 0.0056842 | 2.2453 | 0.011504 | LOC729291 |
| 205428_at | 5.4009 | 0.0056888 | 2.245 | 0.011509 | C3orf58 |
| 2322_at | 5.3999 | 0.0056927 | 2.2447 | 0.011512 | FLT3 |
| 5880_at | -5.3954 | 0.0057096 | 2.2434 | 0.011542 | RAC2 |
| 80323_at | -5.3935 | 0.0057171 | 2.2428 | 0.011552 | CCDC68 |
| 64123_at | -5.3894 | 0.0057327 | 2.2416 | 0.011579 | ELTD1 |
| 2766_at | -5.3863 | 0.0057444 | 2.2408 | 0.011598 | GMPR |
| 647264_at | -5.3846 | 0.005751 | 2.2403 | 0.011607 | LOC647264 |
| 3732_at | -5.3834 | 0.0057555 | 2.2399 | 0.011611 | CD82 |
| 55835_at | -5.3803 | 0.0057674 | 2.239 | 0.011628 | CENPJ |
| 340547_at | 5.38 | 0.0057688 | 2.2389 | 0.011628 | VSIG1 |
| 8776_at | -5.3784 | 0.0057746 | 2.2385 | 0.011632 | MTMR1 |
| 100506243_at | -5.3782 | 0.0057755 | 2.2384 | 0.011632 | KRBOX1 |
| 56998_at | -5.3754 | 0.0057865 | 2.2376 | 0.01165 | CTNNBIP1 |
| 5922_at | -5.3742 | 0.0057911 | 2.2372 | 0.011653 | RASA2 |
| 23022_at | -5.3737 | 0.0057929 | 2.2371 | 0.011653 | PALLD |
| 91156_at | 5.3713 | 0.0058023 | 2.2364 | 0.011668 | IGFN1 |
| 6388_at | 5.3675 | 0.005817 | 2.2353 | 0.011689 | SDF2 |
| 302_at | -5.3673 | 0.0058176 | 2.2353 | 0.011689 | ANXA2 |
| 57509_at | -5.3664 | 0.0058212 | 2.235 | 0.011691 | MTUS1 |
| 10247_at | 5.3658 | 0.0058235 | 2.2348 | 0.011691 | HRSP12 |
| 83468_at | 5.3603 | 0.0058452 | 2.2332 | 0.01173 | GLT8D2 |
| 129450_at | -5.3577 | 0.0058554 | 2.2324 | 0.011746 | TYW5 |
| 339803_at | 5.3521 | 0.0058774 | 2.2308 | 0.011783 | LOC339803 |
| 89782_at | 5.3518 | 0.0058784 | 2.2307 | 0.011783 | LMLN |
| 101927630_at | 5.3506 | 0.0058832 | 2.2304 | 0.011788 | LOC101927630 |
| 89839_at | -5.3493 | 0.0058884 | 2.23 | 0.011793 | ARHGAP11B |
| 23075_at | -5.3483 | 0.0058924 | 2.2297 | 0.011797 | SWAP70 |
| 162681_at | -5.3465 | 0.0058995 | 2.2292 | 0.011806 | C18orf54 |
| 4940_at | -5.3446 | 0.0059071 | 2.2286 | 0.011817 | OAS3 |
| 435_at | 5.3422 | 0.0059164 | 2.2279 | 0.01183 | ASL |
| 10528_at | 5.3415 | 0.0059192 | 2.2277 | 0.011831 | NOP56 |
| 55329_at | 5.3405 | 0.0059233 | 2.2274 | 0.011835 | MNS1 |
| 152518_at | -5.3375 | 0.0059353 | 2.2266 | 0.011849 | NFXL1 |
| 163702_at | -5.3375 | 0.0059353 | 2.2266 | 0.011849 | IFNLR1 |
| 23279_at | -5.3362 | 0.0059403 | 2.2262 | 0.011854 | NUP160 |
| 348254_at | -5.3319 | 0.0059577 | 2.2249 | 0.011884 | CCDC144CP |
| 1739_at | -5.3255 | 0.0059832 | 2.2231 | 0.011931 | DLG1 |
| 100379345_at | -5.3237 | 0.0059905 | 2.2225 | 0.01194 | MIR181A2HG |
| 11234_at | -5.3222 | 0.0059965 | 2.2221 | 0.011948 | HPS5 |
| 399726_at | 5.3187 | 0.0060108 | 2.2211 | 0.011968 | CASC10 |
| 4820_at | -5.3185 | 0.0060116 | 2.221 | 0.011968 | NKTR |
| 10466_at | -5.3156 | 0.0060234 | 2.2202 | 0.011987 | COG5 |
| 100216001_at | 5.3146 | 0.0060275 | 2.2199 | 0.01199 | LINC00704 |
| 5063_at | -5.3121 | 0.0060375 | 2.2191 | 0.012004 | PAK3 |
| 55508_at | 5.3118 | 0.0060391 | 2.219 | 0.012004 | SLC35E3 |
| 161394_at | 5.3097 | 0.0060476 | 2.2184 | 0.012016 | SAMD15 |
| 85236_at | 5.3083 | 0.006053 | 2.218 | 0.012022 | HIST1H2BK |
| 340277_at | -5.3028 | 0.0060756 | 2.2164 | 0.012062 | FAM221A |
| 387590_at | -5.3013 | 0.006082 | 2.216 | 0.012068 | TPTEP1 |
| 80177_at | -5.3009 | 0.0060836 | 2.2158 | 0.012068 | MYCT1 |
| 2329_at | 5.2996 | 0.0060889 | 2.2155 | 0.012074 | FMO4 |
| 158038_at | -5.2978 | 0.0060962 | 2.2149 | 0.012084 | LINGO2 |
| 81605_at | 5.2903 | 0.0061271 | 2.2127 | 0.01214 | URM1 |
| 5705_at | 5.2885 | 0.0061346 | 2.2122 | 0.01215 | PSMC5 |
| 11059_at | -5.2861 | 0.0061447 | 2.2115 | 0.012161 | WWP1 |
| 11346_at | 5.2861 | 0.0061448 | 2.2115 | 0.012161 | SYNPO |
| 8871_at | 5.2833 | 0.0061562 | 2.2107 | 0.012178 | SYNJ2 |
| 4121_at | 5.282 | 0.0061617 | 2.2103 | 0.012184 | MAN1A1 |
| 5441_at | 5.2814 | 0.0061644 | 2.2101 | 0.012185 | POLR2L |
| 148170_at | -5.2801 | 0.0061695 | 2.2097 | 0.01219 | CDC42EP5 |
| 100131378_at | -5.2794 | 0.0061729 | 2.2095 | 0.012192 | C11orf91 |
| 2052_at | 5.2788 | 0.0061751 | 2.2094 | 0.012192 | EPHX1 |
| 5188_at | 5.2776 | 0.0061802 | 2.209 | 0.012193 | GATB |
| 9953_at | -5.2775 | 0.0061805 | 2.209 | 0.012193 | HS3ST3B1 |
| 148022_at | -5.2723 | 0.0062026 | 2.2074 | 0.012231 | TICAM1 |
| 2354_at | 5.2715 | 0.0062058 | 2.2072 | 0.012233 | FOSB |
| 203611_at | 5.2661 | 0.0062284 | 2.2056 | 0.012273 | CDY2B |
| 55612_at | -5.2648 | 0.0062343 | 2.2052 | 0.012279 | FERMT1 |
| 1237_at | -5.2642 | 0.0062367 | 2.205 | 0.012279 | CCR8 |
| 633_at | 5.2631 | 0.0062411 | 2.2047 | 0.012279 | BGN |
| 10935_at | 5.2624 | 0.0062445 | 2.2045 | 0.012279 | PRDX3 |
| 466_at | 5.2623 | 0.0062448 | 2.2045 | 0.012279 | ATF1 |
| 7270_at | -5.2619 | 0.0062465 | 2.2044 | 0.012279 | TTF1 |
| 2880_at | 5.2574 | 0.0062654 | 2.2031 | 0.012312 | GPX5 |
| 6448_at | 5.2548 | 0.0062767 | 2.2023 | 0.012329 | SGSH |
| 55809_at | -5.2526 | 0.0062859 | 2.2016 | 0.012342 | TRERF1 |
| 10178_at | 5.2515 | 0.0062908 | 2.2013 | 0.012347 | TENM1 |
| 116843_at | -5.2468 | 0.0063111 | 2.1999 | 0.012382 | SLC18B1 |
| 50485_at | 5.2456 | 0.0063163 | 2.1995 | 0.012383 | SMARCAL1 |
| 4255_at | 5.2455 | 0.0063165 | 2.1995 | 0.012383 | MGMT |
| 54555_at | 5.2405 | 0.0063381 | 2.198 | 0.012416 | DDX49 |
| 1066_at | -5.2405 | 0.0063383 | 2.198 | 0.012416 | CES1 |
| 5613_at | -5.2386 | 0.0063463 | 2.1975 | 0.012427 | PRKX |
| 79899_at | 5.2373 | 0.0063519 | 2.1971 | 0.012433 | PRR5L |
| 3927_at | 5.2342 | 0.0063655 | 2.1962 | 0.012455 | LASP1 |
| 283578_at | 5.2327 | 0.0063718 | 2.1957 | 0.012462 | TMED8 |
| 55544_at | 5.2319 | 0.0063753 | 2.1955 | 0.012464 | RBM38 |
| 8934_at | 5.2306 | 0.0063809 | 2.1951 | 0.01247 | RAB29 |
| 157285_at | 5.2272 | 0.0063961 | 2.1941 | 0.012492 | SGK223 |
| 6850_at | -5.2269 | 0.0063971 | 2.194 | 0.012492 | SYK |
| 7108_at | -5.2258 | 0.006402 | 2.1937 | 0.012497 | TM7SF2 |
| 101929666_at | -5.2182 | 0.0064354 | 2.1914 | 0.012557 | LOC101929666 |
| 25921_at | 5.2176 | 0.006438 | 2.1912 | 0.012557 | ZDHHC5 |
| 3164_at | -5.2097 | 0.0064729 | 2.1889 | 0.01262 | NR4A1 |
| 4665_at | 5.2076 | 0.0064824 | 2.1883 | 0.012634 | NAB2 |
| 133746_at | -5.2052 | 0.0064931 | 2.1875 | 0.01265 | JMY |
| 3455_at | -5.2031 | 0.0065024 | 2.1869 | 0.012663 | IFNAR2 |
| 100507639_at | 5.1983 | 0.0065238 | 2.1855 | 0.012696 | LOC100507639 |
| 10769_at | -5.1981 | 0.0065246 | 2.1854 | 0.012696 | PLK2 |
| 79670_at | -5.194 | 0.0065429 | 2.1842 | 0.012723 | ZCCHC6 |
| 81856_at | 5.1934 | 0.0065456 | 2.1841 | 0.012723 | ZNF611 |
| 701_at | -5.1928 | 0.0065485 | 2.1839 | 0.012723 | BUB1B |
| 25911_at | 5.1927 | 0.0065487 | 2.1838 | 0.012723 | DPCD |
| 26018_at | -5.1902 | 0.00656 | 2.1831 | 0.01274 | LRIG1 |
| 80243_at | 5.189 | 0.0065657 | 2.1827 | 0.012746 | PREX2 |
| 8078_at | 5.1874 | 0.0065729 | 2.1822 | 0.012751 | USP5 |
| 4071_at | -5.1873 | 0.0065734 | 2.1822 | 0.012751 | TM4SF1 |
| 54503_at | -5.1824 | 0.0065955 | 2.1808 | 0.012789 | ZDHHC13 |
| 23108_at | -5.1804 | 0.0066044 | 2.1802 | 0.012802 | RAP1GAP2 |
| 79836_at | -5.1797 | 0.0066075 | 2.18 | 0.012803 | LONRF3 |
| 85301_at | 5.1781 | 0.0066152 | 2.1795 | 0.012813 | COL27A1 |
| 57221_at | -5.1744 | 0.006632 | 2.1784 | 0.012835 | KIAA1244 |
| 23480_at | 5.1738 | 0.0066344 | 2.1782 | 0.012835 | SEC61G |
| 55681_at | -5.1738 | 0.0066345 | 2.1782 | 0.012835 | SCYL2 |
| 123876_at | 5.1725 | 0.0066406 | 2.1778 | 0.012838 | ACSM2A |
| 716_at | 5.1723 | 0.0066414 | 2.1777 | 0.012838 | C1S |
| 79369_at | 5.1701 | 0.0066515 | 2.1771 | 0.012853 | B3GNT4 |
| 6296_at | 5.1658 | 0.0066714 | 2.1758 | 0.012886 | ACSM3 |
| 5089_at | -5.1652 | 0.0066738 | 2.1756 | 0.012886 | PBX2 |
| 283970_at | -5.1647 | 0.0066762 | 2.1755 | 0.012886 | PDXDC2P |
| 79137_at | 5.1634 | 0.0066824 | 2.1751 | 0.01289 | FAM134A |
| 91368_at | 5.1632 | 0.0066835 | 2.175 | 0.01289 | CDKN2AIPNL |
| 1649_at | -5.1616 | 0.0066905 | 2.1745 | 0.012899 | DDIT3 |
| 3797_at | -5.1599 | 0.0066986 | 2.174 | 0.012909 | KIF3C |
| 153090_at | -5.1564 | 0.0067147 | 2.173 | 0.012932 | DAB2IP |
| 23314_at | -5.1561 | 0.0067162 | 2.1729 | 0.012932 | SATB2 |
| 90634_at | -5.1556 | 0.0067185 | 2.1727 | 0.012932 | N4BP2L1 |
| 27440_at | 5.1542 | 0.0067249 | 2.1723 | 0.012936 | CECR5 |
| 153562_at | -5.1541 | 0.0067256 | 2.1723 | 0.012936 | MARVELD2 |
| 55353_at | -5.1533 | 0.0067292 | 2.172 | 0.012938 | LAPTM4B |
| 10733_at | -5.1502 | 0.0067437 | 2.1711 | 0.012961 | PLK4 |
| 56776_at | -5.1482 | 0.0067529 | 2.1705 | 0.012974 | FMN2 |
| 2237_at | 5.1471 | 0.0067578 | 2.1702 | 0.012978 | FEN1 |
| 8797_at | -5.1436 | 0.0067746 | 2.1691 | 0.013005 | TNFRSF10A |
| 51280_at | 5.1365 | 0.006808 | 2.167 | 0.013065 | GOLM1 |
| 11004_at | -5.1359 | 0.0068108 | 2.1668 | 0.013065 | KIF2C |
| 79084_at | 5.1346 | 0.0068169 | 2.1664 | 0.013072 | WDR77 |
| 440712_at | -5.1266 | 0.0068549 | 2.164 | 0.013139 | C1orf186 |
| 55015_at | -5.1259 | 0.0068578 | 2.1638 | 0.01314 | PRPF39 |
| 54806_at | -5.1254 | 0.0068605 | 2.1636 | 0.01314 | AHI1 |
| 25758_at | -5.1229 | 0.0068721 | 2.1629 | 0.013157 | KIAA1549L |
| 64324_at | -5.1219 | 0.006877 | 2.1626 | 0.013158 | NSD1 |
| 57545_at | 5.1215 | 0.0068791 | 2.1625 | 0.013158 | CC2D2A |
| 5296_at | 5.1212 | 0.0068805 | 2.1624 | 0.013158 | PIK3R2 |
| 80127_at | 5.1145 | 0.0069126 | 2.1604 | 0.013214 | CCDC176 |
| 7468_at | -5.1133 | 0.0069181 | 2.16 | 0.01322 | WHSC1 |
| 9298_at | -5.1051 | 0.006958 | 2.1575 | 0.013291 | SNORD31 |
| 3976_at | -5.1036 | 0.006965 | 2.1571 | 0.013298 | LIF |
| 494335_at | -5.1032 | 0.0069669 | 2.157 | 0.013298 | MIR423 |
| 151742_at | 5.101 | 0.0069775 | 2.1563 | 0.013312 | PPM1L |
| 7130_at | 5.1006 | 0.0069798 | 2.1562 | 0.013312 | TNFAIP6 |
| 285331_at | -5.0979 | 0.0069927 | 2.1554 | 0.013325 | CCDC66 |
| 27352_at | -5.0978 | 0.0069933 | 2.1553 | 0.013325 | SGSM3 |
| 2353_at | 5.0976 | 0.0069944 | 2.1552 | 0.013325 | FOS |
| 9942_at | 5.0959 | 0.0070028 | 2.1547 | 0.013336 | XYLB |
| 141_at | 5.0946 | 0.007009 | 2.1543 | 0.013343 | ADPRH |
| 4856_at | -5.0921 | 0.0070212 | 2.1536 | 0.013361 | NOV |
| 8630_at | -5.0897 | 0.0070329 | 2.1529 | 0.013374 | HSD17B6 |
| 23359_at | 5.0896 | 0.0070334 | 2.1528 | 0.013374 | FAM189A1 |
| 23704_at | 5.0888 | 0.0070374 | 2.1526 | 0.013376 | KCNE4 |
| 51015_at | -5.0832 | 0.007065 | 2.1509 | 0.013424 | ISOC1 |
| 692088_at | -5.0818 | 0.0070717 | 2.1505 | 0.013431 | SNORD50B |
| 85363_at | -5.0803 | 0.0070795 | 2.15 | 0.013441 | TRIM5 |
| 11145_at | -5.079 | 0.0070859 | 2.1496 | 0.013448 | PLA2G16 |
| 54741_at | 5.0762 | 0.0070996 | 2.1488 | 0.013466 | LEPROT |
| 55281_at | 5.0758 | 0.0071014 | 2.1487 | 0.013466 | TMEM140 |
| 56112_at | 5.0755 | 0.0071033 | 2.1485 | 0.013466 | PCDHGA3 |
| 6890_at | -5.0749 | 0.0071061 | 2.1484 | 0.013466 | TAP1 |
| 1468_at | 5.0737 | 0.0071122 | 2.148 | 0.013473 | SLC25A10 |
| 29088_at | 5.0723 | 0.007119 | 2.1476 | 0.013478 | MRPL15 |
| 100302185_at | -5.0719 | 0.0071208 | 2.1475 | 0.013478 | MIR1204 |
| 152002_at | 5.071 | 0.0071252 | 2.1472 | 0.013479 | XXYLT1 |
| 83982_at | 5.0703 | 0.007129 | 2.147 | 0.013479 | IFI27L2 |
| 3898_at | -5.0702 | 0.0071296 | 2.1469 | 0.013479 | LAD1 |
| 10544_at | 5.0697 | 0.0071317 | 2.1468 | 0.013479 | PROCR |
| 23158_at | -5.0686 | 0.0071372 | 2.1465 | 0.013484 | TBC1D9 |
| 79656_at | 5.0675 | 0.0071427 | 2.1461 | 0.013489 | BEND5 |
| 56103_at | 5.0663 | 0.0071491 | 2.1457 | 0.013491 | PCDHGB2 |
| 6491_at | -5.0663 | 0.0071491 | 2.1457 | 0.013491 | STIL |
| 3177_at | -5.0651 | 0.0071548 | 2.1454 | 0.013494 | SLC29A2 |
| 7867_at | 5.0649 | 0.007156 | 2.1453 | 0.013494 | MAPKAPK3 |
| 53834_at | 5.0637 | 0.0071618 | 2.145 | 0.0135 | FGFRL1 |
| 677792_at | -5.0599 | 0.0071813 | 2.1438 | 0.013532 | SNORA1 |
| 3704_at | 5.059 | 0.0071853 | 2.1436 | 0.013534 | ITPA |
| 284069_at | -5.0566 | 0.0071976 | 2.1428 | 0.013552 | FAM171A2 |
| 56113_at | 5.0555 | 0.007203 | 2.1425 | 0.013557 | PCDHGA2 |
| 440584_at | 5.055 | 0.0072058 | 2.1423 | 0.013557 | SLC2A1-AS1 |
| 22998_at | 5.0539 | 0.0072111 | 2.142 | 0.013562 | LIMCH1 |
| 84572_at | 5.0528 | 0.007217 | 2.1416 | 0.013568 | GNPTG |
| 54820_at | -5.0507 | 0.0072275 | 2.141 | 0.013583 | NDE1 |
| 283131_at | -5.0498 | 0.0072319 | 2.1407 | 0.013586 | NEAT1 |
| 57097_at | 5.0432 | 0.0072659 | 2.1387 | 0.013645 | PARP11 |
| 8875_at | -5.0388 | 0.0072883 | 2.1374 | 0.01368 | VNN2 |
| 100302743_at | 5.0384 | 0.0072901 | 2.1373 | 0.01368 | SNORA80B |
| 142680_at | -5.0371 | 0.007297 | 2.1369 | 0.013688 | SLC34A3 |
| 81615_at | 5.0288 | 0.0073399 | 2.1343 | 0.013758 | TMEM163 |
| 115548_at | -5.0281 | 0.0073433 | 2.1341 | 0.013758 | FCHO2 |
| 728407_at | -5.028 | 0.0073439 | 2.1341 | 0.013758 | PARGP1 |
| 23541_at | 5.0276 | 0.0073457 | 2.134 | 0.013758 | SEC14L2 |
| 1716_at | 5.0229 | 0.0073705 | 2.1325 | 0.0138 | DGUOK |
| 1080_at | -5.021 | 0.00738 | 2.1319 | 0.013812 | CFTR |
| 55170_at | 5.0195 | 0.0073879 | 2.1315 | 0.013822 | PRMT6 |
| 145864_at | -5.0174 | 0.0073992 | 2.1308 | 0.013832 | HAPLN3 |
| 723809_at | 5.0169 | 0.0074017 | 2.1307 | 0.013832 | LHFPL3-AS2 |
| 79682_at | -5.0168 | 0.0074018 | 2.1307 | 0.013832 | CENPU |
| 64207_at | -5.0141 | 0.0074162 | 2.1298 | 0.013854 | IRF2BPL |
| 100422865_at | -5.0117 | 0.0074288 | 2.1291 | 0.013865 | MIR4320 |
| 728640_at | 5.0108 | 0.0074333 | 2.1288 | 0.013865 | FAM133CP |
| 151648_at | -5.0102 | 0.0074365 | 2.1286 | 0.013865 | SGOL1 |
| 63893_at | 5.0102 | 0.0074369 | 2.1286 | 0.013865 | UBE2O |
| 2318_at | 5.0099 | 0.0074383 | 2.1285 | 0.013865 | FLNC |
| 2974_at | 5.0098 | 0.0074385 | 2.1285 | 0.013865 | GUCY1B2 |
| 348_at | 5.007 | 0.0074536 | 2.1276 | 0.013888 | APOE |
| 115361_at | -5.0062 | 0.0074578 | 2.1274 | 0.01389 | GBP4 |
| 169792_at | 5.0053 | 0.0074626 | 2.1271 | 0.013894 | GLIS3 |
| 5991_at | -5.0046 | 0.0074658 | 2.1269 | 0.013895 | RFX3 |
| 55332_at | 5.0026 | 0.0074767 | 2.1263 | 0.013906 | DRAM1 |
| 6197_at | 5.0025 | 0.0074774 | 2.1262 | 0.013906 | RPS6KA3 |
| 80215_at | -4.9968 | 0.0075073 | 2.1245 | 0.013956 | RUNX1-IT1 |
| 8382_at | 4.9963 | 0.00751 | 2.1244 | 0.013956 | NME5 |
| 11040_at | 4.9944 | 0.0075204 | 2.1238 | 0.01397 | PIM2 |
| 147841_at | 4.9939 | 0.0075229 | 2.1236 | 0.01397 | SPC24 |
| 303_at | -4.9924 | 0.007531 | 2.1231 | 0.01398 | ANXA2P1 |
| 91768_at | 4.9906 | 0.0075405 | 2.1226 | 0.013992 | CABLES1 |
| 6274_at | 4.9874 | 0.0075576 | 2.1216 | 0.014019 | S100A3 |
| 79789_at | -4.9848 | 0.0075713 | 2.1208 | 0.014039 | CLMN |
| 5768_at | -4.9798 | 0.0075982 | 2.1193 | 0.014084 | QSOX1 |
| 9258_at | -4.9781 | 0.0076075 | 2.1188 | 0.014096 | MFHAS1 |
| 54980_at | 4.9743 | 0.007628 | 2.1176 | 0.014128 | C2orf42 |
| 91862_at | -4.9739 | 0.0076304 | 2.1175 | 0.014128 | MARVELD3 |
| 3434_at | -4.9723 | 0.007639 | 2.117 | 0.014134 | IFIT1 |
| 65996_at | 4.9722 | 0.0076397 | 2.1169 | 0.014134 | CENPBD1P1 |
| 101926996_at | 4.9709 | 0.0076465 | 2.1165 | 0.014141 | RUNDC3A-AS1 |
| 3123_at | 4.9704 | 0.0076492 | 2.1164 | 0.014141 | HLA-DRB1 |
| 10966_at | 4.9685 | 0.0076597 | 2.1158 | 0.014154 | RAB40B |
| 101_at | -4.9678 | 0.0076634 | 2.1156 | 0.014154 | ADAM8 |
| 137695_at | -4.9668 | 0.0076687 | 2.1153 | 0.014154 | TMEM68 |
| 57669_at | -4.9667 | 0.0076693 | 2.1152 | 0.014154 | EPB41L5 |
| 55410_at | 4.9666 | 0.0076703 | 2.1152 | 0.014154 | 55410_at |
| 56101_at | 4.9659 | 0.007674 | 2.115 | 0.014156 | PCDHGB5 |
| 65059_at | -4.9634 | 0.0076873 | 2.1142 | 0.014175 | RAPH1 |
| 8659_at | 4.9626 | 0.0076919 | 2.114 | 0.014178 | ALDH4A1 |
| 57409_at | -4.9588 | 0.0077126 | 2.1128 | 0.014209 | MIF4GD |
| 5740_at | 4.9585 | 0.0077143 | 2.1127 | 0.014209 | PTGIS |
| 151126_at | 4.9573 | 0.0077212 | 2.1123 | 0.014217 | ZNF385B |
| 102724061_at | 4.956 | 0.0077281 | 2.1119 | 0.014223 | LOC102724061 |
| 55320_at | -4.9556 | 0.0077304 | 2.1118 | 0.014223 | MIS18BP1 |
| 8303_at | -4.9547 | 0.007735 | 2.1115 | 0.014226 | SNN |
| 693197_at | -4.9528 | 0.0077457 | 2.1109 | 0.014239 | MIR612 |
| 10391_at | 4.9523 | 0.0077486 | 2.1108 | 0.014239 | CORO2B |
| 10295_at | 4.9519 | 0.0077505 | 2.1107 | 0.014239 | BCKDK |
| 401152_at | 4.9507 | 0.0077572 | 2.1103 | 0.014246 | C4orf3 |
| 352954_at | -4.9485 | 0.0077693 | 2.1096 | 0.014263 | GATS |
| 836_at | -4.9468 | 0.0077788 | 2.1091 | 0.014269 | CASP3 |
| 1294_at | -4.9465 | 0.0077806 | 2.109 | 0.014269 | COL7A1 |
| 65266_at | 4.9464 | 0.0077812 | 2.109 | 0.014269 | WNK4 |
| 319089_at | -4.9454 | 0.0077866 | 2.1087 | 0.014274 | TTC6 |
| 4240_at | -4.9419 | 0.0078062 | 2.1076 | 0.014303 | MFGE8 |
| 123036_at | -4.9416 | 0.007808 | 2.1075 | 0.014303 | TC2N |
| 56062_at | 4.9409 | 0.0078117 | 2.1073 | 0.014304 | KLHL4 |
| 9294_at | -4.9392 | 0.007821 | 2.1067 | 0.014316 | S1PR2 |
| 2635_at | -4.9386 | 0.0078243 | 2.1066 | 0.014316 | GBP3 |
| 101927865_at | -4.9382 | 0.0078267 | 2.1064 | 0.014316 | LOC101927865 |
| 26263_at | -4.9352 | 0.0078436 | 2.1055 | 0.014342 | FBXO22 |
| 84433_at | -4.9344 | 0.0078483 | 2.1052 | 0.014345 | CARD11 |
| 6095_at | 4.9323 | 0.00786 | 2.1046 | 0.014359 | RORA |
| 2243_at | 4.932 | 0.0078616 | 2.1045 | 0.014359 | FGA |
| 200197_at | -4.9313 | 0.0078654 | 2.1043 | 0.014361 | TMEM51-AS1 |
| 55345_at | -4.9292 | 0.0078776 | 2.1036 | 0.014378 | ZGRF1 |
| 1063_at | -4.9259 | 0.0078961 | 2.1026 | 0.014406 | CENPF |
| 4259_at | 4.922 | 0.0079182 | 2.1014 | 0.014438 | MGST3 |
| 5909_at | 4.9218 | 0.0079194 | 2.1013 | 0.014438 | RAP1GAP |
| 27344_at | 4.9204 | 0.007927 | 2.1009 | 0.014447 | PCSK1N |
| 3995_at | 4.9193 | 0.0079333 | 2.1005 | 0.014453 | FADS3 |
| 79725_at | -4.9182 | 0.0079394 | 2.1002 | 0.014454 | THAP9 |
| 84941_at | -4.9182 | 0.0079397 | 2.1002 | 0.014454 | HSH2D |
| 121504_at | 4.9176 | 0.0079429 | 2.1 | 0.014454 | HIST4H4 |
| 493856_at | 4.9143 | 0.007962 | 2.099 | 0.014484 | CISD2 |
| 23624_at | -4.9134 | 0.0079672 | 2.0987 | 0.014488 | CBLC |
| 83857_at | 4.912 | 0.0079751 | 2.0983 | 0.014497 | TMTC1 |
| 11011_at | -4.9089 | 0.007993 | 2.0973 | 0.014525 | TLK2 |
| 166929_at | 4.9056 | 0.0080117 | 2.0963 | 0.014553 | SGMS2 |
| 4489_at | -4.9035 | 0.0080239 | 2.0956 | 0.01457 | MT1A |
| 55831_at | 4.9014 | 0.0080358 | 2.095 | 0.014586 | EMC3 |
| 84930_at | -4.9006 | 0.0080404 | 2.0947 | 0.01459 | MASTL |
| 2669_at | 4.8995 | 0.0080471 | 2.0944 | 0.014596 | GEM |
| 2633_at | 4.8967 | 0.008063 | 2.0935 | 0.014615 | GBP1 |
| 6352_at | -4.8965 | 0.0080645 | 2.0934 | 0.014615 | CCL5 |
| 51776_at | -4.8961 | 0.0080664 | 2.0933 | 0.014615 | ZAK |
| 54708_at | -4.8928 | 0.0080856 | 2.0923 | 0.014639 | 5-Mar |
| 5914_at | 4.8926 | 0.0080867 | 2.0922 | 0.014639 | RARA |
| 134429_at | -4.8919 | 0.0080907 | 2.092 | 0.014639 | STARD4 |
| 100131096_at | -4.8919 | 0.0080909 | 2.092 | 0.014639 | TNRC6C-AS1 |
| 101927060_at | 4.8878 | 0.0081147 | 2.0907 | 0.014675 | LOC101927060 |
| 3939_at | 4.8874 | 0.0081171 | 2.0906 | 0.014675 | LDHA |
| 5585_at | -4.8814 | 0.0081523 | 2.0887 | 0.014734 | PKN1 |
| 2191_at | 4.88 | 0.0081604 | 2.0883 | 0.01474 | FAP |
| 9557_at | 4.8793 | 0.0081646 | 2.0881 | 0.01474 | CHD1L |
| 101927858_at | -4.8793 | 0.0081647 | 2.0881 | 0.01474 | LOC101927858 |
| 3291_at | 4.877 | 0.0081785 | 2.0873 | 0.01476 | HSD11B2 |
| 101927420_at | 4.8741 | 0.0081953 | 2.0864 | 0.01478 | LOC101927420 |
| 63905_at | 4.8741 | 0.0081957 | 2.0864 | 0.01478 | MANBAL |
| 5202_at | -4.8692 | 0.0082242 | 2.0849 | 0.014826 | PFDN2 |
| 1033_at | -4.8685 | 0.0082288 | 2.0847 | 0.014829 | CDKN3 |
| 79628_at | -4.8678 | 0.008233 | 2.0844 | 0.014831 | SH3TC2 |
| 23344_at | 4.8664 | 0.0082412 | 2.084 | 0.014841 | ESYT1 |
| 4192_at | -4.8627 | 0.0082629 | 2.0829 | 0.014874 | MDK |
| 79607_at | -4.8592 | 0.0082842 | 2.0817 | 0.014907 | FAM118B |
| 51057_at | 4.8575 | 0.0082941 | 2.0812 | 0.01492 | WDPCP |
| 8721_at | 4.8568 | 0.0082985 | 2.081 | 0.014922 | EDF1 |
| 5627_at | 4.8525 | 0.0083243 | 2.0797 | 0.014963 | PROS1 |
| 116461_at | -4.8485 | 0.0083485 | 2.0784 | 0.015001 | TSEN15 |
| 101928913_at | 4.8477 | 0.0083533 | 2.0781 | 0.015005 | LINC01515 |
| 22876_at | -4.847 | 0.0083576 | 2.0779 | 0.015007 | INPP5F |
| 339290_at | 4.8392 | 0.0084048 | 2.0755 | 0.015086 | LINC00667 |
| 89944_at | 4.8386 | 0.0084083 | 2.0753 | 0.015087 | GLB1L2 |
| 7474_at | 4.8363 | 0.0084229 | 2.0745 | 0.015108 | WNT5A |
| 58500_at | 4.8352 | 0.0084293 | 2.0742 | 0.015113 | ZNF250 |
| 9337_at | -4.8348 | 0.0084319 | 2.0741 | 0.015113 | CNOT8 |
| 57585_at | -4.8323 | 0.008447 | 2.0733 | 0.015135 | CRAMP1L |
| 100506668_at | 4.8317 | 0.0084511 | 2.0731 | 0.015137 | DYNLL1-AS1 |
| 25999_at | 4.8296 | 0.0084639 | 2.0724 | 0.015154 | CLIP3 |
| 677801_at | 4.8279 | 0.008474 | 2.0719 | 0.015167 | SNORA14A |
| 22829_at | -4.825 | 0.008492 | 2.071 | 0.015194 | NLGN4Y |
| 594839_at | -4.8158 | 0.0085489 | 2.0681 | 0.01529 | SNORA33 |
| 257194_at | -4.8146 | 0.0085565 | 2.0677 | 0.015298 | NEGR1 |
| 203259_at | 4.8122 | 0.0085713 | 2.067 | 0.015319 | FAM219A |
| 23164_at | -4.8103 | 0.0085833 | 2.0663 | 0.01533 | MPRIP |
| 79594_at | 4.8103 | 0.0085833 | 2.0663 | 0.01533 | MUL1 |
| 2524_at | -4.8085 | 0.0085946 | 2.0658 | 0.015344 | FUT2 |
| 10051_at | -4.8079 | 0.0085986 | 2.0656 | 0.015346 | SMC4 |
| 101928584_at | 4.8073 | 0.0086025 | 2.0654 | 0.015348 | LOC101928584 |
| 84176_at | -4.8047 | 0.0086187 | 2.0646 | 0.015368 | MYH16 |
| 58485_at | 4.8044 | 0.0086202 | 2.0645 | 0.015368 | TRAPPC1 |
| 55728_at | -4.8039 | 0.0086235 | 2.0643 | 0.015369 | N4BP2 |
| 222389_at | 4.8021 | 0.0086349 | 2.0637 | 0.015379 | BEND7 |
| 81544_at | -4.802 | 0.0086355 | 2.0637 | 0.015379 | GDPD5 |
| 7092_at | 4.7978 | 0.008662 | 2.0624 | 0.015421 | TLL1 |
| 2193_at | 4.7971 | 0.0086665 | 2.0622 | 0.015423 | FARSA |
| 29937_at | 4.7954 | 0.0086772 | 2.0616 | 0.015437 | NENF |
| 84216_at | 4.7934 | 0.0086902 | 2.061 | 0.015451 | TMEM117 |
| 100506746_at | 4.7932 | 0.0086913 | 2.0609 | 0.015451 | LOC100506746 |
| 101928199_at | -4.7902 | 0.00871 | 2.06 | 0.015479 | LOC101928199 |
| 26043_at | -4.7889 | 0.0087187 | 2.0595 | 0.015489 | UBXN7 |
| 645460_at | -4.7877 | 0.0087263 | 2.0592 | 0.015497 | 645460_at |
| 283554_at | -4.7865 | 0.0087339 | 2.0588 | 0.015503 | GPR137C |
| 84870_at | 4.7862 | 0.0087361 | 2.0587 | 0.015503 | RSPO3 |
| 2919_at | -4.7821 | 0.0087622 | 2.0574 | 0.015544 | CXCL1 |
| 79073_at | 4.7781 | 0.008788 | 2.0561 | 0.015584 | TMEM109 |
| 730755_at | 4.7703 | 0.0088379 | 2.0537 | 0.015665 | KRTAP2-3 |
| 5742_at | -4.77 | 0.0088399 | 2.0536 | 0.015665 | PTGS1 |
| 64595_at | -4.7646 | 0.008875 | 2.0518 | 0.015722 | TTTY15 |
| 51136_at | 4.7605 | 0.0089017 | 2.0505 | 0.015763 | RNFT1 |
| 57455_at | 4.7587 | 0.0089137 | 2.0499 | 0.015779 | REXO1 |
| 4794_at | -4.7556 | 0.0089341 | 2.049 | 0.015809 | NFKBIE |
| 8991_at | 4.7516 | 0.0089603 | 2.0477 | 0.01585 | SELENBP1 |
| 9674_at | -4.7449 | 0.0090044 | 2.0455 | 0.015922 | KIAA0040 |
| 5698_at | -4.7443 | 0.0090084 | 2.0454 | 0.015924 | PSMB9 |
| 2647_at | 4.7437 | 0.0090128 | 2.0451 | 0.015926 | BLOC1S1 |
| 441531_at | 4.7371 | 0.0090563 | 2.043 | 0.015997 | PGAM4 |
| 4345_at | -4.7366 | 0.0090602 | 2.0429 | 0.015999 | CD200 |
| 9738_at | -4.7323 | 0.0090886 | 2.0415 | 0.016043 | CCP110 |
| 101927184_at | 4.7287 | 0.009113 | 2.0403 | 0.01608 | 101927184_at |
| 4939_at | -4.7277 | 0.0091196 | 2.04 | 0.016086 | OAS2 |
| 8564_at | 4.7273 | 0.0091223 | 2.0399 | 0.016086 | KMO |
| 84836_at | 4.7249 | 0.0091381 | 2.0391 | 0.016108 | ABHD14B |
| 203228_at | -4.7239 | 0.0091455 | 2.0388 | 0.016113 | C9orf72 |
| 4863_at | -4.7233 | 0.0091492 | 2.0386 | 0.016113 | NPAT |
| 55709_at | 4.723 | 0.0091511 | 2.0385 | 0.016113 | KBTBD4 |
| 3925_at | -4.7208 | 0.0091659 | 2.0378 | 0.01613 | STMN1 |
| 101927811_at | -4.7207 | 0.0091672 | 2.0378 | 0.01613 | LOC101927811 |
| 23451_at | -4.718 | 0.0091853 | 2.0369 | 0.016146 | SF3B1 |
| 90853_at | 4.7177 | 0.0091872 | 2.0368 | 0.016146 | SPOCD1 |
| 149111_at | -4.7176 | 0.0091875 | 2.0368 | 0.016146 | CNIH3 |
| 10560_at | -4.7174 | 0.0091891 | 2.0367 | 0.016146 | SLC19A2 |
| 57222_at | 4.7123 | 0.0092237 | 2.0351 | 0.016198 | ERGIC1 |
| 9562_at | 4.7119 | 0.0092265 | 2.035 | 0.016198 | MINPP1 |
| 150864_at | -4.7116 | 0.0092285 | 2.0349 | 0.016198 | FAM117B |
| 54472_at | 4.7104 | 0.0092367 | 2.0345 | 0.016207 | TOLLIP |
| 7718_at | -4.7071 | 0.0092593 | 2.0334 | 0.016241 | ZNF165 |
| 6721_at | -4.7064 | 0.0092645 | 2.0332 | 0.016245 | SREBF2 |
| 92312_at | -4.7026 | 0.0092904 | 2.032 | 0.016284 | MEX3A |
| 100616456_at | -4.7013 | 0.0092991 | 2.0316 | 0.016294 | MIR4476 |
| 571_at | -4.6997 | 0.0093106 | 2.031 | 0.016305 | BACH1 |
| 27145_at | 4.6988 | 0.0093165 | 2.0307 | 0.016305 | FILIP1 |
| 102723889_at | 4.6985 | 0.0093186 | 2.0307 | 0.016305 | LOC102723889 |
| 3344_at | -4.6983 | 0.0093203 | 2.0306 | 0.016305 | FOXN2 |
| 23469_at | -4.698 | 0.0093219 | 2.0305 | 0.016305 | PHF3 |
| 645644_at | 4.6958 | 0.0093374 | 2.0298 | 0.016326 | FLJ42627 |
| 387597_at | 4.6923 | 0.009362 | 2.0286 | 0.016364 | ILDR2 |
| 6788_at | -4.6875 | 0.0093949 | 2.0271 | 0.016415 | STK3 |
| 441027_at | 4.6871 | 0.0093981 | 2.027 | 0.016415 | TMEM150C |
| 2615_at | 4.6829 | 0.0094274 | 2.0256 | 0.016461 | LRRC32 |
| 57477_at | 4.6813 | 0.0094385 | 2.0251 | 0.016475 | SHROOM4 |
| 54677_at | -4.6755 | 0.0094793 | 2.0232 | 0.016539 | CROT |
| 92126_at | 4.6751 | 0.0094819 | 2.0231 | 0.016539 | DSEL |
| 400506_at | -4.6671 | 0.0095386 | 2.0205 | 0.016627 | KNOP1 |
| 7046_at | 4.6671 | 0.0095392 | 2.0205 | 0.016627 | TGFBR1 |
| 2118_at | -4.6652 | 0.0095524 | 2.0199 | 0.01664 | ETV4 |
| 79896_at | 4.6651 | 0.0095534 | 2.0198 | 0.01664 | THNSL1 |
| 11183_at | -4.6623 | 0.0095731 | 2.0189 | 0.016669 | MAP4K5 |
| 5601_at | -4.6604 | 0.0095863 | 2.0183 | 0.016686 | MAPK9 |
| 54799_at | -4.6576 | 0.0096067 | 2.0174 | 0.016716 | MBTD1 |
| 55301_at | -4.6564 | 0.0096151 | 2.017 | 0.016721 | OLAH |
| 64398_at | -4.656 | 0.0096182 | 2.0169 | 0.016721 | MPP5 |
| 55540_at | 4.6558 | 0.0096199 | 2.0168 | 0.016721 | IL17RB |
| 9022_at | 4.6553 | 0.0096235 | 2.0167 | 0.016722 | CLIC3 |
| 5097_at | -4.6544 | 0.0096298 | 2.0164 | 0.016727 | PCDH1 |
| 11202_at | -4.6536 | 0.0096353 | 2.0161 | 0.016729 | KLK8 |
| 3832_at | -4.6533 | 0.0096377 | 2.016 | 0.016729 | KIF11 |
| 6565_at | -4.6523 | 0.0096447 | 2.0157 | 0.016735 | SLC15A2 |
| 3786_at | 4.6515 | 0.0096505 | 2.0155 | 0.016739 | KCNQ3 |
| 57462_at | 4.6508 | 0.0096556 | 2.0152 | 0.016742 | KIAA1161 |
| 51174_at | -4.6464 | 0.0096872 | 2.0138 | 0.016791 | TUBD1 |
| 9118_at | -4.6456 | 0.0096936 | 2.0135 | 0.016795 | INA |
| 9126_at | -4.6451 | 0.0096969 | 2.0134 | 0.016795 | SMC3 |
| 2629_at | 4.6444 | 0.009702 | 2.0131 | 0.016795 | GBA |
| 100874353_at | -4.6443 | 0.0097024 | 2.0131 | 0.016795 | RPS6KA2-IT1 |
| 133_at | 4.6433 | 0.0097101 | 2.0128 | 0.016802 | ADM |
| 10423_at | 4.6412 | 0.0097252 | 2.0121 | 0.016822 | CDIPT |
| 5447_at | 4.6402 | 0.0097329 | 2.0118 | 0.01683 | POR |
| 26517_at | 4.6393 | 0.0097389 | 2.0115 | 0.016831 | TIMM13 |
| 23368_at | -4.6388 | 0.0097426 | 2.0113 | 0.016831 | PPP1R13B |
| 3098_at | 4.6387 | 0.0097437 | 2.0113 | 0.016831 | HK1 |
| 202243_at | -4.638 | 0.0097488 | 2.011 | 0.016834 | CCDC125 |
| 8850_at | 4.6367 | 0.0097583 | 2.0106 | 0.016845 | KAT2B |
| 54892_at | -4.6323 | 0.00979 | 2.0092 | 0.016893 | NCAPG2 |
| 54764_at | -4.6292 | 0.0098135 | 2.0082 | 0.016928 | ZRANB1 |
| 338440_at | -4.6283 | 0.0098197 | 2.0079 | 0.016933 | ANO9 |
| 6447_at | -4.6253 | 0.0098416 | 2.0069 | 0.016963 | SCG5 |
| 9227_at | 4.6247 | 0.0098463 | 2.0067 | 0.016963 | LRAT |
| 79932_at | 4.6242 | 0.0098502 | 2.0066 | 0.016963 | KIAA0319L |
| 64847_at | 4.6241 | 0.0098507 | 2.0065 | 0.016963 | SPATA20 |
| 9991_at | -4.6224 | 0.0098632 | 2.006 | 0.016979 | PTBP3 |
| 127700_at | 4.6205 | 0.0098777 | 2.0053 | 0.016998 | OSCP1 |
| 64327_at | -4.6179 | 0.0098965 | 2.0045 | 0.017024 | LMBR1 |
| 80705_at | -4.6172 | 0.0099021 | 2.0043 | 0.017028 | TSGA10 |
| 191_at | 4.6157 | 0.0099129 | 2.0038 | 0.017037 | AHCY |
| 5935_at | 4.6156 | 0.0099139 | 2.0038 | 0.017037 | RBM3 |
| 143872_at | -4.6145 | 0.0099217 | 2.0034 | 0.017044 | ARHGAP42 |
| 64399_at | -4.613 | 0.0099334 | 2.0029 | 0.017057 | HHIP |
| 63934_at | 4.6127 | 0.0099358 | 2.0028 | 0.017057 | ZNF667 |
| 55008_at | -4.6094 | 0.0099599 | 2.0017 | 0.017092 | HERC6 |
| 100506664_at | 4.6068 | 0.0099793 | 2.0009 | 0.01712 | LOC100506664 |
| 170463_at | 4.6027 | 0.010011 | 1.9995 | 0.017168 | SSBP4 |
| 10180_at | -4.602 | 0.010016 | 1.9993 | 0.01717 | RBM6 |
| 9868_at | 4.6013 | 0.010021 | 1.9991 | 0.017173 | TOMM70A |
| 29070_at | 4.6006 | 0.010026 | 1.9989 | 0.017177 | CCDC113 |
| 102723529_at | -4.5995 | 0.010035 | 1.9985 | 0.017179 | LOC102723529 |
| 100131561_at | -4.5995 | 0.010035 | 1.9985 | 0.017179 | FKSG29 |
| 57094_at | 4.5985 | 0.010042 | 1.9982 | 0.017186 | CPA6 |
| 23397_at | -4.598 | 0.010046 | 1.998 | 0.017187 | NCAPH |
| 3142_at | 4.5945 | 0.010072 | 1.9969 | 0.017226 | HLX |
| 2743_at | -4.5934 | 0.010081 | 1.9965 | 0.017234 | GLRB |
| 91694_at | -4.5836 | 0.010155 | 1.9933 | 0.017356 | LONRF1 |
| 23137_at | -4.5822 | 0.010166 | 1.9928 | 0.017369 | SMC5 |
| 1911_at | -4.581 | 0.010176 | 1.9924 | 0.017379 | PHC1 |
| 4157_at | 4.58 | 0.010183 | 1.9921 | 0.017382 | MC1R |
| 10609_at | -4.5798 | 0.010185 | 1.9921 | 0.017382 | LEPREL4 |
| 23154_at | 4.5775 | 0.010203 | 1.9913 | 0.017408 | NCDN |
| 22824_at | 4.5761 | 0.010214 | 1.9908 | 0.01742 | HSPA4L |
| 2194_at | -4.5755 | 0.010218 | 1.9906 | 0.017422 | FASN |
| 1902_at | 4.572 | 0.010245 | 1.9895 | 0.017458 | LPAR1 |
| 64963_at | 4.5718 | 0.010247 | 1.9894 | 0.017458 | MRPS11 |
| 10886_at | -4.5694 | 0.010266 | 1.9886 | 0.017484 | NPFFR2 |
| 84335_at | 4.569 | 0.010269 | 1.9885 | 0.017484 | AKT1S1 |
| 5051_at | 4.5685 | 0.010272 | 1.9883 | 0.017484 | PAFAH2 |
| 9358_at | -4.5681 | 0.010276 | 1.9882 | 0.017484 | ITGBL1 |
| 84465_at | 4.5675 | 0.010281 | 1.988 | 0.017486 | MEGF11 |
| 613_at | -4.5633 | 0.010313 | 1.9866 | 0.017535 | BCR |
| 4603_at | 4.5618 | 0.010325 | 1.9861 | 0.01755 | MYBL1 |
| 7278_at | 4.5585 | 0.010351 | 1.985 | 0.017587 | TUBA3C |
| 93100_at | -4.5581 | 0.010354 | 1.9849 | 0.017587 | NAPRT |
| 9860_at | -4.5575 | 0.010358 | 1.9847 | 0.017589 | LRIG2 |
| 8334_at | 4.557 | 0.010363 | 1.9845 | 0.01759 | HIST1H2AC |
| 143570_at | 4.5555 | 0.010374 | 1.984 | 0.017604 | XRRA1 |
| 8227_at | -4.5511 | 0.010409 | 1.9826 | 0.017656 | AKAP17A |
| 79940_at | 4.5484 | 0.010431 | 1.9817 | 0.017687 | LINC00472 |
| 284406_at | 4.5466 | 0.010444 | 1.9811 | 0.017703 | ZFP82 |
| 84940_at | -4.5463 | 0.010447 | 1.981 | 0.017703 | CORO6 |
| 5878_at | 4.5458 | 0.010451 | 1.9809 | 0.017703 | RAB5C |
| 51596_at | 4.545 | 0.010458 | 1.9806 | 0.017709 | CUTA |
| 26056_at | 4.5418 | 0.010483 | 1.9795 | 0.017743 | RAB11FIP5 |
| 26152_at | -4.5416 | 0.010484 | 1.9795 | 0.017743 | ZNF337 |
| 57574_at | -4.5389 | 0.010506 | 1.9786 | 0.017772 | 4-Mar |
| 55568_at | 4.5372 | 0.010519 | 1.978 | 0.017789 | GALNT10 |
| 728554_at | -4.5364 | 0.010526 | 1.9777 | 0.017794 | LOC728554 |
| 7571_at | -4.5352 | 0.010535 | 1.9774 | 0.017804 | ZNF23 |
| 6617_at | -4.5336 | 0.010549 | 1.9768 | 0.017814 | SNAPC1 |
| 9928_at | -4.5331 | 0.010552 | 1.9767 | 0.017814 | KIF14 |
| 2282_at | -4.533 | 0.010553 | 1.9766 | 0.017814 | FKBP1AP1 |
| 10211_at | 4.5328 | 0.010555 | 1.9765 | 0.017814 | FLOT1 |
| 3340_at | 4.5308 | 0.010571 | 1.9759 | 0.017835 | NDST1 |
| 90427_at | -4.5301 | 0.010577 | 1.9757 | 0.017838 | BMF |
| 90736_at | 4.5293 | 0.010583 | 1.9754 | 0.017843 | FAM104B |
| 100505650_at | -4.5284 | 0.01059 | 1.9751 | 0.017849 | LOC100505650 |
| 10618_at | 4.5273 | 0.010599 | 1.9747 | 0.017858 | TGOLN2 |
| 100505994_at | 4.5213 | 0.010647 | 1.9728 | 0.017933 | LUCAT1 |
| 340371_at | 4.5198 | 0.01066 | 1.9722 | 0.017949 | NRBP2 |
| 84268_at | -4.5166 | 0.010686 | 1.9712 | 0.017986 | RPAIN |
| 121506_at | 4.5159 | 0.010692 | 1.9709 | 0.017991 | ERP27 |
| 84260_at | -4.5152 | 0.010697 | 1.9707 | 0.017993 | TCHP |
| 121456_at | 4.5148 | 0.010701 | 1.9706 | 0.017993 | SLC9A7P1 |
| 11072_at | 4.514 | 0.010707 | 1.9703 | 0.017997 | DUSP14 |
| 401261_at | -4.5124 | 0.01072 | 1.9698 | 0.018013 | FLJ38717 |
| 29950_at | 4.512 | 0.010723 | 1.9697 | 0.018013 | SERTAD1 |
| 3693_at | 4.5089 | 0.010749 | 1.9686 | 0.01805 | ITGB5 |
| 9158_at | 4.5022 | 0.010804 | 1.9664 | 0.018137 | FIBP |
| 101927501_at | -4.4964 | 0.010852 | 1.9645 | 0.018209 | LOC101927501 |
| 4670_at | 4.4961 | 0.010855 | 1.9644 | 0.018209 | HNRNPM |
| 10755_at | -4.4891 | 0.010913 | 1.9621 | 0.018301 | GIPC1 |
| 5238_at | -4.488 | 0.010923 | 1.9617 | 0.01831 | PGM3 |
| 3373_at | 4.4869 | 0.010932 | 1.9613 | 0.01832 | HYAL1 |
| 79731_at | -4.4859 | 0.01094 | 1.961 | 0.018328 | NARS2 |
| 7857_at | 4.4852 | 0.010946 | 1.9608 | 0.018331 | SCG2 |
| 55030_at | -4.4843 | 0.010954 | 1.9604 | 0.018338 | FBXO34 |
| 57531_at | -4.4809 | 0.010982 | 1.9593 | 0.018377 | HACE1 |
| 645332_at | 4.4807 | 0.010984 | 1.9592 | 0.018377 | FAM86C2P |
| 94115_at | -4.4797 | 0.010993 | 1.9589 | 0.018385 | CGB8 |
| 1471_at | 4.4767 | 0.011018 | 1.9579 | 0.018421 | CST3 |
| 79871_at | 4.4749 | 0.011033 | 1.9573 | 0.018435 | RPAP2 |
| 102724108_at | -4.4748 | 0.011034 | 1.9573 | 0.018435 | LOC102724108 |
| 5274_at | 4.4716 | 0.011061 | 1.9562 | 0.018475 | SERPINI1 |
| 8434_at | 4.4707 | 0.011069 | 1.9559 | 0.018481 | RECK |
| 115273_at | 4.4689 | 0.011084 | 1.9553 | 0.0185 | RAB42 |
| 3316_at | 4.4676 | 0.011095 | 1.9549 | 0.018507 | HSPB2 |
| 1998_at | -4.4676 | 0.011095 | 1.9549 | 0.018507 | ELF2 |
| 3705_at | 4.4646 | 0.011121 | 1.9539 | 0.018538 | ITPK1 |
| 55638_at | 4.4645 | 0.011122 | 1.9538 | 0.018538 | SYBU |
| 51422_at | -4.4635 | 0.011131 | 1.9535 | 0.018547 | PRKAG2 |
| 57822_at | -4.463 | 0.011135 | 1.9533 | 0.018547 | GRHL3 |
| 788_at | 4.4626 | 0.011138 | 1.9532 | 0.018547 | SLC25A20 |
| 222171_at | -4.4591 | 0.011168 | 1.952 | 0.018591 | PRR15 |
| 140710_at | -4.458 | 0.011178 | 1.9516 | 0.018601 | SOGA1 |
| 157574_at | -4.4521 | 0.011228 | 1.9497 | 0.018679 | FBXO16 |
| 27439_at | 4.4511 | 0.011237 | 1.9493 | 0.018687 | CECR6 |
| 55024_at | -4.4505 | 0.011243 | 1.9491 | 0.018691 | BANK1 |
| 692195_at | -4.4499 | 0.011247 | 1.9489 | 0.018692 | SNORD75 |
| 8491_at | 4.4493 | 0.011253 | 1.9487 | 0.018695 | MAP4K3 |
| 153222_at | -4.4452 | 0.011289 | 1.9474 | 0.018748 | CREBRF |
| 6819_at | 4.4375 | 0.011356 | 1.9448 | 0.018853 | SULT1C2 |
| 8876_at | -4.4367 | 0.011363 | 1.9445 | 0.018858 | VNN1 |
| 51645_at | 4.4361 | 0.011368 | 1.9443 | 0.018862 | PPIL1 |
| 170690_at | -4.4344 | 0.011383 | 1.9437 | 0.018881 | ADAMTS16 |
| 79802_at | 4.432 | 0.011404 | 1.9429 | 0.018909 | HHIPL2 |
| 112939_at | 4.428 | 0.01144 | 1.9416 | 0.018955 | NACC1 |
| 55061_at | 4.4278 | 0.011441 | 1.9415 | 0.018955 | SUSD4 |
| 129880_at | 4.4275 | 0.011444 | 1.9414 | 0.018955 | BBS5 |
| 6676_at | 4.4263 | 0.011454 | 1.941 | 0.018967 | SPAG4 |
| 5152_at | -4.4231 | 0.011483 | 1.94 | 0.019006 | PDE9A |
| 283687_at | 4.4228 | 0.011486 | 1.9398 | 0.019006 | ST20-AS1 |
| 92_at | 4.4213 | 0.011499 | 1.9393 | 0.019022 | ACVR2A |
| 729603_at | -4.4208 | 0.011504 | 1.9392 | 0.019023 | LOC729603 |
| 80201_at | 4.4196 | 0.011514 | 1.9388 | 0.019032 | HKDC1 |
| 57130_at | 4.4193 | 0.011517 | 1.9387 | 0.019032 | ATP13A1 |
| 55074_at | 4.4184 | 0.011526 | 1.9383 | 0.019041 | OXR1 |
| 407011_at | -4.4172 | 0.011536 | 1.9379 | 0.019051 | MIR23B |
| 55789_at | -4.4161 | 0.011545 | 1.9376 | 0.019061 | DEPDC1B |
| 255743_at | -4.4149 | 0.011556 | 1.9372 | 0.019071 | NPNT |
| 10825_at | 4.4146 | 0.01156 | 1.9371 | 0.019071 | NEU3 |
| 2512_at | 4.4134 | 0.01157 | 1.9367 | 0.019083 | FTL |
| 55776_at | 4.4117 | 0.011585 | 1.9361 | 0.0191 | SAYSD1 |
| 221_at | 4.4114 | 0.011588 | 1.936 | 0.0191 | ALDH3B1 |
| 54880_at | 4.407 | 0.011628 | 1.9345 | 0.019146 | BCOR |
| 10936_at | -4.4068 | 0.01163 | 1.9344 | 0.019146 | GPR75 |
| 11030_at | 4.4067 | 0.011631 | 1.9344 | 0.019146 | RBPMS |
| 5860_at | 4.4065 | 0.011632 | 1.9343 | 0.019146 | QDPR |
| 27076_at | -4.4047 | 0.011649 | 1.9337 | 0.019168 | LYPD3 |
| 153830_at | -4.4019 | 0.011673 | 1.9328 | 0.019202 | RNF145 |
| 254128_at | -4.3998 | 0.011693 | 1.9321 | 0.019229 | NIFK-AS1 |
| 58489_at | -4.3988 | 0.011702 | 1.9317 | 0.019237 | ABHD17C |
| 29095_at | 4.3954 | 0.011733 | 1.9306 | 0.019281 | ORMDL2 |
| 546_at | -4.394 | 0.011746 | 1.9301 | 0.019295 | ATRX |
| 9493_at | -4.3874 | 0.011806 | 1.9279 | 0.019387 | KIF23 |
| 51560_at | 4.3871 | 0.011809 | 1.9278 | 0.019387 | RAB6B |
| 343171_at | -4.3838 | 0.01184 | 1.9267 | 0.019431 | OR2W3 |
| 639_at | -4.3792 | 0.011882 | 1.9251 | 0.01949 | PRDM1 |
| 4751_at | -4.3791 | 0.011884 | 1.925 | 0.01949 | NEK2 |
| 55808_at | -4.3768 | 0.011905 | 1.9243 | 0.019519 | ST6GALNAC1 |
| 6836_at | 4.3746 | 0.011925 | 1.9235 | 0.019543 | SURF4 |
| 580_at | -4.3743 | 0.011928 | 1.9234 | 0.019543 | BARD1 |
| 7146_at | 4.3713 | 0.011955 | 1.9224 | 0.019582 | TNXA |
| 51668_at | 4.3707 | 0.011961 | 1.9222 | 0.019585 | HSPB11 |
| 55352_at | 4.3632 | 0.012032 | 1.9197 | 0.019695 | COPRS |
| 284716_at | 4.3611 | 0.012051 | 1.919 | 0.019714 | RIMKLA |
| 51144_at | -4.3611 | 0.012051 | 1.919 | 0.019714 | HSD17B12 |
| 646201_at | 4.3602 | 0.01206 | 1.9187 | 0.01972 | BASP1P1 |
| 150368_at | -4.3599 | 0.012063 | 1.9185 | 0.01972 | FAM109B |
| 255394_at | -4.3594 | 0.012068 | 1.9184 | 0.019721 | TCP11L2 |
| 22859_at | -4.3536 | 0.012123 | 1.9164 | 0.019804 | LPHN1 |
| 9469_at | 4.3524 | 0.012134 | 1.916 | 0.019816 | CHST3 |
| 9603_at | -4.3504 | 0.012154 | 1.9153 | 0.019842 | NFE2L3 |
| 10072_at | 4.3487 | 0.012169 | 1.9147 | 0.019861 | DPP3 |
| 5315_at | 4.3477 | 0.012179 | 1.9144 | 0.019871 | PKM |
| 5734_at | -4.3471 | 0.012184 | 1.9142 | 0.019873 | PTGER4 |
| 56262_at | -4.346 | 0.012195 | 1.9138 | 0.019884 | LRRC8A |
| 90649_at | -4.3451 | 0.012204 | 1.9135 | 0.019891 | ZNF486 |
| 148932_at | -4.3416 | 0.012238 | 1.9123 | 0.019938 | MOB3C |
| 3108_at | 4.3413 | 0.01224 | 1.9122 | 0.019938 | HLA-DMA |
| 27123_at | 4.336 | 0.012291 | 1.9104 | 0.020009 | DKK2 |
| 116138_at | 4.3359 | 0.012292 | 1.9104 | 0.020009 | KLHDC3 |
| 10542_at | 4.3347 | 0.012304 | 1.9099 | 0.020022 | LAMTOR5 |
| 60401_at | -4.3257 | 0.012392 | 1.9069 | 0.020158 | EDA2R |
| 9133_at | -4.3228 | 0.01242 | 1.9059 | 0.020197 | CCNB2 |
| 102723716_at | 4.3192 | 0.012455 | 1.9047 | 0.020248 | LOC102723716 |
| 124935_at | 4.3138 | 0.012508 | 1.9028 | 0.020328 | SLC43A2 |
| 22903_at | -4.3114 | 0.012532 | 1.902 | 0.020359 | BTBD3 |
| 7056_at | 4.3034 | 0.012612 | 1.8992 | 0.020483 | THBD |
| 115908_at | 4.3008 | 0.012638 | 1.8983 | 0.020518 | CTHRC1 |
| 284297_at | 4.2993 | 0.012652 | 1.8978 | 0.020536 | SSC5D |
| 11273_at | -4.2987 | 0.012658 | 1.8976 | 0.020538 | ATXN2L |
| 6461_at | 4.2959 | 0.012687 | 1.8967 | 0.020575 | SHB |
| 401022_at | 4.2957 | 0.012689 | 1.8966 | 0.020575 | HAGLR |
| 6769_at | 4.2952 | 0.012694 | 1.8964 | 0.020577 | STAC |
| 10495_at | 4.2924 | 0.012722 | 1.8954 | 0.020616 | ENOX2 |
| 2257_at | -4.288 | 0.012766 | 1.8939 | 0.020679 | FGF12 |
| 100009676_at | 4.2875 | 0.012771 | 1.8938 | 0.020682 | ZBTB11-AS1 |
| 57136_at | 4.2863 | 0.012784 | 1.8933 | 0.02069 | APMAP |
| 79657_at | -4.2862 | 0.012785 | 1.8933 | 0.02069 | RPAP3 |
| 54989_at | 4.2848 | 0.012799 | 1.8928 | 0.020705 | ZNF770 |
| 79901_at | -4.2836 | 0.012811 | 1.8924 | 0.020713 | CYBRD1 |
| 92703_at | -4.2835 | 0.012812 | 1.8924 | 0.020713 | TMEM183A |
| 64112_at | 4.283 | 0.012817 | 1.8922 | 0.020716 | MOAP1 |
| 8774_at | -4.2767 | 0.012881 | 1.89 | 0.020808 | NAPG |
| 5885_at | -4.2766 | 0.012882 | 1.89 | 0.020808 | RAD21 |
| 60493_at | 4.276 | 0.012888 | 1.8898 | 0.02081 | FASTKD5 |
| 55190_at | -4.2697 | 0.012953 | 1.8876 | 0.020908 | NUDT11 |
| 5425_at | 4.2682 | 0.012969 | 1.8871 | 0.020923 | POLD2 |
| 79993_at | -4.268 | 0.012971 | 1.887 | 0.020923 | ELOVL7 |
| 84561_at | -4.2672 | 0.012979 | 1.8868 | 0.020929 | SLC12A8 |
| 4919_at | 4.2627 | 0.013026 | 1.8852 | 0.020998 | ROR1 |
| 9467_at | 4.2612 | 0.013042 | 1.8847 | 0.021016 | SH3BP5 |
| 5352_at | 4.2608 | 0.013045 | 1.8846 | 0.021016 | PLOD2 |
| 100506421_at | 4.2604 | 0.01305 | 1.8844 | 0.021016 | LINC01158 |
| 284825_at | 4.2589 | 0.013065 | 1.8839 | 0.021035 | LOC284825 |
| 2874_at | 4.2572 | 0.013083 | 1.8833 | 0.021056 | GPS2 |
| 9653_at | 4.2543 | 0.013113 | 1.8823 | 0.021098 | HS2ST1 |
| 84254_at | 4.2499 | 0.013159 | 1.8808 | 0.021154 | CAMKK1 |
| 6916_at | 4.2497 | 0.013162 | 1.8807 | 0.021154 | TBXAS1 |
| 80164_at | -4.2492 | 0.013166 | 1.8805 | 0.021154 | PRR36 |
| 102723703_at | -4.2492 | 0.013166 | 1.8805 | 0.021154 | LOC102723703 |
| 1673_at | -4.249 | 0.013169 | 1.8805 | 0.021154 | DEFB4A |
| 285282_at | 4.2471 | 0.013189 | 1.8798 | 0.021179 | RABL3 |
| 79616_at | -4.2464 | 0.013196 | 1.8796 | 0.021184 | CCNJL |
| 1407_at | -4.2448 | 0.013213 | 1.879 | 0.021205 | CRY1 |
| 2348_at | -4.2442 | 0.01322 | 1.8788 | 0.021208 | FOLR1 |
| 55605_at | 4.2426 | 0.013236 | 1.8782 | 0.021224 | KIF21A |
| 11000_at | 4.2424 | 0.013238 | 1.8782 | 0.021224 | SLC27A3 |
| 693148_at | -4.2391 | 0.013273 | 1.877 | 0.021272 | MIR563 |
| 57763_at | -4.2388 | 0.013277 | 1.8769 | 0.021272 | ANKRA2 |
| 80723_at | 4.2382 | 0.013283 | 1.8767 | 0.021275 | SLC35G2 |
| 55064_at | -4.2349 | 0.013318 | 1.8756 | 0.021324 | SPATA6L |
| 101410538_at | 4.2289 | 0.013382 | 1.8735 | 0.021421 | MMP24-AS1 |
| 85441_at | -4.2279 | 0.013393 | 1.8731 | 0.021431 | HELZ2 |
| 6880_at | 4.2263 | 0.01341 | 1.8726 | 0.021451 | TAF9 |
| 726_at | -4.2254 | 0.013419 | 1.8723 | 0.021459 | CAPN5 |
| 55328_at | 4.222 | 0.013455 | 1.8711 | 0.021511 | RNLS |
| 92558_at | 4.2214 | 0.013462 | 1.8709 | 0.021515 | CCDC64 |
| 9397_at | 4.2196 | 0.013482 | 1.8702 | 0.02154 | NMT2 |
| 55020_at | 4.2178 | 0.013501 | 1.8696 | 0.021564 | TTC38 |
| 11077_at | -4.2155 | 0.013526 | 1.8688 | 0.021596 | HSF2BP |
| 81890_at | 4.2141 | 0.013542 | 1.8683 | 0.021608 | QTRT1 |
| 88455_at | -4.214 | 0.013543 | 1.8683 | 0.021608 | ANKRD13A |
| 3628_at | -4.2124 | 0.01356 | 1.8677 | 0.02163 | INPP1 |
| 9764_at | 4.2111 | 0.013574 | 1.8673 | 0.021644 | KIAA0513 |
| 4200_at | -4.2107 | 0.013578 | 1.8672 | 0.021644 | ME2 |
| 9946_at | -4.2001 | 0.013695 | 1.8634 | 0.021824 | CRYZL1 |
| 80254_at | -4.1981 | 0.013717 | 1.8628 | 0.021851 | CEP63 |
| 57217_at | -4.1952 | 0.013749 | 1.8617 | 0.021896 | TTC7A |
| 91750_at | -4.1895 | 0.013812 | 1.8597 | 0.02199 | LIN52 |
| 145783_at | 4.1888 | 0.01382 | 1.8595 | 0.021995 | LOC145783 |
| 55049_at | 4.1877 | 0.013832 | 1.8591 | 0.022007 | C19orf60 |
| 5159_at | 4.186 | 0.013851 | 1.8585 | 0.022031 | PDGFRB |
| 256021_at | -4.184 | 0.013874 | 1.8578 | 0.022059 | C12orf79 |
| 1174_at | 4.1835 | 0.013879 | 1.8576 | 0.022061 | AP1S1 |
| 226_at | 4.1828 | 0.013887 | 1.8574 | 0.022067 | ALDOA |
| 79915_at | -4.1814 | 0.013903 | 1.8569 | 0.022085 | ATAD5 |
| 112487_at | 4.1772 | 0.013949 | 1.8554 | 0.022152 | DTD2 |
| 5900_at | 4.1765 | 0.013957 | 1.8552 | 0.022152 | RALGDS |
| 4664_at | -4.1764 | 0.013959 | 1.8552 | 0.022152 | NAB1 |
| 79135_at | -4.1749 | 0.013976 | 1.8546 | 0.022172 | APOO |
| 266727_at | 4.1743 | 0.013982 | 1.8544 | 0.022172 | MDGA1 |
| 123228_at | 4.1741 | 0.013985 | 1.8543 | 0.022172 | SENP8 |
| 513_at | 4.1722 | 0.014007 | 1.8537 | 0.0222 | ATP5D |
| 9317_at | 4.1695 | 0.014037 | 1.8527 | 0.022241 | PTER |
| 2787_at | 4.1689 | 0.014043 | 1.8525 | 0.022244 | GNG5 |
| 122553_at | -4.164 | 0.014099 | 1.8508 | 0.022326 | TRAPPC6B |
| 9615_at | 4.1636 | 0.014104 | 1.8506 | 0.022327 | GDA |
| 6231_at | 4.1608 | 0.014136 | 1.8497 | 0.02237 | RPS26 |
| 133308_at | 4.16 | 0.014145 | 1.8494 | 0.022377 | SLC9B2 |
| 210_at | 4.1582 | 0.014165 | 1.8488 | 0.022402 | ALAD |
| 29078_at | 4.1573 | 0.014176 | 1.8484 | 0.022412 | NDUFAF4 |
| 125893_at | -4.1549 | 0.014203 | 1.8476 | 0.022448 | ZNF816 |
| 83990_at | -4.1531 | 0.014224 | 1.847 | 0.022474 | BRIP1 |
| 468_at | -4.1477 | 0.014287 | 1.8451 | 0.022566 | ATF4 |
| 116238_at | -4.1443 | 0.014327 | 1.8439 | 0.022621 | TLCD1 |
| 5871_at | -4.1402 | 0.014375 | 1.8424 | 0.02269 | MAP4K2 |
| 23412_at | 4.1394 | 0.014384 | 1.8421 | 0.022697 | COMMD3 |
| 11064_at | -4.1373 | 0.014408 | 1.8414 | 0.022729 | CNTRL |
| 152195_at | 4.1362 | 0.014421 | 1.841 | 0.022741 | NUDT16P1 |
| 202181_at | -4.1337 | 0.01445 | 1.8401 | 0.02278 | LOC202181 |
| 56994_at | 4.1328 | 0.01446 | 1.8398 | 0.022789 | CHPT1 |
| 6228_at | 4.1306 | 0.014487 | 1.839 | 0.022823 | RPS23 |
| 282809_at | -4.1292 | 0.014503 | 1.8385 | 0.022842 | POC1B |
| 1047_at | -4.1281 | 0.014516 | 1.8382 | 0.022855 | CLGN |
| 10863_at | 4.1274 | 0.014525 | 1.8379 | 0.022862 | ADAM28 |
| 6137_at | 4.1256 | 0.014546 | 1.8373 | 0.022881 | RPL13 |
| 8936_at | 4.1256 | 0.014546 | 1.8372 | 0.022881 | WASF1 |
| 246126_at | 4.1246 | 0.014557 | 1.8369 | 0.022892 | TXLNGY |
| 84542_at | -4.124 | 0.014565 | 1.8367 | 0.022897 | KIAA1841 |
| 2920_at | -4.1211 | 0.0146 | 1.8357 | 0.022944 | CXCL2 |
| 5129_at | 4.1133 | 0.014693 | 1.8329 | 0.023083 | CDK18 |
| 1196_at | -4.1097 | 0.014736 | 1.8316 | 0.023143 | CLK2 |
| 150684_at | 4.1081 | 0.014755 | 1.8311 | 0.023166 | COMMD1 |
| 283232_at | 4.1064 | 0.014775 | 1.8305 | 0.02319 | TMEM80 |
| 54897_at | -4.1037 | 0.014808 | 1.8295 | 0.023235 | CASZ1 |
| 8495_at | -4.0981 | 0.014876 | 1.8275 | 0.023333 | PPFIBP2 |
| 79413_at | -4.0951 | 0.014912 | 1.8265 | 0.023384 | ZBED2 |
| 29796_at | 4.0894 | 0.014982 | 1.8244 | 0.023486 | UQCR10 |
| 57586_at | -4.089 | 0.014987 | 1.8243 | 0.023486 | SYT13 |
| 254295_at | -4.0885 | 0.014993 | 1.8241 | 0.023488 | PHYHD1 |
| 145389_at | 4.0838 | 0.015051 | 1.8224 | 0.023572 | SLC38A6 |
| 4166_at | -4.0832 | 0.015059 | 1.8222 | 0.023576 | CHST6 |
| 7443_at | -4.0821 | 0.015072 | 1.8218 | 0.02359 | VRK1 |
| 79834_at | 4.0816 | 0.015079 | 1.8216 | 0.023592 | PEAK1 |
| 151651_at | 4.0804 | 0.015094 | 1.8212 | 0.023609 | EFHB |
| 5452_at | -4.079 | 0.015111 | 1.8207 | 0.023629 | POU2F2 |
| 7913_at | -4.0767 | 0.015139 | 1.8199 | 0.023657 | DEK |
| 8748_at | -4.0763 | 0.015144 | 1.8198 | 0.023657 | ADAM20 |
| 9443_at | 4.0761 | 0.015147 | 1.8197 | 0.023657 | MED7 |
| 64743_at | 4.076 | 0.015148 | 1.8196 | 0.023657 | WDR13 |
| 404201_at | 4.0748 | 0.015163 | 1.8192 | 0.023673 | WDFY3-AS2 |
| 100506211_at | 4.0726 | 0.01519 | 1.8184 | 0.023707 | MIR210HG |
| 58526_at | -4.0719 | 0.0152 | 1.8182 | 0.023713 | MID1IP1 |
| 102723989_at | 4.0716 | 0.015203 | 1.8181 | 0.023713 | LOC102723989 |
| 54626_at | -4.0709 | 0.015212 | 1.8178 | 0.023719 | HES2 |
| 8481_at | -4.0607 | 0.01534 | 1.8142 | 0.023912 | OFD1 |
| 93953_at | -4.0572 | 0.015384 | 1.8129 | 0.023972 | ACRC |
| 79763_at | 4.0548 | 0.015415 | 1.8121 | 0.024014 | ISOC2 |
| 51148_at | -4.0533 | 0.015434 | 1.8115 | 0.024035 | CERCAM |
| 91010_at | 4.0516 | 0.015456 | 1.8109 | 0.024062 | FMNL3 |
| 26037_at | 4.0482 | 0.015499 | 1.8097 | 0.02412 | SIPA1L1 |
| 23203_at | 4.0479 | 0.015503 | 1.8096 | 0.02412 | PMPCA |
| 10296_at | 4.0434 | 0.01556 | 1.808 | 0.024201 | MAEA |
| 4708_at | 4.0421 | 0.015577 | 1.8075 | 0.024219 | NDUFB2 |
| 29785_at | -4.0418 | 0.015581 | 1.8074 | 0.024219 | CYP2S1 |
| 5494_at | -4.041 | 0.015591 | 1.8071 | 0.024222 | PPM1A |
| 60684_at | -4.0409 | 0.015593 | 1.8071 | 0.024222 | TRAPPC11 |
| 2668_at | -4.0404 | 0.015599 | 1.8069 | 0.024225 | GDNF |
| 126328_at | 4.0397 | 0.015608 | 1.8067 | 0.024231 | NDUFA11 |
| 64236_at | 4.0386 | 0.015622 | 1.8063 | 0.024245 | PDLIM2 |
| 27132_at | 4.0365 | 0.015649 | 1.8055 | 0.024279 | CPNE7 |
| 115701_at | 4.0356 | 0.015661 | 1.8052 | 0.02429 | ALPK2 |
| 22911_at | 4.0314 | 0.015716 | 1.8037 | 0.024368 | WDR47 |
| 29953_at | -4.0297 | 0.015738 | 1.8031 | 0.024394 | TRHDE |
| 100379224_at | 4.0283 | 0.015755 | 1.8026 | 0.024415 | LOC100379224 |
| 1659_at | -4.0274 | 0.015768 | 1.8022 | 0.024424 | DHX8 |
| 10686_at | -4.0271 | 0.015771 | 1.8021 | 0.024424 | CLDN16 |
| 1508_at | 4.0264 | 0.01578 | 1.8019 | 0.02443 | CTSB |
| 4728_at | 4.0178 | 0.015894 | 1.7988 | 0.024599 | NDUFS8 |
| 57380_at | 4.0164 | 0.015912 | 1.7983 | 0.02462 | MRS2 |
| 5538_at | -4.0153 | 0.015926 | 1.7979 | 0.024633 | PPT1 |
| 26123_at | 4.0079 | 0.016024 | 1.7952 | 0.024777 | TCTN3 |
| 333926_at | -4.0068 | 0.01604 | 1.7948 | 0.024794 | PPM1J |
| 79623_at | 4.0039 | 0.016078 | 1.7938 | 0.024846 | GALNT14 |
| 100506314_at | -4.0034 | 0.016084 | 1.7936 | 0.024847 | LOC100506314 |
| 170506_at | -4.0021 | 0.016102 | 1.7931 | 0.024867 | DHX36 |
| 340075_at | 3.9976 | 0.016162 | 1.7915 | 0.024952 | ARSI |
| 339883_at | -3.9953 | 0.016194 | 1.7907 | 0.024993 | C3orf35 |
| 6159_at | 3.9946 | 0.016203 | 1.7904 | 0.024999 | RPL29 |
| 55852_at | 3.9936 | 0.016216 | 1.79 | 0.025012 | TEX2 |
| 100130691_at | 3.9924 | 0.016232 | 1.7896 | 0.025027 | LOC100130691 |
| 38_at | 3.9922 | 0.016236 | 1.7895 | 0.025027 | ACAT1 |
| 51157_at | 3.9883 | 0.016287 | 1.7881 | 0.025099 | ZNF580 |
| 64225_at | -3.987 | 0.016306 | 1.7877 | 0.025119 | ATL2 |
| 26035_at | -3.9858 | 0.016322 | 1.7872 | 0.025137 | GLCE |
| 116228_at | 3.9832 | 0.016357 | 1.7863 | 0.025183 | COX20 |
| 55975_at | -3.9823 | 0.01637 | 1.7859 | 0.025195 | KLHL7 |
| 64863_at | -3.9818 | 0.016376 | 1.7858 | 0.025196 | METTL4 |
| 590_at | -3.981 | 0.016387 | 1.7855 | 0.025206 | BCHE |
| 3021_at | -3.9796 | 0.016407 | 1.785 | 0.025228 | H3F3B |
| 100289388_at | 3.9792 | 0.016412 | 1.7848 | 0.025229 | KCTD21-AS1 |
| 57082_at | -3.9749 | 0.01647 | 1.7833 | 0.025311 | CASC5 |
| 3980_at | -3.9735 | 0.016491 | 1.7828 | 0.025334 | LIG3 |
| 51582_at | -3.9729 | 0.016498 | 1.7826 | 0.025337 | AZIN1 |
| 100507291_at | -3.9724 | 0.016505 | 1.7824 | 0.025337 | LOC100507291 |
| 386757_at | 3.9722 | 0.016508 | 1.7823 | 0.025337 | SLC6A10P |
| 168620_at | -3.9715 | 0.016518 | 1.7821 | 0.025344 | BHLHA15 |
| 79622_at | 3.969 | 0.016552 | 1.7811 | 0.025389 | SNRNP25 |
| 54869_at | -3.968 | 0.016567 | 1.7808 | 0.025396 | EPS8L1 |
| 100506046_at | -3.9677 | 0.016571 | 1.7806 | 0.025396 | GAS5-AS1 |
| 113452_at | 3.9676 | 0.016572 | 1.7806 | 0.025396 | TMEM54 |
| 254048_at | -3.9663 | 0.016589 | 1.7802 | 0.025415 | UBN2 |
| 63933_at | -3.9625 | 0.016643 | 1.7788 | 0.02549 | MCUR1 |
| 93210_at | 3.9611 | 0.016662 | 1.7783 | 0.025511 | PGAP3 |
| 55224_at | -3.9598 | 0.016681 | 1.7778 | 0.02553 | ETNK2 |
| 51231_at | 3.9595 | 0.016684 | 1.7777 | 0.02553 | VRK3 |
| 619383_at | 3.959 | 0.016691 | 1.7775 | 0.025533 | SCARNA9 |
| 64759_at | 3.9572 | 0.016717 | 1.7768 | 0.025564 | TNS3 |
| 4091_at | 3.956 | 0.016734 | 1.7764 | 0.025575 | SMAD6 |
| 8123_at | -3.9558 | 0.016736 | 1.7764 | 0.025575 | PWAR5 |
| 4824_at | -3.9556 | 0.01674 | 1.7762 | 0.025575 | NKX3-1 |
| 5892_at | 3.9547 | 0.016752 | 1.7759 | 0.025586 | RAD51D |
| 290_at | 3.9523 | 0.016785 | 1.7751 | 0.025629 | ANPEP |
| 79191_at | 3.9515 | 0.016797 | 1.7748 | 0.025636 | IRX3 |
| 9487_at | -3.9513 | 0.0168 | 1.7747 | 0.025636 | PIGL |
| 7360_at | 3.9491 | 0.01683 | 1.7739 | 0.025675 | UGP2 |
| 116984_at | -3.9476 | 0.016851 | 1.7734 | 0.025699 | ARAP2 |
| 10974_at | 3.9459 | 0.016876 | 1.7727 | 0.025728 | ADIRF |
| 10126_at | 3.9448 | 0.016891 | 1.7723 | 0.025743 | DNAL4 |
| 10457_at | -3.9441 | 0.016902 | 1.7721 | 0.025752 | GPNMB |
| 64902_at | 3.9427 | 0.016921 | 1.7716 | 0.025774 | AGXT2 |
| 5139_at | 3.9412 | 0.016942 | 1.771 | 0.025795 | PDE3A |
| 8790_at | 3.941 | 0.016946 | 1.7709 | 0.025795 | FPGT |
| 92689_at | -3.9384 | 0.016983 | 1.77 | 0.025842 | FAM114A1 |
| 4615_at | -3.9381 | 0.016987 | 1.7699 | 0.025842 | MYD88 |
| 9531_at | 3.9357 | 0.017022 | 1.769 | 0.025881 | BAG3 |
| 3159_at | -3.9354 | 0.017025 | 1.7689 | 0.025881 | HMGA1 |
| 54868_at | 3.9352 | 0.017028 | 1.7688 | 0.025881 | TMEM104 |
| 5307_at | 3.9321 | 0.017073 | 1.7677 | 0.025942 | PITX1 |
| 2483_at | 3.9301 | 0.017102 | 1.767 | 0.025975 | FRG1 |
| 24138_at | -3.9298 | 0.017106 | 1.7669 | 0.025975 | IFIT5 |
| 6414_at | 3.928 | 0.017132 | 1.7662 | 0.026001 | SEPP1 |
| 7776_at | -3.9279 | 0.017133 | 1.7662 | 0.026001 | ZNF236 |
| 6483_at | 3.9258 | 0.017164 | 1.7654 | 0.02604 | ST3GAL2 |
| 9644_at | -3.9253 | 0.017171 | 1.7652 | 0.026043 | SH3PXD2A |
| 1209_at | 3.9245 | 0.017182 | 1.7649 | 0.026045 | CLPTM1 |
| 100132169_at | -3.9245 | 0.017183 | 1.7649 | 0.026045 | WASIR2 |
| 153572_at | -3.9236 | 0.017195 | 1.7646 | 0.026056 | IRX2 |
| 4208_at | -3.9203 | 0.017243 | 1.7634 | 0.026121 | MEF2C |
| 3977_at | 3.9159 | 0.017307 | 1.7618 | 0.026209 | LIFR |
| 10277_at | -3.9148 | 0.017324 | 1.7614 | 0.026217 | UBE4B |
| 84623_at | -3.9146 | 0.017326 | 1.7613 | 0.026217 | KIRREL3 |
| 200185_at | 3.9145 | 0.017328 | 1.7613 | 0.026217 | KRTCAP2 |
| 125476_at | -3.9141 | 0.017333 | 1.7611 | 0.026217 | INO80C |
| 4257_at | 3.9129 | 0.017352 | 1.7607 | 0.026238 | MGST1 |
| 4482_at | 3.9114 | 0.017374 | 1.7601 | 0.026263 | MSRA |
| 9721_at | -3.9091 | 0.017407 | 1.7593 | 0.026305 | GPRIN2 |
| 3320_at | -3.9087 | 0.017413 | 1.7591 | 0.026306 | HSP90AA1 |
| 9956_at | -3.907 | 0.017438 | 1.7585 | 0.026337 | HS3ST2 |
| 64418_at | -3.9064 | 0.017447 | 1.7583 | 0.026341 | TMEM168 |
| 201294_at | -3.9031 | 0.017496 | 1.7571 | 0.026394 | UNC13D |
| 201134_at | 3.9028 | 0.017499 | 1.757 | 0.026394 | CEP112 |
| 27019_at | 3.9028 | 0.0175 | 1.757 | 0.026394 | DNAI1 |
| 83638_at | 3.9026 | 0.017502 | 1.7569 | 0.026394 | C11orf68 |
| 80216_at | -3.9013 | 0.017523 | 1.7564 | 0.026416 | ALPK1 |
| 3201_at | 3.9008 | 0.017529 | 1.7562 | 0.026418 | HOXA4 |
| 55796_at | 3.9 | 0.017542 | 1.7559 | 0.026429 | MBNL3 |
| 53340_at | 3.8986 | 0.017562 | 1.7554 | 0.026452 | SPA17 |
| 400322_at | -3.8969 | 0.017587 | 1.7548 | 0.026481 | HERC2P2 |
| 7272_at | -3.8953 | 0.017612 | 1.7542 | 0.026511 | TTK |
| 389114_at | -3.8946 | 0.017622 | 1.7539 | 0.026518 | ZNF662 |
| 10403_at | -3.8942 | 0.017628 | 1.7538 | 0.026519 | NDC80 |
| 9241_at | -3.8933 | 0.017641 | 1.7535 | 0.026531 | NOG |
| 5997_at | 3.8927 | 0.01765 | 1.7533 | 0.026536 | RGS2 |
| 85391_at | -3.8914 | 0.017669 | 1.7528 | 0.026552 | SNORD14E |
| 81035_at | -3.8913 | 0.017671 | 1.7527 | 0.026552 | COLEC12 |
| 7094_at | 3.8901 | 0.017689 | 1.7523 | 0.026571 | TLN1 |
| 10244_at | 3.8859 | 0.017752 | 1.7507 | 0.026658 | RABEPK |
| 219970_at | 3.8843 | 0.017776 | 1.7502 | 0.026686 | GLYATL2 |
| 55139_at | 3.882 | 0.017811 | 1.7493 | 0.026721 | ANKZF1 |
| 7539_at | 3.8819 | 0.017812 | 1.7493 | 0.026721 | ZFP37 |
| 6160_at | -3.8817 | 0.017816 | 1.7492 | 0.026721 | RPL31 |
| 1849_at | -3.8812 | 0.017823 | 1.749 | 0.026724 | DUSP7 |
| 162962_at | 3.8793 | 0.017852 | 1.7483 | 0.02676 | ZNF836 |
| 163786_at | -3.8784 | 0.017866 | 1.748 | 0.026769 | SASS6 |
| 80781_at | 3.8781 | 0.017869 | 1.7479 | 0.026769 | COL18A1 |
| 53635_at | -3.8758 | 0.017905 | 1.747 | 0.02681 | PTOV1 |
| 84133_at | 3.8757 | 0.017907 | 1.747 | 0.02681 | ZNRF3 |
| 9580_at | -3.8736 | 0.017938 | 1.7462 | 0.026849 | SOX13 |
| 27151_at | -3.8702 | 0.017991 | 1.745 | 0.026904 | CPAMD8 |
| 400728_at | -3.8701 | 0.017991 | 1.7449 | 0.026904 | FAM87B |
| 26256_at | -3.8701 | 0.017992 | 1.7449 | 0.026904 | CABYR |
| 11001_at | -3.8685 | 0.018016 | 1.7443 | 0.026932 | SLC27A2 |
| 11033_at | -3.8668 | 0.018043 | 1.7437 | 0.026965 | ADAP1 |
| 57473_at | 3.8662 | 0.018052 | 1.7435 | 0.02697 | ZNF512B |
| 4744_at | 3.8642 | 0.018082 | 1.7427 | 0.027007 | NEFH |
| 3028_at | 3.8627 | 0.018105 | 1.7422 | 0.027033 | HSD17B10 |
| 57404_at | 3.8572 | 0.018191 | 1.7401 | 0.027154 | CYP20A1 |
| 84436_at | 3.8558 | 0.018212 | 1.7396 | 0.027177 | ZNF528 |
| 22863_at | -3.8552 | 0.018221 | 1.7394 | 0.027182 | ATG14 |
| 79077_at | 3.8537 | 0.018244 | 1.7389 | 0.027209 | DCTPP1 |
| 8528_at | 3.8512 | 0.018284 | 1.7379 | 0.02726 | DDO |
| 29965_at | 3.849 | 0.018318 | 1.7371 | 0.027302 | CDIP1 |
| 4947_at | 3.8476 | 0.018341 | 1.7366 | 0.027328 | OAZ2 |
| 80270_at | 3.8461 | 0.018364 | 1.736 | 0.027354 | HSD3B7 |
| 84619_at | 3.8453 | 0.018377 | 1.7357 | 0.027365 | ZGPAT |
| 130355_at | 3.8448 | 0.018384 | 1.7356 | 0.027368 | C2orf76 |
| 85360_at | 3.8437 | 0.018401 | 1.7352 | 0.027385 | SYDE1 |
| 114614_at | 3.8391 | 0.018474 | 1.7334 | 0.027486 | MIR155HG |
| 79142_at | 3.8386 | 0.018481 | 1.7333 | 0.027489 | PHF23 |
| 2588_at | 3.8343 | 0.01855 | 1.7317 | 0.027582 | GALNS |
| 101927477_at | 3.8277 | 0.018655 | 1.7292 | 0.027731 | LINC01507 |
| 11153_at | 3.8269 | 0.018667 | 1.7289 | 0.02774 | FICD |
| 152559_at | -3.8224 | 0.018739 | 1.7273 | 0.027828 | PAQR3 |
| 8260_at | 3.8222 | 0.018742 | 1.7272 | 0.027828 | NAA10 |
| 3109_at | 3.8222 | 0.018743 | 1.7272 | 0.027828 | HLA-DMB |
| 51542_at | -3.8197 | 0.018782 | 1.7262 | 0.027878 | VPS54 |
| 57639_at | -3.8193 | 0.01879 | 1.7261 | 0.027881 | CCDC146 |
| 92104_at | 3.8188 | 0.018798 | 1.7259 | 0.027882 | TTC30A |
| 677814_at | -3.8185 | 0.018802 | 1.7258 | 0.027882 | SNORA31 |
| 442903_at | -3.8176 | 0.018816 | 1.7255 | 0.027889 | MIR331 |
| 101929335_at | -3.8175 | 0.018818 | 1.7254 | 0.027889 | ADAMTS9-AS1 |
| 28988_at | 3.8167 | 0.018832 | 1.7251 | 0.027902 | DBNL |
| 388610_at | 3.8161 | 0.018841 | 1.7249 | 0.027907 | TRNP1 |
| 8841_at | 3.8149 | 0.01886 | 1.7245 | 0.027927 | HDAC3 |
| 403239_at | 3.8085 | 0.018965 | 1.722 | 0.028074 | OR2T27 |
| 115004_at | -3.806 | 0.019006 | 1.7211 | 0.028126 | MB21D1 |
| 1123_at | -3.8055 | 0.019014 | 1.7209 | 0.02813 | CHN1 |
| 5001_at | 3.8041 | 0.019037 | 1.7204 | 0.028156 | ORC5 |
| 102724224_at | 3.8031 | 0.019053 | 1.72 | 0.028171 | LINC01117 |
| 55144_at | 3.8027 | 0.019059 | 1.7199 | 0.028172 | LRRC8D |
| 285943_at | 3.8019 | 0.019072 | 1.7196 | 0.028183 | HOXA-AS2 |
| 55325_at | 3.8003 | 0.019099 | 1.719 | 0.028214 | UFSP2 |
| 6610_at | 3.7949 | 0.019188 | 1.717 | 0.02833 | SMPD2 |
| 3094_at | 3.7948 | 0.019189 | 1.717 | 0.02833 | HINT1 |
| 892_at | -3.792 | 0.019236 | 1.7159 | 0.028391 | CCNC |
| 414918_at | 3.7889 | 0.019287 | 1.7147 | 0.028459 | DENND6B |
| 11322_at | -3.7882 | 0.019298 | 1.7145 | 0.028466 | TMC6 |
| 10915_at | -3.7869 | 0.01932 | 1.714 | 0.02849 | TCERG1 |
| 5092_at | 3.7862 | 0.019332 | 1.7137 | 0.028492 | PCBD1 |
| 100616425_at | 3.7861 | 0.019333 | 1.7137 | 0.028492 | MIR4748 |
| 51313_at | 3.7773 | 0.019481 | 1.7104 | 0.028703 | FAM198B |
| 8706_at | 3.7696 | 0.019611 | 1.7075 | 0.028884 | B3GALNT1 |
| 51693_at | 3.7688 | 0.019624 | 1.7072 | 0.028896 | TRAPPC2L |
| 57489_at | -3.7675 | 0.019646 | 1.7067 | 0.02892 | ODF2L |
| 379_at | 3.7667 | 0.019661 | 1.7064 | 0.028933 | ARL4D |
| 8818_at | 3.7646 | 0.019696 | 1.7056 | 0.028976 | DPM2 |
| 57121_at | -3.7632 | 0.019719 | 1.7051 | 0.028996 | LPAR5 |
| 5955_at | -3.7631 | 0.019722 | 1.7051 | 0.028996 | RCN2 |
| 29066_at | -3.7628 | 0.019727 | 1.7049 | 0.028996 | ZC3H7A |
| 64786_at | -3.7593 | 0.019788 | 1.7036 | 0.029077 | TBC1D15 |
| 100506783_at | 3.754 | 0.019877 | 1.7016 | 0.0292 | HOXD-AS2 |
| 4211_at | 3.7528 | 0.019898 | 1.7012 | 0.029222 | MEIS1 |
| 8470_at | -3.7515 | 0.019921 | 1.7007 | 0.029241 | SORBS2 |
| 51428_at | 3.7514 | 0.019923 | 1.7006 | 0.029241 | DDX41 |
| 121457_at | 3.7505 | 0.019938 | 1.7003 | 0.029254 | IKBIP |
| 8497_at | 3.7494 | 0.019957 | 1.6999 | 0.029274 | PPFIA4 |
| 378805_at | -3.7483 | 0.019977 | 1.6995 | 0.029295 | LINC-PINT |
| 26816_at | 3.7463 | 0.020012 | 1.6987 | 0.029337 | SNORD35A |
| 6662_at | -3.7434 | 0.020061 | 1.6976 | 0.029401 | SOX9 |
| 79762_at | 3.7428 | 0.020073 | 1.6974 | 0.029409 | C1orf115 |
| 1029_at | -3.7415 | 0.020095 | 1.6969 | 0.029433 | CDKN2A |
| 983_at | -3.7409 | 0.020106 | 1.6967 | 0.02944 | CDK1 |
| 80704_at | 3.7396 | 0.020128 | 1.6962 | 0.029464 | SLC19A3 |
| 85450_at | -3.7378 | 0.020159 | 1.6955 | 0.029501 | ITPRIP |
| 29931_at | 3.7371 | 0.020172 | 1.6953 | 0.029511 | LINC00312 |
| 64218_at | -3.7355 | 0.0202 | 1.6947 | 0.029543 | SEMA4A |
| 54979_at | -3.7349 | 0.020211 | 1.6944 | 0.02955 | HRASLS2 |
| 8526_at | -3.7331 | 0.020242 | 1.6937 | 0.029588 | DGKE |
| 10975_at | 3.7325 | 0.020253 | 1.6935 | 0.029591 | UQCR11 |
| 84832_at | 3.7323 | 0.020256 | 1.6934 | 0.029591 | ANKRD36BP1 |
| 1892_at | 3.7305 | 0.020287 | 1.6928 | 0.029628 | ECHS1 |
| 55166_at | -3.7292 | 0.020311 | 1.6923 | 0.029649 | CENPQ |
| 63951_at | 3.729 | 0.020314 | 1.6922 | 0.029649 | DMRTA1 |
| 57804_at | 3.7273 | 0.020344 | 1.6916 | 0.029684 | POLD4 |
| 5184_at | 3.7261 | 0.020366 | 1.6911 | 0.029708 | PEPD |
| 5616_at | -3.7258 | 0.020372 | 1.691 | 0.029708 | PRKY |
| 158160_at | -3.7231 | 0.02042 | 1.69 | 0.029769 | HSD17B7P2 |
| 28971_at | 3.7219 | 0.020441 | 1.6895 | 0.029792 | AAMDC |
| 11190_at | 3.7205 | 0.020466 | 1.689 | 0.029819 | CEP250 |
| 1969_at | -3.7183 | 0.020506 | 1.6881 | 0.029868 | EPHA2 |
| 54935_at | 3.7169 | 0.020531 | 1.6876 | 0.029896 | DUSP23 |
| 646762_at | 3.7152 | 0.02056 | 1.687 | 0.02993 | LOC646762 |
| 55084_at | 3.7145 | 0.020573 | 1.6867 | 0.02994 | SOBP |
| 9077_at | 3.7118 | 0.020621 | 1.6857 | 0.030002 | DIRAS3 |
| 166824_at | 3.7105 | 0.020645 | 1.6852 | 0.030027 | RASSF6 |
| 124923_at | -3.7099 | 0.020657 | 1.6849 | 0.030036 | SGK494 |
| 8192_at | 3.7078 | 0.020693 | 1.6842 | 0.03008 | CLPP |
| 30827_at | 3.7068 | 0.020711 | 1.6838 | 0.030097 | CXXC1 |
| 28962_at | 3.7055 | 0.020736 | 1.6833 | 0.030121 | OSTM1 |
| 23322_at | 3.7053 | 0.020739 | 1.6832 | 0.030121 | RPGRIP1L |
| 11173_at | -3.7048 | 0.020749 | 1.683 | 0.030126 | ADAMTS7 |
| 10489_at | 3.7017 | 0.020805 | 1.6818 | 0.030192 | LRRC41 |
| 55909_at | 3.7016 | 0.020806 | 1.6818 | 0.030192 | BIN3 |
| 9993_at | 3.6995 | 0.020844 | 1.681 | 0.030238 | DGCR2 |
| 7514_at | -3.699 | 0.020853 | 1.6808 | 0.030242 | XPO1 |
| 26049_at | -3.6977 | 0.020877 | 1.6803 | 0.030268 | FAM169A |
| 84984_at | 3.697 | 0.020891 | 1.68 | 0.03028 | CEP19 |
| 93183_at | 3.6962 | 0.020905 | 1.6797 | 0.030291 | PIGM |
| 3643_at | -3.694 | 0.020945 | 1.6789 | 0.030341 | INSR |
| 100033820_at | -3.6932 | 0.02096 | 1.6786 | 0.030346 | SNORD116-28 |
| 64359_at | 3.6931 | 0.020961 | 1.6786 | 0.030346 | NXN |
| 10581_at | 3.6922 | 0.020978 | 1.6782 | 0.030361 | IFITM2 |
| 55004_at | 3.6912 | 0.020997 | 1.6779 | 0.030378 | LAMTOR1 |
| 57188_at | 3.6909 | 0.021001 | 1.6778 | 0.030378 | ADAMTSL3 |
| 84953_at | -3.6895 | 0.021029 | 1.6772 | 0.030409 | MICALCL |
| 54332_at | 3.6884 | 0.021048 | 1.6768 | 0.030427 | GDAP1 |
| 23616_at | -3.6876 | 0.021063 | 1.6765 | 0.030441 | SH3BP1 |
| 158293_at | 3.6859 | 0.021095 | 1.6758 | 0.030478 | FAM120AOS |
| 9040_at | 3.6849 | 0.021113 | 1.6755 | 0.030495 | UBE2M |
| 55130_at | -3.6828 | 0.021152 | 1.6746 | 0.030543 | ARMC4 |
| 55111_at | 3.6817 | 0.021173 | 1.6742 | 0.030564 | PLEKHJ1 |
| 102723356_at | -3.6809 | 0.021188 | 1.6739 | 0.030577 | ZFPM2-AS1 |
| 27339_at | 3.6793 | 0.021217 | 1.6733 | 0.03061 | PRPF19 |
| 170394_at | -3.6776 | 0.021248 | 1.6727 | 0.030646 | PWWP2B |
| 51287_at | 3.6773 | 0.021255 | 1.6725 | 0.030647 | COA4 |
| 283460_at | 3.6757 | 0.021283 | 1.672 | 0.030679 | HNF1A-AS1 |
| 22878_at | -3.675 | 0.021297 | 1.6717 | 0.03069 | TRAPPC8 |
| 2298_at | 3.6747 | 0.021304 | 1.6715 | 0.030691 | FOXD4 |
| 3146_at | -3.6725 | 0.021344 | 1.6707 | 0.03074 | HMGB1 |
| 9949_at | 3.6652 | 0.021482 | 1.6679 | 0.03093 | AMMECR1 |
| 84144_at | -3.6642 | 0.0215 | 1.6676 | 0.030946 | SYDE2 |
| 51380_at | 3.6593 | 0.021594 | 1.6657 | 0.031073 | CSAD |
| 54996_at | 3.6569 | 0.02164 | 1.6647 | 0.03113 | 2-Mar |
| 27090_at | 3.6549 | 0.021677 | 1.664 | 0.031175 | ST6GALNAC4 |
| 153241_at | -3.6542 | 0.021691 | 1.6637 | 0.031186 | CEP120 |
| 64321_at | 3.6517 | 0.02174 | 1.6627 | 0.031247 | SOX17 |
| 23285_at | -3.6511 | 0.021749 | 1.6626 | 0.031252 | KIAA1107 |
| 81893_at | 3.6479 | 0.021812 | 1.6613 | 0.031322 | SLC7A5P1 |
| 3217_at | -3.6477 | 0.021816 | 1.6612 | 0.031322 | HOXB7 |
| 53_at | 3.6476 | 0.021817 | 1.6612 | 0.031322 | ACP2 |
| 5583_at | -3.6469 | 0.021831 | 1.6609 | 0.03133 | PRKCH |
| 11047_at | 3.6467 | 0.021835 | 1.6608 | 0.03133 | ADRM1 |
| 6525_at | 3.6442 | 0.021883 | 1.6599 | 0.03139 | SMTN |
| 1376_at | 3.6382 | 0.022 | 1.6576 | 0.031548 | CPT2 |
| 27443_at | 3.6342 | 0.022078 | 1.656 | 0.031651 | CECR2 |
| 57646_at | -3.6338 | 0.022085 | 1.6559 | 0.031652 | USP28 |
| 100500849_at | -3.6324 | 0.022114 | 1.6553 | 0.031684 | MIR3916 |
| 29887_at | 3.6302 | 0.022156 | 1.6545 | 0.031736 | SNX10 |
| 23277_at | 3.6298 | 0.022164 | 1.6544 | 0.031738 | CLUH |
| 311_at | -3.6275 | 0.022209 | 1.6535 | 0.031794 | ANXA11 |
| 56992_at | -3.6269 | 0.022221 | 1.6532 | 0.031801 | KIF15 |
| 60561_at | -3.6249 | 0.022261 | 1.6525 | 0.031841 | RINT1 |
| 10913_at | -3.6249 | 0.022261 | 1.6525 | 0.031841 | EDAR |
| 25978_at | -3.6214 | 0.022329 | 1.6511 | 0.031928 | CHMP2B |
| 100130890_at | 3.6205 | 0.022348 | 1.6508 | 0.031946 | TSTD3 |
| 25893_at | -3.6198 | 0.022361 | 1.6505 | 0.031948 | TRIM58 |
| 3692_at | 3.6198 | 0.022362 | 1.6505 | 0.031948 | EIF6 |
| 51655_at | 3.619 | 0.022377 | 1.6502 | 0.031961 | RASD1 |
| 55669_at | -3.6184 | 0.02239 | 1.65 | 0.03197 | MFN1 |
| 55505_at | 3.6152 | 0.022452 | 1.6487 | 0.03205 | NOP10 |
| 84674_at | -3.6135 | 0.022487 | 1.6481 | 0.032091 | CARD6 |
| 3148_at | -3.6124 | 0.022508 | 1.6477 | 0.032112 | HMGB2 |
| 7005_at | -3.6114 | 0.022528 | 1.6473 | 0.03213 | TEAD3 |
| 8899_at | -3.61 | 0.022557 | 1.6467 | 0.032162 | PRPF4B |
| 440050_at | 3.6079 | 0.022598 | 1.6459 | 0.032206 | KRTAP5-7 |
| 101930375_at | 3.6073 | 0.022611 | 1.6457 | 0.032206 | LOC101930375 |
| 7852_at | 3.6072 | 0.022614 | 1.6456 | 0.032206 | CXCR4 |
| 64747_at | 3.6071 | 0.022614 | 1.6456 | 0.032206 | MFSD1 |
| 54658_at | 3.6069 | 0.022619 | 1.6455 | 0.032206 | UGT1A1 |
| 23474_at | -3.6038 | 0.02268 | 1.6443 | 0.032283 | ETHE1 |
| 6596_at | -3.6034 | 0.022688 | 1.6442 | 0.032286 | HLTF |
| 23558_at | -3.6008 | 0.022741 | 1.6432 | 0.032352 | WBP2 |
| 648791_at | 3.6 | 0.022759 | 1.6429 | 0.032367 | PPP1R3G |
| 23194_at | -3.5985 | 0.022789 | 1.6423 | 0.032391 | FBXL7 |
| 101927262_at | 3.5981 | 0.022797 | 1.6421 | 0.032391 | LOC101927262 |
| 153020_at | -3.5979 | 0.022801 | 1.6421 | 0.032391 | RASGEF1B |
| 347051_at | -3.5979 | 0.022801 | 1.642 | 0.032391 | SLC10A5 |
| 130814_at | 3.5945 | 0.02287 | 1.6407 | 0.03248 | PQLC3 |
| 56052_at | 3.5901 | 0.022961 | 1.639 | 0.032599 | ALG1 |
| 7643_at | -3.5885 | 0.022993 | 1.6384 | 0.032636 | ZNF90 |
| 145483_at | 3.588 | 0.023003 | 1.6382 | 0.032641 | FAM161B |
| 286205_at | -3.5857 | 0.023051 | 1.6373 | 0.032692 | SCAI |
| 23710_at | 3.5856 | 0.023052 | 1.6373 | 0.032692 | GABARAPL1 |
| 442893_at | -3.5832 | 0.023102 | 1.6363 | 0.03274 | MIR151A |
| 25959_at | 3.583 | 0.023105 | 1.6363 | 0.03274 | KANK2 |
| 57335_at | -3.5828 | 0.02311 | 1.6362 | 0.03274 | ZNF286A |
| 27244_at | -3.5827 | 0.023113 | 1.6361 | 0.03274 | SESN1 |
| 55635_at | -3.58 | 0.023168 | 1.6351 | 0.032809 | DEPDC1 |
| 6733_at | -3.5781 | 0.023207 | 1.6344 | 0.032855 | SRPK2 |
| 9063_at | 3.577 | 0.023229 | 1.634 | 0.032878 | PIAS2 |
| 728323_at | -3.5758 | 0.023254 | 1.6335 | 0.032903 | LOC728323 |
| 54800_at | -3.5729 | 0.023314 | 1.6324 | 0.032979 | KLHL24 |
| 94097_at | 3.5721 | 0.023332 | 1.632 | 0.032995 | SFXN5 |
| 9189_at | 3.5686 | 0.023404 | 1.6307 | 0.033087 | ZBED1 |
| 51021_at | 3.5678 | 0.023422 | 1.6304 | 0.033104 | MRPS16 |
| 55009_at | 3.5649 | 0.023483 | 1.6292 | 0.033181 | C19orf24 |
| 150967_at | 3.5635 | 0.023512 | 1.6287 | 0.033212 | PKI55 |
| 26801_at | 3.5566 | 0.023657 | 1.626 | 0.033407 | SNORD48 |
| 4130_at | 3.5549 | 0.023695 | 1.6253 | 0.033451 | MAP1A |
| 26001_at | 3.5539 | 0.023716 | 1.625 | 0.033457 | RNF167 |
| 23479_at | -3.5536 | 0.023721 | 1.6249 | 0.033457 | ISCU |
| 9806_at | -3.5534 | 0.023725 | 1.6248 | 0.033457 | SPOCK2 |
| 11238_at | 3.5534 | 0.023725 | 1.6248 | 0.033457 | CA5B |
| 55466_at | -3.5504 | 0.02379 | 1.6236 | 0.033538 | DNAJA4 |
| 138162_at | 3.5489 | 0.023822 | 1.623 | 0.033574 | C9orf116 |
| 1152_at | 3.5474 | 0.023855 | 1.6224 | 0.033611 | CKB |
| 54103_at | -3.5449 | 0.023908 | 1.6215 | 0.033668 | GSAP |
| 53981_at | -3.5448 | 0.023909 | 1.6214 | 0.033668 | CPSF2 |
| 5362_at | -3.5433 | 0.023943 | 1.6208 | 0.033706 | PLXNA2 |
| 8775_at | 3.5395 | 0.024023 | 1.6194 | 0.03381 | NAPA |
| 3990_at | 3.5361 | 0.024099 | 1.618 | 0.033906 | LIPC |
| 10420_at | -3.5358 | 0.024105 | 1.6179 | 0.033906 | TESK2 |
| 81847_at | -3.5336 | 0.024152 | 1.6171 | 0.033962 | RNF146 |
| 5393_at | -3.5301 | 0.024228 | 1.6157 | 0.034059 | EXOSC9 |
| 10452_at | 3.5298 | 0.024235 | 1.6156 | 0.034059 | TOMM40 |
| 132320_at | -3.5294 | 0.024244 | 1.6154 | 0.034059 | SCLT1 |
| 3141_at | 3.5292 | 0.024248 | 1.6153 | 0.034059 | HLCS |
| 283417_at | 3.5259 | 0.02432 | 1.614 | 0.03415 | DPY19L2 |
| 4634_at | 3.5231 | 0.024383 | 1.6129 | 0.034229 | MYL3 |
| 2170_at | 3.5194 | 0.024464 | 1.6115 | 0.034333 | FABP3 |
| 5016_at | -3.5185 | 0.024483 | 1.6111 | 0.034345 | OVGP1 |
| 84662_at | 3.5184 | 0.024486 | 1.6111 | 0.034345 | GLIS2 |
| 9702_at | -3.5144 | 0.024574 | 1.6095 | 0.034458 | CEP57 |
| 467_at | -3.5067 | 0.024746 | 1.6065 | 0.034687 | ATF3 |
| 5787_at | -3.5065 | 0.02475 | 1.6064 | 0.034687 | PTPRB |
| 388507_at | 3.5047 | 0.02479 | 1.6057 | 0.034732 | ZNF788 |
| 3654_at | 3.5044 | 0.024797 | 1.6056 | 0.034732 | IRAK1 |
| 256471_at | -3.5036 | 0.024814 | 1.6053 | 0.034747 | MFSD8 |
| 27335_at | 3.5031 | 0.024826 | 1.6051 | 0.034753 | EIF3K |
| 23424_at | -3.5028 | 0.024833 | 1.605 | 0.034754 | TDRD7 |
| 406988_at | -3.4979 | 0.024944 | 1.603 | 0.0349 | MIR205 |
| 10969_at | 3.4946 | 0.025019 | 1.6017 | 0.034987 | EBNA1BP2 |
| 2256_at | 3.4943 | 0.025026 | 1.6016 | 0.034987 | FGF11 |
| 196463_at | 3.4942 | 0.025028 | 1.6016 | 0.034987 | PLBD2 |
| 353142_at | 3.4921 | 0.025075 | 1.6008 | 0.035043 | LCE3A |
| 5432_at | 3.49 | 0.025123 | 1.5999 | 0.0351 | POLR2C |
| 55691_at | -3.4895 | 0.025134 | 1.5997 | 0.035106 | FRMD4A |
| 90441_at | 3.4877 | 0.025176 | 1.599 | 0.035154 | ZNF622 |
| 5794_at | -3.4848 | 0.025241 | 1.5979 | 0.035237 | PTPRH |
| 283848_at | 3.4773 | 0.025415 | 1.5949 | 0.035469 | CES4A |
| 118924_at | -3.4674 | 0.025645 | 1.591 | 0.03578 | FRA10AC1 |
| 677797_at | 3.4651 | 0.025699 | 1.5901 | 0.035845 | SNORA7B |
| 80760_at | 3.461 | 0.025794 | 1.5885 | 0.035968 | ITIH5 |
| 284697_at | -3.46 | 0.025817 | 1.5881 | 0.03599 | BTBD8 |
| 83443_at | 3.4593 | 0.025834 | 1.5878 | 0.036003 | SF3B5 |
| 9855_at | 3.4589 | 0.025843 | 1.5877 | 0.036006 | FARP2 |
| 55954_at | 3.4582 | 0.025861 | 1.5874 | 0.036015 | ZMAT5 |
| 3073_at | 3.4581 | 0.025864 | 1.5873 | 0.036015 | HEXA |
| 10093_at | 3.4564 | 0.025902 | 1.5867 | 0.036057 | ARPC4 |
| 56006_at | -3.4555 | 0.025925 | 1.5863 | 0.03608 | SMG9 |
| 4752_at | -3.4541 | 0.025956 | 1.5858 | 0.036114 | NEK3 |
| 390_at | -3.4528 | 0.025988 | 1.5852 | 0.036148 | RND3 |
| 158_at | 3.4492 | 0.026074 | 1.5838 | 0.036257 | ADSL |
| 64434_at | -3.4483 | 0.026094 | 1.5835 | 0.036275 | NOM1 |
| 7627_at | 3.4425 | 0.026235 | 1.5811 | 0.036447 | ZNF75A |
| 8140_at | 3.4424 | 0.026235 | 1.5811 | 0.036447 | SLC7A5 |
| 156_at | -3.4422 | 0.02624 | 1.581 | 0.036447 | ADRBK1 |
| 64800_at | 3.4414 | 0.02626 | 1.5807 | 0.03646 | EFCAB6 |
| 677812_at | -3.4412 | 0.026264 | 1.5806 | 0.03646 | SNORA29 |
| 4351_at | 3.4391 | 0.026316 | 1.5798 | 0.036513 | MPI |
| 64089_at | -3.4388 | 0.026322 | 1.5797 | 0.036513 | SNX16 |
| 353333_at | 3.4388 | 0.026324 | 1.5797 | 0.036513 | KRTAP10-10 |
| 22868_at | 3.4366 | 0.026375 | 1.5788 | 0.036566 | FASTKD2 |
| 23648_at | 3.4363 | 0.026382 | 1.5787 | 0.036566 | SSBP3 |
| 286437_at | 3.4363 | 0.026384 | 1.5787 | 0.036566 | LOC286437 |
| 822_at | -3.4333 | 0.026454 | 1.5775 | 0.036653 | CAPG |
| 3270_at | 3.4328 | 0.026469 | 1.5773 | 0.036663 | HRC |
| 387787_at | 3.4307 | 0.026518 | 1.5765 | 0.036722 | LIPT2 |
| 11266_at | -3.4287 | 0.026567 | 1.5757 | 0.036779 | DUSP12 |
| 6566_at | 3.4278 | 0.02659 | 1.5753 | 0.036801 | SLC16A1 |
| 5104_at | -3.4264 | 0.026623 | 1.5747 | 0.036836 | SERPINA5 |
| 57213_at | 3.4258 | 0.026638 | 1.5745 | 0.036847 | SPRYD7 |
| 10402_at | -3.4239 | 0.026684 | 1.5737 | 0.0369 | ST3GAL6 |
| 5434_at | 3.42 | 0.026781 | 1.5722 | 0.037015 | POLR2E |
| 28638_at | -3.4199 | 0.026782 | 1.5722 | 0.037015 | TRBC2 |
| 196483_at | 3.4192 | 0.0268 | 1.5719 | 0.03703 | EEF2KMT |
| 79948_at | -3.4183 | 0.026822 | 1.5715 | 0.03705 | LPPR3 |
| 5281_at | -3.4174 | 0.026845 | 1.5711 | 0.037071 | PIGF |
| 79648_at | -3.416 | 0.026878 | 1.5706 | 0.037107 | MCPH1 |
| 51700_at | -3.4153 | 0.026895 | 1.5703 | 0.03712 | CYB5R2 |
| 55671_at | -3.4137 | 0.026936 | 1.5697 | 0.037166 | SMEK1 |
| 57192_at | 3.4089 | 0.027055 | 1.5678 | 0.03732 | MCOLN1 |
| 1195_at | -3.4082 | 0.027073 | 1.5675 | 0.037334 | CLK1 |
| 60468_at | -3.4069 | 0.027104 | 1.567 | 0.037368 | BACH2 |
| 79078_at | 3.4053 | 0.027144 | 1.5663 | 0.037412 | C1orf50 |
| 594837_at | -3.4036 | 0.027186 | 1.5657 | 0.03746 | SNORD101 |
| 150381_at | -3.4003 | 0.02727 | 1.5643 | 0.037564 | PRR34-AS1 |
| 80063_at | -3.3997 | 0.027285 | 1.5641 | 0.037574 | ATF7IP2 |
| 80311_at | -3.3971 | 0.02735 | 1.563 | 0.037654 | KLHL15 |
| 5641_at | 3.386 | 0.027633 | 1.5586 | 0.038033 | LGMN |
| 11262_at | 3.3843 | 0.027674 | 1.5579 | 0.038079 | SP140 |
| 83935_at | -3.3837 | 0.02769 | 1.5577 | 0.038091 | TMEM133 |
| 26019_at | -3.3821 | 0.027731 | 1.557 | 0.038137 | UPF2 |
| 57126_at | 3.3818 | 0.02774 | 1.5569 | 0.038138 | CD177 |
| 5253_at | -3.3791 | 0.027809 | 1.5558 | 0.038223 | PHF2 |
| 26098_at | -3.3775 | 0.02785 | 1.5552 | 0.03827 | EDRF1 |
| 55957_at | 3.3764 | 0.027877 | 1.5548 | 0.038296 | LIN37 |
| 26063_at | 3.3746 | 0.027924 | 1.554 | 0.038349 | DECR2 |
| 100653515_at | -3.374 | 0.02794 | 1.5538 | 0.038361 | KIAA1731NL |
| 4199_at | -3.3689 | 0.028071 | 1.5517 | 0.038531 | ME1 |
| 8558_at | -3.3683 | 0.028088 | 1.5515 | 0.038534 | CDK10 |
| 1382_at | -3.3682 | 0.028089 | 1.5515 | 0.038534 | CRABP2 |
| 3113_at | 3.3653 | 0.028166 | 1.5503 | 0.038618 | HLA-DPA1 |
| 116064_at | 3.3653 | 0.028167 | 1.5503 | 0.038618 | LRRC58 |
| 10322_at | 3.365 | 0.028174 | 1.5502 | 0.038618 | SMYD5 |
| 1052_at | 3.3615 | 0.028266 | 1.5487 | 0.038734 | CEBPD |
| 57143_at | 3.3607 | 0.028286 | 1.5484 | 0.038751 | ADCK1 |
| 23012_at | -3.3591 | 0.028329 | 1.5478 | 0.038799 | STK38L |
| 100859930_at | -3.357 | 0.028384 | 1.5469 | 0.038863 | HEIH |
| 29904_at | 3.3567 | 0.028391 | 1.5468 | 0.038863 | EEF2K |
| 441631_at | 3.353 | 0.028489 | 1.5453 | 0.038986 | TSPAN11 |
| 2706_at | 3.3525 | 0.028502 | 1.5451 | 0.038994 | GJB2 |
| 102724005_at | 3.3511 | 0.02854 | 1.5445 | 0.039035 | LOC102724005 |
| 6548_at | -3.3492 | 0.028591 | 1.5438 | 0.039094 | SLC9A1 |
| 1026_at | -3.3476 | 0.028633 | 1.5431 | 0.03914 | CDKN1A |
| 130574_at | -3.3469 | 0.028652 | 1.5428 | 0.039155 | LYPD6 |
| 25797_at | 3.3466 | 0.028659 | 1.5427 | 0.039155 | QPCT |
| 79651_at | -3.3455 | 0.028689 | 1.5423 | 0.039185 | RHBDF2 |
| 79887_at | 3.3439 | 0.028731 | 1.5416 | 0.039232 | PLBD1 |
| 9020_at | 3.3425 | 0.028768 | 1.5411 | 0.039266 | MAP3K14 |
| 4061_at | -3.3424 | 0.028772 | 1.541 | 0.039266 | LY6E |
| 80307_at | 3.3391 | 0.028859 | 1.5397 | 0.039374 | FER1L4 |
| 728290_at | 3.3384 | 0.028878 | 1.5394 | 0.039389 | LOC728290 |
| 22808_at | 3.3373 | 0.028908 | 1.539 | 0.039419 | MRAS |
| 55520_at | 3.3367 | 0.028925 | 1.5387 | 0.039432 | ELAC1 |
| 54951_at | 3.3362 | 0.028939 | 1.5385 | 0.039432 | COMMD8 |
| 407006_at | -3.336 | 0.028942 | 1.5385 | 0.039432 | MIR221 |
| 2119_at | -3.3358 | 0.028949 | 1.5384 | 0.039432 | ETV5 |
| 92714_at | -3.3329 | 0.029027 | 1.5372 | 0.039527 | ARRDC1 |
| 5575_at | 3.3306 | 0.029089 | 1.5363 | 0.0396 | PRKAR1B |
| 200844_at | 3.3302 | 0.0291 | 1.5361 | 0.0396 | C3orf67 |
| 1175_at | 3.3301 | 0.029104 | 1.536 | 0.0396 | AP2S1 |
| 3134_at | -3.3286 | 0.029145 | 1.5354 | 0.039643 | HLA-F |
| 9175_at | -3.3283 | 0.029152 | 1.5353 | 0.039643 | MAP3K13 |
| 5876_at | -3.3275 | 0.029173 | 1.535 | 0.039662 | RABGGTB |
| 158046_at | 3.3251 | 0.029239 | 1.534 | 0.039734 | NXNL2 |
| 379025_at | 3.325 | 0.029242 | 1.534 | 0.039734 | FLJ31306 |
| 9260_at | 3.3238 | 0.029275 | 1.5335 | 0.039759 | PDLIM7 |
| 23255_at | 3.3237 | 0.029277 | 1.5335 | 0.039759 | MTCL1 |
| 6256_at | 3.3202 | 0.029373 | 1.5321 | 0.039879 | RXRA |
| 55076_at | 3.3146 | 0.029528 | 1.5298 | 0.040079 | TMEM45A |
| 84527_at | 3.3137 | 0.029551 | 1.5294 | 0.040098 | ZNF559 |
| 7869_at | -3.3133 | 0.029563 | 1.5292 | 0.040098 | SEMA3B |
| 538_at | 3.3132 | 0.029567 | 1.5292 | 0.040098 | ATP7A |
| 256281_at | 3.3116 | 0.029609 | 1.5286 | 0.040142 | NUDT14 |
| 22823_at | -3.3114 | 0.029615 | 1.5285 | 0.040142 | MTF2 |
| 8537_at | -3.3078 | 0.029716 | 1.527 | 0.040268 | BCAS1 |
| 396_at | 3.3063 | 0.029757 | 1.5264 | 0.040312 | ARHGDIA |
| 11022_at | -3.3055 | 0.02978 | 1.5261 | 0.040334 | TDRKH |
| 101929407_at | 3.3039 | 0.029825 | 1.5254 | 0.040384 | PTPRD-AS1 |
| 55851_at | 3.3022 | 0.029871 | 1.5248 | 0.040434 | PSENEN |
| 9906_at | 3.3016 | 0.029888 | 1.5245 | 0.040446 | SLC35E2 |
| 55007_at | -3.3003 | 0.029926 | 1.5239 | 0.040487 | FAM118A |
| 8170_at | -3.299 | 0.029962 | 1.5234 | 0.040517 | SLC14A2 |
| 58477_at | 3.2989 | 0.029965 | 1.5234 | 0.040517 | SRPRB |
| 348174_at | 3.2978 | 0.029994 | 1.523 | 0.040546 | CLEC18A |
| 389792_at | 3.2974 | 0.030005 | 1.5228 | 0.04055 | IER5L |
| 401149_at | 3.2971 | 0.030014 | 1.5227 | 0.040551 | LINC01061 |
| 51101_at | -3.2949 | 0.030078 | 1.5218 | 0.040626 | ZC2HC1A |
| 101929021_at | 3.2915 | 0.030174 | 1.5204 | 0.040745 | LOC101929021 |
| 57862_at | 3.2865 | 0.030314 | 1.5184 | 0.040923 | ZNF410 |
| 90589_at | 3.2848 | 0.030363 | 1.5176 | 0.040979 | ZNF625 |
| 50861_at | 3.2835 | 0.030402 | 1.5171 | 0.041019 | STMN3 |
| 100616196_at | 3.2824 | 0.030433 | 1.5167 | 0.041051 | MIR4525 |
| 4688_at | -3.2811 | 0.030468 | 1.5162 | 0.041087 | NCF2 |
| 51564_at | -3.2797 | 0.03051 | 1.5156 | 0.041132 | HDAC7 |
| 3009_at | 3.2764 | 0.030605 | 1.5142 | 0.04125 | HIST1H1B |
| 91289_at | 3.276 | 0.030617 | 1.514 | 0.041252 | LMF2 |
| 147949_at | 3.2755 | 0.030629 | 1.5139 | 0.041252 | ZNF583 |
| 25842_at | 3.2755 | 0.030631 | 1.5138 | 0.041252 | ASF1A |
| 8604_at | 3.2721 | 0.030728 | 1.5125 | 0.04137 | SLC25A12 |
| 5333_at | -3.2691 | 0.030815 | 1.5112 | 0.041477 | PLCD1 |
| 8532_at | -3.267 | 0.030877 | 1.5104 | 0.041542 | CPZ |
| 134266_at | -3.2669 | 0.03088 | 1.5103 | 0.041542 | GRPEL2 |
| 4085_at | -3.2664 | 0.030894 | 1.5101 | 0.04155 | MAD2L1 |
| 3958_at | -3.2576 | 0.031152 | 1.5065 | 0.041886 | LGALS3 |
| 55670_at | 3.2551 | 0.031225 | 1.5055 | 0.041951 | PEX26 |
| 3066_at | -3.2551 | 0.031226 | 1.5055 | 0.041951 | HDAC2 |
| 51719_at | -3.2551 | 0.031226 | 1.5055 | 0.041951 | CAB39 |
| 53344_at | 3.2492 | 0.031398 | 1.5031 | 0.042171 | CHIC1 |
| 10471_at | 3.2479 | 0.031439 | 1.5025 | 0.042214 | PFDN6 |
| 9121_at | -3.2473 | 0.031457 | 1.5023 | 0.042227 | SLC16A5 |
| 4337_at | 3.2459 | 0.031496 | 1.5017 | 0.042268 | MOCS1 |
| 25816_at | -3.2452 | 0.031519 | 1.5014 | 0.042288 | TNFAIP8 |
| 100499177_at | -3.2417 | 0.031622 | 1.5 | 0.042414 | THAP9-AS1 |
| 84243_at | -3.2381 | 0.03173 | 1.4985 | 0.042547 | ZDHHC18 |
| 79959_at | -3.2376 | 0.031746 | 1.4983 | 0.042558 | CEP76 |
| 153733_at | -3.2372 | 0.031757 | 1.4982 | 0.042561 | CCDC112 |
| 102724550_at | 3.2346 | 0.031837 | 1.4971 | 0.042657 | LOC102724550 |
| 10570_at | 3.2335 | 0.031871 | 1.4966 | 0.042691 | DPYSL4 |
| 29108_at | -3.2311 | 0.031943 | 1.4956 | 0.042777 | PYCARD |
| 142913_at | -3.2295 | 0.031992 | 1.495 | 0.04283 | CFL1P1 |
| 6173_at | -3.2276 | 0.032049 | 1.4942 | 0.042894 | RPL36A |
| 1517_at | 3.226 | 0.032097 | 1.4935 | 0.042945 | CTSLP2 |
| 10514_at | 3.2258 | 0.032103 | 1.4935 | 0.042945 | MYBBP1A |
| 9585_at | -3.2248 | 0.032134 | 1.493 | 0.042974 | KIF20B |
| 6498_at | -3.2238 | 0.032164 | 1.4926 | 0.043002 | SKIL |
| 101669767_at | -3.2232 | 0.032182 | 1.4924 | 0.043015 | SCHLAP1 |
| 219931_at | 3.2223 | 0.03221 | 1.492 | 0.043042 | TPCN2 |
| 100616249_at | 3.2155 | 0.032419 | 1.4892 | 0.043309 | MIR4768 |
| 101927836_at | -3.2122 | 0.032522 | 1.4878 | 0.043435 | 101927836_at |
| 79751_at | 3.2108 | 0.032564 | 1.4873 | 0.04348 | SLC25A22 |
| 23589_at | 3.2102 | 0.032584 | 1.487 | 0.043494 | CARHSP1 |
| 57562_at | 3.2092 | 0.032614 | 1.4866 | 0.043518 | KIAA1377 |
| 100505767_at | 3.209 | 0.032619 | 1.4865 | 0.043518 | LOC100505767 |
| 6500_at | -3.2074 | 0.03267 | 1.4859 | 0.043574 | SKP1 |
| 60626_at | 3.2067 | 0.032692 | 1.4856 | 0.043592 | RIC8A |
| 768211_at | -3.205 | 0.032744 | 1.4849 | 0.04365 | RELL1 |
| 729993_at | -3.2014 | 0.032856 | 1.4834 | 0.043778 | SHISA9 |
| 6050_at | 3.2014 | 0.032857 | 1.4834 | 0.043778 | RNH1 |
| 100506282_at | -3.2005 | 0.032887 | 1.483 | 0.043805 | LOC100506282 |
| 134359_at | -3.195 | 0.033059 | 1.4807 | 0.044023 | POC5 |
| 89853_at | -3.1941 | 0.033088 | 1.4803 | 0.04405 | MVB12B |
| 58528_at | 3.1935 | 0.033105 | 1.4801 | 0.04406 | RRAGD |
| 51614_at | 3.1905 | 0.033201 | 1.4788 | 0.044177 | ERGIC3 |
| 2308_at | -3.1874 | 0.033299 | 1.4776 | 0.044295 | FOXO1 |
| 439_at | 3.1815 | 0.033486 | 1.4751 | 0.044532 | ASNA1 |
| 2643_at | -3.18 | 0.033534 | 1.4745 | 0.044585 | GCH1 |
| 102724861_at | 3.1757 | 0.033674 | 1.4727 | 0.044751 | LOC102724861 |
| 51373_at | 3.1756 | 0.033678 | 1.4727 | 0.044751 | MRPS17 |
| 100507460_at | -3.175 | 0.033697 | 1.4724 | 0.044765 | LOC100507460 |
| 51117_at | 3.1703 | 0.033848 | 1.4705 | 0.044954 | COQ4 |
| 255082_at | 3.168 | 0.033924 | 1.4695 | 0.045042 | CASC2 |
| 143884_at | -3.167 | 0.033954 | 1.4691 | 0.04507 | CWF19L2 |
| 4867_at | 3.1665 | 0.033973 | 1.4689 | 0.045084 | NPHP1 |
| 283254_at | 3.1657 | 0.033999 | 1.4685 | 0.045107 | HARBI1 |
| 3005_at | -3.1653 | 0.034011 | 1.4684 | 0.045111 | H1F0 |
| 158586_at | -3.1638 | 0.034059 | 1.4678 | 0.045162 | ZXDB |
| 224_at | -3.1635 | 0.03407 | 1.4676 | 0.045164 | ALDH3A2 |
| 54898_at | 3.1632 | 0.034079 | 1.4675 | 0.045164 | ELOVL2 |
| 1933_at | 3.1627 | 0.034095 | 1.4673 | 0.045174 | EEF1B2 |
| 9114_at | 3.1606 | 0.034163 | 1.4664 | 0.045252 | ATP6V0D1 |
| 400058_at | 3.157 | 0.034283 | 1.4649 | 0.045399 | MKRN9P |
| 10806_at | -3.1509 | 0.034484 | 1.4624 | 0.045653 | SDCCAG8 |
| 59274_at | 3.1491 | 0.034545 | 1.4616 | 0.045722 | MESDC1 |
| 357_at | -3.1486 | 0.034561 | 1.4614 | 0.045731 | SHROOM2 |
| 56675_at | -3.1458 | 0.034655 | 1.4602 | 0.045842 | NRIP3 |
| 23212_at | 3.145 | 0.034681 | 1.4599 | 0.045865 | RRS1 |
| 29766_at | -3.1443 | 0.034702 | 1.4596 | 0.045881 | TMOD3 |
| 64601_at | 3.1404 | 0.034834 | 1.458 | 0.046043 | VPS16 |
| 100616146_at | 3.1334 | 0.03507 | 1.4551 | 0.046343 | MIR4534 |
| 3601_at | -3.1317 | 0.035126 | 1.4544 | 0.046405 | IL15RA |
| 823_at | -3.1298 | 0.035192 | 1.4536 | 0.046479 | CAPN1 |
| 6713_at | -3.1275 | 0.035271 | 1.4526 | 0.046571 | SQLE |
| 100313771_at | -3.1257 | 0.035331 | 1.4518 | 0.046638 | MIR548F2 |
| 84942_at | 3.1224 | 0.035445 | 1.4505 | 0.046776 | WDR73 |
| 6891_at | -3.1217 | 0.035467 | 1.4502 | 0.046793 | TAP2 |
| 6604_at | 3.1206 | 0.035508 | 1.4497 | 0.046835 | SMARCD3 |
| 221079_at | -3.1199 | 0.03553 | 1.4494 | 0.046851 | ARL5B |
| 90161_at | -3.1153 | 0.035688 | 1.4475 | 0.047048 | HS6ST2 |
| 55791_at | -3.1129 | 0.035773 | 1.4464 | 0.047147 | LRIF1 |
| 8131_at | 3.1089 | 0.035909 | 1.4448 | 0.047314 | NPRL3 |
| 83642_at | 3.108 | 0.035942 | 1.4444 | 0.047345 | SELO |
| 100507487_at | 3.1057 | 0.03602 | 1.4435 | 0.047432 | LOC100507487 |
| 58490_at | -3.1055 | 0.036027 | 1.4434 | 0.047432 | RPRD1B |
| 79663_at | -3.1048 | 0.036051 | 1.4431 | 0.047451 | HSPBAP1 |
| 539_at | 3.1026 | 0.036129 | 1.4421 | 0.047541 | ATP5O |
| 4682_at | 3.1018 | 0.036159 | 1.4418 | 0.047558 | NUBP1 |
| 4234_at | 3.1017 | 0.036161 | 1.4418 | 0.047558 | METTL1 |
| 158747_at | -3.0978 | 0.036297 | 1.4401 | 0.047724 | MOSPD2 |
| 65986_at | -3.0976 | 0.036307 | 1.44 | 0.047725 | ZBTB10 |
| 948_at | -3.0965 | 0.036346 | 1.4395 | 0.047764 | CD36 |
| 84976_at | 3.0929 | 0.036472 | 1.438 | 0.047908 | DISP1 |
| 10476_at | 3.0928 | 0.036475 | 1.438 | 0.047908 | ATP5H |
| 2013_at | -3.0908 | 0.036547 | 1.4372 | 0.047989 | EMP2 |
| 7425_at | 3.0899 | 0.036579 | 1.4368 | 0.048019 | VGF |
| 949_at | 3.0895 | 0.036593 | 1.4366 | 0.048026 | SCARB1 |
| 51192_at | -3.0891 | 0.036607 | 1.4364 | 0.048031 | CKLF |
| 9910_at | -3.082 | 0.036859 | 1.4335 | 0.048349 | RABGAP1L |
| 343990_at | -3.0793 | 0.036957 | 1.4323 | 0.048465 | KIAA1211L |
| 100422901_at | -3.0778 | 0.037012 | 1.4317 | 0.048524 | MIR3135A |
| 10226_at | 3.0771 | 0.037036 | 1.4314 | 0.048543 | PLIN3 |
| 25934_at | 3.0746 | 0.037127 | 1.4303 | 0.048649 | NIPSNAP3A |
| 9124_at | 3.0739 | 0.03715 | 1.43 | 0.048667 | PDLIM1 |
| 84933_at | -3.0724 | 0.037205 | 1.4294 | 0.048726 | C8orf76 |
| 84083_at | 3.0697 | 0.037304 | 1.4282 | 0.048843 | ZRANB3 |
| 89876_at | 3.0661 | 0.037435 | 1.4267 | 0.049002 | MAATS1 |
| 101929115_at | -3.0644 | 0.037497 | 1.426 | 0.04907 | LOC101929115 |
| 9833_at | -3.064 | 0.037513 | 1.4258 | 0.049078 | MELK |
| 2027_at | -3.0616 | 0.037601 | 1.4248 | 0.04918 | ENO3 |
| 26785_at | 3.0601 | 0.037656 | 1.4242 | 0.04924 | SNORD63 |
| 55711_at | -3.0597 | 0.03767 | 1.424 | 0.049245 | FAR2 |
| 118432_at | 3.0541 | 0.037875 | 1.4216 | 0.0495 | RPL29P2 |
| 79660_at | -3.0537 | 0.037891 | 1.4215 | 0.049508 | PPP1R3B |
| 135112_at | -3.0526 | 0.037933 | 1.421 | 0.04955 | NCOA7 |
| 53826_at | -3.0512 | 0.037984 | 1.4204 | 0.049603 | FXYD6 |
| 171423_at | 3.0496 | 0.038043 | 1.4197 | 0.049667 | PDIA3P1 |
| 682_at | 3.0476 | 0.038116 | 1.4189 | 0.04975 | BSG |
| 101927957_at | 3.0459 | 0.038182 | 1.4181 | 0.049823 | LINC01572 |
| 254827_at | -3.0448 | 0.038221 | 1.4177 | 0.04986 | NAALADL2 |
| 100506451_at | 3.0439 | 0.038257 | 1.4173 | 0.049892 | RASSF8-AS1 |
| 6748_at | 3.0437 | 0.038265 | 1.4172 | 0.049892 | SSR4 |
| 339745_at | -3.0432 | 0.038281 | 1.417 | 0.0499 | SPOPL |
| 100422931_at | 3.0401 | 0.038398 | 1.4157 | 0.05004 | MIR4304 |
| 55617_at | 3.0393 | 0.03843 | 1.4153 | 0.050068 | TASP1 |
| 5093_at | 3.0385 | 0.038458 | 1.415 | 0.050092 | PCBP1 |
| 64208_at | -3.0373 | 0.038503 | 1.4145 | 0.050138 | POPDC3 |
| 10856_at | 3.0368 | 0.038524 | 1.4143 | 0.050152 | RUVBL2 |
| 23598_at | -3.033 | 0.038668 | 1.4127 | 0.050325 | PATZ1 |
| 101927027_at | 3.0293 | 0.038807 | 1.4111 | 0.050489 | LOC101927027 |
| 56102_at | 3.0291 | 0.038814 | 1.411 | 0.050489 | PCDHGB3 |
| 6164_at | 3.0248 | 0.038978 | 1.4092 | 0.050689 | RPL34 |
| 100423016_at | 3.0199 | 0.039166 | 1.4071 | 0.050921 | MIR3159 |
| 51175_at | -3.0195 | 0.039184 | 1.4069 | 0.050932 | TUBE1 |
| 79005_at | 3.0189 | 0.039205 | 1.4067 | 0.050945 | SCNM1 |
| 8349_at | -3.0174 | 0.039264 | 1.406 | 0.051009 | HIST2H2BE |
| 133957_at | 3.0143 | 0.039382 | 1.4047 | 0.051149 | CCDC127 |
| 8309_at | 3.0138 | 0.039403 | 1.4045 | 0.051163 | ACOX2 |
| 102724571_at | -3.0133 | 0.039421 | 1.4043 | 0.051173 | LOC102724571 |
| 54873_at | 3.0099 | 0.039554 | 1.4028 | 0.051332 | PALMD |
| 7357_at | -3.0078 | 0.039638 | 1.4019 | 0.051428 | UGCG |
| 55632_at | -3.0058 | 0.039713 | 1.4011 | 0.051512 | G2E3 |
| 22929_at | 3.0055 | 0.039724 | 1.4009 | 0.051513 | SEPHS1 |
| 28973_at | 3.0025 | 0.039844 | 1.3996 | 0.051646 | MRPS18B |
| 80271_at | -3.0024 | 0.039848 | 1.3996 | 0.051646 | ITPKC |
| 114801_at | 2.9966 | 0.040078 | 1.3971 | 0.051931 | TMEM200A |
| 284391_at | 2.9959 | 0.040103 | 1.3968 | 0.051947 | ZNF844 |
| 554279_at | -2.9957 | 0.040111 | 1.3967 | 0.051947 | LINC00862 |
| 284371_at | -2.9931 | 0.040213 | 1.3956 | 0.052066 | ZNF841 |
| 84668_at | -2.9859 | 0.040501 | 1.3925 | 0.052424 | FAM126A |
| 4282_at | 2.9833 | 0.040606 | 1.3914 | 0.052547 | MIF |
| 10933_at | -2.9817 | 0.040672 | 1.3907 | 0.052619 | MORF4L1 |
| 221656_at | -2.9801 | 0.040734 | 1.39 | 0.052686 | KDM1B |
| 6309_at | -2.9792 | 0.04077 | 1.3897 | 0.052719 | SC5D |
| 54718_at | -2.9778 | 0.040827 | 1.3891 | 0.052779 | BTN2A3P |
| 80115_at | -2.9747 | 0.040953 | 1.3877 | 0.052927 | BAIAP2L2 |
| 27089_at | 2.9714 | 0.041088 | 1.3863 | 0.053088 | UQCRQ |
| 540_at | 2.9692 | 0.041176 | 1.3854 | 0.053189 | ATP7B |
| 5368_at | 2.9677 | 0.041237 | 1.3847 | 0.053234 | PNOC |
| 3232_at | 2.9677 | 0.041237 | 1.3847 | 0.053234 | HOXD3 |
| 221322_at | -2.9676 | 0.041243 | 1.3846 | 0.053234 | TBC1D32 |
| 151877_at | -2.9625 | 0.041452 | 1.3824 | 0.053485 | MAGI1-IT1 |
| 101929381_at | 2.9623 | 0.041459 | 1.3824 | 0.053485 | LOC101929381 |
| 55558_at | 2.9579 | 0.041641 | 1.3805 | 0.053705 | PLXNA3 |
| 84188_at | -2.9547 | 0.041773 | 1.3791 | 0.053862 | FAR1 |
| 100422895_at | 2.9529 | 0.041848 | 1.3783 | 0.053945 | MIR4294 |
| 6591_at | 2.9499 | 0.041974 | 1.377 | 0.054094 | SNAI2 |
| 5136_at | 2.9486 | 0.042028 | 1.3765 | 0.054149 | PDE1A |
| 66035_at | 2.9482 | 0.042046 | 1.3763 | 0.054158 | SLC2A11 |
| 360023_at | -2.9461 | 0.042131 | 1.3754 | 0.054254 | ZBTB41 |
| 22856_at | 2.9436 | 0.042237 | 1.3743 | 0.054377 | CHSY1 |
| 28957_at | 2.9424 | 0.042286 | 1.3738 | 0.054425 | MRPS28 |
| 283392_at | -2.9372 | 0.042506 | 1.3715 | 0.054694 | TRHDE-AS1 |
| 26973_at | -2.9357 | 0.04257 | 1.3709 | 0.054763 | CHORDC1 |
| 6280_at | -2.9338 | 0.042653 | 1.3701 | 0.054855 | S100A9 |
| 6936_at | -2.9325 | 0.042706 | 1.3695 | 0.054909 | GCFC2 |
| 114882_at | -2.9265 | 0.042965 | 1.3669 | 0.055228 | OSBPL8 |
| 60496_at | 2.926 | 0.042985 | 1.3667 | 0.05524 | AASDHPPT |
| 3006_at | 2.9248 | 0.043038 | 1.3662 | 0.055293 | HIST1H1C |
| 84515_at | -2.9243 | 0.04306 | 1.3659 | 0.055304 | MCM8 |
| 100874364_at | 2.9241 | 0.043068 | 1.3658 | 0.055304 | HOXC-AS2 |
| 2055_at | 2.9218 | 0.043165 | 1.3649 | 0.055406 | CLN8 |
| 3956_at | 2.9217 | 0.04317 | 1.3648 | 0.055406 | LGALS1 |
| 5737_at | 2.9208 | 0.043208 | 1.3644 | 0.055441 | PTGFR |
| 619564_at | -2.9201 | 0.043239 | 1.3641 | 0.055466 | SNORD72 |
| 54606_at | 2.9195 | 0.043266 | 1.3639 | 0.055486 | DDX56 |
| 84294_at | -2.9173 | 0.043362 | 1.3629 | 0.055595 | UTP23 |
| 100113382_at | 2.903 | 0.043985 | 1.3567 | 0.05638 | SNORD105B |
| 7813_at | -2.9016 | 0.044045 | 1.3561 | 0.056442 | EVI5 |
| 56969_at | 2.8979 | 0.044209 | 1.3545 | 0.056638 | RPL23AP32 |
| 9997_at | 2.8963 | 0.044282 | 1.3538 | 0.056708 | SCO2 |
| 84131_at | -2.8962 | 0.044287 | 1.3537 | 0.056708 | CEP78 |
| 54902_at | -2.8889 | 0.044613 | 1.3505 | 0.057112 | TTC19 |
| 7011_at | -2.886 | 0.04474 | 1.3493 | 0.05726 | TEP1 |
| 10224_at | -2.8834 | 0.044858 | 1.3482 | 0.057396 | ZNF443 |
| 497190_at | 2.876 | 0.045196 | 1.3449 | 0.057814 | CLEC18B |
| 91978_at | 2.8755 | 0.045217 | 1.3447 | 0.057825 | TPGS1 |
| 10548_at | 2.8753 | 0.045229 | 1.3446 | 0.057826 | TM9SF1 |
| 8907_at | 2.8709 | 0.045427 | 1.3427 | 0.058065 | AP1M1 |
| 9238_at | 2.8698 | 0.045478 | 1.3422 | 0.058115 | TBRG4 |
| 728963_at | -2.8673 | 0.045592 | 1.3411 | 0.058245 | RPS15AP10 |
| 406890_at | -2.8648 | 0.045705 | 1.34 | 0.058375 | MIRLET7G |
| 2135_at | 2.862 | 0.045839 | 1.3388 | 0.058531 | EXTL2 |
| 25864_at | 2.8582 | 0.046011 | 1.3371 | 0.058727 | ABHD14A |
| 3575_at | 2.8581 | 0.046016 | 1.3371 | 0.058727 | IL7R |
| 28974_at | 2.8497 | 0.046411 | 1.3334 | 0.059217 | C19orf53 |
| 9349_at | 2.8488 | 0.046454 | 1.333 | 0.059256 | RPL23 |
| 148808_at | -2.844 | 0.046676 | 1.3309 | 0.059524 | MFSD4 |
| 7102_at | 2.8428 | 0.046734 | 1.3304 | 0.059583 | TSPAN7 |
| 4883_at | 2.8296 | 0.047366 | 1.3245 | 0.060373 | NPR3 |
| 29886_at | 2.8283 | 0.047427 | 1.324 | 0.060436 | SNX8 |
| 3856_at | 2.8243 | 0.04762 | 1.3222 | 0.060666 | KRT8 |
| 1138_at | -2.8229 | 0.04769 | 1.3216 | 0.060739 | CHRNA5 |
| 54101_at | -2.8204 | 0.047809 | 1.3205 | 0.060876 | RIPK4 |
| 80273_at | 2.8197 | 0.047841 | 1.3202 | 0.060898 | GRPEL1 |
| 9015_at | -2.8195 | 0.047851 | 1.3201 | 0.060898 | TAF1A |
| 746_at | 2.8171 | 0.047968 | 1.319 | 0.061032 | TMEM258 |
| 100302170_at | -2.8152 | 0.048062 | 1.3182 | 0.061135 | MIR1206 |
| 9994_at | -2.8146 | 0.048093 | 1.3179 | 0.06116 | CASP8AP2 |
| 26872_at | 2.8138 | 0.048131 | 1.3176 | 0.061192 | STEAP1 |
| 5959_at | 2.8116 | 0.048238 | 1.3166 | 0.061295 | RDH5 |
| 5283_at | 2.8115 | 0.048244 | 1.3166 | 0.061295 | PIGH |
| 9754_at | 2.8114 | 0.048249 | 1.3165 | 0.061295 | STARD8 |
| 375318_at | 2.8078 | 0.048428 | 1.3149 | 0.061507 | AQP12A |
| 117584_at | 2.8022 | 0.048703 | 1.3124 | 0.06184 | RFFL |
| 4882_at | 2.7965 | 0.048987 | 1.3099 | 0.062185 | NPR2 |
| 729737_at | -2.7943 | 0.049097 | 1.3089 | 0.062309 | LINC01002 |
| 60592_at | -2.7936 | 0.04913 | 1.3087 | 0.062335 | SCOC |
| 6387_at | 2.7929 | 0.049165 | 1.3083 | 0.062364 | CXCL12 |
| 8819_at | 2.7917 | 0.049228 | 1.3078 | 0.062428 | SAP30 |
| 100506649_at | 2.7883 | 0.049396 | 1.3063 | 0.062618 | PXN-AS1 |
| 142891_at | -2.7882 | 0.049403 | 1.3062 | 0.062618 | SAMD8 |
| 374969_at | 2.7846 | 0.049587 | 1.3046 | 0.062836 | CCDC23 |
| 27189_at | -2.7837 | 0.049629 | 1.3043 | 0.062873 | IL17C |
| 126370_at | -2.7832 | 0.049657 | 1.304 | 0.062892 | OR1I1 |
| 160_at | 2.7785 | 0.049893 | 1.302 | 0.063175 | AP2A1 |
