# Supplementary material for: Role of HRTPT in kidney proximal epithelial cell regeneration: Integrative differential expression and pathway analyses using microarray and scRNA‐seq
Source: J Cell Mol Med. 2021 Oct 9;25(22):10466–79. doi: 10.1111/jcmm.16976 (PMC8581341; doi:10.1111/jcmm.16976)
Supplement: Supplementary file 11 — Table S6. A list of pathways associated with differently expressed genes between CD133+ versus CD133‐ Infant Kidney [file JCMM-25-10466-s002.docx]

| **Table S6.** CD133+ versus CD133- Infant Kidney  A Gene Group (David) | | |
| --- | --- | --- |
| NA | | |
| B Pathways (Reactome) | | |
| Pathway | P value |  |
| 1 | 8.30E-05 | Activation of gene expression by SREBF (SREBP) |
| 2 | 1.00E-03 | Regulation of cholesterol biosynthesis by SREBP (SREBF) |
| 3 | 9.00E-03 | Interleukin-10 signaling |
| 4 | 1.00E-02 | Anchoring of the basal body to the plasma membrane |
| 5 | 1.70E-02 | PPARA activates gene expression |
| 6 | 2.10E-02 | Regulation oflipid metabolism by PPARalpha |
| 7 | 3.70E-02 | RAF-independent MAPK1/3 activation |
| 8 | 3.80E-02 | Acyl chain remodeling of PS |
| 9 | 3.90E-02 | Activation of HOX genes duringdifferentiation |
| 10 | 3.90E-02 | Activation of anterior HOX genes in hindbrain |
| C Top Canonical Pathways (Ingenuity) | | |
| Pathway | P value |  |
| 1 | 2.22E-07 | Xenobiotic metabolism signaling |
| 2 | 9.92E-06 | Hepatic fibrosis. Hepatic stellate cell activation |
| 3 | 1.45E-05 | Hepatic fibrosis signaling pathway |
| 4 | 2.34E-05 | LPS/IL-1 mediated inhibition of RXR function |
| 5 | 6.14E-05 | Osteoarthritis pathway |
| D Top Molecular and Cellular Functions (Ingenuity) | | |
| Pathway | P value |  |
| 1 | 2.45E-10-1.27E-41 | Cell death and survival |
| 2 | 3.10E-10-1.01E-38 | Cellular movement |
| 3 | 1.78E-10 – 1.20E-28 | Cell assembly and organization |
| 4 | 1.78E-10 - 1.20E-28 | Cell function and maintenance |
| 5 | 2.28E-10 – 3.02E-22 | Cellular development |
| E Top Upstream Regulators (Ingenuity) | | |
| Pathway | P value |  |
| 1 | 1.18E-40 | Dexamethasone |
| 2 | 1.32E-39 | TGFB1 |
| 3 | 8.96E-39 | TNF |
| 4 | 2.12E-36 | Beta-estradiol |
| 5 | 1.66E-35 | ESR1 |
