# Supplementary material for: Role of HRTPT in kidney proximal epithelial cell regeneration: Integrative differential expression and pathway analyses using microarray and scRNA‐seq
Source: J Cell Mol Med. 2021 Oct 9;25(22):10466–79. doi: 10.1111/jcmm.16976 (PMC8581341; doi:10.1111/jcmm.16976)
Supplement: Supplementary file 12 — Table S7. A list of pathways associated with differently expressed genes between HRTPT Gene Set versus CD133+ Infant Kidney Gene Set [file JCMM-25-10466-s011.docx]

| **Table S7**. HRTPT Gene Set versus CD133+ Infant Kidney Gene Set  A Gene Functional Groups (David) | | |
| --- | --- | --- |
| Group | Score |  |
| 1 | 3.28 | Cell Membrane Organization and Transport: SYT14, FAXDC2, SLCA2, TPCN1, GOLM1, TM4SF4, RXFP1, KCNJ15, SLCA5, GLT8D2, PMP22, CDH6, SLC17A3, CLDN2, SLC43A1, PTH1R, CALCRL, PAQR5 UPK18, TSPAN1, PMEPA1, NLG4AY, GPRC5A, XXR4, ORMDL2, SLC34A2, LHFP, SLC20A1, TMEM154, EMP3, EMP2, TMEM2, SLC17A1, AJAP1, CD47, SLC15A2, SLC15A1, PROM1, TMEM14A, SLC16A4, SLC7A11 |
| 2 | 3.24 | Glycosylation: GLT8D2, ST3GAL1, UGT2A3, ABO, B4GALT5, TMEM154, B4GALT6, UGCG, CHST9, ST6GALNAC1, GCNT1, UPK1B, TSPAN15, ST8SA4, TM4SF4, GALNT14, TMED5, GOLM1 |
| 3 | 2.52 | Wnt Interactions: HAVCR1, VTCN1, TMEM154, CD47, ROR1 |
| 4 | 2.40 | lntegrins: ITGA2, lTGA1, ITGB3, TTGA6, ITGB8 |
| 5 | 1.74 | Endosomal Processing and Trafficking: AGA, LGM, CTSS, CTSB |
| 6 | 1.36 | Interferon Signaling: RND3, GBP2, RAP2B, RAB27B |
| B Pathways (reactome) | | |
| Pathway | P value |  |
| 1 | 0.002 | O-linked glycosyation |
| 2 | 0.002 | Defective B3GALTL causes Peters-plus syndrome |
| 3 | 0.002 | O-glycosylation ofTSR domain containing proteins |
| 4 | 0.005 | Dissolution of fibrin clot |
| 5 | 0.007 | Laminin interactions |
| C Top Canonical Pathways (Ingenuity) | | |
| Pathway | P value |  |
| 1 | 1.94E-05 | Granulocyte adhesion and diapedesis |
| 2 | 2.26E-05 | Leckocyte extravasation and signaling |
| 3 | 7.65E-05 | Virus entry via endocytic pathways |
| 4 | 1.43E-04 | Role of tissue factor in cancer |
| 5 | 1.52E-04 | Putrescine degradation |
| D Molecular and Cellular Functions (Ingenuity) | | |
| Pathway | P value |  |
| 1 | 2.77E-04 - 9.88E-14 | Cellular movement |
| 2 | 2.77E-04 – 3.03E-l l | Cellular development |
| 3 | 8.62E-05 – 3.03E-l l | Cell growth and proliferation |
| 4 | 2.98E-04 – 2.05E-10 | Cell-to-cell signaling and interaction |
| 5 | 3.09E-04 – 9.04E-08 | Cell death and survival |
| E Top Upstream Regulators (Ingenuity) | | |
| Pathway | P value |  |
| 1 | 4.45E-18 | Beta-estradiol |
| 2 | 7.23E- 15 | Tretinoin |
| 3 | 9.54E-13 | Dexamethasone |
| 4 | 1.60E-12 | TGFB1 |
| 5 | 4.65E-12 | MYC |
