# Supplementary material for: Role of HRTPT in kidney proximal epithelial cell regeneration: Integrative differential expression and pathway analyses using microarray and scRNA‐seq
Source: J Cell Mol Med. 2021 Oct 9;25(22):10466–79. doi: 10.1111/jcmm.16976 (PMC8581341; doi:10.1111/jcmm.16976)
Supplement: Supplementary file 13 — Table S8. T‐test outcome of PROM1 and PROM2 across HRTPT, CD133+ infant kidney & udRPCs vs hREPCs datasets [file JCMM-25-10466-s008.docx]

**Table S8**. p-value comparison for PROM1 and PROM2 across HRTPT, CD133+ infant kidney & udRPCs vs hREPCs datasets

| gene | HRTPT | CD133+ infant kidney | udRPCs vs hREPCs |
| --- | --- | --- | --- |
| PROM1 | t= -101.72 (P=0.000000056) | t = -37.487 (P=0.000003024) | t = -1.5453 (P=0.1662) |
| PROM2 | t = 0.89966(P=0.4196) | t = -9.3178 (P=0.00073837) | t = 6.425 (P=0.0003587) |

| t= t stastistics |
| --- |
| P = P value |
