# Supplementary material for: Role of HRTPT in kidney proximal epithelial cell regeneration: Integrative differential expression and pathway analyses using microarray and scRNA‐seq
Source: J Cell Mol Med. 2021 Oct 9;25(22):10466–79. doi: 10.1111/jcmm.16976 (PMC8581341; doi:10.1111/jcmm.16976)
Supplement: Supplementary file 14 — Table S9. A list of pathways associated with differently expressed genes between HRTPT Gene Set vs CD133+ Infant Kidney Gene Set vs African_ udRPCs vs hREPCs Gene Set [file JCMM-25-10466-s004.docx]

| **Table S9**. HRTPT Gene Set vs CD133+ Infant Kidney Gene Set vs African_ udRPCs vs hREPCs Gene Set  A Gene Functional Groups (David) | | |
| --- | --- | --- |
| Group | Score | Genes |
| 1 | 2.31 | Cell Surface/Receptors: TSPAN1, GLT8D2, UGT2A3, EMP1, AJAP1, RXFP1, TMEM2, GPRC5A, TMEM154, HAVCR1, PMP22, UPK1B, VTCN1, TSPAN15, TM4SF4, PROM1, CLDN2, SLC16A4, CALCRL |
| 2 | 1.62 | lntegrins: ITGA2, ITGB3, ITGA6, ITGB8 |
| B Pathways (Reactome) | | |
| Pathway | p value |  |
| 1 | 4.85E-06 | Dissolution of Fibrin Clot |
| 2 | 5.12E-05 | Extracellular Matrix Organization |
| 3 | 2.29E-04 | Molecules Associated with Elastic Fibers |
| 4 | 4.68E-04 | Elastic Fiber Formation |
| 5 | 1.00E-03 | Non-lntegrin Membrane - ECM Interactions |
| 6 | 2.00E-03 | Syndecan Interactions |
| 7 | 2.00E-03 | Laminin Interactions |
| 8 | 2.00E-03 | Assembly of Collagen Fibrils and Other Multimeric Structures |
| 9 | 3.00E-03 | Type 1 Hemidesmosome Assembly |
| 10 | 5.00E-03 | Negative Regulation of Activity TFAP2 (AP2) Family Transcription Factors |
| 11 | 5.00E-03 | Integrin Surface Interactions |
| 12 | 6.00E-03 | Cell Junctional Organization |
| C Top Canonical Pathways (Ingenuity) | | |
| Pathway | p value |  |
| 1 | 1.49E-03 | PXR/RXR Activation |
| 2 | 3.23E-03 | Apelin Cardiac Fibroblast Signalling Pathway |
| 3 | 3.61E-03 | Wnt/-Catenin Signalling |
| 4 | 4.00E-03 | Tumor Microenvironment Pathway |
| 5 |  | Sertoli Cell - Sertoli Cell Junctional signalling |
| D Molecular and Cellular Functions (Ingenuity) | | |
| Pathway | p value |  |
| 1 | 4.53E-03 - 2.77E-06 | Small Molecule Biochemistry |
| 2 | 4.22E-03 - 3.71E-06 | Cellular Movement |
| 3 | 3.69E-03 - 2.12E-05 | Amino Acid Metabolism |
| 4 | 4.53E-03 - 2.12E-05 | Molecular Transport |
| 5 | 4.44E-03 - 3.06E-05 | Cell-to-Cell Signallig and Interaction |
| E Top Upstream Regulators (Ingenuity) | | |
| Pathway | p value |  |
| 1 | 1.15E-06 | Beta-Estradiol |
| 2 | 2.94E-06 | Dexamethasone |
| 3 | 5.24E-06 | Decitabine |
| 4 | 1.21E-05 | NSUN6 |
| 5 | 2.80E-05 | ESR1 |
